# Assessing the Quality of AI Responses to Patient Concerns About Axial Spondyloarthritis: Delphi-Based Evaluation

**DOI:** 10.2196/79153

**Published:** 2026-01-07

**Authors:** Jiaxin Bai, Xiaojian Ji, Jiali Yu, Yiwen Wang, Yufei Guo, Chao Xue, Wenrui Zhang, Jian Zhu

**Affiliations:** 1 Medical School of Chinese People's Liberation Army Beijing China; 2 Department of Rheumatology and Immunology The First Medical Center Chinese People's Liberation Army General Hospital Beijing China; 3 State Key Laboratory of Kidney Diseases, Chinese People's Liberation Army General Hospital Beijing China

**Keywords:** axial spondyloarthritis, axSpA, artificial intelligence, AI, large language model, health management, chronic disease

## Abstract

**Background:**

Axial spondyloarthritis (axSpA) is a chronic autoinflammatory disease with heterogeneous clinical features, presenting considerable complexity for sustained patient self-management. Although the use of large language models (LLMs) in health care is rapidly expanding, there has been no rigorous assessment of their capacity to provide axSpA-specific health guidance.

**Objective:**

This study aimed to develop a patient-centered needs assessment tool and conduct a systematic evaluation of the quality of LLM-generated health advice for patients with axSpA.

**Methods:**

A 2-round Delphi consensus process guided the design of the questionnaire, which was subsequently administered to 84 patients with axSpA and 26 rheumatologists. Patient-identified key concerns were formulated and input into 5 LLM platforms (GPT-4.0, DeepSeek R1, Hunyuan T1, Kimi k1.5, and Wenxin X1), with all prompts and model outputs in Chinese. Responses were evaluated using 2 techniques: an accuracy assessment based on guideline concordance, with independent double blinding by 2 raters (interrater reliability analyzed via Cohen κ), and the AlphaReadabilityChinese analytic tool to assess readability.

**Results:**

Analysis of the validated questionnaire revealed age-related differences. Patients younger than 40 years prioritized symptom management and medication side effects more than those older than 40 years. Distinct priorities between clinicians and patients were identified for diagnostic mimics and drug mechanisms. LLM accuracy was highest in the diagnosis and examination category (mean score 20.4, SD 0.9) but lower in treatment and medication domains (mean score 19.3, SD 1.7). GPT-4.0 and Kimi k1.5 demonstrated superior overall readability; safety remained generally high (disclaimer rates: GPT-4.0 and DeepSeek-R1 100%; Kimi k1.5 88%).

**Conclusions:**

Needs assessment across age groups and observed divergences between clinicians and patients underline the necessity for customized patient education. LLMs performed robustly on most evaluation metrics, and GPT-4.0 achieved 94% overall agreement with clinical guidelines. These tools hold promise as scalable adjuncts for ongoing axSpA support, provided complex clinical decision-making remains under human oversight. Nevertheless, the prevalence of artificial intelligence hallucinations remains a critical barrier. Only through comprehensive mitigation of such risks can LLM-based medical support be safely accelerated.

## Introduction

Axial spondyloarthritis (axSpA) is a chronic inflammatory disorder that predominantly affects the sacroiliac and axial spinal joints. Early symptoms often include chronic atypical low back pain and morning stiffness, with associated manifestations such as tendinitis and arthritis and extra-articular features such as uveitis, inflammatory bowel disease, and psoriasis frequently observed [1]. Despite substantial research progress on axSpA, most studies have been disease centered, with limited focus on patient-oriented assessment. The insidious onset and nonspecific symptoms frequently contribute to delays in recognition and care. Accurate diagnosis requires the integration of clinical signs; laboratory results; and imaging, such as pelvic X-ray or sacroiliac joint magnetic resonance imaging [2]. Many patients lack a clear understanding of the necessity or implications of these examinations. Therapeutic approaches for axSpA encompass both pharmacological and nonpharmacological strategies [3,4], posing additional challenges regarding patient decision-making and informed participation in care. These factors collectively impact axSpA self-management and highlight the urgent need for enhanced patient education. Furthermore, the rapid advancement of large language models (LLMs) has unlocked considerable health care potential [5,6]. As more patients seek advice from artificial intelligence (AI)–based systems, it remains essential to rigorously evaluate the accuracy and quality of medical guidance they provide within axSpA-related contexts.

This study aimed to systematically identify genuine concerns of patients with axSpA via a questionnaire survey and a parallel analysis of the perspectives from clinicians. Patient-derived questions were presented to LLMs, with resulting health advice assessed across 3 dimensions: readability, accuracy, and health disclaimer. These findings offer data-driven insight for clinicians, enabling them to tailor education to the needs and cognitive patterns of diverse patient populations. The results further inform evaluation of LLMs in health counseling, support more nuanced clinical decision-making in diagnosis and treatment, and guide the development of sustainable patient-centered management strategies.

## Methods

### Construction of the Questionnaire

The questionnaire development comprised 3 stages [7,8]. Initially, a comprehensive list of knowledge items was extracted from published questionnaires and the 2022 Assessment of Spondyloarthritis International Society–European Alliance of Associations for Rheumatology recommendations for axSpA management. A Delphi process included rheumatologists, rheumatology graduate students, and patients. They first enriched the list by adding items considered potentially useful, and then the list was reduced to obtain the most important items. Participants in the Delphi rounds were enrolled from the department of rheumatology and immunology of the Chinese PLA General Hospital First Medical Center. The rheumatologists and the rheumatology graduate students invited patients to participate.

In the second stage, the initial version of the questionnaire was created based on the first Delphi round results, formulated by XJ, JB, and JY. Each question was mapped to the extracted item list to ensure comprehensive coverage of clinical features, diagnosis, examination methods, medication options, and prognosis related to axSpA. The instrument was designed for all patients with axSpA features regardless of concomitant peripheral SpA, psoriasis, or inflammatory bowel disease manifestations.

In the third stage, the final Delphi round facilitated consensus among all rheumatology experts and rheumatology graduate students to refine the instrument, with questions selected as essential if chosen by more than two-thirds and useful if chosen by more than half but less than two-thirds of participants. Items deemed redundant and overly complex or those lacking clinical relevance were eliminated, resulting in the finalized version. The questionnaire structure and corresponding item numbers are provided in Multimedia Appendix 1.

### Data Collection and Analysis

For data collection, the finalized questionnaire was digitized and formatted into an online survey. An additional section at its conclusion collected basic demographic and health-related information to support baseline analysis. Participation was anonymous, with clear disclosure that responses would be used solely for research purposes. Recruitment used a Wenjuanxing (an online survey platform) link, and this link was distributed through hospital outpatient clinics [9]. The collected data were categorized and contrasted according to the baseline characteristics of the respondents, including patient age, sex, and occupational category.

To compare differences in attitudes between health care professionals and patients, a separate online survey was administered to medical staff within the rheumatology and immunology department.

### Choice of LLM Chatbots

In selecting LLMs, we included DeepSeek R1 (DeepSeek), Hunyuan T1 (Tencent), Kimi k1.5 (Moonshot AI), Wenxin X1 (Baidu), and GPT-4.0 (OpenAI) [10-13], each possessing strengths in different domains. The comprehensive comparison of these models was intended to more accurately reflect real-world choices and user experiences among patients with axSpA.

### Outcomes and Data Synthesis

The LLM-generated answers were systematically collected by a researcher and organized into bullet points. Each question was submitted independently to the models in a 1-time format to prevent AI memory effects and ensure unbiased responses. Both the patient queries and all LLM outputs were generated in Chinese. Full datasets are provided in Multimedia Appendix 2. Response assessment targeted 3 metrics: accuracy, readability, and health advice disclaimers. Accuracy was defined as the degree of correctness in each LLM’s response to individual items [6-14] benchmarked against the 2022 Assessment of Spondyloarthritis International Society–European Alliance of Associations for Rheumatology guidelines and the Lancet series recommendations [4,15-19]. Two independent raters assessed each suggestion based on a published scoring criterion (Multimedia Appendix 3), with arbitration by a third researcher in case of discrepancies. For example, for scoring, if rater A assigned indicator scores of 4, 3, 3, and 1 and rater B assigned scores of 4, 4, 3, and 1, the raters would discuss any discrepancies (here for the second indicator, 3 vs 4). Irreconcilable differences were resolved by an expert’s decision. The independent raters acknowledged potential subjective bias favoring AI, possibly leading to higher average ratings than seen in previous literature. Interrater reliability was quantified via the Cohen κ statistic.

Readability was defined as the ease or difficulty of reading each text and quantitatively measured using the AlphaReadabilityChinese tool (Shanghai International Studies University) [20]. This analytic framework assesses 9 dimensions of language complexity. Higher scores in some dimensions signal increased reading difficulty, whereas, for the 5 “precision and clarity” dimensions, higher scores equate to better comprehension (Textbox 1).

The key takeaway was that easier-to-understand texts scored low on dimensions of complexity, such as intricate vocabulary and sentence structure, but high on dimensions of precision and clarity, including the use of specific words and unambiguous phrasing.

“Health disclaimers” were defined as warnings within the response that cautioned about specific risks or promoted appropriate and safe patient behaviors, such as recommending medical attention if symptoms persist. Each LLM response was categorized on the basis of the presence or absence of a health disclaimer [21]. The scope of disclaimers encompassed recommendations to seek professional assistance, urgent care, careful medication use, and general consultative language.

**Dimensions of readability.**

**Dimensions where higher scores mean the text is harder to read**

- Lexical richness indicates the use of diverse and complex vocabulary.
- Syntactic richness refers to longer and structurally intricate sentences.
- Semantic richness reflects a high density of content and information.
- Semantic noise represents the presence of redundant or off-topic information that may obscure the main message.

**Dimensions where higher scores mean the text is easier to read**

- Noun or verb precision captures the use of specific nouns and action verbs (eg, “MRI scan” instead of “a type of examination” and “reduce pain” instead of “implement analgesic measures”).
- Semantic clarity measures how directly and unambiguously information is conveyed.

### Statistical Analysis

Statistical analyses were conducted using R (version 3.4.0; R Foundation for Statistical Computing) and RStudio (version 1.0.136; Posit PBC). Assumptions of normality and variance homogeneity informed the use of either ANOVA or Kruskal-Wallis tests for multiple group comparisons of language-difficulty metrics [22,23]; Greenhouse-Geisser or Satterthwaite corrections were applied as needed [24,25]. Categorical data from questionnaire responses were evaluated using chi-square tests or Fisher exact test, where applicable [26,27]. Significance was defined at *P*<.05. Figures were plotted using the *ggplot2* R package.

### Ethical Considerations

Before the first Delphi round, this study was approved by the medical ethics committee of Chinese People’s Liberation Army General Hospital (S2022-255-03). For patients completing the paper-based questionnaire, a dedicated informed consent form was signed to obtain their consent. For those completing the electronic questionnaire, informed consent was obtained through the “check + click button” method—patients were required to check the box and click the confirmation button to verify that they had read and agreed to all terms. During the data collection process, we ensured patient privacy and maintained strict confidentiality of patient data. No compensation was provided to patients for their participation.

## Results

### Construction of the Questionnaire

At the first stage, 31 items were extracted from existing survey instruments. Delphi rounds incorporated 1 senior rheumatology expert with more than 30 years of experience, 3 rheumatologists with extensive clinical expertise, 5 rheumatology graduate students, and 8 patients. The first Delphi round expanded the preliminary list to 50 potentially informative items. In the next stage, a graduate student reformulated these into specific questions and compiled them into a draft questionnaire. The final Delphi round selected 42 questions judged “essential” by more than half (9/17, 53%) of the participants. Figure S1 in Multimedia Appendix 4 provides a detailed flowchart of these procedures.

### Survey Results

**Figure 1:**
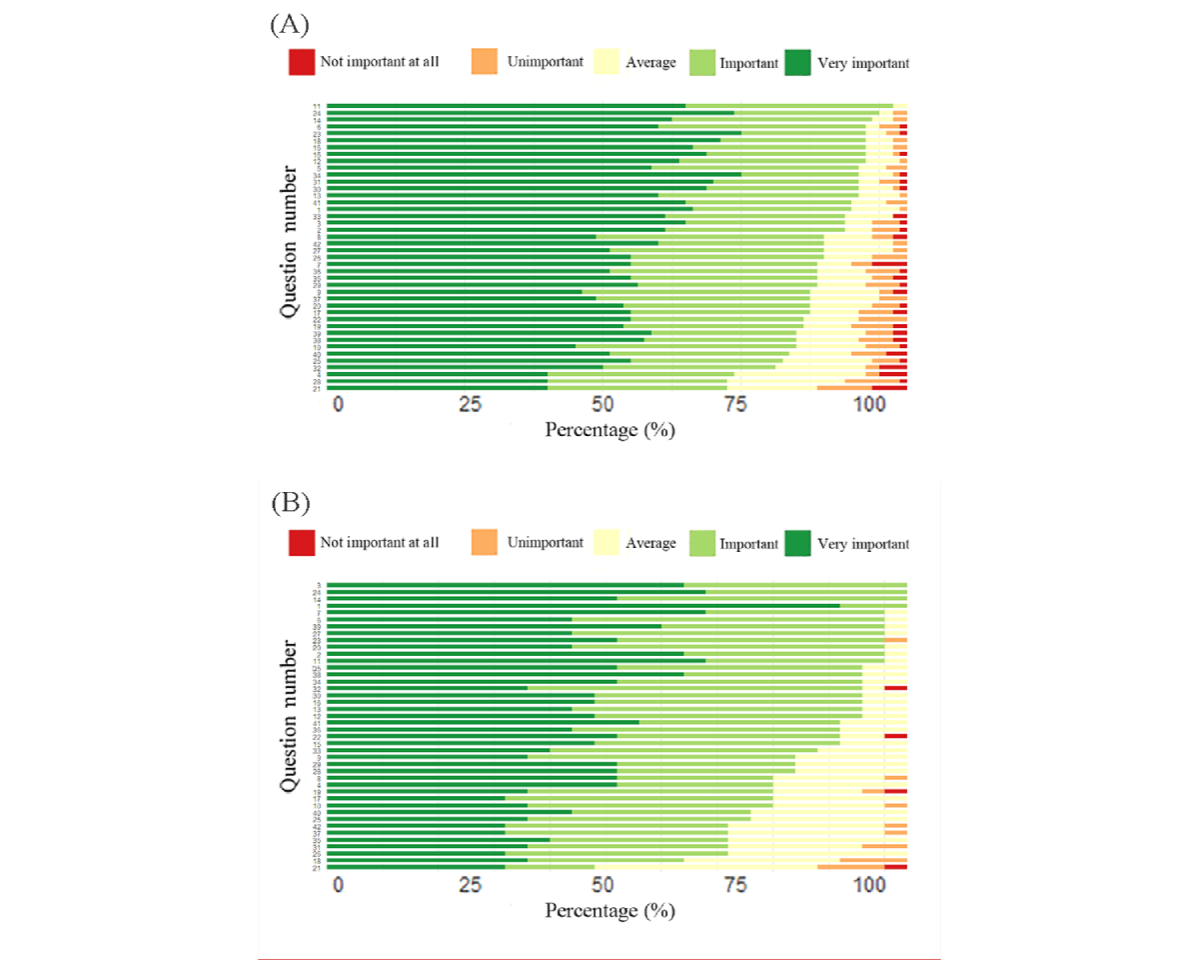
Questionnaire responses from patients and rheumatologists. (A) Patient questionnaire responses. The lengths of the differently colored bars represent the proportion of respondents who selected each option within the total surveyed population. (B) Rheumatologists’ questionnaire responses.

Through the online questionnaire, responses were collected from 84 patients with axSpA. Demographic details and response distributions are presented in Figure 1A and Table 1. The cohort comprised 62 (74%) men and 22 (26%) women, with an average age of 38.01 (SD 10.45) years. Education levels were predominantly bachelor’s degree (n=34, 40%), followed by senior high school (n=24, 29%) and master’s or higher degrees (n=13, 15%). Most (n=47, 56%) held sedentary occupations. Parental health status was most often reported as “good” (n=57, 68%), while self-assessed health was frequently rated as “fair” (n=42, 50%). Family history of ankylosing spondylitis was identified in 27 (32%) participants. In total, 57 (68%) participants used the internet for less than 6 hours a day, and 27 (32%) participants exceeded this threshold. Figure 1A shows that question 11 (“My doctor recommended testing for HLA-B27. What does a positive result mean?”) was the area of greatest concern. To expand the scope of assessment, 26 responses from health care professionals were gathered (Figure 1B), with question 11 also ranking highly in this group. Health care professionals unanimously identified question 1, question 3, question 14, and question 24 as highly important, with no respondents rating them as “neutral,” “unimportant,” or “very unimportant.”

**Figure 2:**
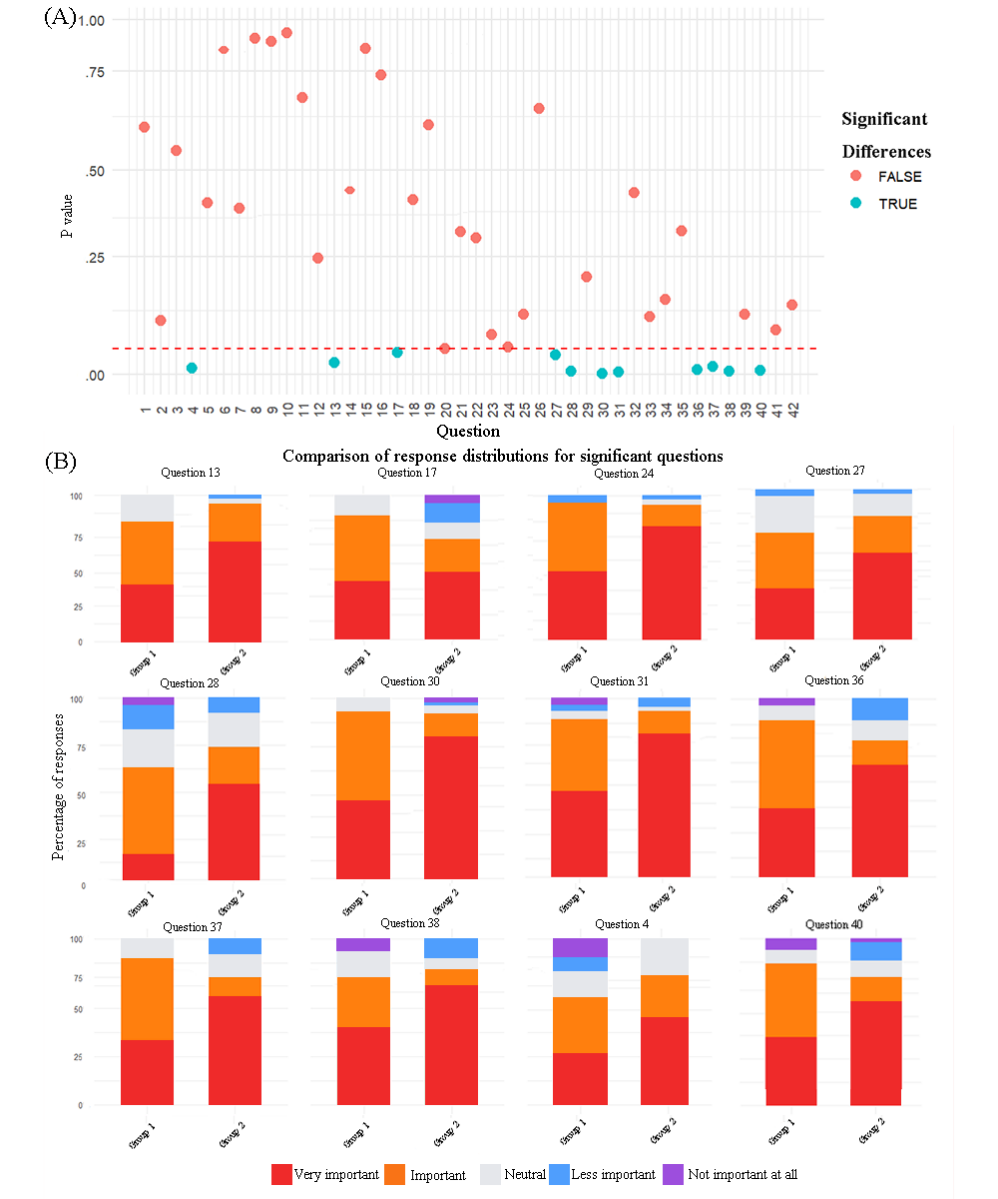
Age-stratified response discrepancy distribution. (A) Scatter points below the red dashed line indicate *P*<.05, suggesting statistically significant differences in answer choices among different age groups for the specific question. (B) Each color block represents the proportion of respondents who selected that option relative to the total. Group 1 was composed of patients older than 40 years, and group 2 was composed of patients younger than 40 years.

**Figure 3:**
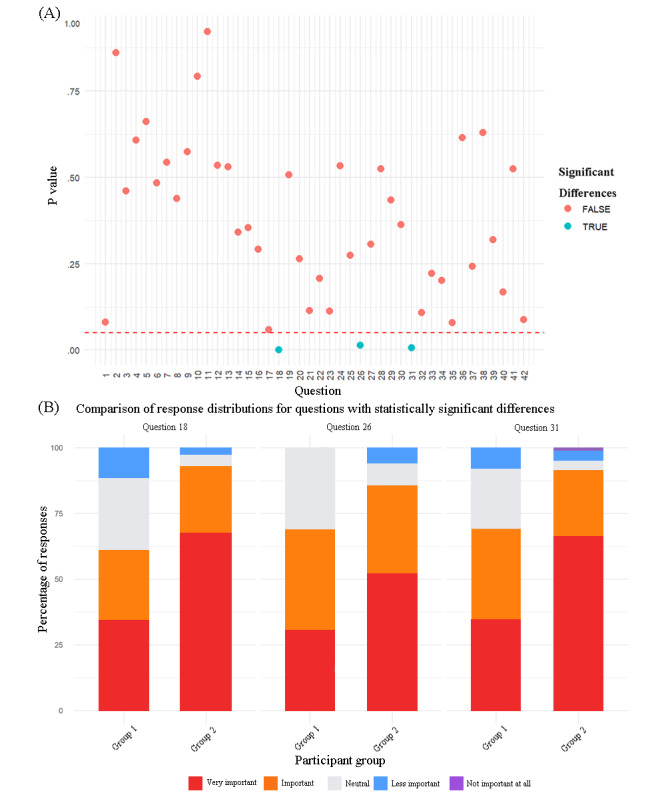
Distribution of response differences between rheumatologists and patients. (A) Scatter points below the red dashed line indicate *P*<.05, suggesting statistically significant differences in answer selection between medical staff and patients for the specific question. (B) Each color block represents the proportion of respondents who selected that option relative to the total. Group 1 was composed of health care professionals, and group 2 was composed of patients.

To explore factors influencing patient prioritization, we compared responses across patient subgroups based on baseline characteristics. The results indicated age was the most significant variable (*P* values ranging from .001 to .05), with 12 questions showing statistically significant age-based differences (question 4, question 13, question 17, question 24, question 27, question 28, question 30, question 31, question 36, question 37, question 38, and question 40; refer to Figures 2A and B. Multimedia Appendix 5 for *P* values). Cross-group analysis of patient versus health care worker priorities revealed statistically significant disparities on 3 questions (question 18, question 26, and question 31; refer to Figures 3A and B. Multimedia Appendix 6 for *P* values).

**Table 1:** Baseline characteristics of the study population (N=84).

Characteristic			Values
**Sex, n (%)**			
	Male	62 (74)	
	Female	22 (26)	
Age (y), mean (SD)			38.01 (10.45)
**Education level, n (%)**			
	Primary school or below	3 (4)	
	Junior high school	10 (12)	
	Senior high school	24 (29)	
	Bachelor’s degree	34 (40)	
	Master’s degree or above	13 (15)	
**Sedentary occupation, n (%)**			
	Yes	47 (56)	
	No	37 (44)	
**Parental health status, n (%)**			
	Good	57 (68)	
	Fair	23 (27)	
	Poor	4 (5)	
**Personal health status, n (%)**			
	Good	33 (39)	
	Fair	42 (50)	
	Poor	9 (11)	
**Family history of axial spondyloarthritis, n (%)**			
	Yes	27 (32)	
	No	57 (68)	
**Family history of hereditary diseases, n (%)**			
	Yes	19 (23)	
	No	65 (77)	
**Daily internet use duration (h), n (%)**			
	<6	57 (68)	
	>6	27 (32)	

### AI Consultation Opinion Quality Assessment

#### Overview

The 42 patient-derived questions were submitted to all 5 selected LLMs, each generating independent responses to avoid memory bias. Outputs were collected and systematically aggregated into bullet point summaries reflecting health consultation content. Three core attributes—readability, accuracy, and incorporation of health disclaimers—were then assessed for each model’s output.

#### Accuracy

**Figure 4:**
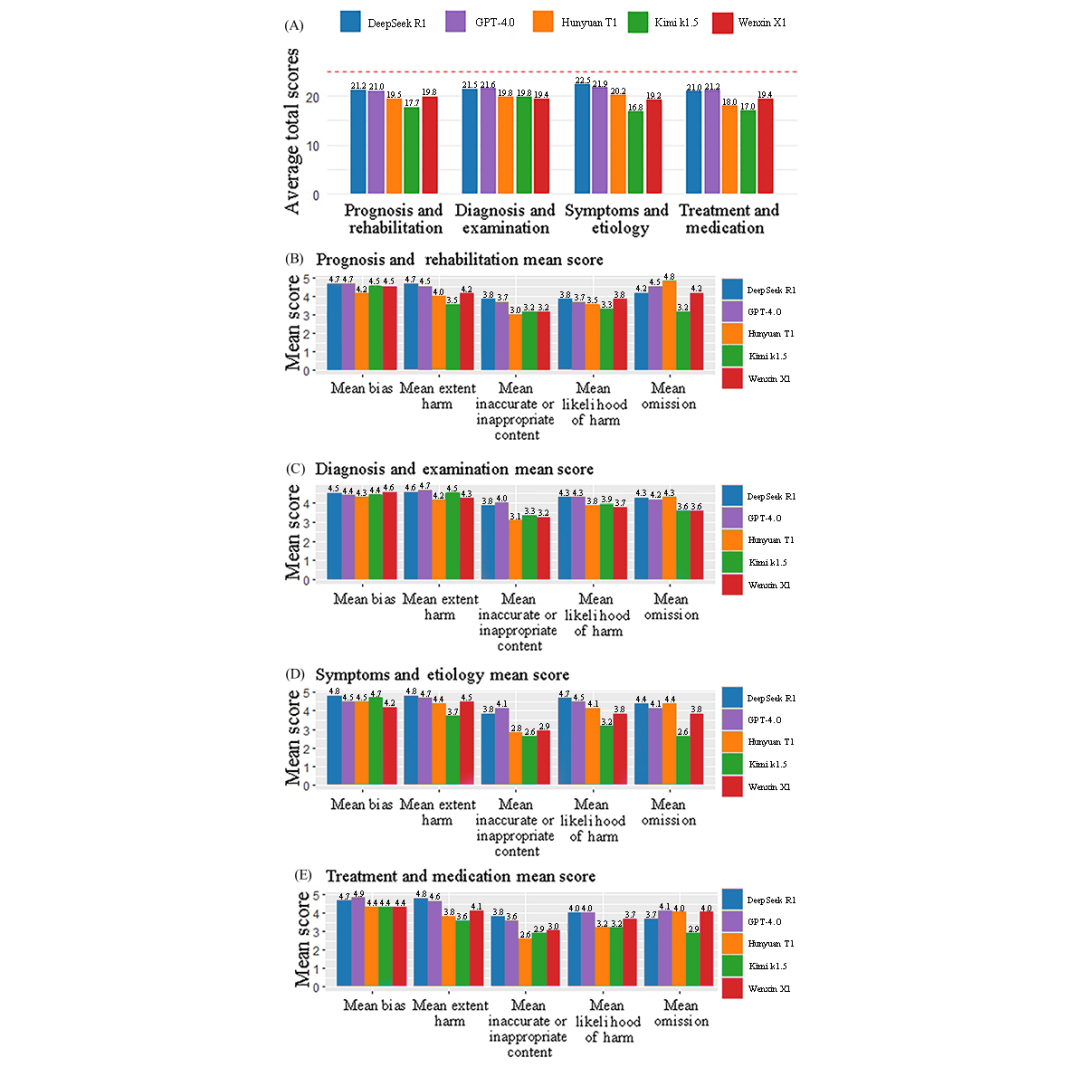
Overall and module-specific score charts. (A) Overall score. (B-E) Scores by module. DS: DeepSeek R1; GPT: GPT-4.0; HY: Hunyuan T1; KM: Kimi k1.5; WX: Wenxin X1.

The 5 LLMs generated 1052 recommendations for the 42 items, including repeated suggestions for the same question across models. Interrater reliability was excellent (Cohen κ=0.947; Figure S2 Multimedia Appendix 4). The diagnosis and examination category yielded the highest average accuracy across models (mean score 20.4, SD 0.9), while the treatment and medication domain scored lowest (mean score 19.3, SD 1.7). Model-specific performance data across domains and question items are provided in Figure 4A; additional breakdowns are detailed in Figures 4B-E; Multimedia Appendix 7 presents complete values. Comparative analysis highlighted that the LLMs’ lowest scores consistently occurred in the “inaccurate or inappropriate content” category, indicating vulnerability to these errors. In contrast, the highest average scores were in the “bias,” suggesting a strong model’s ability to avoid bias in health consultation outputs. Overall, model performance was satisfactory, with total accuracy scores ranging from 16.8 to 22.5. The highest scoring questions spanned all domains (question 3: 23.4 points, question 11: 23.2 points, question 38: 18.2 points, and question 40: 22.4 points), while the lowest scores were concentrated in questions involving nuanced or controversial information (question 6: 17.6 points, question 20: 16.4 points, question 34: 16.6 points, and question 38: 18.2 points).

#### Readability

The readability of LLM-generated health consultation responses was measured using the AlphaReadabilityChinese tool. Comparative analysis of the 5 LLMs’ outputs, as visualized via a heat map in Figure S3 in Multimedia Appendix 4 and detailed in Multimedia Appendix 8, revealed no significant model differences in noun-verb or content-word semantic precision. Kimi k1.5 excelled in lexical richness, verb accuracy, and semantic noise, while GPT-4.0 demonstrated superior syntactic richness, noun accuracy, semantic richness, and semantic clarity. DeepSeek R1, Hunyuan T1, and Wenxin X1 exhibited similar readability performance overall.

#### Disclaimers About Health Advice

Figure S4 in Multimedia Appendix 4 demonstrates that most LLM outputs contained health advice disclaimers, with GPT 4.0 and DeepSeek R1 including such disclaimers in responses to all 42 questions. Kimi k1.5 provided the fewest responses but still included disclaimers in 37 (88%) of the 42 cases.

## Discussion

This study directly addressed real-world concerns of patients with axSpA by fostering collaboration between rheumatologists and patients to develop a comprehensive questionnaire encompassing symptoms, diagnosis, treatment, and prognosis. Subsequent validation with an 84-patient sample demonstrated that the tool reliably reflects patient-identified uncertainties and supports health care professionals in identifying prioritized and neglected issues. This facilitates the creation of targeted educational programs to enhance long-term chronic disease management.

However, marked discrepancies emerged between professionals and patients in the perceived importance of certain topics. For instance, question 18 (“What diseases is this condition likely to be misdiagnosed as?”) was rated more highly by patients than by clinicians [28,29]. Question 31 (“Do biologic agents carry addiction potential?”) and question 26 (“What are the mechanistic differences between NSAIDs, corticosteroids, and analgesics in pain management?”) also showed such divergence [30]. These differences may reflect gaps in professional knowledge transfer, whereby clinicians, familiar with drug mechanisms and risk profiles, may underestimate the informational value these issues hold for patients. This knowledge gap highlights potential inadequacies in current educational practices and underscores the need for efforts to bridge understanding between clinicians and patients in future interventions.

Age is a significant driver of patient perception [31]. Analysis of patients grouped by age (older or younger than 40 years) revealed 12 questions with statistically significant differences, particularly related to symptom management, medication side effects, and prognosis. Younger patients showed increased concern, whereas no significant differences in baseline demographic characteristics were detected (Multimedia Appendix 9). Two main explanations were identified: first, younger patients showed greater interest in novel biological agents and their related mechanisms or risks; second, life stage difference shaped priorities, with patients younger than 40 years demonstrating greater family-planning awareness and early diagnoses mitigating confusion over questions such as question 17. Furthermore, considering axSpA often manifests in early adulthood, older patients, who have lived with the disease for longer, may be more accustomed to standard interventions and less reliant on new information [32]. Collectively, these findings highlight the necessity for age-specific patient education to reflect diverse literacy and life stage requirements, with future health promotion strategies tailored accordingly [33].

A persistent problem observed was AI hallucination, in which LLMs produced confidently stated yet unsourced or inaccurate statistics. For example, in question 41, Hunyuan T1 claimed, “Spinal mobility: 30 minutes of daily yoga can increase the maintenance rate of spinal range of motion by 55% [5-year follow-up data].” While evidence does support mobility benefits of yoga in axSpA through mechanistic pathways, such as muscle strengthening or inflammation reduction, no research corroborates a 55% improvement rate or the alleged 5-year dataset [34]. Although LLMs demonstrated generally strong performance, the safety risk posed by confidently delivered but unfounded claims remains substantial, a threat that cannot be ignored if patients act on these unsubstantiated data. Teaching patients to appraise such claims critically is vital for maximizing LLMs’ potential to support chronic disease management while safeguarding patient health [35].

Despite intermodel variability in accuracy for medical advice [36], the LLMs overall performed robustly in this study. Accuracy ratings in this study were higher compared to previous research, which may be attributable to our open-ended, patient-focused question format and relatively accommodating scoring criteria [37,38]. Ongoing advances in AI technology may also explain this improvement. Notably, the “bias” consistently produced high scores, reflecting a strong capacity to provide wide-ranging yet balanced recommendations. However, the inclination for models to sometimes produce superficially authoritative yet insufficiently substantiated advice, especially regarding clinical management, introduces significant risk. For example, in response to glucocorticoid-related queries (question 35), Wenxin X1 recommended glucocorticoids for pain management without thorough context, potentially exposing patients to avoidable complications, including osteoporosis and serious infections [39,40]. These instances typically resulted in lower “inaccurate or inappropriate content” scores.

Our findings showed that high-scoring LLM responses generally addressed well-established topics with strong supporting evidence. As seen in responses to question 40 (“Can Traditional Chinese Medicine [TCM] treatments replace Western pharmacological therapies?”), all models consistently advised against substituting traditional Chinese medicine (TCM) for Western medicine. GPT-4.0’s response indicated that TCM currently lacks conclusive evidence comparable to that of Western medicine in key efficacy outcomes such as bone protection and symptom control [41,42]. It further clarified that while TCM can serve as an effective adjunctive therapy, Western medicine should remain the foundational treatment approach. Although TCM or acupuncture may serve as useful adjuncts in the management of ankylosing spondylitis, they cannot yet replace the central role of Western medications. We recommend that one works with a specialist to build an integrated, individualized treatment plan that is grounded in Western medicine and supplemented by TCM modalities.

Conversely, lower-scoring questions were primarily those related to medication recommendations. Medication management is highly individualized, requiring customized clinical judgment based on expertise and a comprehensive understanding of the patient’s profile [36,43,44]. Authoritative but uncontextualized LLM guidance may mislead if presented without real-time clinical oversight, posing a substantial safety risk. Patients must be cautioned that any specific medication recommendations from LLMs must always be reviewed and validated by licensed health care professionals before being acted upon.

Readability was an essential metric; both Kimi k1.5 and GPT-4.0 excelled in generating patient-facing content with concise, clear language and minimal jargon, greatly enhancing accessibility and user comprehension [45,46]. These findings underscore a path for further model refinements to improve the communication of medical information to lay audiences.

Most LLMs systematically incorporated health disclaimers, such as “This information cannot replace professional medical advice.” [47,48], which is integral to patient safety. However, inconsistent disclaimer inclusion for less critical questions was observed, calling for the standardization of safety messages across all LLM-generated medical content. Despite generally appropriate use of disclaimers, occasional omissions were noted, representing a residual safety concern, as their absence may increase the risk of patients misinterpreting or misapplying AI-generated advice. To address this, future iterations of medical LLMs should enforce uniform attachment of health advice disclaimers to every health-oriented output, regardless of perceived question severity.

Our study also has some limitations. External generalizability is restricted by the sample size (84 patients and 26 rheumatologists) and single-center, urban tertiary hospital setting, which may limit the applicability of results to broader populations with axSpA with different demographics, health literacy, or health care access. For instance, patients in this top-tier hospital may have distinct expectations, backgrounds, or experiences compared to those in regional or rural centers. In addition, the generalizability of LLM performance and user acceptance may vary by familiarity with digital health tools and local medicolegal contexts. Further multicenter studies spanning diverse socioeconomic and health care environments are necessary to validate these findings and extend the questionnaire’s utility. In addition, reliance on 2 raters for accuracy assessments introduces some subjective bias, although this was minimized via strict guideline adherence and a structured arbitration protocol involving a third researcher. Finally, the exclusive use of Chinese-language responses may not fully extrapolate to other linguistic settings.

This research emphasizes the urgency of patient-centered communication tools in axSpA management and illuminates critical shortcomings in current educational practices. The continual evolution of LLMs offers significant promise and unique challenges for supporting chronic disease care with personalized, accessible, and evidence-grounded information. Addressing AI hallucination through improved model development, integrated fact-checking, and explicit cautionary guidance is imperative to ensure responsible and safe adoption of LLMs in patient health care.

## Appendix

Multimedia Appendix 1 Final version of the questionnaire.

Multimedia Appendix 2 All original responses from 5 large language models.

Multimedia Appendix 3 Scoring standard.

Multimedia Appendix 4 Comparative analysis of the 5 large language models' outputs.

Multimedia Appendix 5 Specific results of chi-square test 1.

Multimedia Appendix 6 Specific results of chi-square test 2.

Multimedia Appendix 7 The scoring results of the various models.

Multimedia Appendix 8 Specific results of chi-square test 3.

Multimedia Appendix 9 Baseline characteristics of different age groups.

## Abbreviations

AI: artificial intelligence
axSpA: axial spondyloarthritis
LLM: large language model
TCM: traditional Chinese medicine

## Data Availability

All data generated or analyzed during this study are included in this published article and its supplementary information files.
