# Supplementary material for: Assessing the Quality of AI Responses to Patient Concerns About Axial Spondyloarthritis: Delphi-Based Evaluation
Source: JMIR AI. 2026 Jan 7;5:e79153. doi: 10.2196/79153 (PMC12824573; doi:10.2196/79153)
Supplement: Multimedia Appendix 1 [file ai_v5i1e79153_app1.doc]

Supplement Table 1. Finalized survey questionnaire

| Question | Answer | A | B | C | D | E |
| --- | --- | --- | --- | --- | --- | --- |
| Q1: What are the potential etiological factors contributing to my lumbosacral pain? |  | Very important | Important | Average | Unimportant | Not Important at All |
| Q2: The exacerbation of nocturnal back pain with improvement upon activity – what underlying pathology might this pattern suggest? |  | Very important | Important | Average | Unimportant | Not Important at All |
| Q3: What could be the pathogenesis of morning stiffness severe enough to limit mobility? |  | Very important | Important | Average | Unimportant | Not Important at All |
| Q4: Is there a clinical association between my lumbar pain, ocular pruritus, and diarrheal symptoms? |  | Very important | Important | Average | Unimportant | Not Important at All |
| Q5: Could my back pain indicate a serious systemic disorder? |  | Very important | Important | Average | Unimportant | Not Important at All |
| Q6: Under what clinical circumstances should I seek urgent medical evaluation for this low back pain? |  | Very important | Important | Average | Unimportant | Not Important at All |
| Q7: Given my family history of ankylosing spondylitis (AS), what is the likelihood of my current back pain being AS-related? |  | Very important | Important | Average | Unimportant | Not Important at All |
| Q8: What are the pathognomonic features distinguishing inflammatory back pain in AS from mechanical low back pain? |  | Very important | Important | Average | Unimportant | Not Important at All |
| Q9: Why would chest wall pain raise clinical suspicion for ankylosing spondylitis? |  | Very important | Important | Average | Unimportant | Not Important at All |
| Q10: Is there evidence supporting psychosomatic contributions to chronic low back pain? |  | Very important | Important | Average | Unimportant | Not Important at All |
| Q11: What is the diagnostic significance of HLA-B27 seropositivity in spondyloarthropathy evaluation? |  | Very important | Important | Average | Unimportant | Not Important at All |
| Q12: How should elevated acute-phase reactants (CRP and ESR) be interpreted in the context of suspected AS? |  | Very important | Important | Average | Unimportant | Not Important at All |
| Q13: Why might radiographic findings remain unremarkable despite severe clinical symptoms? |  | Very important | Important | Average | Unimportant | Not Important at All |
| Q14: What is the prognostic implication of bone marrow edema in sacroiliac joints on MRI for AS diagnosis? |  | Very important | Important | Average | Unimportant | Not Important at All |
| Q15: What are the validated classification criteria for definitive AS diagnosis? |  | Very important | Important | Average | Unimportant | Not Important at All |
| Q16: What is the clinical rationale for comprehensive imaging (X-ray/CT/MRI) and serological testing in AS workup? |  | Very important | Important | Average | Unimportant | Not Important at All |
| Q17: Does advanced age preclude new-onset ankylosing spondylitis diagnosis? |  | Very important | Important | Average | Unimportant | Not Important at All |
| Q18: What are the most common diagnostic mimics of AS in clinical practice? |  | Very important | Important | Average | Unimportant | Not Important at All |
| Q19: How should persistent diagnostic uncertainty be addressed when clinical suspicion contradicts specialist opinions? |  | Very important | Important | Average | Unimportant | Not Important at All |
| Q20: What analgesic protocols are recommended for undifferentiated axial pain? |  | Very important | Important | Average | Unimportant | Not Important at All |
| Q21: What is the estimated effective radiation dose of standard sacroiliac CT and its associated health risks? |  | Very important | Important | Average | Unimportant | Not Important at All |
| Q22: Why might AS diagnosis remain uncertain despite HLA-B27 positivity, inflammatory markers, and suggestive symptoms? |  | Very important | Important | Average | Unimportant | Not Important at All |
| Q23: What are the long-term functional prognosis and disability risks in untreated AS? |  | Very important | Important | Average | Unimportant | Not Important at All |
| Q24: What is the anticipated duration of disease-modifying therapy in AS management? |  | Very important | Important | Average | Unimportant | Not Important at All |
| Q25: Are there contraindications to vaccination during immunomodulatory treatment? |  | Very important | Important | Average | Unimportant | Not Important at All |
| Q26: What are the mechanistic differences between NSAIDs, corticosteroids, and analgesics in pain management? |  | Very important | Important | Average | Unimportant | Not Important at All |
| Q27: What are the safety considerations for analgesic dose escalation in refractory pain? |  | Very important | Important | Average | Unimportant | Not Important at All |
| Q28: Could drug-induced chromaturia indicates hepatorenal toxicity? |  | Very important | Important | Average | Unimportant | Not Important at All |
| Q29: What defines biologic DMARDs in the context of targeted AS therapy? |  | Very important | Important | Average | Unimportant | Not Important at All |
| Q30: What is the risk profile of TNF-α inhibitors regarding opportunistic infections? |  | Very important | Important | Average | Unimportant | Not Important at All |
| Q31: Do biologic agents carry addiction potential? |  | Very important | Important | Average | Unimportant | Not Important at All |
| Q32: What alternative therapies exist for AS patients with contraindications to biologics (e.g., HBV/TB coinfection)? |  | Very important | Important | Average | Unimportant | Not Important at All |
| Q33: Is concomitant conventional DMARD use necessary with biologic therapy? |  | Very important | Important | Average | Unimportant | Not Important at All |
| Q34: What are the withdrawal protocols for biologic agents in sustained remission? |  | Very important | Important | Average | Unimportant | Not Important at All |
| Q35: What is the therapeutic role of glucocorticoids in AS management? |  | Very important | Important | Average | Unimportant | Not Important at All |
| Q36: How should perioperative medication (including biologics) be managed in AS patients undergoing surgery? |  | Very important | Important | Average | Unimportant | Not Important at All |
| Q37: Are there pharmacokinetic interactions between AS medications and common OTC drugs (e.g., cold remedies)? |  | Very important | Important | Average | Unimportant | Not Important at All |
| Q38: What are the fertility preservation strategies and medication adjustments for AS patients planning conception? |  | Very important | Important | Average | Unimportant | Not Important at All |
| Q39: How should disease-modifying agents be managed during pregnancy preparation? |  | Very important | Important | Average | Unimportant | Not Important at All |
| Q40: What is the evidence hierarchy comparing traditional Chinese medicine (TCM) with Western pharmacotherapy in AS control? |  | Very important | Important | Average | Unimportant | Not Important at All |
| Q41: What are the exercise recommendations for AS patients experiencing active axial pain? |  | Very important | Important | Average | Unimportant | Not Important at All |
| Q42: What are the surgical indications for end-stage AS complications? |  | Very important | Important | Average | Unimportant | Not Important at All |
