# Supplementary material for: Assessing the Quality of AI Responses to Patient Concerns About Axial Spondyloarthritis: Delphi-Based Evaluation
Source: JMIR AI. 2026 Jan 7;5:e79153. doi: 10.2196/79153 (PMC12824573; doi:10.2196/79153)
Supplement: Multimedia Appendix 2 [file ai_v5i1e79153_app2.doc]

Appendix 1. All original responses of Large Language Models (LLMs)

DeepSeek

1.强直性脊柱炎（AS）相关的腰背疼痛通常具有**炎症性背痛**的特征，但你的症状也可能是其他原因引起的。以下是需要你重点关注的信息：

---

### **一、强直性脊柱炎（AS）的典型疼痛特点**

1. **晨僵明显**：早晨起床时腰背僵硬感＞30分钟，活动后减轻。

2. **夜间痛醒**：尤其在后半夜或凌晨因疼痛醒来，翻身困难。

3. **“活动后缓解”**：疼痛在休息时加重，适度活动（如散步）后减轻。

4. **慢性病程**：症状持续3个月以上，可能伴随臀部交替性疼痛。

⚠️ 若符合以上特征，需尽早就诊风湿免疫科，通过**HLA-B27检测、骶髂关节MRI/X线**进一步确认。

---

### **二、其他可能导致腰背痛的常见原因**

1. **机械性损伤**

- 肌肉劳损、腰椎间盘突出、骨质增生

- 特点：活动后疼痛加重，休息缓解，无晨僵。

2. **骨质疏松**

- 中老年人群高发，疼痛可能伴随身高变矮、驼背。

3. **感染或肿瘤**

- 如脊柱结核、骨髓炎或转移瘤，可能伴随发热、体重下降。

4. **内脏牵涉痛**

- 肾结石（单侧绞痛+血尿）、胰腺炎（上腹放射性痛）、盆腔炎（女性下腹坠痛）等。

---

### **三、建议下一步行动**

1. **观察记录症状**：

- 疼痛时间（是否夜间/晨起加重）、伴随症状（发热、皮疹、腹泻等）、活动后是否缓解。

- 家族史（亲属是否有AS、银屑病或炎症性肠病）。

2. **尽快就医检查**：

- **优先排查AS**：风湿免疫科（查HLA-B27、CRP、ESR、骶髂关节影像）。

- **其他科室**：骨科（排除椎间盘问题）、泌尿科/妇科（排查内脏疾病）。

3. **日常缓解措施**：

- **适度活动**：游泳、瑜伽等伸展运动，避免久坐。

- **热敷/冷敷**：急性疼痛期冷敷，慢性僵硬期热敷。

- **避免自行用药**：非甾体抗炎药（如布洛芬）可短期缓解，但长期使用需医生指导。

---

### **四、重要提醒**

即使怀疑是AS，早期规范治疗也可有效控制病情进展，请勿过度焦虑。及时就医明确诊断是关键！

如果需要进一步分析，请补充以下信息：

① 疼痛具体位置（下腰/胸背/颈部）？

② 是否有足跟痛、眼炎（葡萄膜炎）或肠道不适？

③ 近期是否做过相关检查？

2.根据你描述的症状（**夜间背痛明显，活动后缓解**），确实需要优先考虑**炎症性背痛**的可能病因，但其他疾病也不能完全排除。以下是具体分析和建议：

---

### **一、最可能的疾病方向**

#### **1. 脊柱关节炎（包括强直性脊柱炎）**

- **高度警示信号**：

✅ 夜间痛醒（尤其是凌晨3-5点）

✅ 活动后疼痛减轻（与机械性腰痛相反）

✅ 晨僵＞30分钟

- **下一步建议**：

尽快就诊风湿免疫科，检查 **HLA-B27基因、骶髂关节MRI（敏感度高于X线）、CRP/ESR（炎症指标）**。

#### **2. 其他类型的脊柱关节炎**

- **反应性关节炎**：可能伴随尿道炎、结膜炎或肠道感染史。

- **银屑病关节炎**：若有银屑病（牛皮癣）皮疹或指甲凹陷，需警惕。

- **肠病性关节炎**：伴随克罗恩病/溃疡性结肠炎的腹泻、腹痛等。

#### **3. 纤维肌痛综合征**

- **特点**：广泛性肌肉疼痛、疲劳、睡眠障碍，但无关节肿胀或炎症指标升高。

- **排查点**：按压18个特定部位（如颈部、肩部、髋部）是否有多处压痛。

---

### **二、需要警惕的“危险信号”**

如果出现以下情况，需立即就医（可能提示严重疾病）：

⚠️ 发热、体重减轻、盗汗（感染/肿瘤风险）

⚠️ 下肢麻木无力、大小便失禁（脊髓压迫）

⚠️ 胸痛、呼吸困难（心肺疾病牵涉痛）

---

### **三、建议立即采取的行动**

#### **1. 记录症状细节（就诊时直接提供给医生）**

- 疼痛具体部位（下背部、胸椎、肋骨？是否向臀部放射？）

- 是否伴随其他症状：足跟痛、眼红/视力模糊（葡萄膜炎）、皮疹、腹泻等。

- 疼痛持续总时长（是否＞3个月？）

#### **2. 就医检查建议**

- **必查项目**：

- **骶髂关节MRI**（早期AS的最佳检测手段）

- **HLA-B27**（约90% AS患者阳性，但阳性≠确诊）

- **炎症指标**：CRP、ESR

- **可选排查**：

- 骨密度检测（排除骨质疏松）

- 泌尿系/妇科超声（排除肾结石、盆腔炎）

#### **3. 暂时缓解疼痛的方法**

- **夜间睡眠**：选择硬板床，避免软床加重脊柱弯曲；侧卧时在两腿间夹枕头。

- **热敷**：睡前用40℃热毛巾敷疼痛部位15分钟，缓解肌肉痉挛。

- **简单拉伸**：醒后做“猫牛式”脊柱伸展（跪姿，交替拱背和塌腰）。

- **避免自行用药**：非甾体抗炎药（如塞来昔布）可短期缓解疼痛，但需医生评估后使用。

---

### **四、关于“强直性脊柱炎”的特别说明**

即使最终确诊AS，也不必过度恐慌：

1. **早诊早治可控制**：生物制剂（如TNF-α抑制剂）可显著延缓骨融合。

2. **功能锻炼是关键**：坚持游泳、太极等运动，能维持关节活动度。

3. **避免误区**：

✖️ 切勿盲目正骨、推拿（可能加重炎症）。

✖️ 不要依赖止痛药掩盖症状。

---

### **五、下一步总结**

1. 若条件允许，**72小时内预约风湿免疫科**（优先选三甲医院）。

2. 如果夜间疼痛难以忍受，可急诊排查肾结石、主动脉夹层等急症。

3. 补充回答以下信息，我会进一步帮你分析：

🔹 疼痛是否从臀部开始逐渐向上蔓延？

🔹 是否有家族成员患银屑病、虹膜炎或慢性腹泻？

🔹 近期是否感染过（如腹泻、尿道炎）？

3.你的症状（**晨起身体僵硬无法活动**）高度提示存在**炎症性关节病变**，尤其是与**脊柱关节炎**相关的疾病（如强直性脊柱炎）。以下是详细分析和针对性建议：

---

### **一、首要怀疑方向：炎症性关节病**

#### **1. 强直性脊柱炎（AS）**

- **核心特征**：

✅ 晨僵＞30分钟，活动后缓解

✅ 夜间腰痛（凌晨加重，活动后减轻）

✅ 可能伴随足跟痛、臀部交替痛、眼炎（葡萄膜炎）

- **关键检查**：

🔍 **骶髂关节MRI**（早期发现骨髓水肿）

🔍 **HLA-B27基因检测**（约90% AS患者阳性）

🔍 **炎症指标**（CRP、ESR升高提示活动性炎症）

#### **2. 类风湿关节炎（RA）**

- **区别点**：

✅ 晨僵同样明显，但主要影响 **小关节**（手指、手腕）

✅ 关节对称性肿胀，可能伴随类风湿结节

✅ 血液检查：抗CCP抗体、类风湿因子（RF）阳性

- **注意**：RA极少单独导致脊柱僵硬，需排查是否合并其他疾病。

#### **3. 银屑病关节炎（PsA）**

- **警示信号**：

✅ 银屑病皮疹或指甲病变（甲凹陷、甲剥离）

✅ 可能累及脊柱或单侧大关节（如膝关节）

#### **4. 其他脊柱关节炎**

- 反应性关节炎（近期有肠道/泌尿道感染史）

- 肠病性关节炎（伴随克罗恩病/溃疡性结肠炎）

---

### **二、其他可能原因（需排查）**

1. **骨关节炎（退行性病变）**

- 中老年多见，晨僵通常＜30分钟，活动后短暂缓解。

- X线可见骨赘形成、关节间隙狭窄。

2. **纤维肌痛综合征**

- 广泛性肌肉僵硬+压痛，但无关节肿胀或炎症指标异常。

- 常合并疲劳、失眠、焦虑。

3. **感染或肿瘤**（少见但危险）

- 如脊柱结核、骨髓炎或淋巴瘤，可能伴随发热、体重下降。

---

### **三、急需就医的“危险信号”**

若出现以下情况，需 **24小时内急诊**：

⚠️ 突发肢体无力或麻木（脊髓压迫风险）

⚠️ 高热（体温＞39℃）+ 背部剧痛（感染或肿瘤）

⚠️ 视力急剧下降、眼红眼痛（葡萄膜炎急诊）

---

### **四、下一步行动建议**

#### **1. 优先就诊科室**

- **风湿免疫科**（重点排查AS、类风湿关节炎）

- **骨科**（排除椎间盘突出、骨关节炎）

#### **2. 关键检查清单**

| **检查项目** | **目的** |

|----------------------|--------------------------------------|

| 骶髂关节MRI | 早期发现AS特征性炎症（敏感性最高） |

| HLA-B27基因检测 | 辅助诊断AS（非确诊依据） |

| CRP、ESR | 评估全身炎症活跃度 |

| 抗CCP抗体、RF | 排除类风湿关节炎 |

| X线/CT（腰椎、骨盆） | 观察骨质破坏、钙化 |

#### **3. 今日可做的自我缓解**

- **晨僵管理**：

🔹 醒后先做 **床上伸展**：仰卧屈膝，双手抱膝缓慢拉向胸部，保持10秒。

🔹 **热敷僵硬部位**：用热毛巾或暖宝宝（40℃左右）敷15分钟，促进血液循环。

- **生活习惯调整**：

🔹 睡硬板床，避免软床加重脊柱弯曲。

🔹 侧睡时双腿间夹枕头，保持脊柱中立位。

- **避免错误行为**：

✖️ 不要暴力拉伸或盲目推拿（可能加重炎症）。

✖️ 勿长期自行服用止痛药（掩盖病情，延误诊断）。

---

### **五、关于“强直性脊柱炎”的补充说明**

1. **早期治疗可阻断进展**：

- 若确诊AS，生物制剂（如依那西普、阿达木单抗）可显著抑制炎症，预防骨融合。

2. **功能锻炼比药物更重要**：

- 每日坚持 **游泳、八段锦、瑜伽**（脊柱旋转、扩胸运动）。

3. **预后**：

- 规范治疗下，大多数患者可维持正常工作和生活，但需避免负重和久坐。

---

### **六、请补充以下信息（方便进一步分析）**

1. 晨僵主要部位：下背部/颈部/全身关节？

2. 是否出现过眼睛发红、视力模糊？

3. 家族中是否有类似疾病（如AS、银屑病、炎症性肠病）？

及时就医是明确诊断的关键！你的症状有高度治疗价值，请勿拖延。

4.你的腰背疼痛合并 **眼睛瘙痒** 和 **腹泻**，确实可能存在关联！这种多系统症状的组合提示 **全身性炎症性疾病** 或 **免疫反应异常**，需重点关注以下可能性：

---

### **一、需优先考虑的关联性疾病**

#### **1. 脊柱关节炎相关疾病**

- **肠病性关节炎**

✅ **关联机制**：克罗恩病/溃疡性结肠炎（IBD）与脊柱关节炎共病率高达20%，肠道炎症释放的炎性因子可攻击关节和眼睛。

✅ **典型表现**：

🔹 **腹泻**：黏液脓血便、腹痛（IBD特征）

🔹 **眼部症状**：葡萄膜炎（眼红、畏光、视力模糊）或结膜炎（眼痒、分泌物）

🔹 **腰背痛**：炎症性背痛（夜间加重，活动缓解）

- **反应性关节炎**

✅ **触发因素**：肠道/泌尿系感染（如沙门氏菌、衣原体）后2-4周发病。

✅ **经典三联征**：

🔹 关节炎（腰背/膝关节肿痛）

🔹 尿道炎（排尿痛）

🔹 结膜炎（眼红、眼痒）

✅ **其他表现**：足底筋膜炎、皮肤溢脓性角化症。

#### **2. 强直性脊柱炎（AS）合并并发症**

- **AS本身可引起关节外损害**：

🔹 **眼部**：急性前葡萄膜炎（需警惕！表现为眼红、疼痛、视力下降，而非单纯眼痒）。

🔹 **肠道**：约10% AS患者合并亚临床肠道炎症（轻度腹泻/腹痛）。

#### **3. 白塞病（Behçet’s disease）**

- **典型三联征**：

✅ 口腔/生殖器溃疡

✅ 葡萄膜炎（眼红、眼痛）

✅ 皮肤结节性红斑

- **可能伴随**：关节炎、肠道溃疡（腹泻、便血）。

#### **4. 过敏或自身免疫交叉反应**

- **食物过敏/不耐受**（如乳糖不耐受）：

🔹 腹泻 + 肠黏膜炎症 → 触发全身免疫反应 → 关节痛、结膜充血（眼痒）。

- **环境过敏**（花粉/尘螨）：

🔹 过敏性结膜炎（眼痒为主）与腰痛可能为巧合，需排查其他关联。

---

### **二、关键鉴别点与检查建议**

#### **1. 症状细节对比**

| **疾病** | **腰背痛特点** | **腹泻特征** | **眼部表现** |

|--------------------|----------------------|---------------------------|---------------------------|

| 肠病性关节炎 | 炎性背痛 | 黏液脓血便、反复发作 | 葡萄膜炎（红痛＞痒） |

| 反应性关节炎 | 下肢大关节肿痛 | 感染后水样泻 | 结膜炎（眼痒、分泌物） |

| AS合并葡萄膜炎 | 典型AS炎性背痛 | 无或轻度腹泻 | 急性前葡萄膜炎（急症！） |

| 白塞病 | 游走性关节炎 | 肠道溃疡性腹泻（可便血） | 葡萄膜炎+口腔/生殖器溃疡 |

#### **2. 急需完成的检查**

- **必查项目**：

1. **风湿免疫相关**：HLA-B27、CRP、ESR、抗核抗体（ANA）。

2. **肠道评估**：粪便钙卫蛋白（鉴别IBD）、便常规+潜血、必要时肠镜。

3. **眼部检查**：裂隙灯检查（确诊葡萄膜炎）、过敏原检测。

- **影像学**：骶髂关节MRI（评估AS）、关节超声（排查滑膜炎）。

---

### **三、下一步行动指南**

1. **立即就诊科室**：

- **风湿免疫科**（核心科室，排查AS、反应性关节炎、白塞病）

- **消化内科**（评估腹泻原因，区分IBD或感染）

- **眼科**（明确眼痒是否为葡萄膜炎或过敏）

2. **症状记录要点**：

- 腹泻频率、是否带血/黏液？

- 眼痒是否伴随眼红、畏光、视力下降？

- 是否有口腔/生殖器溃疡、皮疹？

3. **临时缓解措施**：

- **眼部**：冷敷缓解瘙痒，避免揉眼（防感染）。

- **肠道**：清淡饮食（暂避乳制品、高纤维食物），口服补液盐防脱水。

- **腰部**：继续热敷+适度拉伸，避免久坐。

---

### **四、重要提醒**

1. **葡萄膜炎是急诊**！若眼痒发展为眼红、疼痛、视物模糊，需 **24小时内就诊眼科**，延误可能致永久视力损伤。

2. **便血+腹痛**提示IBD或感染，需尽快肠镜检查。

3. 勿自行使用抗生素或激素眼药水，可能掩盖病情。

请尽快完善检查，多学科协作才能准确揪出病因！

5.理解你的担忧！**腰疼可能是多种疾病的表现，其中绝大多数并不严重，但确实存在少数需要紧急处理的情况**。以下是基于最新医学指南的客观分析，帮助你初步判断风险：

---

### **一、常见且通常不严重的腰痛原因**

1. **肌肉或韧带劳损**

- **诱因**：久坐、搬运重物、错误姿势运动。

- **特点**：局部压痛，活动受限，休息1-2周可缓解。

2. **腰椎间盘突出**

- **典型表现**：腰痛向单侧下肢放射（坐骨神经痛），咳嗽/打喷嚏时加重。

- **风险**：若未压迫神经，多数可通过保守治疗恢复。

3. **退行性骨关节炎**

- **人群**：中老年为主，X线可见骨赘形成。

- **特点**：晨僵＜30分钟，活动后短暂缓解。

---

### **二、可能提示严重疾病的“危险信号”**

若腰痛伴随以下任一情况，需 **立即就医**：

⚠️ **全身症状**：

- 不明原因发热（＞38℃）

- 体重骤降（3个月内下降＞10%）

- 夜间盗汗（床单浸湿）

⚠️ **神经系统异常**：

- 下肢麻木/无力

- 大小便失禁或排尿困难

⚠️ **疼痛特征异常**：

- 静息痛（不动时更痛，尤其是夜间）

- 进行性加重，止痛药无法缓解

- 胸背痛合并呼吸困难（警惕主动脉夹层）

⚠️ **癌症风险因素**：

- 癌症病史（尤其是乳腺癌、肺癌、前列腺癌）

- 年龄＞50岁+新发腰痛

---

### **三、需要排查的严重疾病**

| **疾病类型** | **关键线索** | **确诊检查** |

|--------------------|---------------------------------------|-------------------------------|

| **脊柱感染** | 发热+局部皮肤红肿热痛 | MRI+血培养 |

| **脊髓压迫** | 下肢无力+大小便障碍 | 急诊MRI |

| **恶性肿瘤转移** | 夜间剧痛+体重下降+癌症史 | PET-CT/骨扫描 |

| **腹主动脉瘤** | 突发撕裂样痛+血压异常 | 腹部超声/CTA |

| **肾盂肾炎** | 腰痛+发热+尿频尿痛 | 尿常规+肾脏超声 |

---

### **四、建议你立即采取的行动**

1. **初步自测**：

- 尝试做“直腿抬高试验”：仰卧抬腿，若＜60°时出现下肢放射性痛，提示神经根受压。

- 按压腰部肌肉：若局部压痛明显且无放射痛，多为肌肉问题。

2. **就医优先级判断**：

- **24小时内急诊**：出现任何上述“危险信号”。

- **1周内就诊**：疼痛持续＞2周不缓解，或影响日常活动。

- **普通门诊**：轻微疼痛无危险信号，可先观察1-2周。

3. **推荐检查**：

- **基础检查**：腰椎X线、血常规、CRP（筛查感染/炎症）。

- **深度检查**（若基础检查异常）：腰椎MRI、肿瘤标志物、骨扫描。

---

### **五、你可以尝试的自我管理**

- **急性期（＜48小时）**：

🔹 冰敷疼痛部位（每次15分钟，每日3次），减轻炎症。

🔹 短期（3-5天）使用非甾体抗炎药（如布洛芬）。

- **慢性期（＞3个月）**：

🔹 强化核心肌群：每天做“平板支撑”（从30秒开始）。

🔹 避免久坐：每1小时起身活动5分钟。

- **绝对禁忌**：

✖️ 疼痛期间避免推拿正骨（可能加重神经损伤）。

✖️ 勿长期服用止痛药掩盖症状。

---

### **六、总结：你的腰痛严重吗？**

- **低风险**：仅表现为肌肉酸痛，无危险信号，活动后缓解→ 观察1-2周。

- **中高风险**：符合任何一条“危险信号”→ **立即就医**。

**即使是严重疾病，早期干预也能显著改善预后**，请根据自身情况及时行动！

6.**腰痛的严重性评估及就医建议**

---

### **一、腰痛可能的严重性分析**

腰痛多数由常见问题引起，但少数情况可能提示严重疾病。以下为快速判断要点：

#### **1. 需立即就医的“危险信号”**

出现以下任一症状，**24小时内就诊**（急诊优先）：

⚠️ **下肢无力/麻木**或大小便失禁（提示脊髓压迫）

⚠️ **持续发热+夜间盗汗**（感染/肿瘤风险）

⚠️ **突发剧烈腰痛**，尤其是伴随腹痛或胸痛（警惕主动脉夹层、肾结石嵌顿）

⚠️ **体重骤降**（3个月内下降＞10%）

⚠️ **癌症病史或长期激素治疗**（警惕转移性骨病）

#### **2. 可能提示中度风险的情况**

建议**1周内就诊**：

🔸 疼痛持续＞2周且无缓解迹象

🔸 疼痛向腿部放射（可能神经受压）

🔸 晨僵＞30分钟，活动后缓解（炎症性背痛）

🔸 近期外伤史（如跌倒后疼痛加重）

#### **3. 低风险情况**

可暂时观察（1-2周）：

🔹 疼痛局限在腰部，活动后减轻

🔹 无危险信号，可能与久坐、姿势不良相关

---

### **二、是否需要看医生？——快速决策指南**

| **你的情况** | **行动建议** |

|----------------------------------|----------------------------------|

| 有上述任何“危险信号” | **立即挂急诊**（优先骨科/神经科）|

| 疼痛影响睡眠或日常活动 | **3天内预约骨科/风湿科** |

| 疼痛轻微但持续＞2周 | **1周内普通门诊排查** |

| 偶发疼痛，休息后缓解且无其他症状 | 观察+自我护理（见下文） |

---

### **三、自我护理与观察期管理**

#### **1. 急性期（疼痛＜48小时）**

- **冰敷**：用毛巾包裹冰袋敷疼痛处，每次15分钟，每日3-4次。

- **药物**：短期（≤3天）用布洛芬或对乙酰氨基酚止痛。

- **休息**：避免弯腰提重物，但不要绝对卧床（可短时间散步）。

#### **2. 慢性疼痛（＞3个月）**

- **热敷**：用40℃热毛巾敷腰部，每日2次，每次20分钟。

- **核心训练**：每天做“臀桥”（仰卧抬臀）3组，每组10次。

- **姿势调整**：久坐时使用腰靠，电脑屏幕与眼睛平齐。

#### **3. 必须避免的行为**

✖️ 盲目正骨/推拿（可能加重神经损伤）

✖️ 长期依赖止痛药掩盖症状

✖️ 疼痛期间高强度运动（如举铁、仰卧起坐）

---

### **四、就医前准备**

1. **记录症状**：

- 疼痛部位（画图标记）、发作时间（晨起/夜间？）、加重/缓解因素。

- 是否伴随腿麻、发热、排尿异常等。

2. **准备问题**：

- “我的腰痛最可能是什么原因？”

- “需要做哪些检查？（如X光、MRI）”

- “如果吃药无效，下一步治疗方案是什么？”

---

### **五、总结：你的下一步**

- **有危险信号** → **立即急诊**

- **无危险信号但影响生活** → **3天内就诊**

- **轻微疼痛** → 观察1-2周，无效则就医

**早期干预可避免多数严重并发症**，切勿因犹豫延误诊断！

7.---

### **一、腰痛是否严重？关键风险信号判断**

腰痛多数由非严重问题引起，但需警惕以下 **“危险信号”**，若出现需 **立即就医**：

⚠️ **下肢无力/麻木**或大小便失禁（脊髓压迫）

⚠️ **持续发热+体重骤降**（感染/肿瘤可能）

⚠️ **静息痛/夜间痛醒**（尤其是凌晨3-5点加重）

⚠️ **胸背撕裂样剧痛**（主动脉夹层风险）

**若无非危险信号**，常见原因如肌肉劳损、腰椎间盘突出等，通常不严重。

---

### **二、家族史+背痛：强直性脊柱炎（AS）风险分析**

#### **1. 家族史显著增加AS风险**

- **遗传关联**：AS与 **HLA-B27基因**强相关（约90%患者阳性），一级亲属患病风险提高5-10倍。

- **你的风险**：有AS家族史+背痛，需优先排查AS，但非绝对确诊依据。

#### **2. AS的典型症状对比**

| **你的症状** | **是否符合AS？** |

|----------------------|----------------------------------|

| 背痛夜间加重、活动缓解 | ✅ 高度符合（炎症性背痛特征） |

| 晨僵＞30分钟 | ✅ 典型AS表现 |

| 家族中有AS患者 | ✅ 重要风险因素 |

| 疼痛局限在腰部 | ⚠️ AS常从骶髂关节向上蔓延 |

#### **3. 确诊AS的核心条件**

- **影像学证据**：骶髂关节炎（X线/MRI显示关节侵蚀或骨髓水肿）

- **临床特征**：炎症性背痛+HLA-B27阳性（或其他脊柱关节病表现）。

---

### **三、建议立即采取的行动**

#### **1. 优先就诊科室**

- **风湿免疫科**：针对性排查AS（约50% AS患者初诊被误诊为腰肌劳损）。

- **骨科**：排除腰椎间盘突出、骨质增生等机械性病因。

#### **2. 关键检查清单**

| **检查项目** | **意义** |

|----------------------|----------------------------------------|

| **骶髂关节MRI** | 早期发现AS特征性炎症（优于X线） |

| **HLA-B27基因检测** | 辅助诊断（阳性需结合症状，阴性不完全排除） |

| **CRP/ESR** | 评估全身炎症活跃度 |

| **腰椎X线/CT** | 排除骨质增生、椎间盘病变 |

#### **3. 日常管理（就诊前）**

- **记录症状**：晨僵时长、夜间痛醒频率、活动后是否缓解。

- **热敷+拉伸**：每日热敷下背部20分钟，做“猫牛式”脊柱伸展。

- **避免久坐**：每1小时起身活动，选择硬板床睡觉。

---

### **四、强直性脊柱炎（AS）的明确与排除**

#### **1. 若确诊AS**

- **早治疗可控制**：非甾体抗炎药（如塞来昔布）联合生物制剂（如TNF-α抑制剂）可显著延缓病情。

- **功能锻炼最关键**：游泳、瑜伽、太极等维持脊柱灵活性。

#### **2. 若排除AS**

- 可能为其他脊柱关节炎（如银屑病关节炎）或机械性腰痛，针对性治疗预后良好。

---

### **五、总结：你的下一步决策**

1. **无危险信号** → 1周内挂 **风湿免疫科**，完成骶髂关节MRI+HLA-B27检测。

2. **有夜间痛醒/晨僵** → 72小时内就诊，优先排查AS。

3. **出现下肢麻木/发热** → 立即急诊。

**即使有家族史，AS也非必然**！及时检查才能明确诊断，避免过度焦虑。

8.以下是强直性脊柱炎（AS）相关的背痛与其他常见背痛的区别，按核心特征分类说明：

---

### **一、疼痛性质对比**

| **特征** | **强直性脊柱炎（AS）** | **其他常见背痛（如肌肉劳损、腰椎间盘突出）** |

|------------------------|--------------------------------------|--------------------------------------------|

| **发作模式** | 慢性渐进性（持续≥3个月） | 急性或间歇性（数天至数周） |

| **昼夜节律** | ✅ 夜间痛醒（尤其是凌晨） | ❌ 白天活动后加重，夜间休息缓解 |

| **活动影响** | 活动后减轻，休息不缓解甚至加重 | 活动后加重，休息缓解 |

| **晨僵时间** | ＞30分钟，缓慢缓解 | ＜30分钟，快速缓解 |

| **疼痛部位** | 从骶髂关节（臀部深处）向上蔓延至脊柱 | 集中在腰部局部，可能放射至下肢（坐骨神经痛）|

---

### **二、伴随症状对比**

| **症状类型** | **AS典型表现** | **其他背痛常见表现** |

|------------------------|--------------------------------------|--------------------------------------------|

| **关节外表现** | 葡萄膜炎（眼红/痛/视力模糊）、足跟痛、肠道炎症（腹泻） | 无或仅局部肌肉紧张/压痛 |

| **全身症状** | 低热、疲劳（炎症活动期） | 通常无 |

| **姿势变化** | 晚期可能脊柱强直、驼背 | 保护性姿势（如侧弯） |

---

### **三、病因与危险因素**

| **类别** | **AS** | **其他背痛** |

|------------------------|--------------------------------------|--------------------------------------------|

| **核心病因** | 自身免疫性炎症（攻击韧带附着点） | 机械损伤、退行性变、神经压迫 |

| **遗传风险** | ✅ HLA-B27阳性（90%患者） | 无关 |

| **年龄** | 青壮年起病（20-40岁） | 各年龄段（退行性病变多见于中老年） |

---

### **四、诊断方法差异**

| **检查手段** | **AS确诊关键** | **其他背痛常用检查** |

|------------------------|--------------------------------------|--------------------------------------------|

| **影像学** | 骶髂关节MRI（骨髓水肿）、X线（晚期骨融合） | 腰椎X线/CT（骨质增生、椎间盘突出） |

| **实验室检查** | HLA-B27、CRP/ESR（炎症指标） | 通常正常，或仅局部肌酶升高 |

---

### **五、何时需高度怀疑AS？**

若背痛符合以下 **≥4项**，应尽早就诊风湿免疫科：

1️⃣ 年龄＜40岁

2️⃣ 隐匿起病（缓慢加重）

3️⃣ 活动后改善，休息无效

4️⃣ 夜间痛（伴翻身困难）

5️⃣ 晨僵＞30分钟

6️⃣ HLA-B27阳性或家族史

---

### **六、下一步建议**

1. **优先排查AS**：

- **必查项目**：骶髂关节MRI、HLA-B27、CRP/ESR。

- **就诊科室**：风湿免疫科（非骨科！）。

2. **临时缓解措施**：

- **热敷**：40℃热毛巾敷疼痛部位，每日2次。

- **拉伸**：每日做“猫牛式”脊柱伸展（跪姿拱背/塌腰交替）。

- **避免久坐**：每小时起身活动5分钟。

3. **禁忌**：

✖️ 自行长期服用止痛药（掩盖炎症指标，延误诊断）。

✖️ 暴力推拿/正骨（可能加重脊柱不稳）。

---

**早期明确诊断是阻止AS骨融合的关键！** 若符合上述特征，请72小时内就医。

9.以下是医生可能将你的 **胸口疼痛** 与 **强直性脊柱炎（AS）** 关联的原因，以及需要排查的其他可能性：

---

### **一、强直性脊柱炎（AS）为何可能导致胸痛？**

#### **1. 胸廓受累的炎症机制**

- **肋椎关节/胸锁关节炎**：AS的炎症可累及肋骨与脊柱、胸骨的连接处，导致 **胸壁深部钝痛**，深呼吸、咳嗽时加重。

- **胸廓扩张受限**：AS晚期可能因骨融合导致胸廓活动度下降（正常胸廓扩张度≥2.5cm，AS患者常＜2cm）。

- **胸骨柄炎**：胸骨正中压痛，可能误以为是“心脏痛”。

#### **2. 其他AS相关胸痛原因**

- **主动脉根部病变**：AS患者主动脉瓣关闭不全风险增加，可能引发胸痛（需心脏超声排查）。

- **骨质疏松**：长期炎症或激素治疗导致胸椎压缩骨折（中老年AS患者需警惕）。

---

### **二、医生怀疑AS的“关键线索”**

1. **既往症状支持AS**

- 已存在的 **炎性背痛**（夜间痛、晨僵＞30分钟、活动缓解）。

- **家族史**（亲属确诊AS或其他脊柱关节炎）。

2. **胸痛特征符合AS模式**

- ✅ 疼痛与呼吸/体位改变相关（如转身、深呼吸时加重）。

- ✅ 胸壁压痛明显（非心脏压榨感）。

- ✅ 伴随其他AS症状（如足跟痛、葡萄膜炎）。

---

### **三、需紧急排除的致命性胸痛**

即使怀疑AS，也必须优先排除以下急症：

| **疾病** | **胸痛特点** | **关键检查** |

|------------------|-------------------------------|--------------------------|

| **心绞痛/心梗** | 压榨感、向左臂/下颌放射 | 心电图、心肌酶谱 |

| **肺栓塞** | 突发呼吸困难+咯血 | D-二聚体、CT肺动脉造影 |

| **主动脉夹层** | 撕裂样剧痛，背部放射 | 胸腹主动脉CTA |

**若胸痛伴随以下情况，立即挂急诊**：

⚠️ 大汗、恶心、濒死感

⚠️ 突发呼吸困难或晕厥

⚠️ 血压骤升或四肢脉搏不对称

---

### **四、确诊AS相关胸痛的核心步骤**

#### **1. 针对性检查**

- **AS相关**：

🔹 胸椎/胸骨MRI（评估肋椎关节炎症）

🔹 胸廓扩张度测量（站立位深吸气与深呼气胸围差）

🔹 HLA-B27、CRP/ESR复查

- **排除其他病因**：

🔹 心电图、心脏超声（排除心源性）

🔹 胸部X线/CT（排查肺炎、气胸）

#### **2. 治疗试验**

- 若使用 **非甾体抗炎药（如塞来昔布）** 后胸痛显著缓解，支持炎症性病因（如AS）。

---

### **五、日常管理与注意事项**

1. **急性期缓解**：

- 避免深呼吸/大笑等加剧疼痛的动作。

- 热敷疼痛部位（40℃热毛巾，每日2次）。

2. **长期应对**：

- **呼吸训练**：练习腹式呼吸（减少胸廓活动负担）。

- **姿势矫正**：避免含胸驼背，使用靠垫维持脊柱直立。

3. **禁忌**：

✖️ 自行推拿胸部关节（可能加重炎症）。

✖️ 盲目使用激素（需医生评估炎症程度后决定）。

---

### **六、总结：医生的逻辑与你的行动**

1. **为何怀疑AS**：胸廓关节炎症是AS的典型关节外表现，结合你既往的炎性背痛特征，医生需优先排查。

2. **必须做的检查**：心电图、胸椎MRI、炎症指标。

3. **下一步**：

- 若胸痛剧烈或伴随危险信号→ **立即急诊**。

- 若稳定→ 尽快预约 **风湿免疫科+心血管内科** 联合评估。

**AS相关胸痛多为可控的炎症反应，但必须排除致命性疾病！** 及时检查是安全的核心。

10.腰痛与情绪压力确实可能存在密切关联，这种关系涉及 **生理-心理-社会因素** 的复杂互动。以下是科学解释与针对性建议：

---

### **一、情绪压力如何诱发或加重腰痛？**

#### **1. 生理机制**

- **肌肉张力升高**：压力触发“战斗或逃跑”反应，导致 **腰背部肌肉持续紧绷**（如竖脊肌、腰方肌），引发缺血性疼痛。

- **炎症反应增强**：长期压力促使皮质醇分泌紊乱，加剧全身低度炎症，可能激活背部的疼痛信号通路。

- **神经敏感化**：压力通过中枢神经系统放大疼痛感知（中枢敏化），使原本轻微的腰痛被感知为剧烈疼痛。

#### **2. 心理行为机制**

- **疼痛-压力恶性循环**：

🔄 腰痛→活动受限→焦虑/抑郁→肌肉进一步紧张→疼痛加重。

- **行为改变**：压力大时可能久坐不动、姿势不良（如蜷缩驼背），加速腰椎退变。

---

### **二、支持情绪相关性腰痛的关键证据**

1. **研究数据**：

- 慢性腰痛患者中，约30%合并焦虑或抑郁（普通人群约10%）。

- 心理压力评分高的人群，腰痛复发风险增加2-3倍。

2. **临床特征**：

- 疼痛范围模糊、游走（非固定点疼痛）。

- 对治疗反应差，但影像学检查无严重器质性病变。

---

### **三、管理建议：打破“压力-疼痛”循环**

#### **1. 生理干预**

- **放松肌肉**：每日练习 **渐进式肌肉放松法**（依次收紧-放松全身肌群）。

- **有氧运动**：快走、游泳（每周≥150分钟），促进内啡肽分泌，缓解压力性肌肉紧张。

- **热敷+按摩**：用热盐袋敷腰部15分钟，配合拇指按压腰眼穴（第4腰椎棘突旁开3寸）。

#### **2. 心理干预**

- **认知行为疗法（CBT）**：识别并纠正“疼痛灾难化”思维（如“我永远好不了”）。

- **正念冥想**：每日10分钟专注呼吸，降低压力激素（皮质醇）水平。

- **情绪日记**：记录压力事件与腰痛强度的关联，寻找触发因素。

#### **3. 生活方式调整**

- **睡眠优化**：保证7-8小时睡眠（睡眠不足加剧疼痛敏感度）。

- **社交支持**：加入腰痛患者支持小组，减少孤独感。

- **避免咖啡因/酒精**：这些物质可能加重焦虑和肌肉紧张。

---

### **四、何时需寻求专业帮助？**

1. **心理科/心身医学科就诊信号**：

- 腰痛伴随持续情绪低落、失眠、兴趣丧失＞2周。

- 疼痛程度与影像学检查结果不匹配（如轻微椎间盘突出但剧痛）。

2. **多学科联合治疗**：

- 风湿科/骨科排除器质性疾病后，联合心理科制定生物-心理-社会整合治疗方案。

---

### **五、重要提醒**

- **勿忽视器质性疾病**：情绪相关性腰痛需在排除腰椎间盘突出、AS等疾病后诊断。

- **短期用药**：严重焦虑时可短期使用SSRI类药物（需医生指导），但非根本解决之道。

**你的腰痛可能是身心共同发出的信号**，综合干预才能标本兼治！

11.HLA-B27阳性是强直性脊柱炎（AS）等脊柱关节病的重要参考指标，但它本身不能单独作为确诊依据。以下是需要您了解的关键信息：

---

### 1. **HLA-B27与强直性脊柱炎的关系**

- **强关联性**：约90%的强直患者HLA-B27呈阳性，但阳性≠必然患病。

- **基因易感性**：HLA-B27是免疫系统相关基因，阳性者患AS的风险显著升高，但具体机制尚未完全明确。

- **种族差异**：亚洲人群HLA-B27阳性率约4-8%，而强直患者中阳性率远高于此，需结合其他指标综合判断。

---

### 2. **阳性结果的临床意义**

- **支持诊断**：若存在慢性炎症性腰背痛（持续3个月以上，晨僵、活动后减轻）、骶髂关节炎（影像学证据）等症状，HLA-B27阳性可辅助确诊AS。

- **排除性意义**：阴性结果可降低AS可能性，但极少数AS患者（约10%）仍可能为阴性。

- **关联其他疾病**：阳性也可能与银屑病关节炎、反应性关节炎、炎症性肠病相关关节炎、急性前葡萄膜炎等相关。

---

### 3. **下一步建议**

- **完善检查**：需结合骶髂关节X线/MRI（观察炎症或结构损伤）、血沉（ESR）/C反应蛋白（CRP）（炎症指标）、关节活动度评估等。

- **关注症状**：记录疼痛特点（夜间加重、晨僵＞30分钟）、外周关节肿痛、足跟痛、眼部炎症等细节。

- **遗传咨询**：HLA-B27可遗传，直系亲属患病风险略增，但无需过度担忧。

---

### 4. **您需要知道的注意事项**

- **无需治疗基因**：HLA-B27阳性本身无需干预，重点在于控制已出现的炎症。

- **定期随访**：即使目前无症状，建议长期关注关节和眼部健康。

- **生活方式**：规律锻炼（如游泳、瑜伽）、避免久坐、睡硬板床有助于延缓病情进展。

---

请与医生充分沟通您的症状和检查结果，综合制定诊疗计划。早期干预可显著改善预后，不必因单一指标阳性过度焦虑。

12.C-反应蛋白（CRP）和血沉（ESR）升高与强直性脊柱炎（AS）可能存在关联，但需结合其他症状和检查综合分析。以下是您需要了解的信息：

---

### **1. CRP和血沉升高的意义**

- **炎症标志物**：两者均反映体内存在炎症反应，但无法直接指向具体疾病。

- **与AS的关联**：

- **活动期AS**：约50-70%的强直患者会出现CRP/血沉升高，提示疾病处于活动期（如关节炎症活跃）。

- **非特异性**：数值高低与症状严重程度不完全一致，部分AS患者即使病情进展，炎症指标也可能正常。

---

### **2. 需排除的其他可能性**

若您尚未确诊AS，需考虑其他导致CRP/血沉升高的疾病：

- **感染**：如细菌/病毒感染、结核等。

- **其他自身免疫病**：类风湿关节炎、银屑病关节炎、系统性红斑狼疮等。

- **局部炎症**：牙周炎、泌尿生殖系统炎症等。

- **肿瘤或组织损伤**：如手术、外伤后。

---

### **3. 结合AS诊断的关键点**

仅凭CRP/血沉升高不能确诊AS，需同时满足以下条件：

- **典型症状**：慢性炎症性腰背痛（夜间加重、晨僵＞30分钟、活动后缓解）、交替性臀部疼痛、足跟痛、外周关节炎等。

- **影像学证据**：骶髂关节炎（X线或MRI显示骨质破坏或炎症）。

- **HLA-B27检测**：阳性结果可支持诊断（但非必需）。

---

### **4. 下一步建议**

- **完善影像学检查**：优先进行骶髂关节MRI（早期炎症敏感）或X线（中晚期结构损伤）。

- **排查感染/其他疾病**：如近期有发热、咳嗽、尿痛等症状，需配合血常规、尿常规等检查。

- **动态监测**：定期复查CRP/血沉，观察数值变化趋势（治疗有效时可能下降）。

---

### **5. 注意事项**

- **无需恐慌**：炎症指标升高仅提示需要进一步排查，不代表病情严重。

- **早期干预**：若确诊AS，尽早使用非甾体抗炎药（NSAIDs）或生物制剂控制炎症，可延缓关节损伤。

- **生活方式调整**：适度运动（如游泳、瑜伽）、避免久坐、戒烟（吸烟加重炎症）。

---

请将检查结果与具体症状（如疼痛特点、持续时间）结合，与医生讨论后续诊疗方案。

13.---

### **核心原因解析**

即使X光片未显示异常，但您仍感到剧烈疼痛，可能与以下因素相关：

---

#### **1. X光片的局限性**

- **早期病变无法捕捉**：

X光主要显示骨骼结构变化（如骨质破坏、关节融合），但强直性脊柱炎（AS）早期炎症多集中在软组织、滑膜或骨髓，此时X光可能完全正常。

**示例**：AS的骶髂关节炎需达到Ⅱ级（轻度硬化或侵蚀）才能在X光上显现，而早期Ⅰ级病变（骨髓水肿）只能通过MRI发现。

- **疼痛来源非骨骼**：

疼痛可能源于肌肉痉挛、韧带炎症或神经压迫（如椎间盘突出），这些软组织问题X光无法显示。

---

#### **2. 需考虑的其他疾病**

若医生已排除AS，需排查以下疼痛原因：

- **软组织损伤**：腰肌劳损、筋膜炎（久坐或运动不当引发）。

- **神经性疼痛**：坐骨神经痛、椎间盘突出（需结合CT/MRI明确）。

- **纤维肌痛**：广泛性肌肉疼痛伴疲劳，无影像学异常。

- **内脏牵涉痛**：肾结石、盆腔炎症等内脏疾病可能放射至腰背。

---

#### **3. 针对AS的进一步排查**

若怀疑AS但X光阴性，建议：

- **骶髂关节MRI**：检测骨髓水肿、滑膜炎等早期炎症（AS特征性表现）。

- **动态观察**：3-6个月后复查X光，观察是否出现缓慢进展的骨质破坏。

- **结合实验室指标**：HLA-B27、CRP/血沉升高可增加AS可能性。

---

#### **4. 疼痛管理建议**

无论病因是否明确，可采取以下措施缓解疼痛：

- **药物**：非甾体抗炎药（如塞来昔布）对炎症性疼痛效果显著。

- **理疗**：热敷、低频电刺激、针灸改善局部血液循环。

- **运动康复**：核心肌群训练（如平板支撑）、游泳减轻脊柱负荷。

---

### **关键行动清单**

1. **尽快完善MRI检查**（尤其骶髂关节和脊柱）。

2. **记录疼痛规律**：是否夜间加重、晨僵持续时间、活动后是否缓解。

3. **排除其他疾病**：尿常规（排查肾病）、妇科/泌尿科检查（排除盆腔疾病）。

---

若MRI仍无异常，需考虑非器质性疼痛（如心因性因素），但务必优先排除器质性疾病。与医生保持沟通，明确后续随访计划。

14.---

### **核心答案速览**

**骨髓水肿本身并非疾病，而是炎症或损伤的表现**，可能与强直性脊柱炎（AS）相关，但需结合其他症状和检查综合判断。以下是详细分析：

---

### **1. 骨髓水肿的临床意义**

- **本质**：骨髓水肿是骨髓腔内液体异常聚集（炎症、微骨折或充血），在MRI上表现为高信号。

- **严重性**：

- **可逆性**：及时干预（如抗炎治疗）可能完全吸收，不留后遗症。

- **进展风险**：若长期未控制，可能发展为骨侵蚀、纤维化或骨赘形成（如AS的骨桥）。

---

### **2. 骨髓水肿与强直性脊柱炎的关系**

- **AS的典型表现**：

AS早期骶髂关节炎常伴骨髓水肿（MRI特征性改变），是诊断“中轴型脊柱关节炎”的重要依据。

**提示AS的线索**：

✅ 骨髓水肿集中在骶髂关节或脊柱椎体边缘

✅ 伴随慢性炎症性腰背痛（夜间痛、晨僵＞30分钟）

✅ HLA-B27阳性、CRP/血沉升高

- **非AS的可能性**：

若骨髓水肿出现在其他部位（如膝关节、髋关节），需排查创伤、感染、骨关节炎或类风湿关节炎。

---

### **3. 明确诊断的关键步骤**

| **检查/症状** | **意义** |

|-------------------------|-------------------------------------------------------------------------|

| **骶髂关节MRI** | 确认骨髓水肿是否对称性分布于骶髂关节（AS典型表现） |

| **HLA-B27检测** | 阳性结果支持AS，但阴性不排除 |

| **炎症指标（CRP/血沉）** | 持续升高提示炎症活跃，需警惕AS活动期 |

| **疼痛特点** | 是否符合AS的“炎症性背痛”（活动后减轻、休息无缓解、夜间痛醒） |

---

### **4. 下一步建议**

- **完善AS诊断标准**：

若存在以下两项，需高度怀疑AS：

➔ 骶髂关节炎（MRI或X线证实）

➔ ≥1个AS特征性症状（如葡萄膜炎、指炎、足跟痛）

- **鉴别其他疾病**：

➔ 短期剧烈疼痛+骨髓水肿：排查感染（如骨髓炎）或应力性骨折

➔ 多关节受累：类风湿关节炎、银屑病关节炎

- **治疗方向**：

➔ 若倾向AS：尽早使用非甾体抗炎药（NSAIDs）或生物制剂抑制炎症，延缓骨破坏

➔ 若病因不明：短期抗炎治疗+3-6个月后复查MRI观察水肿变化

---

### **5. 您需要关注的预警信号**

- **提示病情进展**：

🔴 疼痛从腰骶部向胸椎、颈椎蔓延

🔴 晨僵时间延长（＞1小时）

🔴 出现驼背、呼吸受限（胸廓活动度下降）

- **需立即就医的情况**：

🔴 突发视力下降（警惕急性前葡萄膜炎）

🔴 下肢麻木或无力（警惕脊柱压迫）

---

### **总结**

骨髓水肿可能是AS的早期信号，但需结合影像学、实验室指标和症状综合判断。**不必过度焦虑，但务必规范就诊**，早期治疗可显著改善预后。

15.---

### **自我初步评估与确诊强直性脊柱炎（AS）的关键步骤**

---

#### **一、需警惕的典型症状**

若以下症状持续≥3个月，需怀疑AS可能：

1. **炎症性腰背痛**

- ✅ **夜间痛醒**：睡眠中因疼痛醒来，活动后缓解。

- ✅ **晨僵＞30分钟**：早晨起床时腰部僵硬，活动后减轻。

- ✅ **臀部交替痛**：单侧或双侧臀部深处疼痛，可能放射至大腿。

2. **外周关节症状**

- ✅ 足跟痛（跟腱炎）

- ✅ 手指/脚趾肿胀（指炎/趾炎）

3. **关节外表现**

- ✅ 反复发作的急性前葡萄膜炎（眼睛红、痛、畏光）

- ✅ 银屑病皮疹或炎症性肠病（如克罗恩病）

---

#### **二、必须完成的医学检查**

| **检查类型** | **目的** |

|--------------------|-------------------------------------------------------------------------|

| **骶髂关节影像学** | 骶髂关节炎是AS的核心诊断依据：<br>- **X线**：观察骨质破坏、关节间隙变化（适合中晚期）<br>- **MRI**：检测早期骨髓水肿、滑膜炎（适合症状明显但X线阴性者） |

| **HLA-B27检测** | 阳性结果支持诊断，但阴性不排除AS（约10%患者阴性） |

| **炎症指标** | CRP/血沉升高提示疾病活动，但部分患者指标正常 |

| **关节活动度评估** | 医生检查胸廓扩张度、Schober试验（腰椎活动度）等 |

---

#### **三、诊断标准参考（简化版）**

若满足以下条件，需高度怀疑AS：

1. **影像学确认骶髂关节炎**（X线或MRI显示单侧Ⅲ级或双侧Ⅱ级以上病变）。

2. **符合≥1项AS特征症状**：

- 炎症性腰背痛

- 足跟痛/指炎

- 葡萄膜炎

- 对NSAIDs（如布洛芬）反应良好

---

#### **四、自我排查与就医建议**

1. **记录症状日记**

- 疼痛部位、持续时间、夜间是否加重、晨僵时长。

- 拍照记录关节肿胀或皮肤异常（如银屑病）。

2. **优先排查项目**

- 骶髂关节MRI（尤其年轻患者或早期症状者）。

- 风湿免疫科专科就诊（避免误诊为腰肌劳损或腰椎间盘突出）。

3. **鉴别诊断**

- 类风湿关节炎（对称性小关节肿痛）。

- 致密性骨炎（多见于育龄女性，骶髂关节骨密度增高但无破坏）。

---

#### **五、注意事项**

- **勿自行诊断**：即使症状高度符合，仍需医生结合检查确诊。

- **早诊早治**：发病前5年是黄金治疗期，可显著延缓关节强直。

- **避免误区**：

❌ 仅凭HLA-B27阳性确诊AS

❌ 依赖膏药、按摩代替抗炎治疗

❌ 忽视眼睛、肠道等关节外症状

---

### **行动清单**

1. **立即就医**：挂风湿免疫科，完善骶髂关节MRI和HLA-B27检测。

2. **短期对症处理**：疼痛明显时可短期服用非甾体抗炎药（如塞来昔布），观察是否缓解。

3. **生活方式干预**：

- 每日拉伸（猫牛式、骨盆倾斜运动）。

- 睡硬板床，避免久坐和弯腰负重。

---

**提示**：AS的诊断需综合症状、影像和实验室结果，即使初步怀疑，也请保持冷静，规范就诊可有效控制病情。

16.---

### **核心回答**

**有必要按照医生建议完成所有检查**。X光、CT、MRI和抽血化验在强直性脊柱炎（AS）的诊断中各司其职，互为补充。以下是具体分析：

---

### **1. 各项检查的核心作用**

| **检查类型** | **优势** | **针对AS的用途** | **局限性** |

|--------------|----------|------------------|------------|

| **X光** | 快速、低成本 | 观察骶髂关节骨质破坏、脊柱骨桥形成（中晚期病变） | 早期炎症（如骨髓水肿）无法显示 |

| **CT** | 高分辨率骨骼成像 | 清晰显示细微骨侵蚀、关节间隙变化 | 辐射量较高，软组织炎症显示不足 |

| **MRI** | 无辐射、软组织敏感 | 检测早期骨髓水肿、滑膜炎（AS活动期关键证据） | 费用较高，对钙化/骨桥显示不如CT |

| **抽血化验** | 评估全身状态 | 检测炎症指标（CRP/血沉）、HLA-B27基因、排除其他疾病（如类风湿因子） | 无法直接确诊AS |

---

### **2. 为什么需要联合检查？**

- **避免漏诊**：

AS早期（起病1-2年内）X光可能完全正常，需MRI捕捉骨髓水肿；而X光/CT可明确晚期结构损伤。

- **鉴别诊断**：

抽血化验可区分AS与类风湿关节炎（RF/CCP阴性）、感染性疾病（白细胞升高）。

- **全面评估**：

MRI显示炎症活动度，X光/CT显示骨质破坏程度，血液指标指导用药（如生物制剂需排除结核感染）。

---

### **3. 不做的潜在风险**

| **若跳过检查** | **可能后果** |

|----------------|-------------|

| **拒绝MRI** | 漏诊早期AS，延误治疗导致不可逆骨融合 |

| **拒绝X光/CT** | 无法评估骨质破坏进展，影响分期和治疗选择 |

| **拒绝抽血** | 无法排除感染/其他免疫病，增加误用激素或生物制剂的风险 |

---

### **4. 关于辐射的顾虑（CT/X光）**

- **实际风险**：单次骶髂关节CT辐射约3-5mSv（相当于自然环境中1年辐射量），远低于致癌阈值（100mSv）。

- **权衡利弊**：若疑似AS，明确诊断的收益远大于辐射风险，且医生会控制检查频率。

---

### **5. 优化检查的策略**

- **分步进行**：

1️⃣ 优先完成骶髂关节MRI和抽血（HLA-B27、CRP/血沉）；

2️⃣ 若MRI提示炎症，补做X光或CT评估骨质破坏；

3️⃣ 脊柱症状明显时加做脊柱MRI。

- **替代方案**：

➔ 经济受限时：与医生沟通优先做MRI和抽血，暂缓CT；

➔ 担心辐射：用MRI替代CT（但CT对骨细节更清晰）。

---

### **6. 什么情况下可减少检查？**

- **高度典型症状+明确影像证据**：如MRI已显示骶髂关节炎且HLA-B27阳性，X光或CT可酌情省略。

- **随访期**：若已确诊AS，复查时可能仅需抽血（监测炎症指标）和针对性影像。

---

### **总结**

**务必配合完成所有检查**，尤其是初诊阶段。AS的诊断需“症状+影像+化验”三联印证，单一检查无法全面评估。若有特殊困难（如经济或时间限制），可与医生协商调整顺序，但勿自行删减项目。

17.---

### **核心回答**

**会。强直性脊柱炎（AS）并非“青少年专属病”**，尽管高发于15-30岁人群，但40岁以上成人仍可能患病（称为“晚发型AS”）。以下是您需要了解的详细信息：

---

### **1. AS的发病年龄范围**

- **主要人群**：约80%的AS患者在45岁前确诊，高峰期为20-30岁。

- **晚发型AS**：

- **定义**：≥45岁首次出现症状并确诊（占AS患者约5-10%）。

- **特点**：

✅ 脊柱症状较轻，但外周关节（如膝、踝）受累更常见。

✅ 骶髂关节炎进展较慢，HLA-B27阳性率略低（约60-70% vs 青少年AS的90%）。

---

### **2. 成年人出现AS的预警信号**

若您有以下症状，需警惕AS可能：

- **炎症性腰背痛**：夜间痛醒、晨僵＞30分钟、活动后缓解（区别于腰椎间盘突出的机械性疼痛）。

- **交替性臀部疼痛**：单侧或双侧臀部深处钝痛，可能误诊为“坐骨神经痛”。

- **关节外表现**：反复发作的急性前葡萄膜炎（眼红、畏光、视力模糊）。

---

### **3. 成年人需重点鉴别的疾病**

| **疾病** | **与AS的差异点** |

|-----------------------|------------------------------------------|

| **骨关节炎** | 疼痛活动后加重，晨僵＜30分钟，无夜间痛醒 |

| **弥漫性特发性骨肥厚（DISH）** | 多见于50岁以上，脊柱骨赘形成但骶髂关节正常 |

| **类风湿关节炎** | 对称性小关节（手指、手腕）肿痛，类风湿因子阳性 |

---

### **4. 确诊建议**

- **关键检查**：

➔ **骶髂关节MRI**：即使X光正常，MRI可发现早期骨髓水肿。

➔ **HLA-B27检测**：阳性结果支持诊断（但阴性不排除）。

- **就诊科室**：风湿免疫科（非骨科或疼痛科），避免漏诊。

---

### **5. 注意事项**

- **勿因年龄自我排除**：有60岁以上AS新确诊案例，尤其有家族史者。

- **关注非典型症状**：成人AS可能以足跟痛、指炎或葡萄膜炎为首发表现。

- **治疗差异**：成人AS对非甾体抗炎药（NSAIDs）反应可能不如青少年敏感，需个体化方案。

---

### **总结**

年龄不是AS的绝对门槛，若出现疑似症状（如夜间腰背痛、晨僵），**无论年龄均应及时就诊**。早诊早治可显著延缓疾病进展，最大限度保持关节功能。

18.---

### **强直性脊柱炎（AS）易被误诊的疾病及鉴别要点**

强直性脊柱炎因症状多样且早期表现不典型，常被误诊为以下疾病：

---

#### **1. 腰椎间盘突出/腰肌劳损**

- **误诊原因**：均以腰背痛为主诉。

- **鉴别要点**：

✅ **疼痛性质**：

- AS：炎症性疼痛（夜间加重、晨僵＞30分钟、活动后缓解）。

- 腰椎间盘突出：机械性疼痛（活动后加重，休息缓解）。

✅ **影像学差异**：

- AS：骶髂关节MRI可见骨髓水肿，X线后期显示骨桥形成。

- 腰椎间盘突出：CT/MRI显示椎间盘突出压迫神经根。

---

#### **2. 类风湿关节炎（RA）**

- **误诊原因**：外周关节肿痛重叠。

- **鉴别要点**：

✅ **受累关节**：

- AS：以脊柱、骶髂关节为主，外周关节多为下肢大关节（膝、踝）。

- RA：对称性小关节（手指、腕关节）受累。

✅ **实验室检查**：

- AS：类风湿因子（RF）阴性，HLA-B27可能阳性。

- RA：RF和抗CCP抗体常阳性。

---

#### **3. 纤维肌痛**

- **误诊原因**：广泛性肌肉疼痛和疲劳感相似。

- **鉴别要点**：

✅ **疼痛特点**：

- AS：定位明确（腰骶部、臀部），伴随晨僵和活动受限。

- 纤维肌痛：全身多部位游走性疼痛，无明确炎症证据。

✅ **检查结果**：

- AS：CRP/血沉升高，影像学可见骶髂关节炎。

- 纤维肌痛：炎症指标正常，无影像学异常。

---

#### **4. 致密性骨炎**

- **误诊原因**：骶髂关节影像学改变（X线显示骨密度增高）。

- **鉴别要点**：

✅ **人群特征**：

- AS：多见于男性。

- 致密性骨炎：育龄女性为主（尤其妊娠后）。

✅ **症状差异**：

- AS：动态进展性疼痛，伴脊柱活动受限。

- 致密性骨炎：疼痛较轻，无晨僵或夜间痛。

---

#### **5. 银屑病关节炎（PsA）**

- **误诊原因**：脊柱受累和HLA-B27阳性可能重叠。

- **鉴别要点**：

✅ **皮肤表现**：

- PsA：银屑病皮疹或指甲病变（顶针样凹陷）。

- AS：无典型皮肤症状。

✅ **影像学特征**：

- PsA：脊柱骨赘多为非对称性，可伴“笔帽样”指骨改变。

---

#### **6. 反应性关节炎/感染性关节炎**

- **误诊原因**：关节炎和HLA-B27阳性。

- **鉴别要点**：

✅ **感染史**：

- 反应性关节炎：发病前有泌尿系或肠道感染史。

- AS：无明确感染触发因素。

✅ **病程**：

- 反应性关节炎：多为自限性（3-6个月缓解）。

---

#### **7. 骨质疏松**

- **误诊原因**：腰背痛、驼背等重叠症状。

- **鉴别要点**：

✅ **疼痛特点**：

- 骨质疏松：机械性疼痛（活动负重后加重），无晨僵。

✅ **骨密度检测**：

- 骨质疏松：T值≤-2.5。

- AS：骨密度可能正常或局部升高（骨赘形成）。

---

### **易误诊的核心原因**

1. **症状早期不典型**：AS起病隐匿，仅表现为间歇性腰酸。

2. **过度依赖单一检查**：仅凭X光正常或HLA-B27阴性排除AS。

3. **医生认知局限**：非风湿科医生对炎症性背痛特征不熟悉。

---

### **如何减少误诊？**

- **就诊科室**：优先选择风湿免疫科。

- **完善检查**：骶髂关节MRI（早期诊断金标准）+ HLA-B27检测。

- **详细记录症状**：重点描述夜间痛、晨僵、活动缓解特点。

---

**提示**：若曾被诊断为上述疾病但治疗无效，需重新评估AS可能！

19.---

### **逐步解决方案：当医生排除AS但您仍有疑虑时**

即使多位医生认为您不符合强直性脊柱炎（AS）的诊断标准，但您的症状持续存在，以下是为您量身定制的行动指南：

---

#### **一、重新梳理症状：建立客观记录**

1. **制作症状日记**

- **记录内容**：

✅ 疼痛部位（如腰骶部、臀部、足跟）

✅ 疼痛时间（是否夜间加重、晨僵持续时间）

✅ 活动影响（活动后是否缓解、久坐后是否僵硬）

✅ 伴随症状（如眼睛红痛、肠道不适、皮疹）

- **工具建议**：使用手机APP（如“疼痛日志”）或纸质表格每日填写。

2. **量化症状严重度**

- 用1-10分评估疼痛强度，标记止痛药使用频率及效果。

- 拍摄关节肿胀或活动受限的视频（如弯腰、转身困难）。

---

#### **二、获取第二意见：精准选择医生与检查**

1. **锁定专科医生**

- **优先选择**：三甲医院风湿免疫科副主任医师及以上职称（经验更丰富）。

- **就诊准备**：携带既往所有检查报告（X光、MRI、血液化验）及症状日记。

2. **针对性要求检查**

- **关键项目**：

➔ **骶髂关节MRI（脂肪抑制序列）**：即使X光正常，可发现早期骨髓水肿。

➔ **HLA-B27复查**：部分实验室可能出现假阴性，换机构复测。

➔ **炎症指标动态监测**：连续2-3次检测CRP/血沉（避免单次正常误导）。

3. **明确提问**

- 询问医生：“我的症状不符合AS的诊断标准，最可能的原因是什么？”

- 要求医生解释：“哪些检查或症状排除了AS？是否有其他类似疾病需排查？”

---

#### **三、排查其他疾病：系统性鉴别诊断**

| **可能误诊疾病** | **鉴别检查** | **症状差异点** |

|-----------------------|---------------------------------------|------------------------------------------|

| **纤维肌痛** | 压痛点评估、排除性诊断 | 广泛性疼痛，无晨僵或影像学异常 |

| **银屑病关节炎** | 皮肤科会诊（查银屑病皮疹/指甲病变） | 皮肤鳞屑、指甲顶针样凹陷 |

| **反应性关节炎** | 询问近期感染史（肠道/泌尿生殖） | 突发关节炎，多与感染相关，3-6个月自限 |

| **弥漫性特发性骨肥厚（DISH）** | 脊柱X光（前纵韧带钙化） | 多见于50岁以上，无骶髂关节炎 |

---

#### **四、探索功能医学与辅助评估**

1. **炎症深度检测**（若常规检查阴性）

- **可选项目**：IL-17、TNF-α等细胞因子检测（部分AS患者升高）。

- **注意**：此类项目需在专科医生指导下进行，避免过度检查。

2. **基因检测扩展**

- 除HLA-B27外，检测ERAP1、IL23R等AS相关基因（研究级项目，临床意义待明确）。

---

#### **五、制定症状管理计划**

即使未确诊AS，仍需缓解当前不适：

1. **药物干预**

- 短期使用非甾体抗炎药（如依托考昔）测试效果：若疼痛显著缓解，提示炎症性病因。

2. **物理治疗**

- **针对性方案**：

➔ 热疗+超声波：缓解肌肉痉挛。

➔ 脊柱稳定性训练（普拉提）：增强核心肌群。

3. **心理支持**

- 慢性疼痛常伴焦虑/抑郁，可考虑认知行为疗法（CBT）或心理咨询。

---

#### **六、终极确认：诊断性治疗**

若仍高度怀疑AS但证据不足，可与医生协商尝试：

- **诊断性治疗**：规范使用NSAIDs 2-4周，观察症状改善（AS患者通常反应显著）。

- **生物制剂试探**：在极少数情况下，若其他治疗无效且影像学高度可疑，短期使用TNF抑制剂（如阿达木单抗）观察疗效。

---

### **关键提醒**

- **避免自我标签**：过度关注AS可能加重“躯体化症状”，保持开放心态。

- **时效性随访**：即使当前无AS证据，建议每6-12个月复查骶髂关节MRI（尤其症状持续或加重时）。

- **法律保障**：若多次就诊仍无法明确诊断，可通过医院医务科申请多学科会诊（MDT）。

---

### **行动清单**

1. **本周内**：完成症状日记模板，预约风湿免疫科专家号。

2. **1个月内**：完成骶髂关节MRI（优先选择3.0T设备）及HLA-B27复查。

3. **3个月内**：根据检查结果决定是否启动诊断性治疗。

---

**最终建议**：医学存在不确定性，但您的健康值得持续追踪。即使最终排除AS，也应与医生合作找到症状的真正根源，实现精准治疗。

20.---

### **核心建议：未确诊但疼痛时的安全用药与自我管理**

即使尚未明确诊断，您仍可在医生指导下采取以下措施缓解疼痛。**请严格遵循以下原则**：

⚠️ **注意**：用药仅为对症处理，需同步排查病因，避免掩盖潜在疾病进展。

---

#### **一、可短期使用的安全药物**

| **药物类型** | **推荐药物** | **适用情况** | **用法与注意事项** |

|-----------------------|--------------------|----------------------------------|-----------------------------------------------------------------------------------|

| **非甾体抗炎药（NSAIDs）** | 布洛芬、塞来昔布 | 炎症性疼痛（夜间加重、晨僵明显） | ➔ **剂量**：布洛芬400mg每8小时，塞来昔布200mg每日1次<br>➔ **禁忌**：胃溃疡、肾功能不全慎用，连续使用≤7天 |

| **对乙酰氨基酚** | 泰诺林 | 机械性/非炎症性疼痛 | ➔ **剂量**：500mg每6-8小时，每日最大4g<br>➔ **禁忌**：肝功能异常者禁用 |

| **肌松药** | 乙哌立松 | 肌肉痉挛性疼痛 | ➔ **剂量**：50mg每日3次，短期使用（≤3天）<br>➔ **副作用**：嗜睡、乏力 |

---

#### **二、药物使用关键原则**

1. **优先选择NSAIDs**：若疼痛符合“炎症性特征”（活动后减轻、夜间加重），NSAIDs可能更有效。

2. **避免联用药物**：

- ❌ 同时使用两种NSAIDs（增加胃出血风险）。

- ❌ NSAIDs+糖皮质激素（如泼尼松）未经医生指导。

3. **监测预警信号**：

- 用药后出现黑便、腹痛→ 立即停药并就医（警惕消化道出血）。

- 疼痛持续＞1周无缓解→ 需重新评估病因。

---

#### **三、非药物疼痛管理方案**

1. **物理疗法**

- **热敷**：适用于肌肉僵硬（每次15-20分钟，每日2-3次）。

- **冷敷**：急性疼痛或肿胀时使用（冰袋包裹毛巾，每次10分钟）。

2. **运动干预**

- **核心训练**：平板支撑、桥式运动（增强脊柱稳定性，每日10-15分钟）。

- **拉伸放松**：猫牛式、骨盆倾斜（缓解腰背紧张，晨起/睡前各1组）。

3. **生活方式调整**

- 睡硬板床，避免久坐超过1小时。

- 使用腰部支撑靠垫，减轻脊柱压力。

---

#### **四、需立即就医的预警症状**

出现以下情况时，**停止自行用药并急诊**：

- 🔴 下肢麻木/无力（警惕神经压迫或脊髓损伤）。

- 🔴 发热伴疼痛（可能提示感染或肿瘤）。

- 🔴 突发剧烈胸痛或呼吸困难（排除心血管或胸椎病变）。

---

#### **五、下一步明确诊断的建议**

1. **完善关键检查**（若未完成）：

- 骶髂关节MRI（排查早期强直性脊柱炎）。

- 风湿免疫全套（ANA、RF、HLA-B27等）。

2. **分诊建议**：

- 疼痛以腰背为主→ 风湿免疫科。

- 伴下肢放射痛→ 骨科或神经外科。

- 伴皮疹/眼部症状→ 皮肤科或眼科。

---

### **总结**

**短期可安全使用NSAIDs或对乙酰氨基酚控制疼痛，但务必同步就医明确病因**。疼痛是身体的警示信号，盲目止痛可能延误诊断。若您已等待检查结果，可向医生要求加急处理或开具临时对症药物处方。

21.---

### **医学检查的辐射剂量与健康影响解析**

即使您需要多次进行影像学检查，也不必过度担忧辐射风险。以下是不同检查的辐射剂量及安全性的详细分析：

---

#### **一、常见检查的辐射剂量对比**

| **检查项目** | **典型辐射剂量（mSv）** | **等效自然环境暴露时间** | **备注** |

|----------------------------|------------------------|--------------------------|-----------------------------------|

| **胸部X光（正位）** | 0.1 mSv | 10天 | 相当于乘坐4小时飞机的辐射量 |

| **腰椎X光（正侧位）** | 1.5 mSv | 6个月 | 辐射集中于腰骶部 |

| **骶髂关节CT** | 3-5 mSv | 1-2年 | 低剂量CT可降至1-2 mSv |

| **全身PET-CT** | 20-25 mSv | 8-10年 | 含放射性药物（如18F-FDG） |

| **腹部CT平扫** | 8-10 mSv | 3-4年 | 儿童需调低剂量 |

| **MRI（任何部位）** | **0 mSv** | 无辐射 | 使用磁场和无线电波，无电离辐射 |

| **超声** | **0 mSv** | 无辐射 | 使用声波成像 |

---

#### **二、辐射安全阈值与风险**

1. **国际标准**：

- **普通人年安全限值**：≤50 mSv（国际辐射防护委员会, ICRP建议）。

- **致癌风险阈值**：>100 mSv（累积剂量超过此值，癌症风险略微增加）。

2. **实际风险计算**：

- 单次骶髂关节CT（5 mSv）的致癌风险：约 **0.05%**（即每2000人中可能增加1例癌症）。

- **对比**：吸烟者终生患癌风险约19%，自然患癌风险约25%。

3. **孕妇/儿童特殊保护**：

- 孕妇尽量避免腹部/盆腔CT，必要时用铅衣防护胎儿。

- 儿童采用“ALARA原则”（尽可能低剂量），设备自动调节辐射量。

---

#### **三、如何降低辐射风险？**

1. **优化检查选择**：

- 优先选择无辐射检查（如MRI、超声）。

- 用低剂量CT替代常规CT（剂量减少50%以上）。

2. **防护措施**：

- 穿戴铅围裙/颈套（尤其保护甲状腺和性腺）。

- 检查时保持静止，避免重复扫描。

3. **检查间隔管理**：

- 非必要不重复检查，尤其是CT（建议间隔≥6个月）。

- 记录既往检查的辐射剂量（部分医院提供“辐射护照”）。

---

#### **四、何时需警惕辐射风险？**

| **情况** | **建议** |

|-------------------------|-------------------------------------------------------------------------|

| **1年内多次CT检查** | 累计剂量＞20 mSv时，与医生讨论替代方案（如MRI） |

| **儿童或计划怀孕者** | 必须告知医生，优先选择MRI或超声 |

| **放射相关职业者** | 需记录职业暴露剂量（年上限20 mSv），避免医学检查叠加 |

---

#### **五、核心结论**

- **诊断收益＞辐射风险**：必要检查的辐射剂量远低于致癌阈值，延误诊断的危害更大。

- **无需恐慌单次检查**：一次骶髂关节CT（3-5 mSv）的辐射风险可忽略不计。

- **主动沟通**：检查前询问医生：“是否有无辐射的替代方案？能否用低剂量技术？”

---

### **行动清单**

1. **检查前**：告知医生您的辐射暴露史（如近期其他CT/X光）。

2. **检查中**：配合技师使用铅防护设备。

3. **检查后**：向医院索要辐射剂量报告，加入个人健康档案。

**提示**：现代影像设备已大幅降低辐射剂量，且医生会严格把控检查指征。理性看待辐射，优先确保疾病精准诊断！

22.---

### **核心原因解析**

即使HLA-B27阳性、血常规异常且症状符合强直性脊柱炎（AS），医生暂不确诊的原因可能涉及以下关键点：

---

#### **一、AS诊断的“金标准”未被满足**

强直性脊柱炎的诊断需同时满足 **临床症状+影像学证据**，单一指标异常不足以确诊：

1. **影像学门槛**：

- **必需条件**：X线或MRI显示明确的 **骶髂关节炎**（如X线双侧≥Ⅱ级或单侧≥Ⅲ级病变）。

- **您的情况**：若骶髂关节影像（X光/CT/MRI）未达到上述标准，即使其他指标异常，仍无法确诊。

2. **症状特异性不足**：

- 炎症性背痛（夜间加重、晨僵）可能与其他疾病重叠，如银屑病关节炎、反应性关节炎或炎症性肠病相关脊柱炎。

---

#### **二、血常规异常的局限性**

| **指标** | **可能意义** | **与AS的关联性** |

|----------------|-----------------------------|--------------------------------------|

| **白细胞升高** | 感染、应激反应、其他炎症疾病 | AS活动期可能正常或轻度升高，非特异性 |

| **血小板增高** | 慢性炎症、贫血、肿瘤 | AS患者可能因炎症反应继发升高 |

| **血沉/CRP高** | 体内存在炎症 | 约30-40%的AS患者炎症指标正常 |

**关键点**：血常规异常需排除感染、肿瘤等更紧急的疾病后才能归因于AS。

---

#### **三、HLA-B27阳性的意义与局限**

- **支持AS诊断**：阳性结果结合典型症状和影像学可强化诊断。

- **非确诊依据**：

- 健康人群中约4-8%携带HLA-B27且终生不发病。

- 阳性者患AS的概率约为5-20%，仍需其他证据支持。

---

#### **四、医生暂不诊断的可能考量**

1. **鉴别诊断未完成**：

- 排查类风湿关节炎（RF/抗CCP抗体）、银屑病关节炎（皮肤/指甲病变）、感染性关节炎（细菌培养）等。

2. **影像学证据不足**：

- 早期AS的骶髂关节炎仅MRI可检出（骨髓水肿），若未进行MRI或结果不明确，医生会谨慎诊断。

3. **病程时间较短**：

- AS确诊常需症状持续≥3个月，并观察影像学动态变化（如3-6个月后复查）。

---

### **下一步行动建议**

1. **明确影像学状态**：

- 若未做骶髂关节MRI，立即预约（脂肪抑制序列敏感检测早期炎症）。

- 若已有MRI，请医生详细描述是否存在骨髓水肿、关节面侵蚀等AS特征。

2. **完善鉴别诊断**：

- 检查类风湿因子（RF）、抗CCP抗体、ANA谱（排除其他自身免疫病）。

- 若有肠道症状（腹泻、腹痛），行肠镜排查炎症性肠病。

3. **短期对症治疗+观察**：

- 试用NSAIDs（如塞来昔布）2-4周：若疼痛显著缓解，支持炎症性病因。

- 记录症状变化：疼痛是否向胸椎/颈椎蔓延，是否出现足跟痛、虹膜炎等。

4. **寻求第二意见**：

- 携带所有检查报告至三甲医院风湿免疫科专家处复核（尤其关注MRI读片差异）。

---

### **重要注意事项**

- **勿自行贴标签**：焦虑可能加重疼痛感知，AS确诊需严格标准。

- **动态随访**：即使当前未确诊，建议每6个月复查骶髂关节MRI及炎症指标。

- **早期干预**：若高度怀疑AS但证据不足，医生可能按“脊柱关节炎”给予预防性治疗（如NSAIDs）。

---

### **总结**

**HLA-B27阳性和症状符合AS只是诊断拼图的一部分，缺乏影像学证据或存在其他疾病可能性时，医生需保持谨慎**。您应积极配合完善检查（尤其是MRI），同时关注症状演变，及时与医生沟通调整评估策略。

23.强直性脊柱炎（AS）的预后因人而异，但大多数患者通过规范治疗和科学管理，可以**有效控制病情进展，避免严重残疾**。是否发展为残疾主要取决于以下因素，以及您如何应对：

---

### 一、关键影响因素

1. **早期诊断与治疗**

- 若在疾病早期（如出现炎症性腰背痛、关节僵硬时）及时确诊并开始治疗，可显著延缓骨骼融合和脊柱变形。

2. **治疗依从性**

- 坚持用药（如非甾体抗炎药、生物制剂等）可抑制炎症，减少关节损伤。

- 定期复查（血沉、C反应蛋白、影像学检查）帮助医生调整方案。

3. **日常自我管理**

- **规律锻炼**：游泳、瑜伽、拉伸运动可维持关节活动度，防止脊柱僵硬。

- **姿势保护**：避免久坐，睡觉时选择硬板床+薄枕，保持脊柱生理曲度。

- **戒烟**：吸烟会加速病情进展，增加残疾风险。

4. **个体差异**

- 约10-20%患者可能发展为严重脊柱强直，但多与未规范治疗或合并其他并发症有关。

---

### 二、您可以采取的行动

1. **医疗层面**

- 与风湿免疫科医生保持长期随访，必要时联合康复科制定运动计划。

- 若传统药物效果不佳，可考虑生物制剂（如TNF-α抑制剂），研究显示其可显著延缓结构损伤。

2. **生活方式干预**

- **每日晨僵管理**：晨起热水浴或热敷，轻柔活动后再下床。

- **强化核心肌群**：平板支撑、桥式运动等增强腰部肌肉支撑力。

- **疼痛管理**：急性期可短期使用止痛药，避免长期依赖。

3. **心理与社会支持**

- 加入AS患者社群（如公益组织），获取经验分享与情感支持。

- 若出现焦虑/抑郁情绪，及时寻求心理咨询。

---

### 三、预后数据参考

- **研究显示**：规范治疗下，超过80%的患者能维持正常工作生活能力，仅有少数晚期患者可能出现脊柱强直或髋关节受累需置换手术。

---

**请记住**：AS是“可控制”的慢性病，而非“必然致残”的绝症。您的行动力直接决定疾病走向——从今天开始坚持治疗计划，未来生活依然可以充满可能性。

若需要更具体的用药或锻炼建议，请告知您的当前症状和用药情况，我会进一步为您分析。

24.强直性脊柱炎（AS）的药物治疗时长因人而异，主要取决于**病情活动度、药物反应及治疗目标**。以下是不同阶段和用药类别的分析框架，供您参考：

---

### 一、药物治疗的核心原则

1. **目标导向**

- **短期目标**：快速缓解疼痛、晨僵和炎症（通常需数周至数月）。

- **长期目标**：抑制疾病进展、预防关节强直和畸形（可能需要数年甚至终身用药）。

2. **分层治疗**

- **非甾体抗炎药（NSAIDs）**：控制症状和炎症的一线药物。

- **生物制剂（如TNF-α/IL-17抑制剂）**：中重度活动期或对NSAIDs无效时的核心治疗。

- **传统抗风湿药（如柳氮磺吡啶）**：对外周关节炎可能有效，但对中轴病变效果有限。

---

### 二、不同药物的用药时长建议

| **药物类型** | **用药时间范围** | **停药可能性** |

|-----------------------|---------------------------|--------------------------------------------------------------------------------|

| **非甾体抗炎药** | 数月至数年 | 病情稳定后可能减量或间歇使用（如仅在疼痛时服用），但需警惕停药后复发风险。 |

| **生物制剂** | 长期（通常≥2年） | 部分患者在持续缓解（≥6个月无活动）后可尝试逐步减量，但需密切监测复发迹象。 |

| **柳氮磺吡啶** | 6个月至数年 | 外周关节症状缓解后可考虑减停，但对中轴病变无效，不作为长期核心用药。 |

---

### 三、何时可能调整或停药？

1. **病情持续缓解**

- 若症状（疼痛、晨僵）消失、炎症指标（血沉、CRP）正常、影像学无进展，可尝试逐步减药。

- **注意**：AS易复发，减药需在医生指导下缓慢进行（如生物制剂从每2周1次延长至每4周1次）。

2. **药物副作用或禁忌**

- 如出现严重感染（生物制剂相关）、消化道出血（NSAIDs相关）等，需暂停或换药。

3. **特殊人群**

- 备孕/妊娠期：需调整用药（如停用甲氨蝶呤、部分生物制剂）。

---

### 四、关键注意事项

1. **避免自行停药**

- 即使症状缓解，炎症可能仍在 silently progressing（静默进展），需通过影像学（MRI/X线）评估。

2. **减药后的替代管理**

- 强化物理治疗（如每日脊柱伸展运动）、控制体重、戒烟等，减少复发风险。

3. **定期复查**

- 每3-6个月评估炎症指标和关节功能，每年1次影像学检查（如骶髂关节MRI）。

---

### 五、现实案例参考

- **案例1**：早期AS患者，规律使用NSAIDs+生物制剂2年后病情稳定，减为最低剂量维持，5年未出现脊柱强直。

- **案例2**：晚期AS患者因未规范用药，10年内进展为脊柱竹节样变，需长期依赖生物制剂控制疼痛。

---

**总结**：AS的用药时长无法一概而论，部分患者可能需要终身间歇性或低剂量维持治疗，但通过早期干预和规范管理，许多患者可显著减少药物依赖。建议与风湿科医生共同制定个体化方案，动态调整治疗策略。

25.在强直性脊柱炎（AS）治疗期间能否接种疫苗，需根据**药物类型、疫苗种类及病情活动度**综合判断。以下是具体分析框架：

---

### 一、基本原则

1. **优先接种灭活疫苗**（安全性高）

2. **慎用减毒活疫苗**（免疫抑制者禁用）

3. **接种时机**：建议在病情稳定期接种，避开疾病活动期或药物调整期。

---

### 二、不同药物对疫苗接种的影响

| **药物类型** | **可接种疫苗类型** | **注意事项** |

|-------------------------|-----------------------------------|------------------------------------------------------------------------------|

| **非甾体抗炎药（NSAIDs）** | 所有疫苗（包括灭活疫苗和减毒活疫苗） | 不影响疫苗效果，无需调整用药。 |

| **生物制剂（TNF-α/IL-17抑制剂等）** | ✔️ **灭活疫苗**（如流感疫苗、新冠疫苗、乙肝疫苗等）<br/> ❌ **减毒活疫苗**（如麻疹、水痘、带状疱疹活疫苗） | - 疫苗效果可能减弱（建议接种后检测抗体）<br/> - 接种时间：生物制剂注射前1-2周或用药间隔期。 |

| **糖皮质激素（如泼尼松）** | ✔️ 灭活疫苗<br/> ❌ 减毒活疫苗（若剂量≥20mg/日且持续≥2周） | 大剂量激素会抑制免疫，需咨询医生调整接种时间。 |

| **传统抗风湿药（如甲氨蝶呤、柳氮磺吡啶）** | ✔️ 灭活疫苗<br/> ❌ 减毒活疫苗 | 甲氨蝶呤可能降低疫苗效果，接种前可考虑暂停1-2周（需医生评估）。 |

---

### 三、重点疫苗推荐与禁忌

1. **流感疫苗**

- **推荐**：每年接种灭活流感疫苗（针剂），鼻喷流感疫苗（减毒活疫苗）禁用。

2. **新冠疫苗**

- **推荐**：灭活疫苗（如科兴、国药）或重组蛋白疫苗（如智飞）安全；mRNA疫苗也可考虑。

3. **肺炎球菌疫苗**

- **推荐**：13价/23价多糖疫苗（灭活），尤其适合长期用生物制剂者。

4. **带状疱疹疫苗**

- **禁忌**：活疫苗（Zostavax）禁用；重组亚单位疫苗（Shingrix）安全且推荐（需接种2剂）。

---

### 四、接种后注意事项

1. **观察不良反应**

- 常见局部红肿、低热等，一般1-3天缓解；若出现持续高热或AS症状加重，及时就医。

2. **抗体检测**

- 接种后1-2月可检测特异性抗体（如乙肝表面抗体、新冠中和抗体），评估是否需要补种。

3. **药物调整**

- 接种后无需停用AS治疗药物，除非医生特别建议。

---

### 五、特殊场景处理

1. **计划使用生物制剂前**

- 建议提前2-4周完成必要疫苗接种（如乙肝、肺炎球菌疫苗）。

2. **接触传染病后**

- 若暴露于麻疹/水痘等，禁用减毒活疫苗，需注射免疫球蛋白（如已用生物制剂）。

---

### 总结建议

1. **必打疫苗**：流感、新冠、肺炎球菌疫苗（灭活型）。

2. **避免疫苗**：卡介苗、口服脊髓灰质炎疫苗、麻疹/风疹/腮腺炎（MMR）活疫苗等。

3. **个体化方案**：接种前与风湿科医生沟通当前用药和病情，制定接种计划。

**注意**：目前研究显示，规范接种灭活疫苗不会显著增加AS复发风险，反而可降低感染（如流感）诱发的病情波动。

26.以下是关于“抗炎药”、“消炎药”和“止疼药”的区别的详细解释：

---

### **一、核心区别总结**

| **类别** | **作用目标** | **常见药物** | **适用场景** | **注意事项** |

|---------------|----------------------------|-----------------------------|-----------------------------|-----------------------------|

| **抗炎药** | 抑制炎症反应（非感染性） | 非甾体抗炎药（NSAIDs）、糖皮质激素 | 关节炎、强直性脊柱炎等自身免疫病 | 长期使用需监测胃肠道、肝肾等副作用 |

| **消炎药** | 中文常误指抗生素（实际应为抗炎药） | 抗生素（如青霉素、头孢） | **细菌感染**（如扁桃体炎、肺炎） | 仅针对细菌感染，滥用易导致耐药性 |

| **止疼药** | 单纯缓解疼痛（不抗炎） | 对乙酰氨基酚、阿片类（如吗啡） | 头痛、术后疼痛等非炎症性疼痛 | 部分药物有成瘾风险，需严格遵医嘱 |

---

### **二、具体分类与作用机制**

#### 1. **抗炎药（Anti-inflammatory Drugs）**

- **定义**：直接抑制炎症反应（红肿热痛）的药物，针对非感染性炎症。

- **分类**：

- **非甾体抗炎药（NSAIDs）**：

- **代表药物**：布洛芬、双氯芬酸、塞来昔布。

- **作用**：抑制COX酶，减少前列腺素合成（降低炎症和疼痛）。

- **适用**：强直性脊柱炎、类风湿关节炎的疼痛和炎症控制。

- **糖皮质激素**：

- **代表药物**：泼尼松、地塞米松。

- **作用**：强效抗炎和免疫抑制，但长期用副作用大（骨质疏松、血糖升高等）。

- **适用**：急性炎症爆发期或难治性AS短期控制。

#### 2. **消炎药（中文误称，实为抗生素）**

- **定义**：中文口语中常将“消炎药”错误等同于抗生素，实际抗生素仅针对细菌感染，对非感染性炎症无效。

- **常见药物**：青霉素、头孢菌素、阿奇霉素等。

- **作用**：杀灭或抑制细菌，消除感染源。

- **误区警示**：

- **强直性脊柱炎的炎症是无菌性炎症**，抗生素治疗无效（除非合并感染）。

- 滥用抗生素会引发耐药性，需严格区分感染与非感染性炎症。

#### 3. **止疼药（镇痛药）**

- **定义**：单纯缓解疼痛，不针对炎症本身。

- **分类**：

- **非阿片类**：

- **对乙酰氨基酚**：仅有解热镇痛作用，无抗炎效果。

- **适用**：轻度疼痛（头痛、牙痛），适合不能耐受NSAIDs的患者。

- **阿片类**：

- **代表药物**：曲马多、吗啡。

- **作用**：作用于中枢神经，强效镇痛。

- **适用**：中重度疼痛（如晚期AS严重骨痛），但需警惕成瘾性和呼吸抑制风险。

---

### **三、强直性脊柱炎（AS）中的药物选择**

- **首选抗炎药**：NSAIDs（如塞来昔布）是AS的一线药物，既能抗炎又能止痛。

- **止疼药的补充角色**：

- 若NSAIDs效果不足，可短期联用对乙酰氨基酚。

- 阿片类药物仅在难治性剧痛时谨慎使用（需避免长期依赖）。

- **禁用误区**：

- AS的疼痛源于炎症，单纯用“止疼药”（如对乙酰氨基酚）可能掩盖炎症进展，需以抗炎药为基础治疗。

---

### **四、常见问题解答**

#### 1. **“布洛芬既是抗炎药又是止疼药？”**

- 是的！NSAIDs类药物（如布洛芬）具有双重作用：抗炎 + 镇痛，但本质属于抗炎药。

#### 2. **“医生开抗生素治疗AS，是否合理？”**

- **不合理**！除非合并细菌感染（如尿路感染），否则抗生素对AS无效。

#### 3. **“长期吃抗炎药会上瘾吗？”**

- NSAIDs和激素不会成瘾，但需注意副作用（如胃溃疡、肾损伤）。阿片类止疼药可能成瘾，需严格遵医嘱。

---

### **五、用药建议**

1. **AS患者优先选择NSAIDs**：控制炎症是核心，止痛是附加效果。

2. **避免滥用抗生素**：无菌性炎症无需“消炎药”（抗生素）。

3. **止疼药谨慎使用**：仅在抗炎药效果不足时短期联用。

**关键原则**：

- 所有药物需在风湿科医生指导下使用，定期复查调整方案！

若有更多疑问，可提供当前用药情况，为您进一步分析。

27.---

### **核心建议总结**

在服用止疼药后仍感疼痛时，**切勿自行随意增加剂量**！是否加量需根据以下因素综合判断：

---

### **一、关键分析步骤**

1. **明确药物类型与剂量限制**

| **药物类型** | **单次剂量上限** | **每日最大剂量** | **用药间隔** |

|---------------------|------------------------|------------------------|--------------------|

| **对乙酰氨基酚** | ≤1000mg/次 | ≤4000mg/天 | ≥4-6小时 |

| **布洛芬** | 400-800mg/次 | ≤3200mg/天 | ≥6-8小时 |

| **阿片类药物** | 严格遵医嘱（如曲马多） | 个体化方案 | 按处方要求 |

- **注意**：若药物为复方制剂（如某些感冒药含对乙酰氨基酚），需合并计算总量，避免超量。

2. **检查是否规范用药**

- 是否按说明书剂量服用？

- 是否未到下次服药时间就急于加量？

- 是否空腹服药（部分药物需饭后服用以减少刺激）？

3. **区分疼痛性质**

- **炎症性疼痛**（如AS急性发作）：需联合抗炎药（如NSAIDs）而非单纯依赖止疼药。

- **神经性或机械性疼痛**：可能需要调整治疗方案（如加用神经调节药物或物理治疗）。

---

### **二、不同场景下的处理方案**

#### **场景1：服用的是非处方药（如对乙酰氨基酚/布洛芬）**

- **若未超剂量且间隔足够**：

- 可尝试辅助措施（如热敷、拉伸、休息），等待药物起效（通常需1-2小时）。

- **仍无效**：联系医生，考虑换用更强效药物（如处方级NSAIDs或短期联用其他镇痛药）。

- **若已达最大剂量**：

- **立即停用**，避免肝损伤（对乙酰氨基酚）或胃出血（布洛芬）风险。

- 改用其他缓解方式（如冷敷、理疗），并尽快就医。

#### **场景2：服用的是阿片类药物（如曲马多、羟考酮）**

- **严格遵循处方剂量**，不可自行加量（有成瘾和呼吸抑制风险）。

- 若疼痛未缓解，需联系医生调整方案（如增加剂量、换药或联合非药物治疗）。

#### **场景3：疼痛性质不明或持续加重**

- **立即就医**：可能是疾病进展、感染或其他并发症（如骨折、内脏问题）。

- 需排查：发热、局部红肿、活动受限加重等危险信号。

---

### **三、替代疼痛管理策略**

1. **非药物干预**

- **物理治疗**：热敷/冷敷、电疗、超声波治疗。

- **运动康复**：温和的脊柱伸展运动（如瑜伽、游泳）。

- **心理调节**：冥想、深呼吸缓解焦虑导致的疼痛感知增强。

2. **药物联用方案**

- 在医生指导下，抗炎药（如塞来昔布）与弱阿片类（如曲马多）短期联用。

- 避免同时使用两种NSAIDs（增加副作用风险）。

---

### **四、风险警示**

1. **对乙酰氨基酚过量**：

- ＞4000mg/天可导致急性肝衰竭，表现为恶心、黄疸、意识模糊。

2. **NSAIDs过量**：

- 引发胃溃疡、肾损伤，长期滥用增加心血管风险。

3. **阿片类风险**：

- 呼吸抑制、便秘、成瘾性，需严格监管使用。

---

### **五、何时必须就医？**

- 疼痛持续＞72小时且药物无效。

- 出现新症状（如麻木、无力、发热）。

- 怀疑药物副作用（如呕血、黑便、皮疹）。

---

### **总结**

**“仍疼就加药”是危险行为！** 正确做法：

1. 核对当前用药是否规范。

2. 联合非药物措施缓解疼痛。

3. 联系医生调整方案（如升级抗炎治疗或联合用药）。

**注**：强直性脊柱炎的疼痛管理应以控制炎症为核心（如坚持使用NSAIDs或生物制剂），而非依赖单纯止疼药掩盖症状。

28.---

### **核心结论**

服药后尿液变黄可能是正常现象，也可能是药物副作用或身体异常信号，需结合具体药物和伴随症状判断。以下是详细分析：

---

### **一、可能原因分类**

#### 1. **药物代谢产物（通常无害）**

- **柳氮磺吡啶**（治疗AS外周关节的药物）：

- 代谢产物（磺胺吡啶）经肾脏排泄，**使尿液呈深黄色或橙色**，属正常现象。

- **处理**：无需停药，但需多喝水减少尿液浓度。

- **维生素B群（尤其是B2）**：

- 服用复合维生素或B族补充剂后，尿液可呈亮黄色。

- **其他药物**：

- 利福平（抗结核药）、大黄类药物（泻药）等也可能导致尿黄。

#### 2. **脱水或饮食因素**

- **饮水不足**：尿液浓缩导致颜色加深（深黄色）。

- **食物影响**：胡萝卜、甜菜根、火龙果等含天然色素的食物可能暂时改变尿色。

#### 3. **需警惕的异常情况**

- **肝脏问题**：

- 胆红素升高（如肝炎、药物性肝损伤）会导致尿液呈**深茶色或可乐色**，常伴乏力、皮肤发黄（黄疸）。

- **溶血或肌肉损伤**：

- 血红蛋白尿（酱油色尿）或肌红蛋白尿（剧烈运动后），需紧急就医。

- **尿路感染**：

- 尿液浑浊发黄，伴尿频、尿痛或发热。

---

### **二、强直性脊柱炎（AS）常用药物与尿液颜色关联**

| **药物类型** | **是否可能导致尿黄** | **风险提示** |

|-----------------------|----------------------|-------------------------------------------|

| **非甾体抗炎药（NSAIDs）** | 罕见 | 长期大量用可能致肾损伤（尿色加深伴泡沫尿）。 |

| **柳氮磺吡啶** | 常见 | 明确导致尿黄，多喝水即可。 |

| **生物制剂** | 极罕见 | 通常不影响尿液颜色。 |

| **糖皮质激素** | 无直接关联 | 长期用需监测血糖、电解质。 |

---

### **三、自我排查与应对步骤**

1. **核对当前用药**

- 若正在服用**柳氮磺吡啶或复合维生素**，尿黄很可能是正常现象。

- 若无上述药物，需排查是否为新药或近期调整剂量。

2. **调整饮水与观察**

- **增加饮水量**至每日2000ml以上，观察尿液是否变浅（脱水导致的尿黄会在补水后缓解）。

- 暂停可疑食物（如维生素B补充剂）1-2天，观察尿色变化。

3. **危险信号识别（需立即就医）**

- 尿液呈**深茶色/可乐色** + 皮肤发黄、恶心（提示肝损伤）。

- 尿液**浑浊有异味** + 尿痛/发热（提示感染）。

- 尿色异常持续＞3天且补水无效。

---

### **四、就医检查建议**

1. **基础检查**：尿常规（判断有无红细胞、白细胞、胆红素）、肝功能（ALT、AST、胆红素）。

2. **特殊检查**（必要时）：腹部超声（排查肝胆疾病）、药物血药浓度监测。

---

### **五、处理方案**

- **正常药物代谢**：无需干预，保持充足水分。

- **疑似肝损伤**：立即停药并就医，检查肝功能。

- **尿路感染**：抗生素治疗（需医生开具处方）。

---

### **总结建议**

1. 若正在使用**柳氮磺吡啶**或**维生素B**，尿黄多为正常现象，多喝水即可。

2. 出现**异常尿色+其他症状**（如黄疸、发热）需立即就医。

3. 记录用药、饮食和尿色变化，就诊时提供详细信息以帮助医生判断。

**注意**：切勿因尿黄自行停药，尤其是AS治疗药物（如柳氮磺吡啶），需医生评估后决定是否调整方案。

29.---

### **生物制剂（Biologics）的定义**

生物制剂是一类通过**生物工程技术**制造的药物，靶向抑制导致强直性脊柱炎（AS）的关键炎症因子（如TNF-α、IL-17等）。与传统化学药物不同，生物制剂直接作用于免疫系统的特定环节，从而更精准地控制炎症，延缓疾病进展。

---

### **一、生物制剂的核心特点**

1. **靶向性强**

- 针对AS炎症通路中的特定分子（如TNF-α、IL-17），减少全身免疫抑制的副作用。

2. **疗效显著**

- 研究显示，约60-70%的AS患者使用生物制剂后，疼痛、晨僵和活动受限明显改善。

3. **需注射给药**

- 多为皮下注射或静脉输注（如依那西普、阿达木单抗需皮下注射，英夫利昔单抗需静脉输注）。

---

### **二、强直性脊柱炎常用生物制剂类型**

| **类型** | **代表药物** | **作用靶点** | **适用情况** |

|---------------------|-------------------|---------------|---------------------------------|

| **TNF-α抑制剂** | 阿达木单抗（修美乐）、依那西普（恩利）、英夫利昔单抗（类克） | TNF-α | 中重度AS，尤其伴髋关节受累或脊柱活动受限 |

| **IL-17抑制剂** | 司库奇尤单抗（可善挺）、依奇珠单抗（拓咨） | IL-17A | TNF-α抑制剂无效或不耐受者，合并银屑病 |

| **IL-12/23抑制剂** | 乌司奴单抗（喜达诺） | IL-12/IL-23 | 目前AS适应症较少，更多用于银屑病 |

---

### **三、生物制剂的作用机制**

以**TNF-α抑制剂**为例：

1. **阻断炎症信号**：TNF-α是AS炎症的核心介质，生物制剂结合并中和TNF-α，抑制下游炎症反应。

2. **延缓结构损伤**：减少骨破坏和韧带骨化，降低脊柱强直风险。

3. **快速起效**：部分患者用药2-4周后症状显著缓解。

---

### **四、哪些患者建议使用生物制剂？**

1. **中重度活动期AS**：

- 常规治疗（NSAIDs）无效或无法耐受。

- 炎症指标（CRP、血沉）持续升高，影像学显示进展。

2. **伴髋关节受累**：

- 髋关节破坏可能致残，需优先使用生物制剂保护关节功能。

3. **合并其他疾病**：

- 如银屑病、炎症性肠病（克罗恩病/溃疡性结肠炎），可选用兼顾多病的生物制剂（如抗TNF-α药物）。

---

### **五、生物制剂的优缺点**

| **优点** | **缺点** |

|----------------------------------|----------------------------------|

| 快速缓解症状（数周内起效） | 费用较高（年费用约3-15万元） |

| 显著延缓脊柱强直和关节破坏 | 需长期用药（停药易复发） |

| 靶向治疗，副作用相对较少 | 感染风险增加（如结核、真菌感染） |

---

### **六、使用生物制剂的注意事项**

1. **用药前筛查**：

- 结核筛查（T-SPOT/PPD试验）、乙肝/HIV检测，排除潜伏感染。

2. **定期监测**：

- 每3-6个月复查炎症指标、肝肾功能；每年1次胸片或骶髂关节MRI。

3. **禁忌与慎用**：

- 活动性感染、恶性肿瘤、心力衰竭患者禁用。

4. **生活方式配合**：

- 戒烟（吸烟降低疗效）、规律锻炼（维持关节活动度）。

---

### **七、常见问题解答**

1. **“生物制剂能治愈AS吗？”**

- 不能根治，但可长期控制炎症，部分患者可实现临床缓解（无症状）。

2. **“需要终身打针吗？”**

- 多数患者需长期维持治疗，少数病情稳定者可尝试减量或延长注射间隔。

3. **“国产和进口生物制剂哪个好？”**

- 国产生物类似药（如格乐立、安健宁）疗效与进口药相当，价格更低，可优先考虑。

---

### **总结**

生物制剂是AS治疗的“精准武器”，尤其适合传统治疗无效的中重度患者。其使用需严格评估感染风险、经济条件和疾病活动度，并在风湿科医生指导下制定个体化方案。**关键原则**：早用、规范用、长期管理！

30.---

### **生物制剂的副作用风险分析**

生物制剂在强直性脊柱炎（AS）治疗中疗效显著，但其副作用需科学管理。总体而言，**副作用风险可控**，但需重点关注感染和免疫相关反应。以下是具体分类与应对策略：

---

### **一、副作用风险分级**

#### **1. 常见轻度反应（发生率＞10%）**

| **类型** | **表现** | **处理建议** |

|-------------------|-----------------------------|------------------------------|

| **注射部位反应** | 红肿、瘙痒、硬结 | 冷敷，更换注射部位，通常1-3天消退。 |

| **流感样症状** | 低热、头痛、肌肉酸痛 | 多休息、补水，必要时用对乙酰氨基酚。 |

| **消化道不适** | 恶心、腹泻 | 随餐服药，症状持续需就医。 |

#### **2. 中重度风险（发生率1-10%）**

| **类型** | **表现** | **高危人群** | **处理建议** |

|-------------------|-----------------------------|------------------------------|------------------------------|

| **感染风险增加** | 呼吸道感染、皮肤感染、结核复发 | 未规范筛查结核/乙肝者 | 用药前必须筛查潜伏感染，出现发热/咳嗽及时就医。 |

| **过敏反应** | 皮疹、荨麻疹、呼吸困难 | 过敏体质者 | 立即停药并急诊处理。 |

| **肝酶升高** | 无症状性ALT/AST升高 | 合并脂肪肝或乙肝携带者 | 定期监测肝功能，必要时保肝治疗。 |

#### **3. 罕见但严重风险（＜1%）**

| **类型** | **表现** | **高危药物** | **处理建议** |

|-------------------|-----------------------------|------------------------------|------------------------------|

| **脱髓鞘疾病** | 视力模糊、肢体麻木 | TNF-α抑制剂 | 立即停药，神经科会诊。 |

| **心力衰竭加重** | 气促、水肿 | 既往心功能不全者 | 禁用TNF-α抑制剂，换用IL-17抑制剂。 |

| **恶性肿瘤** | 淋巴瘤、皮肤癌风险略增 | 长期用药者（证据尚不明确） | 定期体检，避免过度担忧。 |

---

### **二、不同生物制剂的副作用差异**

| **药物类型** | **主要风险** | **相对优势** |

|--------------------|------------------------------|------------------------------|

| **TNF-α抑制剂** | 感染、结核复发、脱髓鞘反应 | 对中轴症状和髋关节保护更佳 |

| **IL-17抑制剂** | 念珠菌感染、加重炎症性肠病 | 感染风险略低于TNF抑制剂 |

| **JAK抑制剂** | 血栓、血脂升高 | 口服方便，无需注射 |

---

### **三、副作用预防与管理策略**

1. **用药前严格筛查**

- **必查项目**：结核（T-SPOT/PPD）、乙肝五项、HIV、胸部CT。

- **禁忌症**：活动性感染、恶性肿瘤、重度心衰。

2. **用药期间监测**

- **每3个月**：血常规、肝肾功能、炎症指标（CRP/ESR）。

- **每年**：胸片、乙肝病毒DNA（携带者）、皮肤检查（防癌）。

3. **感染防控**

- **日常防护**：避免接触传染源，流感季节接种灭活疫苗。

- **出现感染**：暂停生物制剂直至感染治愈，必要时用抗生素。

4. **特殊人群调整**

- **结核潜伏感染者**：先抗结核治疗3-4周，再启动生物制剂。

- **乙肝携带者**：全程抗病毒治疗（如恩替卡韦）。

---

### **四、患者常见疑问解答**

#### **1. “生物制剂副作用比传统药物大吗？”**

- **不一定**！生物制剂靶向性强，肝肾毒性小于传统药物（如甲氨蝶呤），但感染风险略高。总体安全性可控。

#### **2. “出现副作用必须停药吗？”**

- **分情况**：

- 轻度注射反应：可继续用药，对症处理。

- 严重感染或过敏：立即停药并就医。

#### **3. “如何降低副作用风险？”**

- 规范筛查、定期复查、保持良好卫生习惯（如勤洗手、避免生食）。

---

### **五、总结建议**

1. **理性看待副作用**：生物制剂的风险远低于AS uncontrolled（未控制疾病）导致的残疾，切勿因恐惧副作用延误治疗。

2. **医患协作管理**：用药期间与风湿科医生保持沟通，及时反馈异常症状。

3. **优先选择安全性更高的药物**：如IL-17抑制剂对结核风险较低，适合潜伏感染患者。

**关键原则**：

- 副作用可控，疗效获益明确，规范用药下绝大多数患者可安全使用生物制剂！

31.---

### **核心策略**

若因乙肝、结核或其他感染无法使用生物制剂，需根据感染类型和疾病活动度，选择**替代治疗方案+感染控制**的联合策略。以下是具体应对方案：

---

### **一、不同感染状态的处理原则**

| **感染类型** | **能否使用生物制剂** | **替代治疗路径** |

|---------------------|-------------------------------|---------------------------------------------------------------------------------|

| **活动性结核** | ❌ 禁用 | 1. **优先抗结核治疗**（HRZE方案6-9个月）<br>2. 结核治愈后评估是否启用生物制剂。 |

| **潜伏结核感染** | ⚠️ 预防性抗结核后可用 | 1. **异烟肼预防治疗**（9个月）<br>2. 治疗1月后可启动生物制剂（优选IL-17抑制剂）。 |

| **慢性乙肝** | ⚠️ 病毒活跃复制期禁用 | 1. **抗病毒治疗**（恩替卡韦/替诺福韦）控制HBV-DNA至阴性<br>2. 病情稳定后可谨慎使用TNF抑制剂，联用抗病毒药。 |

| **乙肝携带者** | ⚠️ 需全程抗病毒防护 | 同慢性乙肝处理，密切监测HBV-DNA和肝功能。 |

| **HIV感染** | ⚠️ 免疫功能极低下者（CD4＜200）禁用 | 1. 控制HIV至病毒载量检测不到、CD4＞200<br>2. 可考虑使用IL-17抑制剂（感染风险较低）。 |

| **其他活动性感染** | ❌ 感染控制前禁用 | 1. 针对性抗感染治疗（细菌/真菌/寄生虫）<br>2. 感染痊愈后重启生物制剂。 |

---

### **二、无法使用生物制剂时的替代治疗选择**

#### 1. **强化传统药物治疗**

- **NSAIDs（非甾体抗炎药）**：

- 如双氯芬酸、塞来昔布，需足量足疗程（4周以上评估疗效）。

- **注意**：长期使用需监测胃肠道/肾脏副作用。

- **柳氮磺吡啶**：

- 对外周关节炎有效，但对中轴症状（脊柱炎症）效果有限。

- **糖皮质激素**：

- 短期用于急性发作（如关节腔注射或口服泼尼松），避免长期全身使用。

#### 2. **新型小分子药物（JAK抑制剂）**

- **代表药物**：托法替布、乌帕替尼。

- **优势**：口服方便，对中轴症状有效（需评估感染风险）。

- **限制**：乙肝/结核患者需谨慎，可能增加血栓和感染风险。

#### 3. **物理治疗与生活方式干预**

- **规律锻炼**：游泳、瑜伽维持脊柱活动度，延缓强直。

- **物理疗法**：热疗、超声波缓解局部炎症。

- **戒烟限酒**：降低疾病进展风险。

---

### **三、分场景处理方案**

#### **场景1：合并活动性结核/乙肝，需延迟生物制剂治疗**

1. **优先控制感染**：

- 结核：HRZE四联抗结核治疗（异烟肼+利福平+吡嗪酰胺+乙胺丁醇）。

- 乙肝：恩替卡韦/替诺福韦抗病毒，每月监测HBV-DNA。

2. **过渡期AS治疗**：

- 使用NSAIDs+物理治疗控制症状，必要时短期激素。

#### **场景2：潜伏结核/乙肝携带状态**

1. **预防性治疗**：

- 结核：异烟肼300mg/日 ×9个月。

- 乙肝：恩替卡韦/替诺福韦全程防护。

2. **选择低风险生物制剂**：

- IL-17抑制剂（如司库奇尤单抗）对结核再激活风险低于TNF抑制剂。

#### **场景3：感染风险极高无法使用任何生物制剂**

1. **联合传统药物**：

- NSAIDs + 柳氮磺吡啶 + 局部激素注射。

2. **JAK抑制剂尝试**：

- 如托法替布（需排除乙肝/结核活动性感染）。

---

### **四、关键注意事项**

1. **多学科协作**：

- 感染科与风湿科联合制定方案，平衡感染控制与AS治疗。

2. **定期监测**：

- 结核：每3-6个月胸片或干扰素释放试验（IGRA）。

- 乙肝：每3个月HBV-DNA、肝功能。

3. **疫苗防护**：

- 感染控制后接种肺炎球菌、流感灭活疫苗，降低后续感染风险。

---

### **五、总结建议**

即使存在感染限制，AS仍有多种治疗选择：

1. **感染活动期**：以抗感染+传统药物过渡。

2. **感染控制后**：优选IL-17抑制剂或JAK抑制剂，联用抗病毒/抗结核防护。

3. **终身管理**：戒烟、锻炼、定期复查，最大限度保护关节功能。

**重要提示**：切勿因感染风险放弃治疗！通过规范管理，多数患者可实现疾病控制与感染防护的双重目标。

32.---

### **核心结论**

使用生物制剂后是否需联用其他药物，取决于**疾病活动度、合并症状及治疗目标**。多数情况下，生物制剂可作核心治疗，但需根据个体情况联用其他药物或非药物干预。以下是具体分析：

---

### **一、需联用其他药物的常见场景**

#### 1. **疾病活动度高（急性发作期）**

- **联用NSAIDs**（如塞来昔布）：

- 生物制剂起效需2-12周，初期联用NSAIDs可快速控制疼痛和炎症。

- **建议**：症状缓解后逐步减停NSAIDs，避免长期联用增加胃肠道/肾脏负担。

#### 2. **合并外周关节炎或附着点炎**

- **联用柳氮磺吡啶**：

- 生物制剂对中轴症状（脊柱）效果显著，但对部分患者外周关节炎症控制不足。

- **注意**：柳氮磺吡啶对中轴病变无效，仅针对外周症状。

#### 3. **难治性疼痛或并发症**

- **短期联用糖皮质激素**：

- 局部关节腔注射（如膝关节）或短期口服泼尼松（＜10mg/日），用于急性炎症爆发。

- **止疼药（对乙酰氨基酚）**：

- 仅在生物制剂未完全起效时临时使用，避免掩盖炎症进展。

#### 4. **合并其他自身免疫病**

- **联用免疫抑制剂**：

- 如合并银屑病用甲氨蝶呤，合并炎症性肠病用美沙拉嗪，需医生评估药物相互作用。

---

### **二、无需联用的情况**

1. **病情稳定期**：

- 生物制剂单药维持治疗（如阿达木单抗每2周1次），炎症指标（CRP/ESR）正常且无结构进展。

2. **无外周关节受累**：

- 单纯中轴型AS患者，生物制剂单药通常足够。

---

### **三、用药联用原则**

| **联用类型** | **推荐方案** | **避免组合** |

|---------------------|------------------------------|------------------------------|

| **生物制剂+NSAIDs** | 安全且常见，短期联用 | 长期联用增加副作用风险 |

| **生物制剂+激素** | 局部注射安全，全身激素慎用 | 避免长期大剂量激素联用 |

| **生物制剂+JAK抑制剂** | 尚无明确证据，可能增加感染风险 | 除非临床试验或医生特别建议 |

---

### **四、非药物干预的协同作用**

即使使用生物制剂，仍需坚持以下措施以优化疗效：

1. **规律锻炼**：

- 每日脊柱伸展（如猫牛式）、游泳等维持关节活动度。

2. **物理治疗**：

- 热敷、超声波治疗缓解局部僵硬。

3. **生活方式调整**：

- 戒烟（吸烟降低生物制剂疗效）、保持标准体重（减轻关节负荷）。

---

### **五、何时可停用其他药物？**

1. **症状完全缓解**：

- 若生物制剂单药控制良好（无疼痛、晨僵＜15分钟、CRP正常），可逐步停用NSAIDs。

2. **影像学稳定**：

- 骶髂关节或脊柱MRI显示无新发炎症或骨破坏，提示可简化治疗方案。

---

### **六、总结建议**

1. **初始治疗期**：生物制剂联用NSAIDs快速控炎，随后逐步减停NSAIDs。

2. **长期维持期**：生物制剂单药为主，根据症状叠加物理治疗或短期对症药物。

3. **定期评估**：每3-6个月复查炎症指标和影像学，动态调整用药方案。

**关键提示**：

- 生物制剂是AS治疗的“主力军”，但合理联用其他药物可弥补其局限性。

- 所有联用方案需经风湿科医生评估，切勿自行调整药物组合！

33.---

### **核心结论**

糖皮质激素（激素）在强直性脊柱炎（AS）治疗中**作用有限**，仅作为特定情况下的辅助治疗，而非核心方案。以下是具体分析：

---

### **一、激素在AS治疗中的定位**

#### 1. **适用场景**

- **外周关节炎或附着点炎急性发作**：

- 局部关节腔或肌腱端注射（如膝关节、跟腱炎），快速缓解炎症和疼痛。

- **中轴症状（脊柱）难治性疼痛**：

- 短期口服低剂量激素（如泼尼松≤10mg/日）过渡，待生物制剂/NSAIDs起效后逐渐停用。

- **合并虹膜炎/葡萄膜炎**：

- 局部眼用激素（滴眼液）联合全身治疗。

#### 2. **不推荐场景**

- **长期全身使用激素**：

- 中轴型AS对激素反应差，且副作用风险远大于获益。

- **作为一线治疗替代NSAIDs或生物制剂**：

- 激素无法延缓骨赘形成或脊柱强直，治标不治本。

---

### **二、激素的局限性及风险**

| **问题类型** | **具体风险** |

|------------------|-----------------------------------------------------------------------------|

| **疗效局限** | 对中轴炎症（骶髂关节、脊柱）效果微弱，无法改变疾病进展。 |

| **短期副作用** | 血糖升高、血压波动、失眠、消化道溃疡。 |

| **长期副作用** | 骨质疏松（AS患者本就骨代谢异常）、股骨头坏死、感染风险增加、库欣综合征。 |

| **停药反应** | 突然减停可能诱发肾上腺危象，需严格遵医嘱缓慢减量。 |

---

### **三、激素使用原则**

#### 1. **局部注射优先**

- **方法**：关节腔或肌腱端注射曲安奈德/得宝松，每年同一关节注射≤3次。

- **优势**：靶向性强，全身副作用小。

#### 2. **全身用药严格限制**

- **剂量**：泼尼松≤10mg/日，疗程＜3个月。

- **联用保护措施**：

- 补充钙剂+维生素D（防骨质疏松）。

- 胃黏膜保护剂（如奥美拉唑）预防消化道出血。

#### 3. **避免滥用激素**

- **禁忌**：无明确炎症证据的慢性疼痛、单纯腰背痛（非活动期AS）。

---

### **四、替代治疗方案（优于激素）**

| **治疗目标** | **优选方案** |

|--------------------|-----------------------------------------------------------------------------|

| **控制中轴炎症** | NSAIDs（如塞来昔布）或生物制剂（TNF-α/IL-17抑制剂）。 |

| **外周关节炎** | 柳氮磺吡啶、生物制剂联合NSAIDs。 |

| **急性疼痛管理** | 短期联用对乙酰氨基酚，而非依赖激素。 |

| **预防骨破坏** | 坚持抗炎治疗（生物制剂）+ 钙/维生素D + 负重运动。 |

---

### **五、患者常见误区解答**

#### 1. **“激素效果立竿见影，为什么医生不让我用？”**

- 激素虽能快速缓解症状，但无法阻止AS的核心病理进程（韧带骨化、脊柱强直），且长期副作用可能加重病情（如骨质疏松导致骨折风险）。

#### 2. **“听说打封闭针（激素）能治强直，靠谱吗？”**

- 仅针对局部关节/肌腱炎症有效，对脊柱病变无效，反复注射可能损伤软骨。

#### 3. **“用了激素后疼痛复发，能自己加量吗？”**

- 绝对禁止！擅自加量会导致副作用累积，应联系医生调整核心治疗方案（如升级生物制剂）。

---

### **六、总结建议**

1. **AS治疗核心**：以NSAIDs和生物制剂为主，激素仅作为“临时拐杖”。

2. **局部注射**：安全有效，但需严格限制频率和部位。

3. **全身激素**：仅在急性期短期过渡使用，避免长期依赖。

4. **副作用防控**：用药期间定期监测骨密度、血糖和血压。

**关键原则**：

- 激素不是AS的根治手段，规范抗炎治疗（抑制IL-17/TNF-α）才是延缓残疾的关键！

34.---

### **核心结论**

使用生物制剂后是否需联用其他药物，取决于**疾病活动度、合并症状及治疗目标**。多数情况下，生物制剂可作核心治疗，但需根据个体情况联用其他药物或非药物干预。以下是具体分析：

---

### **一、需联用其他药物的常见场景**

#### 1. **疾病活动度高（急性发作期）**

- **联用NSAIDs**（如塞来昔布）：

- 生物制剂起效需2-12周，初期联用NSAIDs可快速控制疼痛和炎症。

- **建议**：症状缓解后逐步减停NSAIDs，避免长期联用增加胃肠道/肾脏负担。

#### 2. **合并外周关节炎或附着点炎**

- **联用柳氮磺吡啶**：

- 生物制剂对中轴症状（脊柱）效果显著，但对部分患者外周关节炎症控制不足。

- **注意**：柳氮磺吡啶对中轴病变无效，仅针对外周症状。

#### 3. **难治性疼痛或并发症**

- **短期联用糖皮质激素**：

- 局部关节腔注射（如膝关节）或短期口服泼尼松（＜10mg/日），用于急性炎症爆发。

- **止疼药（对乙酰氨基酚）**：

- 仅在生物制剂未完全起效时临时使用，避免掩盖炎症进展。

#### 4. **合并其他自身免疫病**

- **联用免疫抑制剂**：

- 如合并银屑病用甲氨蝶呤，合并炎症性肠病用美沙拉嗪，需医生评估药物相互作用。

---

### **二、无需联用的情况**

1. **病情稳定期**：

- 生物制剂单药维持治疗（如阿达木单抗每2周1次），炎症指标（CRP/ESR）正常且无结构进展。

2. **无外周关节受累**：

- 单纯中轴型AS患者，生物制剂单药通常足够。

---

### **三、用药联用原则**

| **联用类型** | **推荐方案** | **避免组合** |

|---------------------|------------------------------|------------------------------|

| **生物制剂+NSAIDs** | 安全且常见，短期联用 | 长期联用增加副作用风险 |

| **生物制剂+激素** | 局部注射安全，全身激素慎用 | 避免长期大剂量激素联用 |

| **生物制剂+JAK抑制剂** | 尚无明确证据，可能增加感染风险 | 除非临床试验或医生特别建议 |

---

### **四、非药物干预的协同作用**

即使使用生物制剂，仍需坚持以下措施以优化疗效：

1. **规律锻炼**：

- 每日脊柱伸展（如猫牛式）、游泳等维持关节活动度。

2. **物理治疗**：

- 热敷、超声波治疗缓解局部僵硬。

3. **生活方式调整**：

- 戒烟（吸烟降低生物制剂疗效）、保持标准体重（减轻关节负荷）。

---

### **五、何时可停用其他药物？**

1. **症状完全缓解**：

- 若生物制剂单药控制良好（无疼痛、晨僵＜15分钟、CRP正常），可逐步停用NSAIDs。

2. **影像学稳定**：

- 骶髂关节或脊柱MRI显示无新发炎症或骨破坏，提示可简化治疗方案。

---

### **六、总结建议**

1. **初始治疗期**：生物制剂联用NSAIDs快速控炎，随后逐步减停NSAIDs。

2. **长期维持期**：生物制剂单药为主，根据症状叠加物理治疗或短期对症药物。

3. **定期评估**：每3-6个月复查炎症指标和影像学，动态调整用药方案。

**关键提示**：

- 生物制剂是AS治疗的“主力军”，但合理联用其他药物可弥补其局限性。

- 所有联用方案需经风湿科医生评估，切勿自行调整药物组合！

35.---

### **核心结论**

糖皮质激素（激素）在强直性脊柱炎（AS）治疗中**作用有限**，仅作为特定情况下的辅助治疗，而非核心方案。以下是具体分析：

---

### **一、激素在AS治疗中的定位**

#### 1. **适用场景**

- **外周关节炎或附着点炎急性发作**：

- 局部关节腔或肌腱端注射（如膝关节、跟腱炎），快速缓解炎症和疼痛。

- **中轴症状（脊柱）难治性疼痛**：

- 短期口服低剂量激素（如泼尼松≤10mg/日）过渡，待生物制剂/NSAIDs起效后逐渐停用。

- **合并虹膜炎/葡萄膜炎**：

- 局部眼用激素（滴眼液）联合全身治疗。

#### 2. **不推荐场景**

- **长期全身使用激素**：

- 中轴型AS对激素反应差，且副作用风险远大于获益。

- **作为一线治疗替代NSAIDs或生物制剂**：

- 激素无法延缓骨赘形成或脊柱强直，治标不治本。

---

### **二、激素的局限性及风险**

| **问题类型** | **具体风险** |

|------------------|-----------------------------------------------------------------------------|

| **疗效局限** | 对中轴炎症（骶髂关节、脊柱）效果微弱，无法改变疾病进展。 |

| **短期副作用** | 血糖升高、血压波动、失眠、消化道溃疡。 |

| **长期副作用** | 骨质疏松（AS患者本就骨代谢异常）、股骨头坏死、感染风险增加、库欣综合征。 |

| **停药反应** | 突然减停可能诱发肾上腺危象，需严格遵医嘱缓慢减量。 |

---

### **三、激素使用原则**

#### 1. **局部注射优先**

- **方法**：关节腔或肌腱端注射曲安奈德/得宝松，每年同一关节注射≤3次。

- **优势**：靶向性强，全身副作用小。

#### 2. **全身用药严格限制**

- **剂量**：泼尼松≤10mg/日，疗程＜3个月。

- **联用保护措施**：

- 补充钙剂+维生素D（防骨质疏松）。

- 胃黏膜保护剂（如奥美拉唑）预防消化道出血。

#### 3. **避免滥用激素**

- **禁忌**：无明确炎症证据的慢性疼痛、单纯腰背痛（非活动期AS）。

---

### **四、替代治疗方案（优于激素）**

| **治疗目标** | **优选方案** |

|--------------------|-----------------------------------------------------------------------------|

| **控制中轴炎症** | NSAIDs（如塞来昔布）或生物制剂（TNF-α/IL-17抑制剂）。 |

| **外周关节炎** | 柳氮磺吡啶、生物制剂联合NSAIDs。 |

| **急性疼痛管理** | 短期联用对乙酰氨基酚，而非依赖激素。 |

| **预防骨破坏** | 坚持抗炎治疗（生物制剂）+ 钙/维生素D + 负重运动。 |

---

### **五、患者常见误区解答**

#### 1. **“激素效果立竿见影，为什么医生不让我用？”**

- 激素虽能快速缓解症状，但无法阻止AS的核心病理进程（韧带骨化、脊柱强直），且长期副作用可能加重病情（如骨质疏松导致骨折风险）。

#### 2. **“听说打封闭针（激素）能治强直，靠谱吗？”**

- 仅针对局部关节/肌腱炎症有效，对脊柱病变无效，反复注射可能损伤软骨。

#### 3. **“用了激素后疼痛复发，能自己加量吗？”**

- 绝对禁止！擅自加量会导致副作用累积，应联系医生调整核心治疗方案（如升级生物制剂）。

---

### **六、总结建议**

1. **AS治疗核心**：以NSAIDs和生物制剂为主，激素仅作为“临时拐杖”。

2. **局部注射**：安全有效，但需严格限制频率和部位。

3. **全身激素**：仅在急性期短期过渡使用，避免长期依赖。

4. **副作用防控**：用药期间定期监测骨密度、血糖和血压。

**关键原则**：

- 激素不是AS的根治手段，规范抗炎治疗（抑制IL-17/TNF-α）才是延缓残疾的关键！

36.---

### **核心原则**

手术前后是否继续使用药物（包括生物制剂）需根据**手术类型、药物种类及疾病活动度**综合评估，以平衡**感染风险**与**病情控制需求**。以下是分阶段管理方案：

---

### **一、术前药物管理策略**

#### **1. 生物制剂**

| **药物类型** | **停药时间** | **依据** |

|--------------------|---------------------------|-------------------------------------------------------------------------|

| **TNF-α抑制剂** | 术前2-4周停用（如阿达木单抗半衰期约2周） | 降低术后感染风险（尤其是骨科/关节手术）。 |

| **IL-17抑制剂** | 术前1-2周停用（如司库奇尤单抗半衰期约4周） | IL-17抑制剂感染风险略低，可适当缩短停药时间。 |

| **JAK抑制剂** | 术前至少1周停用 | 增加血栓风险，需更早停药（具体遵医嘱）。 |

#### **2. 其他AS药物**

| **药物类型** | **处理建议** |

|--------------------|-----------------------------------------------------------------------------|

| **NSAIDs** | 术前5-7天停用（如布洛芬、塞来昔布），减少术中出血风险及肾损伤。 |

| **糖皮质激素** | 不可突然停药！需根据剂量调整：<br>- 长期使用者：术前调整剂量（如泼尼松≥5mg/日需静脉激素覆盖）。<br>- 短期使用者：逐步减量至术前停药。 |

| **止痛药** | 对乙酰氨基酚可继续使用（不影响凝血），阿片类药物按麻醉科建议调整。 |

---

### **二、术后恢复期用药管理**

#### **1. 重启生物制剂时机**

| **手术类型** | **建议恢复时间** | **注意事项** |

|--------------------|--------------------------|------------------------------------------|

| **清洁手术（无感染）** | 术后10-14天，伤口愈合良好且无感染迹象 | 优先选择IL-17抑制剂（感染风险更低）。 |

| **感染高风险手术** | 术后4-6周，确认无感染 | 需密切监测体温、伤口渗出及炎症指标（CRP）。 |

#### **2. 术后疼痛管理替代方案**

- **短期使用对乙酰氨基酚**：不干扰凝血，适合术后镇痛。

- **局部冷敷/理疗**：减轻肿胀和疼痛，减少药物依赖。

- **谨慎使用阿片类**：仅在剧痛时短期使用，避免成瘾。

---

### **三、分场景处理建议**

#### **场景1：择期手术（如关节置换）**

1. **术前1个月**：与风湿科医生评估病情，逐步停生物制剂。

2. **过渡期**：使用NSAIDs或低剂量激素控制症状（需术前1周停NSAIDs）。

3. **术后**：优先控制感染风险，延迟生物制剂重启至伤口愈合。

#### **场景2：急诊手术（如骨折/肠穿孔）**

1. **立即停用所有免疫抑制药物**（生物制剂、JAK抑制剂）。

2. **术后抗感染治疗**：广谱抗生素覆盖，延迟生物制剂至感染完全控制。

#### **场景3：微创手术（如内镜/牙科手术）**

- **低感染风险手术**：生物制剂可不停用，但需加强术后感染监测。

---

### **四、关键注意事项**

1. **多学科协作**：外科医生、麻醉科与风湿科共同制定用药计划。

2. **感染监控**：术后每日检查伤口，每周复查CRP/血常规，警惕迟发感染。

3. **病情反弹处理**：若停生物制剂后AS急性发作，可短期使用激素过渡（避免长期使用）。

---

### **五、总结建议**

1. **术前**：根据药物半衰期和手术类型提前停药，优先控制感染风险。

2. **术后**：确保伤口愈合后再逐步重启生物制剂，联用物理治疗减少药物依赖。

3. **个体化方案**：需结合手术紧迫性、AS活动度及患者整体状况动态调整。

**务必告知医生您正在使用的所有药物**，以确保手术安全和术后恢复顺利！

37.---

### **核心结论**

强直性脊柱炎（AS）治疗药物与感冒药的联用需谨慎，部分组合可能导致**副作用叠加或相互作用**，需根据具体药物成分调整。以下是分类解析与安全用药建议：

---

### **一、强直常用药与感冒药的潜在风险**

#### **1. 非甾体抗炎药（NSAIDs） + 感冒药**

| **感冒药成分** | **风险** | **建议** |

|----------------------|-------------------------------------------------------------------------|-------------------------------------------------------------------------|

| **对乙酰氨基酚** | 与NSAIDs（如布洛芬）联用增加肝肾负担，长期可能致肝损伤或胃出血。 | 短期联用需间隔4-6小时，总剂量控制（对乙酰氨基酚≤3000mg/天）。 |

| **其他NSAIDs（如双氯芬酸）** | 感冒药含NSAIDs（如某些复方感冒片），联用导致重复用药，加重副作用。 | 仔细阅读说明书，避免重复成分，必要时单用对乙酰氨基酚。 |

| **伪麻黄碱（鼻减充血剂）** | NSAIDs可能升高血压，联用伪麻黄碱加重心血管负担（尤其高血压患者）。 | 优先选择不含伪麻黄碱的感冒药（如氯雷他定单方抗组胺药）。 |

#### **2. 生物制剂（如TNF-α抑制剂） + 感冒药**

| **风险类型** | **说明** | **建议** |

|----------------------|-------------------------------------------------------------------------|-------------------------------------------------------------------------|

| **免疫抑制叠加** | 生物制剂降低免疫力，感冒药不直接冲突，但若感冒合并细菌感染需抗生素治疗时，感染风险增加。 | 感冒期间密切监测体温，若持续发热或咳黄痰，及时就医排查感染。 |

| **肝酶升高** | 部分感冒药（如对乙酰氨基酚）与生物制剂联用可能增加肝损伤风险。 | 定期查肝功能（ALT/AST），感冒期间避免饮酒。 |

#### **3. 柳氮磺吡啶/甲氨蝶呤 + 感冒药**

| **风险** | **处理建议** |

|----------------------|-------------------------------------------------------------------------|

| **骨髓抑制风险** | 免疫抑制剂（如甲氨蝶呤）与感冒药联用可能加重白细胞减少，增加感染风险。 | 感冒期间监测血常规，若白细胞下降明显，暂停甲氨蝶呤。 |

| **磺胺类药物过敏** | 柳氮磺吡啶含磺胺基团，避免联用含磺胺的感冒药（罕见）。 | 选择成分单一的感冒药，如对乙酰氨基酚+右美沙芬。 |

---

### **二、安全联用方案**

#### **1. 优选感冒药类型**

- **单纯解热镇痛**：对乙酰氨基酚（不与NSAIDs联用时）。

- **抗组胺药**：氯雷他定、西替利嗪（无伪麻黄碱）。

- **止咳药**：右美沙芬（中枢性镇咳，无成瘾性）。

#### **2. 避免的感冒药成分**

- **含NSAIDs的复方制剂**：如阿司匹林、布洛芬复方感冒片。

- **含伪麻黄碱的复方药**：新康泰克、泰诺（伪麻黄碱可能升高血压）。

- **含可待因的止咳药**：与免疫抑制剂联用可能加重便秘或呼吸抑制。

---

### **三、分场景处理建议**

#### **场景1：仅需退热/止痛**

- **方案**：对乙酰氨基酚（≤3000mg/天）单用，暂停AS的NSAIDs（如布洛芬）。

- **注意**：退热后48小时无反复，可恢复原AS用药。

#### **场景2：鼻塞/流涕为主**

- **方案**：生理盐水冲洗鼻腔 + 氯雷他定（抗组胺药），避免伪麻黄碱。

#### **场景3：咳嗽/痰多**

- **方案**：右美沙芬（干咳）或乙酰半胱氨酸（祛痰），避免可待因。

---

### **四、用药期间监测与应急处理**

1. **监测指标**：

- 肝功能（尤其联用对乙酰氨基酚或生物制剂）。

- 血常规（使用免疫抑制剂者）。

2. **危险信号**：

- 皮肤黄染（肝损伤）、呕血/黑便（消化道出血）、喘息/皮疹（过敏反应）→ 立即停药并急诊。

---

### **五、总结建议**

1. **感冒药选择原则**：成分单一、不含NSAIDs或伪麻黄碱，避免与AS治疗药副作用叠加。

2. **优先非药物缓解**：多喝水、休息、物理降温（冰敷）减少药物依赖。

3. **咨询医生/药师**：告知正在使用的AS药物，获取个性化方案（如调整剂量或用药间隔）。

**关键提示**：

- 若AS处于活动期（如血沉/CRP升高），感冒可能诱发病情加重，需加强抗炎治疗而非仅对症处理！

- 所有药物（包括OTC感冒药）均需阅读说明书，避免成分重复或禁忌组合。

38.---

### **强直性脊柱炎（AS）患者怀孕/备孕的科学管理**

强直性脊柱炎（AS）患者可以怀孕并生育健康宝宝，但需在孕前、孕期和产后进行系统管理，以平衡疾病控制与母婴安全。以下是具体建议：

---

### **一、AS对怀孕的影响**

1. **疾病活动度与妊娠风险**

- **病情稳定期**：妊娠风险与健康人群相近，多数可顺利分娩。

- **高疾病活动度**：可能增加早产、低出生体重风险，需孕前控制病情。

- **孕期症状变化**：约1/3患者孕期症状减轻（孕激素抗炎作用），但产后6个月内复发风险较高。

2. **脊柱活动受限的影响**

- **麻醉风险**：严重脊柱强直可能增加椎管内麻醉难度，需提前与麻醉科沟通。

- **分娩方式**：若无骨盆受累，可尝试自然分娩；若脊柱/髋关节严重强直，建议剖宫产。

---

### **二、孕前准备与药物调整**

#### **1. 孕前评估与计划**

- **最佳妊娠时机**：病情稳定≥6个月（CRP/血沉正常，无关节肿痛）。

- **多学科会诊**：风湿科 + 产科 + 生殖科联合制定方案。

#### **2. 药物调整（孕前3-6个月）**

| **药物类型** | **孕前处理建议** |

|-----------------------|---------------------------------------------------------------------------------|

| **NSAIDs（如布洛芬）** | 孕前3个月停用，改用对乙酰氨基酚短期镇痛（避免影响排卵和胚胎着床）。 |

| **甲氨蝶呤** | 孕前3个月停用，补充叶酸（5mg/日）至孕后12周。 |

| **来氟米特** | 需用消胆胺洗脱（11天）或停药2年，否则致畸风险高。 |

| **生物制剂（TNF-α抑制剂）** | 可继续使用至妊娠中期（如阿达木单抗），部分药物（如依那西普）可持续用至孕晚期。 |

| **糖皮质激素** | 泼尼松≤10mg/日相对安全，但需监测血糖和血压。 |

---

### **三、孕期药物管理（安全用药推荐）**

| **药物类型** | **孕期安全性** | **使用建议** |

|-----------------------|-----------------------------------------------------------------------------|------------------------------------------|

| **TNF-α抑制剂** | ✔️ 孕早中期安全（胎盘转运少）<br> ❌ 孕晚期慎用（如英夫利昔单抗可能影响胎儿免疫） | 建议孕30周前停用，或选择胎盘通过率低的药物（如赛妥珠单抗）。 |

| **IL-17抑制剂** | 数据有限，孕早期慎用 | 病情需要时权衡利弊，优先TNF抑制剂。 |

| **对乙酰氨基酚** | ✔️ 全程可用（镇痛首选） | 每日≤3000mg，避免长期大剂量。 |

| **泼尼松** | ✔️ 低剂量安全（≤10mg/日） | 用于急性发作，需补充钙和维生素D。 |

| **柳氮磺吡啶** | ✔️ 安全（需补充叶酸） | 剂量≤2g/日，监测肝功能。 |

---

### **四、孕期监测与管理**

1. **疾病活动监测**

- 每3个月评估关节疼痛、晨僵及炎症指标（CRP/血沉）。

- 若病情加重：可短期使用泼尼松或调整生物制剂。

2. **胎儿健康监测**

- 孕20周后每4周超声评估胎儿发育（尤其使用生物制剂者）。

- 无创DNA/羊水穿刺排除染色体异常（药物影响风险极低）。

3. **生活方式干预**

- **运动**：孕期瑜伽、游泳维持脊柱灵活性（避免仰卧过久）。

- **姿势保护**：使用孕妇枕减轻腰背压力，避免久站久坐。

- **营养**：补充钙（1000mg/日）、维生素D（800IU/日）及叶酸。

---

### **五、分娩与产后管理**

1. **分娩方式选择**

- 自然分娩：适合无严重脊柱/髋关节强直、骨盆条件良好者。

- 剖宫产：若存在脊柱强直影响麻醉或胎位异常。

2. **产后哺乳与用药**

- **生物制剂**：TNF抑制剂（如阿达木单抗）哺乳期可用（乳汁中浓度极低）。

- **NSAIDs**：布洛芬在哺乳期安全，避免双氯芬酸。

- **糖皮质激素**：泼尼松＜20mg/日可哺乳（服药后4小时再喂奶）。

3. **产后复发预防**

- 产后6周重启生物制剂（如哺乳期可用药物）。

- 避免劳累，逐步恢复锻炼。

---

### **六、遗传风险与生育建议**

- **遗传概率**：AS与HLA-B27相关，若父母一方阳性，子女遗传概率约50%，但仅5-20%携带者会发病。

- **男性患者注意**：备孕前停用来氟米特（需洗脱）和沙利度胺，其他药物（如生物制剂）不影响精子质量。

---

### **七、总结建议**

1. **孕前**：病情稳定是关键，提前调整药物，补充叶酸。

2. **孕期**：以TNF抑制剂和对乙酰氨基酚为核心，定期多学科随访。

3. **产后**：警惕疾病复发，安全用药兼顾哺乳。

**重要提示**：AS患者完全可实现成功妊娠，但需严格遵循个体化方案，切勿自行停药或换药！

39.---

### **备孕期间强直性脊柱炎（AS）用药调整指南**

强直性脊柱炎患者在备孕期间需科学调整药物，以**兼顾疾病控制与胎儿安全**。以下是分性别、分药物的具体建议：

---

### **一、备孕通用原则**

1. **目标**：孕前3-6个月控制病情稳定（无疼痛、炎症指标正常）。

2. **多学科协作**：风湿科 + 产科联合制定方案，提前评估药物风险。

3. **补充叶酸**：每日0.4-5mg（具体剂量根据用药史调整）。

---

### **二、女性患者药物调整方案**

#### **1. 必须停用的药物**

| **药物** | **停药时间** | **替代方案** |

|-------------------|---------------------|------------------------------------------|

| **甲氨蝶呤** | 孕前3个月 | 改用生物制剂（如TNF-α抑制剂）或柳氮磺吡啶。 |

| **来氟米特** | 孕前2年，或洗脱（消胆胺11天） | 洗脱后需测血药浓度确认 ≤0.02mg/L。 |

| **JAK抑制剂** | 孕前1个月 | 换用TNF-α抑制剂（如阿达木单抗）。 |

#### **2. 可继续使用的药物**

| **药物** | **使用建议** |

|------------------------|-----------------------------------------------------------------------------|

| **TNF-α抑制剂** | 备孕期可继续使用（如阿达木单抗、赛妥珠单抗），孕早期部分药物需调整。 |

| **IL-17抑制剂** | 数据有限，备孕期慎用，优先选择TNF抑制剂。 |

| **柳氮磺吡啶** | 安全，但需补充叶酸（每日5mg）。 |

| **对乙酰氨基酚** | 短期镇痛首选，每日≤3000mg。 |

| **低剂量泼尼松（≤10mg/日）** | 可短期用于控制急性炎症，需监测血糖和血压。 |

---

### **三、男性患者药物调整**

| **药物** | **处理建议** |

|-------------------|-----------------------------------------------------------------------------|

| **甲氨蝶呤** | 无需提前停药（不影响精子质量），但部分指南建议停药3个月。 |

| **来氟米特** | 建议停药并洗脱（同女性）。 |

| **生物制剂** | 无需调整，可正常备孕。 |

| **NSAIDs** | 不影响精子，但长期使用可能降低生育力，备孕期可换对乙酰氨基酚。 |

---

### **四、替代治疗与注意事项**

1. **疼痛管理**：

- **禁用NSAIDs**：备孕女性停用布洛芬、塞来昔布等，改用对乙酰氨基酚。

- **物理治疗**：热敷、游泳、瑜伽缓解疼痛。

2. **疾病监控**：

- 每3个月复查血沉（ESR）、C反应蛋白（CRP），确保病情稳定。

- 若病情复发：优先使用TNF抑制剂或低剂量激素过渡。

3. **生活方式干预**：

- **戒烟**：吸烟降低药物疗效，增加流产风险。

- **适度运动**：维持脊柱灵活性，避免久坐。

---

### **五、备孕时间线示例**

| **阶段** | **行动要点** |

|------------------|-----------------------------------------------------------------------------|

| **孕前6个月** | 停用甲氨蝶呤/来氟米特，开始补充叶酸（5mg/日）。 |

| **孕前3个月** | 停用NSAIDs/JAK抑制剂，换用生物制剂或柳氮磺吡啶。 |

| **孕前1个月** | 复查炎症指标，确认病情稳定；男性完成药物洗脱（如需）。 |

---

### **六、常见问题解答**

#### **1. “停药后病情加重怎么办？”**

- **优先选择孕期安全药物**：如TNF抑制剂（阿达木单抗）或低剂量激素，而非冒险使用致畸药物。

#### **2. “叶酸要吃多少？”**

- **普通女性**：0.4mg/日。

- **曾用甲氨蝶呤者**：5mg/日（抵消甲氨蝶呤的叶酸拮抗作用）。

#### **3. “生物制剂影响精子/卵子质量吗？”**

- **无证据表明影响**，男女性均可正常备孕。

---

### **七、总结建议**

1. **提前规划**：至少孕前3-6个月调整药物，避免意外怀孕风险。

2. **核心药物**：TNF抑制剂（如阿达木单抗）是备孕期安全首选。

3. **动态监测**：定期评估病情与药物安全性，确保平稳过渡至妊娠期。

**重要提示**：个体差异大，所有方案需经风湿科医生确认，切勿自行停药！

40.---

### **核心结论**

目前，**中药或针灸无法替代西药（如非甾体抗炎药、生物制剂）作为强直性脊柱炎（AS）的核心治疗方案**。中西医结合可能作为辅助手段，但需基于科学证据和规范治疗框架。以下是详细分析：

---

### **一、中医治疗的局限性**

#### **1. 中药的疗效与风险**

| **类型** | **潜在作用** | **局限性** |

|----------------|-----------------------------------------------------------------------------|----------------------------------------------------------------------------|

| **祛风除湿类中药** | 可能缓解关节疼痛、僵硬（如雷公藤、白芍总苷） | - 起效慢，无法快速控制急性炎症<br>- 部分中药有肝肾毒性（如雷公藤可致生殖损伤） |

| **活血化瘀类中药** | 改善局部血液循环（如丹参、三七） | - 缺乏大规模临床研究证明对AS脊柱病变有效<br>- 与西药可能存在相互作用（如增强抗凝药效果） |

| **中成药** | 部分复方制剂（如痹祺胶囊）可能短期缓解症状 | - 成分复杂，质量控制参差不齐<br>- 可能含未标注的西药成分（如激素）导致副作用 |

#### **2. 针灸的辅助作用**

- **可能改善症状**：通过刺激穴位缓解疼痛、放松肌肉，但对炎症和骨破坏无直接抑制作用。

- **研究证据不足**：多数研究样本量小、设计不严谨，无法证明其替代西药的有效性。

---

### **二、西医治疗不可替代的核心原因**

1. **控制炎症与延缓结构损伤**

- **NSAIDs**（如塞来昔布）：快速抑制前列腺素，缓解疼痛和晨僵。

- **生物制剂**（如TNF-α抑制剂）：靶向阻断炎症因子，显著延缓脊柱强直和髋关节破坏。

- **证据等级**：西医方案有大量随机对照试验（RCT）和长期随访数据支持。

2. **疾病进展风险**

- AS的自然病程显示，未经规范抗炎治疗的患者中，约30%会发展为严重脊柱强直或髋关节受累致残。

- 中药/针灸无法阻止骨赘形成或韧带骨化，延误治疗可能导致不可逆损伤。

---

### **三、中西医结合的应用场景**

在规范使用西药的基础上，中医可尝试以下辅助治疗（需医生指导下进行）：

| **场景** | **中医干预** | **注意事项** |

|------------------------|---------------------------------------|-----------------------------------------------------------------------------|

| **西药副作用管理** | 中药调理胃肠道（如党参、白术） | 避免与西药同服，间隔1-2小时，防止相互作用。 |

| **缓解慢性疼痛** | 针灸、艾灸局部穴位（如肾俞、命门） | 避免在皮肤感染或血小板减少时使用。 |

| **改善疲劳与睡眠** | 酸枣仁汤、归脾汤等安神类方剂 | 需中医师辨证施治，避免自行滥用。 |

---

### **四、风险警示**

1. **自行停用西药**：

- 可能导致炎症失控、骨破坏加速，甚至出现脊柱骨折或髋关节强直。

2. **中药安全性问题**：

- 部分中药（如马兜铃酸）有明确肾毒性，长期使用可致肾衰竭。

- 中成药添加激素/镇痛药（如地塞米松、双氯芬酸）导致副作用风险。

---

### **五、总结建议**

1. **核心治疗**：以NSAIDs、生物制剂等西药为主，早期规范使用是避免残疾的关键。

2. **辅助选择**：在病情稳定期，可尝试中医调理（如针灸缓解僵硬、中药改善体质），但需选择正规中医院。

3. **禁忌行为**：

- 拒绝西药，完全依赖中医治疗。

- 轻信“祖传秘方”“彻底治愈AS”等不实宣传。

**重要提示**：

- 若考虑中西医结合治疗，务必告知风湿科医生您使用的中药/针灸方案，以避免药物相互作用或延误病情！

- AS是慢性病，需长期管理，任何治疗调整均应以科学证据和医生指导为前提。

41.---

### **核心结论**

即使存在腰痛，**强直性脊柱炎（AS）患者仍需坚持科学锻炼**！合理的运动不仅能缓解疼痛，还能延缓脊柱强直、维持关节功能。关键在于**选择适合的运动类型、控制强度并及时调整**。以下是具体指导：

---

### **一、AS患者必须锻炼的核心原因**

1. **对抗炎症与僵硬**

- 运动促进血液循环，帮助清除炎症因子，减轻晨僵和疼痛。

- 长期缺乏活动会加速韧带骨化和脊柱融合。

2. **维持关节活动度**

- 脊柱和髋关节的伸展运动可防止畸形（如驼背、颈椎强直）。

3. **增强肌肉保护**

- 核心肌群（腰背、腹部）强化可分担脊柱压力，减少损伤风险。

---

### **二、腰痛时如何安全锻炼？**

#### **1. 急性炎症期（疼痛剧烈、血沉/CRP明显升高）**

| **推荐运动** | **作用** | **注意事项** |

|----------------------|---------------------------------|-----------------------------|

| **深呼吸训练** | 维持胸廓活动度，防止肋骨融合。 | 坐/立位缓慢深吸气，每日3组×10次。 |

| **温和拉伸** | 缓解肌肉痉挛，改善僵硬。 | 仰卧位抱膝至胸部，保持15秒，左右交替。 |

| **水中活动** | 浮力减轻关节负荷，放松肌肉。 | 水温32-35℃，仅行走或漂浮，避免剧烈动作。 |

#### **2. 慢性疼痛期（轻度不适，炎症指标正常）**

| **推荐运动** | **频率/强度** | **细节要点** |

|----------------------|--------------------------------|-----------------------------|

| **游泳（蛙泳/自由泳）** | 每周3-4次，每次30分钟 | 避免蝶泳（过度伸展腰椎）。 |

| **瑜伽/普拉提** | 每周2-3次，以拉伸和核心训练为主 | 避免脊柱过度后仰（如轮式）。 |

| **太极/八段锦** | 每日20分钟，动作缓慢流畅 | 重心转移时保持腰背挺直。 |

---

### **三、腰痛锻炼的禁忌与警告**

| **动作/行为** | **风险** | **替代方案** |

|----------------------|---------------------------------|-----------------------------|

| **长时间弯腰** | 加重腰椎压力，诱发椎间盘突出。 | 改用深蹲（保持腰背直立）取物。 |

| **高强度跑跳** | 冲击力加速髋关节损伤。 | 选择椭圆机或骑自行车（调高车座）。 |

| **仰卧起坐** | 增加脊柱剪切力，加重疼痛。 | 平板支撑（30秒×3组）强化核心。 |

---

### **四、疼痛管理结合锻炼的策略**

1. **运动前准备**

- 热敷或热水浴10分钟，放松肌肉。

- 口服NSAIDs（如塞来昔布）30分钟后再锻炼，减轻疼痛干扰。

2. **运动中调整**

- 使用护腰（仅限急性期短期使用），避免依赖。

- 疼痛评分＞4分（0-10分）时立即停止，改为休息或低强度活动。

3. **运动后恢复**

- 冰敷疼痛部位15分钟（急性期）。

- 补充水分和蛋白质，促进肌肉修复。

---

### **五、个性化锻炼方案制定**

1. **评估疾病状态**

- 炎症活动期：以温和拉伸为主，避免负重。

- 稳定期：逐步加入抗阻训练（如弹力带）。

2. **根据受累部位调整**

- **髋关节受累**：多练桥式、侧抬腿，少做深蹲。

- **胸椎强直**：增加扩胸运动和呼吸训练。

3. **定期复评**

- 每3个月由康复科医生评估关节活动度和肌肉力量，调整运动计划。

---

### **六、总结建议**

1. **必须坚持**：即使疼痛，每日至少完成10分钟拉伸+20分钟有氧运动。

2. **循序渐进**：从低强度开始，逐渐增加时长和难度。

3. **疼痛是信号，而非禁令**：学会区分“活动性疼痛”（可耐受）与“损伤性疼痛”（需停止）。

**关键原则**：

- **动比不动好**，但**科学动比盲目动更重要**！

- 若运动后疼痛持续＞2小时或次日加重，需降低强度并咨询康复医生。

42.---

强直性脊柱炎（AS）患者是否需要手术，主要取决于**疾病是否导致严重的结构性损伤或功能障碍**。绝大多数患者通过规范药物治疗和康复锻炼即可控制病情，**手术并非首选治疗方案**，但在以下特定情况下可能需要考虑：

---

### **一、需要手术的常见情况**

| **手术类型** | **适应症** | **手术目的** |

|-----------------------|---------------------------------------------------------------------------|------------------------------------------|

| **髋关节置换术** | 髋关节严重破坏、强直或融合，导致行走困难或剧烈疼痛。 | 恢复关节活动度，改善行走能力。 |

| **脊柱矫形手术** | 严重驼背畸形（脊柱后凸＞60°），影响呼吸、视线或平衡。 | 矫正脊柱曲度，减少内脏压迫。 |

| **椎体骨折修复术** | 因骨质疏松或外伤导致脊柱骨折，伴神经压迫或脊柱不稳定。 | 稳定脊柱，解除神经压迫。 |

| **椎管减压术** | 脊柱强直合并椎管狭窄，出现严重神经症状（如肢体麻木、大小便障碍）。 | 缓解神经压迫，防止永久性损伤。 |

---

### **二、手术评估的关键因素**

1. **疾病活动度**：需在炎症控制稳定期手术（血沉/CRP正常），否则可能加重术后感染风险。

2. **年龄与健康状况**：年轻患者优先选择关节置换（假体寿命约15-20年），老年患者需评估心肺功能。

3. **生活质量影响**：若畸形或疼痛已严重限制日常生活（如无法平视、坐下或行走），手术获益更大。

---

### **三、手术风险与局限性**

| **风险类型** | **具体内容** |

|-----------------------|---------------------------------------------------------------------------|

| **感染** | 长期使用生物制剂或激素的患者感染风险增加（需术前停药2-4周）。 |

| **血栓** | 脊柱或关节术后需长期卧床，深静脉血栓风险升高（需抗凝治疗）。 |

| **假体松动/磨损** | 髋关节置换术后假体可能因活动过度或骨质疏松提前失效（年轻患者需谨慎）。 |

| **神经损伤** | 脊柱手术可能损伤脊髓或神经根，导致瘫痪风险（发生率＜1%）。 |

---

### **四、非手术治疗的核心地位**

1. **药物治疗**：

- **NSAIDs**（如塞来昔布）：缓解疼痛和炎症，延缓骨赘形成。

- **生物制剂**（如TNF-α抑制剂）：抑制疾病进展，降低关节破坏风险。

2. **康复锻炼**：

- 每日脊柱伸展（如猫牛式）、游泳等维持关节活动度，预防畸形。

3. **物理治疗**：

- 热疗、超声波缓解局部僵硬，增强肌肉力量。

---

### **五、总结建议**

1. **绝大多数患者无需手术**：通过早期规范治疗（药物+锻炼）可有效避免严重畸形。

2. **手术是最后选择**：仅在结构损伤严重影响功能时考虑，需由风湿科与骨科医生共同评估。

3. **术后仍需长期管理**：即使手术成功，仍需坚持抗炎治疗和康复训练，防止病情复发或假体问题。

**关键提示**：

- 若出现进行性驼背、髋关节活动丧失或神经症状，尽早就诊评估手术指征！

- 手术无法治愈AS，但可显著改善生活质量，需权衡利弊后决策。

**Chat GPT 4.0**

**一、诊断前的疑问与需求（在这部分问题前，请加入“这是我目前的症状，请你作为一名专业的医务工作人员向我提供健康意见”预设环境，每个问题请分别询问，避免“AI记忆”对回答造成干扰）**

1. **导致我腰背疼痛的原因是什么？这是我目前的症状，请你作为一名专业的医务工作人员向我提供健康意见**

腰背疼痛可能是由于多种原因引起的，包括肌肉劳损、椎间盘问题、姿势不良、骨骼问题、内脏疾病以及精神压力等。不过，要确定确切的原因，需要具体了解你的详细症状、病史以及生活习惯。以下是导致腰背疼痛的一些常见原因：

### 医疗专业建议：

#### 1. **症状自查**

- 回忆疼痛的触发因素：是运动导致，还是突发？

- 确认是否有腿部麻木、肌肉乏力、或其他放射性症状。

#### 2. **调整生活习惯**

- **姿势纠正**：是否有习惯性弯腰？建议保持脊柱自然曲线。

- **适当休息和缓解压力**：特别是处于久坐或高压状态时。

- **避免不当用力**：不要突然提重物！

#### 3. **物理疗法及舒缓方法**

- 适当进行腰背肌肉的锻炼或放松（如瑜伽、腰背伸展运动）。

- 热敷或物理按摩缓解轻微疼痛。

#### 4. **注意警示症状**

如果出现以下情况，应立刻就医：

- 疼痛加剧且持续时间超过数天。

- 有腿部麻木或肌力减弱。

- 发热或体重异常下降。

#### 5. **体检与医学检查**

- 如问题持续，建议通过咨询医生进行详细检查（如X光、核磁共振或血液检测）以排除潜在疾病。

1. **晚上睡觉的时候背痛的厉害，起来活动反而会好一些，我这种痛可能是什么疾病？这是我目前的症状，请你作为一名专业的医务工作人员向我提供健康意见**

对于您提到的睡觉时背部疼痛显著，起床活动后疼痛有所缓解的症状，这种情况可能与多种原因相关，以下是不常见的医学推测，但仅供您参考，最终需结合详细的检查来诊断：

。

### **建议与指导：**

#### 1. **评估生活方式和习惯**

- **观察睡姿和睡眠环境**：确保使用适当支撑的床垫和枕头，避免因床垫过硬或过软引发的肌肉或脊柱问题。

- **改良身心行为**：在白天适当增加活动和锻炼，尤其是针对背部肌肉的强化和拉伸运动（例如瑜伽或核心锻炼）。

#### 2. **注意其他伴随症状**

- 是否有晨起僵硬、关节疼痛或活动受限？

- 是否有乏力、发热、体重减轻等全身症状？

- 是否有尿频、尿痛或小便异常？

#### 3. **就医检查建议**

请尽快预约医生，特别是**骨科、风湿免疫科或内科**，并考虑以下检查：

- **影像学检查**：

- X光片或MRI，可明确脊柱或软组织的结构异常。

- **实验室检查**：

- 血液指标（如HLA-B27，用于排查强直性脊柱炎；炎症反应指标如C反应蛋白、血沉）。

- **尿液检查**：

- 排查潜在的肾脏问题。

---

### 4.注意事项

这种症状若不重视可能会影响生活质量甚至加重原有疾病风险，尽早寻求专业诊断和治疗至关重要。平时注意记录疼痛发生的规律和诱因以便医生诊断。

您若有进一步的具体情况（如疼痛是否伴随其它症状、药物使用史），可随时补充说明，我能更详细为您提供帮助。

1. **早上起床身体硬的动不了，可能是什么问题？这是我目前的症状，请你作为一名专业的医务工作人员向我提供健康意见**

身体早上起床时感觉“硬得动不了”，可能与以下原因有关：

### 建议

1. **居家调理**：

- 做一些晨起拉伸运动，比如**全身拉伸、瑜伽、轻微活动**，可以帮助缓解僵硬。

- 检查你的睡姿、床垫和枕头，尝试选择符合人体工学的产品。

- 睡前用温水泡脚或者局部热敷，促进血液循环。

2. **观察症状**：

- 观察僵硬的持续时间：仅仅几分钟缓解，还是持续较长时间？

- 记录近期是否有其他不适，如关节红肿、疼痛、疲惫，甚至体重减轻。

- 注意是否有晨僵超过30分钟的情况，这通常可能提示风湿性疾病。

3. **寻医问诊**：

如果症状持续或加重，建议尽早到医院就诊，特别是以下情况：

- **晨僵持续时间超过30分钟**，或伴关节红肿、削瘦等信号。

- 感觉疲劳、食欲不振、体重异常下降。

- 出现明显的关节疼痛或活动受限。

可以咨询以下科室：

- **风湿免疫科**：排查类风湿性关节炎、痛风等免疫性或代谢性疾病。

- **骨科**：检查是否存在骨骼或脊椎问题。

- **康复医学科**：在医生指导下，学习正确的运动和姿势改善。

---

### 注意事项

- 如果僵硬感伴有剧烈疼痛或其他全身严重症状（如发烧、出汗、皮疹），应立即就医。

- 避免自行服用止疼药或激素类药物，以免掩盖病情。

希望您尽快查明原因，恢复健康。如能提供进一步的症状细节，我可以帮助更精准地分析！

1. **腰疼和我眼睛痒、拉肚子有没有关联？这是我目前的症状，请你作为一名专业的医务工作人员向我提供健康意见**

虽然我不是医生，但可以为你提供一些健康建议。不过，为了更好地理解你的症状并判断是否相关，以下是一些可能的解释和建议：

### 健康建议

1. **观察病情**：

- 如果症状持续（如腹泻超过2天，腰痛无法缓解，眼睛症状加重），应及时就医。

- 记录症状出现的时间、加重或缓解的因素，并注意是否有其他相关症状（如发热、尿液异常）。

2. **保持水分**：

- 腹泻可能导致脱水，建议多喝温水和电解质以防止脱水。

- 如果腰痛涉及肾脏问题，保持适当饮水也能帮助代谢废物。

3. **饮食调整**：

- 避免辛辣、油腻和不易消化的食物，改以清淡饮食。

- 如果怀疑过敏，避免接触可能的过敏源（如尘螨、花粉）。

4. **注意眼睛护理**：

- 对眼睛痒，可清洗眼部或使用人工泪液，但避免揉眼。

- 如果眼睛有红肿或分泌物增多，应考虑就诊眼科检查。

5. **及时就医**：

- 如果伴有发热、尿痛、血便、视力模糊或症状显著加重，或难以判断症状是否相关，建议尽快前往医院，进行详细检查（如血液化验、尿液检查、肠胃镜或影像学检查）。

### 建议去的科室

- **消化内科**：检查腹泻原因。

- **眼科**：检查眼部瘙痒是否为炎症或过敏。

- **骨科或风湿免疫科**：若腰痛明显或怀疑全身性疾病，可进一步检测强直性脊柱炎或其他潜在问题。

- **泌尿外科**：若腰痛怀疑和肾脏相关。

症状复杂而非单一问题时，联络全科医生可能是一个较好的初步选择。如果方便，尽早安排体检以排除潜在疾病。

1. **我的腰疼会不会是很严重的疾病？这是我目前的症状，请你作为一名专业的医务工作人员向我提供健康意见**

腰疼可能由多种原因引起，从轻微的不适到潜在的严重疾病，原因包括肌肉劳损、姿势问题、椎间盘问题以及其他潜在的内科或外科疾病。在提供具有针对性的意见前，请尽量描述您的情况，包括以下内容：

1.**疼痛性质**：疼痛是刺痛、钝痛还是间歇性放射痛？

**疼痛部位**：是在下腰部、两侧、还是局部集中？

**病史**：您是否有相关外伤史？是否有长期坐姿或弯腰工作的习惯？

**诱因**：疼痛是否因活动、弯曲或坐着时间过久而加重？

**伴随症状**：是否有发热、下肢麻木、无力、排尿困难或大便失禁？

**持续时间**：这种疼痛是否是突然发生的？或已持续存在一段时间？

**缓解方法**：是否尝试过休息、热敷或其他缓解疼痛的方式？效果如何？

2.通常情况下，腰疼的常见原因包括：

- **肌肉拉伤或韧带损伤**：多与突然的运动或长时间姿势不当有关。

- **椎间盘突出**：可能伴随向腿部放射的疼痛（坐骨神经痛）。

- **姿势不正或久坐**：长期不正确的坐姿会引起慢性的腰部不适。

- **肾脏问题**：例如肾结石或肾炎，可能引起腰部两侧的疼痛，往往伴有尿频、尿痛等。

- **更为严重的疾病**：如骨折、肿瘤、椎管狭窄等，可能导致持续难以缓解的深层疼痛。

3.### 需警惕的危险信号

如果您出现以下情况，请尽快就医：

- 疼痛持续加重或严重影响日常活动。

- 伴有发热、体重减轻、虚弱、明显的神经症状。

- 身体的一侧肢体无力或者麻木。

- 尿失禁或大便失控。

- 显著的夜间疼痛或休息时无法缓解的疼痛。

如果您愿意提供症状的具体描述，我可以帮您进一步分析或建议是否该就医检查。

健康问题总是不可忽视，早诊早治是最重要的。

1. **我这种腰痛，要不要去看医生？****这是我目前的症状，请你作为一名专业的医务工作人员向我提供健康意见**

很高兴能为您提供帮助！但由于我并非真正的医护人员，也无法取代专业医生的诊断，仅能基于您描述的症状提供一些初步建议。因此，鉴于“腰痛”可能的原因非常多，您可以先思考以下问题并观察自己的具体情况：

### 常见原因：

1.腰痛的原因可以从轻微到严重不等，以下是几个常见的情况：

- **肌肉扭伤或紧张**：如搬重物或姿势不正确导致。

- **腰椎间盘问题**：包括椎间盘突出、椎管狭窄等。

- **姿势不良**：长时间的坐姿或站姿不佳。

- **内脏问题**：例如肾脏感染或结石等，可能引起辐射性腰痛。

- **风湿或其他炎症性疾病**：如强直性脊柱炎。

- 少数情况下，可能提示更严重的问题，例如感染或肿瘤。

2.### 如果出现以下“危险信号”，应尽快就医：

. 严重的疼痛影响正常生活，或者在休息后也没有改善。

. 腰痛伴有腿部明显的麻木、刺痛、虚弱。

. 不明原因的体重减轻、全身乏力、发烧。

. 伴随尿频、尿急或小便困难（可能涉及肾脏或泌尿感染问题）。

. 痛感严重到影响睡眠，或夜间疼痛加重。

3.### 建议的初步处理（如果不严重）：

- **休息与避免长时间的姿势压力**：给腰部充分休息。

- **局部热敷或冷敷**：急性疼痛建议冷敷，慢性问题可能用热敷缓解。

- **适当伸展**：进行一些轻柔的伸展运动，避免扭伤或过度拉伸。

- **合理使用非处方止痛药**：如布洛芬或扑热息痛用以缓解症状，但不可长期依赖。

不过，这些仅为一般性的建议，疼痛的具体情况和背后的原因需要根据您描述的信息进一步判断。如果疼痛持续且不明原因，还是建议您尽早前往正规医疗机构挂号诊治。这将确保您的症状得到及时且正确的处理！

1. **我家里有亲戚是强直性脊柱炎，现在我背痛会不会也是呀？这是我目前的症状，请你作为一名专业的医务工作人员向我提供健康意见**

首先，非常理解您对健康的担忧。强直性脊柱炎（AS）是一种常见的炎性疾病，主要影响脊柱和骶髂关节，但并不是所有背痛的人都一定与此疾病有关。以下是一些综合的、基于专业医学的参考意见，帮助您进一步了解和判断：

### 背痛是否与强直性脊柱炎相关

1. **特征性症状**：

- 强直性脊柱炎患者的疼痛通常表现为**炎性腰背痛**，常发生在年轻人群（20-40岁）。

- 疼痛可能在夜间加重，早晨起床感觉僵硬，但通过活动后有所改善。

- 严重时可能伴随脊柱活动受限，以及背部肌肉长期紧张僵硬。

2. **其他症状**：

- 强直性脊柱炎还可能伴随骶髂关节区域的疼痛（臀部深处的酸痛感）。

- 在某些病例中，患者可能出现全身乏力、疲劳、甚至其他部位如髋关节、膝关节的疼痛。

- 可合并“虹膜炎”或其他免疫性反应性问题。

3. **高危因素**：

- **家族史**：如果您的亲戚患有这种疾病，您可能遗传性携带HLA-B27基因（一种与强直性脊柱炎相关的基因），但这不等于您一定会发病。

- 是否有长期久坐、体姿不佳、压力等生活习惯可能引发肌肉劳损。

---

### 对您目前的建议

1. **观察背痛的性质与过程**：

- 背痛是否伴随**夜间睡眠干扰**或晨起僵硬，或通过锻炼后缓解？若是，可能与炎性问题相符。

- 是否存在明确诱因（例如搬重物、扭伤、过度久坐导致的肌肉劳损）？这可能更多是机械性疼痛。

2. **建议体检**：

- 前往正规医院的骨科或风湿免疫科，描述您的症状并获取专业建议。

- 医生通常会进行**骶髂关节X光**或**MRI检查**，并可能安排**HLA-B27基因检测**以明确诊断。

- 除此之外，还可能检查炎症指标（如C反应蛋白CRP和血沉ESR）。

3. **自我管理**：

- 注意良好的坐卧姿势，避免久坐和长时间驼背；

- 适量进行低冲击的运动（如游泳或瑜伽）来保护和加强脊柱柔韧性；

- 注意饮食健康，避免服用烟酒等可能加重炎性反应的习惯。

如果您有后续检查报告或需要具体化症状分析，请随时说明，我可以进一步为您提供帮助！

1. **强直性脊柱炎的背痛和其他背痛有什么区别？这是我目前的症状，请你作为一名专业的医务工作人员向我提供健康意见**

强直性脊柱炎（AS）是一种慢性炎性疾病，主要影响脊柱和骨盆的骶髂关节，也可能波及周围的关节和其他组织。它的症状可能与普通的背痛混淆，但有一些明显的区分点可以帮助识别强直性脊柱炎的背痛。以下是两者的区别以及我的一些健康建议：

### **1. 背痛的区别**

#### **强直性脊柱炎的背痛特点**

- **起病隐匿**：强直性脊柱炎的背痛通常是缓慢起病，而不是突然发生。

- **疼痛类型**：多为**炎性背痛**，清晨时疼痛更为明显，并伴有晨僵（起床后脊柱僵硬感）。

- **运动可缓解**：活动后疼痛减轻，而久坐或休息时会加重。

- **疼痛位置**：疼痛通常集中在**下腰部**，骶髂关节（腰部和臀部交界处）是最常受累的部位，有时疼痛可能延伸到臀部或大腿区域。

- **年轻人发病为主**：发病通常在40岁之前，尤以20岁至30岁之间常见。

- **可能伴随其他症状**：

- 下背僵硬；

- 胸廓活动受限，深呼吸时感觉胸腔紧缩；

- 眼部炎症（虹膜炎）或全身其他慢性炎症症状。

#### **普通机械性背痛的特点**

- **起病：**通常**突然发作**，可能与特定姿势、不当运动、外伤等诱因相关。

- **疼痛特点：**多为**机械性背痛**，劳动时疼痛加重，休息后可缓解。

- **疼痛部位：**集中于肌肉或椎间盘区域，可能伴随肌肉痉挛，但往往不涉及骶髂关节。

- **晨僵少见：**通常不会有明显的晨僵，休息后反而感觉好转。

---

### **2. 健康建议**

如果你怀疑你的背痛与强直性脊柱炎有关，我建议采取以下措施：

#### **对症护理**

- **适度活动**：早晨感到僵硬时，轻柔的伸展运动可能有所帮助；长期保持脊柱的灵活性对控制症状很有帮助。

- **热敷或温水泡澡**：热敷可以缓解疼痛和僵硬，尤其是清晨背痛明显时。

- **保持良好姿势**：注意坐姿、站姿，以减少脊柱的压力。

3. **立即就医的原因和建议**

如果你的背痛符合以下情况，建议尽快就医：

. 清晨痛明显，活动后缓解，但久坐或躺下后又加剧；

. 背痛持续时间**超过3个月**，而且逐渐加重；

. 背部疼痛伴有晨僵、臀部疼痛，或胸廓扩张受限；

. 出现其他相关表现，如**视力模糊、眼红（虹膜炎）、疲劳或其他关节疼痛**。

就医时，可以咨询风湿免疫科医生，在咨询中可能需要进一步检查，比如:

- 骶髂关节X光或者MRI扫描来确定是否有炎症或骨骼改变；

- 血液检查：例如**HLA-B27基因检测**、C反应蛋白（CRP）和血沉（ESR），以了解炎症状态。

4.#### **慢性管理**

如果确诊为强直性脊柱炎，需要采取长期管理，这可能包括：

. **药物治疗**：

- **NSAIDs（非甾体抗炎药）**：如常见的布洛芬，用于缓解疼痛和炎症；

- **生物制剂**：如抑制肿瘤坏死因子（TNF）或IL-17的药物（例如依那西普、阿达木单抗等），可以有效控制疾病进展。

. **物理治疗**：专业的理疗可以帮助保持关节灵活性。

. **定期随访**：避免疾病对脊柱和其他器官（如眼部、心脏等）的长期损害。

---

5.### ** 预防和生活方式调整**

尽管某些因素（如基因）不可更改，但以下方法可以帮助改善强直性脊柱炎的症状并提高生活质量：

- 适度锻炼：如游泳、瑜伽、普拉提，有助于改善活动范围和背部的僵硬。

- 健康饮食：优质蛋白、Omega-3脂肪酸（如三文鱼、亚麻籽油）和抗炎食品（如蔬菜和水果）可能有助于减轻炎症。

- 睡眠：确保睡在较硬的床垫上，以支持脊柱的自然曲线。

- 控制情绪：慢性疾病可能带来压力，学习一些情绪管理技巧（如冥想）可能有帮助。

---

### **总结**

你提到的背痛可能与强直性脊柱炎有关，这需要通过临床检查和辅助检查来确诊。如果你符合上述炎性背痛的特征，我建议尽早前往风湿免疫科进行评估，以便确诊和开始治疗。这种疾病的早期管理可以显著改善症状并预防长期并发症！

1. **最近我胸口疼，为什么医生会觉得我可能是强直性脊柱炎？这是我目前的症状，请你作为一名专业的医务工作人员向我提供健康意见**

强直性脊柱炎（Ankylosing Spondylitis, AS）是一种慢性炎性疾病，主要影响脊柱和骶髂关节，但也可能伴随其他关节疼痛或身体症状。医生会考虑这个诊断，可能是基于您胸痛的性质及其与强直性脊柱炎的典型表现之间的相似性。

1.### 为什么胸口疼与强直性脊柱炎相关？

强直性脊柱炎不仅会影响脊柱，还可能波及肋骨与脊柱连接的地方或胸骨与肋骨的连接处（即肋椎关节或胸骨-肋软骨关节），导致胸部疼痛。一些患者因为这些关节的炎症，会感到胸部僵硬或胸腔活动受限，尤其是在深呼吸或运动时。

2.### 可能的其他相关症状（医生考虑的关键信息）

以下是医生可能会通过临床症状、病史或检查排查，并最终评估是否为强直性脊柱炎的关键点：

. **下腰背部疼痛和晨僵**：

- 疼痛通常在夜间或清晨较明显；

- 随活动疼痛缓解（而非加重）。

. **活动受限**：

- 上述疼痛可能逐渐限制您的脊柱运动，甚至影响日常动作。

. **家族史**：

- 是否有近亲属（如父母、兄弟姐妹）患有类似疾病？

. **HLA-B27检测**：

- 强直性脊柱炎与基因HLA-B27密切相关，医生可能会建议您进行相关血液测试。

. **影像学表现**：

- 验证骶髂关节的炎症（例如通过X光或MRI影像）。

3.### 胸口疼如何与其他疾病区分？

除此之外，医生需要非常谨慎，因为胸口疼也可能是以下疾病的表现：

- 心血管疾病（如心绞痛、心脏病）；

- 胸膜炎或肺部疾病；

- 胃肠道疾患（如胃食管反流病或胃溃疡）；

- 肌肉骨骼的其他病变（如肋软骨炎）。

因此，医生始终会通过体格检查、实验室指标（如炎症相关指标CRP、ESR）及影像研究等手段综合判断。

---

### 您的下一步建议：

4. **遵循医生的检查安排**：

- 如果尚未影像学检查（如X光或MRI），请及时进行；

- 配合完成HLA-B27等化验。

5. **记录症状变化**：

- 注意胸痛发生的时段、具体的位置、诱发或缓解的因素（如呼吸、体位变化等）。

6. **生活调整**：

- 坚持适度运动，尤其是增强脊柱灵活性的拉伸锻炼（需在医生指导下进行）；

- 保持正确的姿势和良好的睡眠支撑。

7. **药物治疗**（由医生指导）：

- 如果确诊为强直性脊柱炎，治疗方案可能包括非甾体抗炎药（NSAIDs）与生物制剂。

### 温馨提示

请及时与专业医生沟通您的疼痛特点，避免自行盲目用药。胸部疼痛虽听上去是轻症，也可能是更严重潜在疾病的信号。通过系统评价，能够找到最佳的治疗方式。

1. **腰疼和我的情绪压力有关么？这是我目前的症状，请你作为一名专业的医务工作人员向我提供健康意见**

腰痛和情绪压力之间可能存在一定的关系。在一些情况下，情绪压力和心理压力会导致肌肉紧张，特别是腰部区域的肌肉，这可能引发或加重腰痛。另外，长期的压力也可能通过内分泌系统对身体产生全身性的影响，例如使炎症反应加剧、增加疼痛的感知等。

根据目前的医学观点，以下几点值得注意：

### 1. **压力和腰痛的关联**

- **肌肉紧张**：情绪压力通常会导致肌肉处于较长时间的紧张状态，尤其在颈背和腰部容易受影响，从而引起或加剧疼痛。

- **神经系统影响**：长期的压力可能通过影响自主神经系统，降低身体的自我修复能力，延缓慢性疼痛的恢复。

- **睡眠不足**：压力可能影响睡眠质量，而睡眠不足会降低身体对于疼痛的耐受力，进一步加重不适感。

### 2. **您的症状可能的原因**

为了更具体地评估，以下是一些因病区分腰痛的可能原因：

- **肌肉或软组织劳损**：如果您近期有长时间保持一个姿势、剧烈运动或者搬重物，肌肉劳损是常见原因。

- **椎间盘问题**：如腰椎间盘突出、椎管狭窄，这通常与神经压迫有关，会引起腿部麻木或射痛。

- **压力性腰痛**：如果没有其他明确的异常可能，则心理因素和肌肉紧张都可能是主要诱因。

- **内科或妇科疾患**：如长期腰痛伴随其他症状（尿频、发热、经期变化等）可能需要关注肾脏、泌尿系统或盆腔问题。

### 3. **健康管理及建议**

无论腰痛是否由情绪压力引起，以下方法可能有所帮助：

- **放松减压**：

- 练习深呼吸、冥想或瑜伽，可以缓解压力，促进肌肉放松。

- 保证有规律的生活作息，尤其是充足的睡眠时间。

- **适度运动**：

- 做一些轻缓的拉伸运动，例如猫式伸展、下犬式等瑜伽动作，帮助缓解腰部压力。

- 避免过度运动或举重物，以免加重。

- **健康饮食**：

- 富含抗炎成分的食物，如鱼类、绿叶蔬菜和全谷物，可能有助于减轻炎症。

- **寻求心理支持**：

- 如果发现因压力导致的痛症较重，可以考虑与心理健康顾问或医生进行沟通。

- **就诊排查**：

- 若有持续性剧烈腰痛、伴随其他不适（如腿痛、麻木、发热等），请尽早就医，由医生排查器质性病因。

### 4. **医疗评估的必要性**

如果您的腰痛情况持续超过数周，或者加重，尤其伴随以下症状，则需要及时看医生：

- 下肢麻木或无力。

- 尿失禁或大便失禁。

- 发热或其他感染症状。

- 腰痛在夜间加重、影响睡眠。

您可以从以上建议尝试调整生活方式，并注意观察身体反应。如果有任何问题不确定，也欢迎进一步沟通！

**二、诊断过程中的困惑（在这部分问题前，请加入“目前我疑诊为强直性脊柱炎，在确诊过程中我产生了如下疑惑，请你作为一名专业的医务工作人员向我提供健康意见”，每个问题请分别询问，避免“AI记忆”对回答造成干扰）**

1. **目前我疑诊为强直性脊柱炎，在确诊过程中我产生了如下疑惑医生让我查HLA-B27，这个指标阳性是什么意思？请你作为一名专业的医务工作人员向我提供健康意见**

强直性脊柱炎（AS）是一种以脊柱及其周围关节慢性炎症为特点的疾病。HLA-B27是一种与强直性脊柱炎及其他一些自身免疫性疾病相关的重要遗传标志物。下面是对HLA-B27指标阳性的意义和相关专业解读：

### 1. **HLA-B27阳性是什么？**

HLA-B27是一类人体白细胞抗原系统（HLA）中的特定分子，主要与免疫系统功能相关。它的存在或缺失由基因决定。

- **阳性**意味着你的基因中存在这一抗原。

- 它与多种免疫性疾病，尤其是强直性脊柱炎，有显著相关性。

### 2. **HLA-B27阳性是否等于患病？**

并不是所有HLA-B27阳性的人都会患上强直性脊柱炎：

- 在普通人群中，HLA-B27阳性者约占8%-10%，但大多数不会发展为强直性脊柱炎。

- 然而，在强直性脊柱炎患者中，大约有80%-95%会是HLA-B27阳性。

- 换句话说，HLA-B27阳性增加了患强直性脊柱炎的风险，但不是决定性因素。

### 3. **HLA-B27检查的意义**

医生通过检测HLA-B27是为了：

- 辅助诊断强直性脊柱炎及类似的疾病。

- 如果有典型症状（如下背痛、晨僵、关节疼痛或活动受限）并伴随HLA-B27阳性，诊断强直性脊柱炎的可能性更高。

### 4. **HLA-B27阳性患者的健康建议**

如果你的HLA-B27检测结果为阳性，并担心强直性脊柱炎，以下几点有助于你的了解和管理：

- **疾病筛查**：除HLA-B27外，其它检查（如影像学检查：脊柱或骨盆X线/MRI）和临床症状同样重要。

- **监测症状**：早期症状如反复的下腰痛、晨僵、活动受限和夜间疼痛应得到关注。

- **健康管理**：

- 保持良好的体态和日常锻炼：如游泳、瑜伽有助于增强柔韧性。

- 避免长期保持固定、不良的坐姿或姿势。

- 如果确诊，应采取抗炎治疗和理疗措施。

### 5. **下一步建议**

- 根据医生建议完成相关补充检查（如影像学、炎症指标如C反应蛋白CRP或血沉ESR）。

- 如果确诊为强直性脊柱炎，积极与风湿免疫科医生合作，开展个性化的治疗和管理计划。

希望这些信息对你有帮助！如有进一步问题，可随时咨询医生或我继续帮你解答。祝你健康！

1. **目前我疑诊为强直性脊柱炎，在确诊过程中我产生了如下疑惑我的C-反应蛋白，血沉都高，和强直性脊柱炎有关系么？ 请你作为一名专业的医务工作人员向我提供健康意见**

强直性脊柱炎（Ankylosing Spondylitis，AS）是一种慢性、炎症性疾病，主要影响脊柱及骶髂关节，属于自身免疫相关性疾病。以下是关于C-反应蛋白（CRP）和血沉（ESR）与强直性脊柱炎相关性的解释和建议：

### 1. **C-反应蛋白（CRP）和血沉（ESR）在强直性脊柱炎中的意义**

- **CRP（C-反应蛋白）**：这是体内炎症反应的一个敏感指标。在强直性脊柱炎患者中，CRP水平升高可能表明疾病活动性增强，即某些炎症过程正在加重。这是临床判断炎症程度的重要参考之一。

- **ESR（红细胞沉降率，血沉）**：和CRP类似，ESR升高通常也是炎症反应的标志之一。在强直性脊柱炎患者中，ESR的升高可以反映疾病的活动状态。

- 并非所有强直性脊柱炎患者都会出现CRP和ESR的升高，尤其是早期患者或者疾病活动性较低的患者可能表现为正常。

### 2. **强直性脊柱炎与CRP、ESR的关系**

- **疾病活动性指标**：CRP和ESR的升高通常提示患者的强直性脊柱炎可能处于活动性状态，但它们并不是特异性的诊断指标。其他炎症性疾病、感染等因素也可导致这些指标升高。

- **个体差异**：部分强直性脊柱炎患者即使疾病活动显著，CRP和ESR也可能维持在正常范围，尤其在症状表现轻微时。

- **诊断中的辅助作用**：在结合临床症状（如晨僵、骶髂关节疼痛）、影像学检查（MRI或X线骶髂关节变化）及基因检测（如HLA-B27）后，CRP和ESR的变化有助于了解疾病的活动状态，但不能单独作为诊断依据。

### 3. **应对您的疑问的建议**

- **进一步诊断**：您需要综合其他检查结果来明确诊断，建议确认是否已经进行过以下检查：

- **影像学检查**：如骶髂关节的X线、MRI评估是否存在典型炎症或骨侵蚀性变化；

- **HLA-B27基因检测**：此标记在强直性脊柱炎患者中阳性率较高（但不是确诊的唯一条件）。

- **关注症状**：留意是否有晨僵、腰背部疼痛、疲劳等典型强直性脊柱炎症状。

- **排除其他病因**：CRP和ESR的升高可能与其他炎性或感染性疾病有关，因此需要排除可能的其他原因（如感染性疾病或其他风湿性疾病）。

- **就医跟踪**：及时与风湿免疫科或骨科专业医生沟通，将实验室和影像学结果结合起来进行综合判断。

### 4. **生活与管理建议**

- **身体活动**：适度的功能锻炼和运动（如游泳、瑜伽）有助于保持脊柱的灵活性。

- **健康饮食**：保持均衡饮食，避免过度饮酒及高糖高脂饮食。

- **早期干预**：如果确诊，应根据医生建议及时开始治疗（包括药物治疗如非甾体抗炎药或生物制剂）。

- **心理调整**：保持平稳心态，避免因疾病诊断疑虑过多而影响情绪。

如有其他具体症状或报告结果，分享后可进一步为您更精确地分析并提供健康建议。

1. **目前我疑诊为强直性脊柱炎，在确诊过程中我产生了如下疑惑为什么我疼的厉害，医生说我X光片没有问题？请你作为一名专业的医务工作人员向我提供健康意见**

您好，首先感谢您信任我，并向我提出您的困惑。强直性脊柱炎（AS，也称为脊柱关节病）确实是一种较复杂的疾病，患者在早期往往会面对一些类似您提到的情况，比如症状明显但影像学检查（如X光片）并未显示异常。这可能让人困惑，但这是强直性脊柱炎的早期表现特点之一。以下是一些可能的原因，以及我给出的健康建议：

1.### 为什么X光片可能未显示问题？

. **疾病早期变化不明显：**

- 在强直性脊柱炎的早期，尤其是病变刚开始阶段，炎症可能主要集中在骶髂关节的软骨或周围韧带上。这些变化在X光片上可能尚未显现。

- X光片通常更适合观察骨骼结构的明显异常，而对于早期的软组织或微小的骨质变化，灵敏度较低。

. **影像技术局限：**

- X光片的分辨率有限，如果您处于疾病早期，可能需要更敏感的检查手段，如**MRI（核磁共振）**，它能够检测软骨、韧带及早期炎症变化。

. **疼痛未必完全由结构性变化引起：**

- 疼痛可能源于炎症反应带来的释放物，例如炎性因子，它们会刺激神经，引起显著的疼痛感，而不一定与骨骼结构紊乱直接相关。

---

2.### 强直性脊柱炎早期诊断的重要性：

在强直性脊柱炎中，早期发现和干预非常重要。确诊过程可能需要结合以下方面：

. **症状体征：**

- 腰背痛，尤其是**夜间痛**或**晨僵感**，是强直性脊柱炎的典型症状。

- 疼痛往往伴有活动后缓解的特征。

. **血液检查：**

- 检测炎症指标（如C反应蛋白CRP、红细胞沉降率ESR）是否升高。

- 检查HLA-B27基因，这是强直性脊柱炎的重要标志，尽管不是每位患者都携带。

**影像学检查：**

- 核磁共振（MRI）比X光片更能清晰显示骶髂关节和其他部位的炎症。

---

### 我的建议

您可以采取以下步骤更好地与医生配合，促进诊断和管理：

1. **继续与主治医生沟通：**

- 如果X光片正常但症状显著，建议向医生明确提出进行更高级的影像学检查（如MRI）以及其他辅助检查。

2. **记录详细症状：**

- 日常生活中记录疼痛时间、部位、晨僵时长及活动后是否缓解等症状，提供给医生参考。

3. **早期管理：**

- 若确诊为强直性脊柱炎，治疗目标是控制症状、延缓病情进展，包括使用**非甾体抗炎药（NSAIDs）**、**生物制剂**或**疾病修饰抗风湿药物（DMARDs）**。

- 平时坚持适当的锻炼（如游泳、拉伸），可以改善症状，防止脊柱僵硬。

4. **心理调节与支持：**

- 不要因为短暂的困惑而焦虑，疾病确诊和治疗需要一个过程。保持积极乐观的心态，和家人或朋友分享您的感受，及时寻求心理支持。

5. **定期随访：**

- 即使症状能够缓解，也请按时回到医院复查，监测病情。

---

### 总结

疼痛与影像学检查结果不完全一致并不罕见，尤其在强直性脊柱炎的早期阶段。继续和专业医生保持沟通，完善检查手段，例如MRI或血液指标检测，可能有助于明确诊断。与此同时，早期采取健康管理措施可以有效改善症状并提高生活质量。

祝您早日查明病因，缓解疼痛！如果有进一步问题，随时欢迎咨询。

1. **目前我疑诊为强直性脊柱炎，在确诊过程中我产生了如下疑惑骶髂关节核磁显示的骨髓水肿严重么，会不会是强直性脊柱炎？请你作为一名专业的医务工作人员向我提供健康意见**

关于您的情况和疑问，我可以从医学的角度为您提供一些信息与建议，但请注意以下内容仅供参考，具体诊断和治疗建议请咨询您的专业医生。

---

**1. 骶髂关节骨髓水肿和强直性脊柱炎的关系：**

- 骶髂关节骨髓水肿在影像学检查（如核磁共振，MRI）中常表现为骶髂关节周围的高信号，这种情况可能提示炎症活动。

- 在强直性脊柱炎（Ankylosing Spondylitis，AS）的诊断过程中，骶髂关节的炎症是非常重要的参考指标。骨髓水肿常被视为炎性反应的直接证据，尤其是在骶髂关节区域，这可能与强直性脊柱炎相关。

- 但需要注意，骶髂关节的骨髓水肿并不是强直性脊柱炎的特异性表现，也可能见于其他原因引起的骶髂关节炎症（如感染、创伤、其他风湿性疾病等）。

**2. 强直性脊柱炎的诊断：**

- AS是一种风湿性疾病，通常与**HLA-B27阳性**、慢性背部疼痛和僵硬、影像学异常（如骶髂关节炎症）等特征相关。

- 医生通常会综合多种信息来作出诊断，包括：

- **临床表现**：如慢性背痛（通常超过3个月，休息后加重，活动后缓解）、晨僵、外周关节症状等。

- **实验室检查**：如HLA-B27基因检测、有无炎症标志物（如C反应蛋白CRP、血沉ESR）的升高。

- **影像学**：MRI检查是否显示骶髂关节有炎症性改变、骨髓水肿或骨质增生。

**3. 下一步建议：**

- **明确诊断**：建议与风湿免疫科医生进行充分沟通，进一步结合临床表现与检查结果进行判断。

- **排除其他疾病**：虽然骶髂关节水肿倾向于炎性改变，但也需要排查是否有其他因素（如感染性原因、创伤或者其他系统性疾病）导致类似表现。

- **日常管理**：在诊断明确前，无需过度担心，但谨记保持适当的运动（如避免过于剧烈的活动，同时增加低强度的伸展训练，如游泳或瑜伽），以保持关节的柔韧性和功能。

- **遵循医生建议**：医生可能会安排进一步的实验室检查、影像学检查或者尝试性药物治疗（如非甾体抗炎药NSAIDs），以帮助缓解症状和明确病因。

如有进一步的疑问，建议随时与风湿科或骨科医生沟通。您的配合和定期复查对疾病的早期诊断和干预尤为重要。

1. **目前我疑诊为强直性脊柱炎，在确诊过程中我产生了如下疑惑我该怎么看我是不是强直性脊柱炎？请你作为一名专业的医务工作人员向我提供健康意见**

强直性脊柱炎（AS）是一种以慢性炎症为主的自身免疫性疾病，主要累及脊柱和骶髂关节。确诊这一疾病通常需要结合病史、症状、体格检查、影像学检查以及实验室检查。那么，针对你的问题，以下是一些健康建议，供你参考：

---

### 自我评估：

**1. 症状观察：**

- **疼痛特点：** 是否存在慢性腰背部疼痛，尤其是晨起或久坐后加重，活动后通常会有所缓解。

- **晨僵表现：** 起床后关节僵硬的持续时间是否超过30分钟。

- **其他症状：** 是否有髋关节、肩关节或膝关节疼痛；眼睛炎症（虹膜炎）、疲劳或偶尔的发热。

**2. 疾病家族史：**

- 是否有家族成员早年发生类似症状或被确诊为强直性脊柱炎。

---

### 病院诊断步骤：

为了明确是否为强直性脊柱炎，应具体进行以下检查步骤：

**1. 医学影像学检查：**

- **X线检查：** 骶髂关节是否有损害（如关节融合、骨侵蚀等）。

- **核磁共振（MRI）：** MRI比X线更早期捕捉骶髂关节和脊柱的炎症或骨髓水肿。

**2. 实验室检查：**

- **HLA-B27检测：** HLA-B27阳性与强直性脊柱炎高度相关，但并非所有患者都阳性。

- **炎性指标：** 血沉（ESR）和C反应蛋白（CRP）的升高可能提示炎症活跃，但部分患者检查会正常。

**3. 体格检查：**

- 检查脊柱活动度，如前屈、后伸和侧弯是否受限。

- 骶髂关节或椎体是否有压痛。

---

### 后续：

如果你怀疑自己可能患有强直性脊柱炎，以下是几个必要的健康建议：

1. **寻求专业帮助：**

- 优先去风湿免疫科进行详细的诊断。

- 针对你的症状，医生可能会安排进一步的检查，共同制定治疗和管理计划。

2. **治疗管理：**

- **药物治疗：** 包括非甾体抗炎药（NSAIDs）、生物制剂（如抗TNF或IL-17抑制剂）等。

- **非药物干预：** 身体锻炼（如游泳、瑜伽）和物理治疗可以帮助维持脊柱的活动度。

3. **心理支持：**

- 如果你正在确诊过程中感到心理压力或担忧，可以与亲友或专业心理医生交流，获得情绪支持。

---

### 生活方式建议：

- 保持规律运动，避免长时间久坐；

- 选择健康的饮食，减少炎症触发因素；

- 保持合适睡姿，避免进一步加剧脊柱病变。

强直性脊柱炎的确诊并非单一检查能明确，需要综合考虑多方面信息。如果尚未确诊，建议保持平常心，及时寻求专业医疗结构支持，尽快明确诊断并制定合适的治疗计划。

1. **目前我疑诊为强直性脊柱炎，在确诊过程中我产生了如下疑惑医生开的X光，CT，磁共振检查这些影响检查和抽血化验我有必要全都做么？请你作为一名专业的医务工作人员向我提供健康意见**

强直性脊柱炎 (AS) 是一种慢性炎症性疾病，确诊可能需要结合临床症状、体征以及各种检查结果。以下是关于您提到的X光、CT、磁共振，以及抽血检查的作用和必要性的一些专业信息和建议：

---

### 1. **影像学检查的作用**

- **X光检查**：

- 最基础的影像学检查，主要用于观察骶髂关节、脊柱的早期变化，如关节间隙变窄、骨质侵蚀或骨赘形成。

- 通常作为确诊强直性脊柱炎的重要初步手段。

- 缺点是强直性脊柱炎在疾病初期的改变可能不容易通过X光显示。

- **CT检查**：

- 适合观察骨骼的细节，尤其是骶髂关节的早期病变，可以更好地显示微小的骨质破坏或关节粘连。

- 比X光更敏感，但放射剂量也相对较高。

- **磁共振成像 (MRI)**：

- 是目前诊断强直性脊柱炎较为敏感的影像学方法，特别是早期。

- 能检测出骶髂关节和脊柱的炎症（水肿）等软组织病变，是成像检查中的“金标准”。

- 通常在早期诊断有症状但X光检查阴性时使用。

**建议**：如果病情早期、X光未见明显异常而高度怀疑强直性脊柱炎，MRI检查可能是更优先的选择。是否需要做CT检查，可以根据您的具体病情及医生的建议而定。

---

### 2. **抽血化验的作用**

- **炎症指标**：

- 检查如 C反应蛋白 (CRP) 和红细胞沉降率 (ESR)，可反映体内是否存在炎症活动，但这些指标非特异性。

- **免疫相关检查**：

- **HLA-B27基因检测**：强直性脊柱炎患者中有很高比例为HLA-B27阳性（但并非所有AS患者都阳性，且阳性也不一定就有AS）。

- 结合HLA-B27和其他指标检测，可以增加诊断的依据。

- **其他血液检查**：

- 用于排除类似症状的其他疾病，例如类风湿因子 (RF) 用于排除类风湿关节炎。

**建议**：血液检查是确诊强直性脊柱炎的重要辅助，并可以用来评估炎症程度和排除其他类似疾病，建议配合进行。

---

### 3. **是否需要全都做？**

- **医生检查的必要性**：

医生开具的检查通常是根据您的病史、症状及体格检查结果综合判断的，不是每个患者都需要做所有检查。

- **个体化策略**：

- 如果临床症状及X光就能明确诊断，可能无需进一步MRI或CT检查。

- 如果X光结果不典型，但症状高度怀疑，建议优先MRI。

- 如果经济条件有限，可以与医生讨论，根据症状选择性进行有助于诊断的关键检查。

---

### 4. **行动建议**

. **与医生沟通：** 把你的经济、时间顾虑以及对检查的重要性疑惑及时与医生交流，医生可以调整检查方案。

. **遵循科学诊断流程：** 影像学检查与血液检查在确诊强直性脊柱炎时有互补作用，合理搭配有助于明确病因、确定治疗方向。

. **早期诊断与治疗：** 强直性脊柱炎确诊后及时治疗可以有效控制病情进展，因此建议积极配合医生进行必要的检查。

---

### 总结

目前进行X光、CT、MRI以及抽血检验是否全部都需要，应根据医生评估您当前病情的严重程度、初步检查结果以及经济条件来决定。MRI通常用于早期精确诊断或症状不典型的情况；抽血则对于评估炎症和进一步确诊具有重要意义。

若仍有困惑，请直接向主治医生咨询，他/她能够结合您的具体情况为您量身制定个性化的检查和治疗方案。

1. **目前我疑诊为强直性脊柱炎，在确诊过程中我产生了如下疑惑我已经不是青少年了，还会得强直性脊柱炎么？请你作为一名专业的医务工作人员向我提供健康意见**

强直性脊柱炎（AS）是一种慢性炎症性疾病，主要影响脊柱和骶髂关节，属于脊柱关节病的一种。虽然它通常在青少年或年轻成人（通常是16-30岁之间）中发病，但并不意味着年龄较大的人群不会被诊断为该病。以下是一些专业的医学意见，帮助您更好地理解这一疾病及其诊断：

### 1. **年龄与强直性脊柱炎的发病**

- 强直性脊柱炎的确多见于青少年和年轻人，但也有部分患者在30岁以后首次出现症状，甚至在40岁以后才被诊断。虽然这种情况相对少见，但并非不可能。

- 如果您已经出现了相关症状（如慢性下背痛、晨僵、活动后缓解的疼痛等），年龄并不是排除诊断的决定性因素。

### 2. **症状和早期识别**

- 强直性脊柱炎的典型症状包括：

- 慢性下背痛，尤其是夜间加重；

- 早晨起床后僵硬感，需要活动后才能缓解；

- 疼痛可能会向臀部、大腿后侧放射；

- 关节肿胀或疼痛（如膝关节、脚踝等）。

- 如果您有这些症状，建议尽快就医，进行进一步的检查。

### 3. **诊断的关键**

- **影像学检查**：骶髂关节的X线或MRI是诊断强直性脊柱炎的重要依据，特别是早期可能需要通过MRI发现炎症性改变。

- **实验室检查**：HLA-B27基因检测在强直性脊柱炎患者中阳性率较高，但并非所有患者都携带此基因，且阳性也不一定意味着患病。

- **炎症指标**：如血液中的C反应蛋白（CRP）或红细胞沉降率（ESR）升高，可能提示炎症活动。

### 4. **年龄较大患者的特殊性**

- 年龄较大的患者可能因疾病进展缓慢或症状不典型而容易被误诊为其他疾病（如骨关节炎、腰椎间盘突出等）。

- 如果您有疑似症状，建议尽早与风湿科医生沟通，进行全面评估。

### 5. **健康建议**

- **保持积极的生活方式**：适当的锻炼（如游泳、瑜伽）有助于维持脊柱的灵活性，减轻疼痛。

- **关注心理健康**：慢性疾病可能会带来心理压力，保持积极心态对治疗有帮助。

- **遵医嘱治疗**：如果确诊为强直性脊柱炎，早期治疗（如非甾体抗炎药、免疫抑制剂或生物制剂）可以有效控制病情，延缓疾病进展。

- **定期复查**：与医生保持密切联系，定期监测疾病活动情况。

### 6. **与医生沟通**

- 请与您的主治医生详细讨论您的症状、检查结果以及治疗方案。如果有疑问，可以寻求第二意见，特别是专科风湿病医生的评估。

如果您有任何进一步的问题或需要更详细的指导，请随时告诉我，我将尽力为您解答！

1. **目前我疑诊为强直性脊柱炎，在确诊过程中我产生了如下疑惑这种病容易误诊成什么？请你作为一名专业的医务工作人员向我提供健康意见**

强直性脊柱炎（Ankylosing Spondylitis, AS）属于一种慢性炎性疾病，主要影响脊柱、骶髂关节以及全身其他部位，还可能伴有其他系统的炎症表现（如虹膜炎、关节炎等）。在确诊过程中，由于类似症状的疾病较多，该病的确诊确实容易受到一些因素干扰而发生误诊。以下是该病容易误诊的几类疾病，以及相关健康建议：

---

### **容易误诊的疾病**

1. **腰椎间盘突出症或其他退行性脊柱疾病：**

- **相似点：** 都可能伴有腰背部的疼痛或僵硬。

- **区别点：** 强直性脊柱炎通常表现为“炎性腰背痛”（夜间疼痛，晨起僵硬，活动后改善），而退行性疾病疼痛与活动往往关系不大。

2. **纤维肌痛综合征：**

- **相似点：** 会出现广泛的肌肉和骨骼疼痛。

- **区别点：** 强直性脊柱炎还会伴随炎性改变（如骶髂关节影像学异常），而纤维肌痛是一种功能性疼痛综合征，无特定的炎症标志。

3. **类风湿关节炎：**

- **相似点：** 都是自身免疫性的风湿病，早期可能有小关节炎症状。

- **区别点：** 强直性脊柱炎多以脊柱和骶髂关节疼痛为主，而类风湿关节炎以小关节受累为主（如手指、腕关节等），血清RF及CCP抗体检测能帮助区分。

4. **痛风或假性痛风：**

- **相似点：** 关节炎表现。

- **区别点：** 痛风多累及外周关节（如大脚趾），其痛风石及尿酸水平可帮助分辨。

5. **其他风湿性病（如银屑病关节炎、反应性关节炎）：**

- **相似点：** 关节炎的表现，部分患者可能与AS早期极为相似。

- **区别点：** 需要结合患者病史（如是否有皮肤表现或感染史）和影像学诊断。

---

### **健康建议**

1. **配合详细检查：**

- 通过影像学检查（如骶髂关节CT或MRI）确认骶髂关节炎症状。

- 血清学检查（如HLA-B27阳性率）对AS诊断有较高的参考价值。

2. **注意症状特征：**

- 如果出现长时间的夜间腰痛、晨起僵硬 (>30分钟)，且持续数月或见症状活动后缓解，这可能提示炎性腰痛。

3. **排查误诊风险：**

- 如果对诊断有疑问，可到拥有风湿免疫科的大型综合医院就诊，寻求专业的风湿病医生意见。

4. **保持良好的生活方式：**

- 定期锻炼（如游泳、瑜伽），改善脊柱的活动能力及缓解僵硬。

- 坚持正确的姿态，避免长期卧床。

- 多关注饮食均衡，多摄入富含钙和维生素D的食物，保护骨健康。

5. **心理调整与疾病管理：**

- 勇敢面对确诊和治疗，强直性脊柱炎在规范治疗下，许多患者可以保持良好的生活质量。

- 如果需要，可以加入患者支持团体交流经验。

---

### **下一步建议**

为了避免误诊和漏诊，建议您尽快寻找风湿免疫科专科医生进行全面评估和确诊。根据症状表现和检查，会根据指南制定适合您的治疗方案，例如非甾体抗炎药（NSAIDs）、生物制剂（抗TNF-α药物）等。切勿自行停药或治疗。

如有更多问题，请随时联系我，我会为您提供更多医学相关资讯！

1. **目前我疑诊为强直性脊柱炎，在确诊过程中我产生了如下疑惑几个医生说我不是这个病了，但是我还是觉得自己像，我该怎么办？请你作为一名专业的医务工作人员向我提供健康意见**

强直性脊柱炎（AS）是一种慢性炎症性疾病，其诊断可能较复杂，尤其是在早期症状不典型的情况下。你的疑虑是完全正常的，但为了保证诊断的准确性和科学性，以及避免过度担忧或延误治疗，以下是我作为专业健康工作者的一些建议：

---

### 1. **确认诊断：寻求专业风湿病医生的意见**

- **建议看专科医生**：如果还没有就诊风湿免疫科，尽量前往正规医院的风湿免疫科或其他权威临床机构。强直性脊柱炎的诊断通常由专业风湿科医生以详细病史、家族史、影像学检查（如X光、MRI）和实验室检查（如HLA-B27基因检测、炎性指标）综合得出。

- **接受全面检查**：如医生提出你可能不是强直性脊柱炎，建议详细记录下他们的依据，例如影像结果是否排除了骶髂关节炎，HLA-B27是否阴性等。

---

### 2. **关注症状并记录变化**

对于某些患者，早期症状可能类似于其他疾病（如机械性腰痛或纤维肌痛综合征）。你可以通过以下方式整理自己的健康状态：

- **记录疼痛特征**：

- 是否为夜间疼痛且无法缓解？

- 是否为晨僵持续30分钟以上？

- 运动是否缓解疼痛？

- **观察关节以外症状**：

- 是否有眼部炎症（如虹膜炎）？

- 是否伴随皮肤症状（如银屑病）？

- 是否出现消化症状（如腹泻或肠炎）？

通过这样的记录，你能提供医生更多依据，同时也帮助自己更清楚地了解病情。

---

### 3. **保持坦诚沟通并尊重医疗建议**

如果你对某位医生的诊断产生疑虑，可以进行以下选择：

- **咨询第二意见**：你可以寻找其他专业医生或医学中心进行“第二诊疗意见咨询”。

- **坦诚提出疑问**：在与医生沟通时，把你的疑惑、依据（如症状或者网络学习的诊断标准）有逻辑地讲出来，医生会更容易理解你的担忧并给出具体答复。

---

### 4. **不要排除其他疾病**

由于强直性脊柱炎的症状可能与某些其他疾病（如腰部椎间盘问题、纤维肌痛、类风湿性关节炎）相似，医生可能在为你排查的过程中否定AS的诊断。这意味着在现代医学中有时诊断过程需要更长时间，注意避免自行下结论。

---

### 5. **心理健康支持**

连续的疑诊过程可能会导致焦虑、害怕甚至抑郁情绪。如果你感到过度担忧，不妨：

- **寻求心理支持**：你可以向心理科专业人士倾诉你的情绪，缓解心理压力。

- **参与社区支持**：可以加入一些患者论坛，了解其他人确诊及管理病情的经验。但同时记得，这些仅供参考，医学决策始终要以医生为主。

---

### 总结

在疑诊阶段，冷静是最重要的。你可以选择以下步骤：

1. 到正规医院风湿免疫科接受进一步检查。

2. 记录症状，明确病情演变。

3. 寻求第二诊疗意见。

4. 注意健康生活方式（适度运动、健康饮食、规律作息），避免其他可能加重症状的因素。

强直性脊柱炎虽然是一种慢性疾病，但目前有良好的治疗方法（包括药物如生物制剂以及康复训练），其预后完全取决于早诊断、早治疗和长期管理。如果最终你确诊并积极治疗，是有可能保持良好的生活质量的。

如有进一步问题，请随时告诉我，祝你健康！

1. **目前我疑诊为强直性脊柱炎，在确诊过程中我产生了如下疑惑现在我还没确诊，但是我还是痛，我该吃什么药么？请你作为一名专业的医务工作人员向我提供健康意见**

作为一名专业健康顾问，我可以为您提供以下建议。需要注意的是，我的回答不能替代专业医生的诊疗建议，您在采取任何医疗措施前，请务必咨询您的主治医生或专业医疗团队。

### 1. **暂时用药的原则**

在尚未确诊为强直性脊柱炎（AS）时，如果疼痛已严重影响到您的生活质量，可以考虑在医生指导下使用一些缓解疼痛或消炎的药物。但同时以下几点是重要的：

- **非甾体抗炎药（NSAIDs）**：例如布洛芬、双氯芬酸钠等，通常是强直性脊柱炎缓解疼痛和炎症的第一选择。如果症状较轻，可以短期使用，观察症状是否缓解。

- **避免长期用药**：在尚未明确诊断前，不建议长期自行使用消炎止痛药，以避免掩盖症状。

- **谨慎对待激素类药物**：避免自行使用激素类药物，它们通常不是治疗强直性脊柱炎的主要选择，且长期使用会带来副作用。

### 2. **生活方式上的调整**

- **热敷缓解不适**：疼痛部位可以采用温热敷的方式来帮助放松肌肉，缓解疼痛。

- **适当运动**：如果疼痛允许，可以做一些轻柔的伸展和背部锻炼，以防止关节僵硬（在专业理疗师或医生的指导下进行）。

- **注意保护脊柱**：避免长时间弯腰、久坐等不良姿势。

### 3. **确认诊断的重要性**

强直性脊柱炎是一种慢性、免疫系统相关的全身性炎性疾病，如果确诊，治疗方案可能包括：

- **疾病改善的抗风湿药（DMARDs）**：如柳氮磺吡啶。

- **生物制剂**：如抗TNF-α药物（依那西普、阿达木单抗等），对控制炎症和减少病情进展效果显著。

- **康复调整**：包括理疗、运动指导和矫正姿势等。

在确诊过程中的阶段，不建议随意加大用药或尝试不熟悉的药物。准确的诊断结果非常重要，以便医生为您制定最佳的治疗方案。

### 4. **与医生保持良好沟通**

您目前的症状和疑似诊断情况需要：

- 详细的临床评估（如症状、体格检查）。

- 相关血液检查（如HLA-B27基因检测）。

- 影像学检查（如X光、MRI）确认是否有骶髂关节炎或其他病变。

请尽快与风湿免疫科或相关专科医生联系，尽早确认诊断并开始对症治疗。

### 温馨提示

由于我提供的建议无法涵盖个体化医疗需求，请勿完全依赖此信息判断自身健康状况。在症状加重时，请立即联系医疗机构。

1. **目前我疑诊为强直性脊柱炎，在确诊过程中我产生了如下疑惑一次CT检查大概要会吸收多少剂量的辐射？对健康影响大么？请你作为一名专业的医务工作人员向我提供健康意见**

作为一名模拟的健康助手，我可以为您提供专业的答疑和指导。首先，希望您保持冷静，针对疑诊的强直性脊柱炎（AS）积极进行诊断和治疗。关于CT检查和辐射剂量的问题，这里是详细说明：

---

### **一、CT检查的辐射剂量**

1. **CT辐射剂量的范围：**

CT扫描的辐射剂量根据扫描的部位和设备不同有所差异。

- *一般的胸部或腹部CT：* 一次CT检查的有效剂量约为 **5-10毫西弗（mSv）**。

- *脊柱CT：* 通常在 **6-15毫西弗（mSv）** 之间，具体剂量依赖于扫描设备的类型和标准。

> 举例：一次脊柱CT可能相当于自然背景辐射1至2年的吸收量。

2. **辐射与风险的比较：**

- 一次CT相关的辐射剂量通常很小，非累积暴露情况下对人体的健康风险非常低。

- 为了便于感知风险，CT检查的辐射剂量与自然背景辐射量或一些日常暴露（如长距离飞行）相近，远低于职业性辐射工人的安全限。

---

### **二、健康影响与安全性**

- **对健康的潜在影响：**

- 低剂量辐射（如一次CT）的健康影响被认为是极小的，正常人体有修复辐射损伤的能力。

- 只有长期多次、高剂量辐射暴露（如核电厂事故或大量放疗）才可能会显著增加患癌症的风险。

- **您无需过度担忧：**

医院在进行CT检查时会严格控制辐射剂量，确保检查的安全性。同时，CT的诊断价值往往远远高于潜在的风险，特别是当用于明确诊断时。

---

### **三、建议与注意事项**

1. **与医生沟通：**

- 在进行CT检查前，您可以详细与医生沟通，了解检查的必要性。

- 如果担心辐射问题，医生可能会考虑使用低辐射剂量模式或替代的检查方法（如MRI或超声）。

2. **避免不必要的重复检查：**

- 若CT检查已经提供足够信息，应避免短期内再次进行其它类似辐射检查。

- 保留您的以往影像资料（如CD或报告），递交给医生供诊断参考。

3. **保持均衡的生活方式，帮助减少辐射可能的影响：**

- **饮食：** 增加抗氧化剂（如新鲜蔬菜、水果）的摄入；适量补充维生素C和维生素E。

- **运动：** 维持身体健康有助于提升细胞的自我修复能力。

---

### **特别提醒**

强直性脊柱炎是一种慢性炎性疾病，尽管需要积极确诊和治疗，但长期管理同样十分重要。与诊疗团队保持紧密联系，科学决策检查和治疗方案，能帮助更好地控制病情。

如果仍有疑问，欢迎您继续咨询！

1. **目前我疑诊为强直性脊柱炎，在确诊过程中我产生了如下疑惑我HLA-B27是阳性，抽血化验检查也有很多指标异常，症状也符合强直性脊柱炎，为什么医生说不能诊断强直呢？请你作为一名专业的医务工作人员向我提供健康意见**

强直性脊柱炎(AS)的确诊可能是一个复杂的过程，尽管HLA-B27阳性和其他异常指标及症状为支持性证据，但医学诊断是基于多方面的综合评估。以下是可能导致暂时无法明确诊断的几个原因，以及相关健康建议：

### 1. **HLA-B27阳性不是AS诊断的唯一依据**

- **HLA-B27只是一个易感基因**。

虽然大部分强直性脊柱炎患者HLA-B27阳性，但并非所有HLA-B27阳性的人都会患病。尤其是在普通人群中，少部分HLA-B27阳性的人可能一生都不会发病。

- 所以，仅仅依靠HLA-B27阳性还不足以确诊强直，医生还需要结合其他诊断标准。

---

### 2. **临床症状与影像学表现的结合**

强直性脊柱炎的诊断更多依赖以下几个方面：

**(1)** 临床表现（如腰背痛、晨僵、活动后好转等）。

**(2)** 放射学和MRI检查的结果，尤其是骶髂关节的炎症或结构变化。

如果您在影像学或症状表现方面尚未达到明确诊断标准，医生可能暂时无法确诊。

---

### 3. **其他疾病可能有类似的表现**

有些其他疾病，如反应性关节炎、银屑病性关节炎、肠病相关性关节炎等，也可能表现出类似强直性脊柱炎的症状和检查异常。因此，医生可能需要排除其他疾病后，才能最终确诊。

---

### 4. **疾病的早期阶段诊断困难**

有些患者在疾病早期仅有轻微的不适或检查指标异常，但尚未发展到明确的强直性脊柱炎阶段。在这种情况下，医生可能会采取观察随访的方式，以判断疾病的进展情况。

---

### 健康建议

1. **继续配合医生进一步的检查与随访**

医生可能需要您定期复查血液、影像学检查（比如骶髂关节的MRI），以及观察症状的变化。这是为了在疾病的早期阶段及时干预。

2. **注重早期治疗和管理**

如果怀疑强直性脊柱炎，早期治疗可以延缓疾病的进展，即便尚未明确确诊，也可以在医生指导下采取一定的健康管理措施，如抗炎药物、康复运动等。

3. **生活方式调整**

- **坚持适量运动**：如游泳、瑜伽或适度的伸展运动，有助于保持关节灵活性和改善症状。

- **良好的体姿习惯**：避免长时间固定姿势，注意避免重体力劳动。

- **保持体重正常范围**：过重可能增加脊柱和关节的负担。

4. **关注心理健康**

诊断过程中的不确定性可能对心理造成一定压力和焦虑，建议与医生、家人沟通，同时保持积极的心态。

5. **及时复查**

强直性脊柱炎可能具有慢性进展性，所以定期复诊和血液、影像学检查很重要，这帮助识别病情变化。

---

如果您仍有疑问，建议直接与治疗您的风湿免疫科医生沟通，获取更详细的个性化医疗建议，同时可以寻求第二专家意见。如果有家族史等风险因素，也可以重点向医生说明，进一步帮助诊断决策。

**三、确定诊断后的治疗与管理 （在这部分问题前，请加入“目前我已确诊为强直性脊柱炎，在后续治疗过程中我产生了如下疑惑，请你作为一名专业的医务工作人员向我提供健康意见”，每个问题请分别询问，避免“AI记忆”对回答造成干扰）**

1. **目前我已确诊为强直性脊柱炎，在后续治疗过程中我产生了如下疑惑:我最后会不会残疾？请你作为一名专业的医务工作人员向我提供健康意见**

作为一名专业的健康顾问，我理解确诊强直性脊柱炎（AS）可能会让人感到焦虑和疑惑。我要首先强调的是，每位患者的病程与治疗效果可能存在差异，但通过及时诊断和有效治疗，大多数强直性脊柱炎患者可以保持较高的生活质量并避免残疾。

以下是关于强直性脊柱炎的核心信息以及我对你的健康建议：

### 强直性脊柱炎是否会导致残疾？

1. **病情的自然进展**：

- 如果不进行治疗，AS可能导致脊柱的逐渐僵硬，严重时会导致驼背（脊柱前屈畸形）。

- 在少部分病例中，关节外症状（如髋关节、膝关节受累）可能引起活动受限。

- 然而，早期识别和干预可以显著减少病情进展带来的问题。

2. **接受治疗后的预后**：

- 现代医学已经有了很大进步，特别是生物制剂和靶向药物的应用，可以很好地控制炎症，减缓疾病进程。

- 积极的物理治疗和日常锻炼可有效保持关节的灵活性，降低畸形和功能丧失的风险。

3. **风险因素**：

- 病情持续未控制（如不服药或延误治疗）和不规律的生活习惯可能加重疾病负担。

- 吸烟、长期熬夜等不健康因素也会加速病情恶化。

### 健康建议

1. **接受规范治疗**：

- 按照医生建议坚持药物治疗，包括非甾体抗炎药（NSAIDs）、抗风湿药和生物制剂等。

- 定期随访和检查（如影像学和炎症指标评估），了解自身病程发展。

2. **保持身体活动**：

- 强调核心肌群锻炼和每日做延展性运动（如瑜伽、游泳等），这有助于保持脊柱灵活性。

- 避免长期固定不动，可尝试在日常工作和生活中加入适量活动。

3. **科学饮食和健康生活方式**：

- 尽量选择均衡饮食，多摄取抗炎食物（如富含欧米伽-3脂肪酸、蔬菜水果等）。

- 避免吸烟和酗酒，这些均与疾病加重相关。

- 保持规律作息，减轻精神压力。

4. **心理健康管理**：

- 如果感到情绪低落或焦虑，不要犹豫寻求心理支持，与家人和朋友分享感受。

- 加入患者支持群体，与其他AS患者交流经验，可能有所帮助。

5. **预防畸形进展**：

- 选择合适的床垫和枕头，尽量保持脊柱的自然形态，避免不良姿势。

- 学会和纠正日常体态问题，例如站立时挺胸抬头、坐直等。

### 总结

如果你积极配合治疗，并注重生活方式干预，绝大多数情况下强直性脊柱炎患者可以避免严重残疾。关键在于早期干预、定期随访、调整生活方式。

如有进一步具体问题（如药物副作用、锻炼方法等），可以随时咨询我或者你的主治医生。我祝愿你在未来的治疗和生活中保有信心，并获得良好的健康结果！

1. **目前我已确诊为强直性脊柱炎，在后续治疗过程中我产生了如下疑惑:我的治疗需要吃多久药？请你作为一名专业的医务工作人员向我提供健康意见**

强直性脊柱炎（Ankylosing Spondylitis, AS）是一种慢性自身免疫性疾病。目前无法完全治愈，但通过规范的治疗和管理，可以有效控制症状、延缓病情进展并提高生活质量。关于“需要吃多久药”的问题，以下是一些专业建议：

### 1. **长期治疗的必要性**

- 强直性脊柱炎通常需要**长期甚至终生治疗**。这是因为该病属于慢性炎症性疾病，一旦停药可能导致症状复发或疾病进展，增加关节畸形、活动受限等风险。

- 治疗目标是减少炎症、缓解疼痛、防止脊柱僵硬及关节损害，提高患者的生活质量。

### 2. **药物治疗的时间长短取决于以下因素：**

- **病情的严重程度**：早期轻度患者可能需短期使用药物来控制症状，同时结合康复锻炼。若病情较重或已经出现不可逆的关节损害，药物治疗需要长期维持。

- **治疗反应**：通过观察药物的效果和副作用，医生会根据症状、影像学（如X光、核磁共振）或炎症指标来调整用药时间。

- **停药标准**：一些患者在病情完全缓解（症状消失且无影像学进展）后，可考虑在医生指导下减少药物剂量或试行停药，但需谨慎。

### 3. **常见药物的用药建议**

- **NSAIDs（非甾体抗炎药）**：如布洛芬、塞来昔布等，通常是强直性脊柱炎的一线药物，用于缓解疼痛和炎症。根据症状可以间断使用或连续服用。

- **DMARDs（改善病情的抗风湿药物）**：如甲氨蝶呤，对外周关节受累有效，通常需长期使用。

- **生物制剂**：如TNF-α抑制剂（依那西普、阿达木单抗等）、IL-17抑制剂（司库奇尤单抗），对严重患者效果显著，需考虑长期维持治疗。

- **糖皮质激素**：多用于急性发作期，一般短期使用，避免长期副作用。

### 4. **其他建议**

- **定期复诊**：至少每3-6个月复查一次，评估病情控制情况并调整治疗方案。

- **遵循个体化治疗原则**：每个患者病情不同，治疗方案会因人而异。严格按照风湿免疫科医生的建议进行用药，不可擅自减少或停药。

- **结合功能锻炼**：药物控制症状的同时，结合合理的康复运动（如拉伸运动、游泳、瑜伽等）可帮助改善脊柱和关节的灵活性，延缓僵硬。

### 5. **关于长期药物的可能风险**

长期服用药物可能带来一些副作用，比如胃肠道问题（NSAIDs）、肝肾功能受损（生物制剂）等，因此需要定期体检（如血常规、肝肾功能、影像检查等）以确保安全。

### 6. **心理支持**

再强调一点，强直性脊柱炎的治疗是一个长期过程。保持良好的心理状态、与医生保持沟通并积极应对非常重要。

希望这些建议对您有所帮助！若有更多疑问，建议咨询您的主治医生以获得最准确的指导。

1. **目前我已确诊为强直性脊柱炎，在后续治疗过程中我产生了如下疑惑:服药治疗期间我可以打疫苗么？请你作为一名专业的医务工作人员向我提供健康意见**

作为一名确诊为强直性脊柱炎的患者，在接受治疗期间是否可以接种疫苗，需要根据治疗方案、所用药物类型，以及疫苗种类等多方面综合考虑。以下为一些专业的健康建议：

### 1. **了解药物的影响**

强直性脊柱炎的常用治疗方法包括非甾体抗炎药（NSAIDs）、免疫抑制剂（如甲氨蝶呤）、以及生物制剂（如TNF-α抑制剂、IL-17抑制剂）。

- **非甾体抗炎药（NSAIDs）**：通常不会对疫苗接种产生显著影响，可以正常接种。

- **免疫抑制剂和生物制剂**：这些药物可能会降低免疫系统的反应力。对于这类药物的使用患者，需要特别关注疫苗类型（活疫苗或灭活疫苗），因为某些活疫苗可能会引发安全问题。

### 2. **疫苗种类的选择**

- **灭活疫苗**（如流感疫苗、乙肝疫苗、新冠灭活疫苗等）：一般是安全的，即使在使用免疫抑制剂的情况下也适宜接种。

- **活疫苗**（如麻腮风疫苗、带状疱疹疫苗、黄热病疫苗等）：通常不建议在免疫功能受到抑制的情况下接种，因为可能导致疫苗病原的异常增殖。应在停药一定时间后再接种，具体停药及接种时机需要咨询主治医师。

### 3. **接种疫苗的时机**

- 在病情稳定且症状可控时接种疫苗，以减少接种疫苗可能对身体的额外负担。

- 如果正在使用免疫抑制剂或生物制剂，应视情况避开用药的“效果最强”时段（比如用药前后几天）接种疫苗。

- **季节性疫苗**（如流感疫苗）建议根据流行期提前规划接种时间。

### 4. **与医生沟通**

每个患者的身体状况和病情不同，建议在接种疫苗前咨询风湿科医生或免疫专科医生，提供以下信息以帮助医生决策：

- 当前使用的药物及治疗方案。

- 接种疫苗的必要性和时机。

- 过往的疫苗接种和过敏史。

### 5. **监测反应**

接种疫苗后，注意观察身体反应，一旦出现异常（如过敏反应、持续的高热等），及时就医。

**总结：** 强直性脊柱炎患者在服药期间是否可以接种疫苗需视具体情况而定，大部分灭活疫苗是安全的，但活疫苗需要特别小心，建议您在接种疫苗前咨询您的主治医生，以保障安全和效果。

1. **目前我已确诊为强直性脊柱炎，在后续治疗过程中我产生了如下疑惑:“抗炎药”、“消炎药”和“止疼药”有什么区别？请你作为一名专业的医务工作人员向我提供健康意见**

作为一名专业医务顾问，我很理解您的疑惑。以下是“抗炎药”、“消炎药”和“止疼药”的区别，以及它们在强直性脊柱炎治疗中的应用和建议：

### 1. **抗炎药**

- **定义**：抗炎药是指能够抑制机体炎症反应的药物，包括非甾体抗炎药（NSAIDs）、糖皮质激素，以及一些生物制剂（如TNF抑制剂等）。

- **作用机制**：通过抑制炎症介质的释放或作用，从而减轻炎症反应。

- **适用情况**：

- 强直性脊柱炎是一种以慢性炎症为主要病理机制的疾病，抗炎药通常是治疗的基础药物。

- 非甾体抗炎药（如布洛芬、双氯芬酸）可以缓解炎症和疼痛。

- 如果症状严重，生物制剂（如阿达木单抗）可能会被推荐。

### 2. **消炎药**

- **定义**：消炎药是一种更广义的概念，泛指能够缓解或消除炎症的药物，实际上也包括抗炎药在内。

- **适用范围**：

- 消炎药不仅包含用于系统性炎症的抗炎药，也包含用于局部感染或炎症的药物（如抗生素）。

- 在强直性脊柱炎中，细菌感染通常不属于主要病因，因此抗生素类药物使用较少。

### 3. **止疼药**

- **定义**：止疼药是专门用于缓解或消除疼痛的药物，包括非处方药（如对乙酰氨基酚）和处方药（如阿片类药物）。

- **作用机制**：

- 阻断神经系统对疼痛信号的传递，或减少大脑对疼痛的感知。

- 与抗炎药不同，止疼药通常不直接作用于炎症本身，而是缓解痛感。

- **适用情况**：

- 如果强直性脊柱炎的疼痛症状较为明显，但炎症并不严重，医生可能会单独使用止疼药。

- 注意不要长期依赖止疼药，因为过度使用可能导致耐药性或副作用。

---

### 强直性脊柱炎治疗中的应用建议

1. **首选抗炎药物**：

- 非甾体抗炎药（NSAIDs）是治疗强直性脊柱炎的基础，既能缓解疼痛，也能控制疾病活动。

- 如果NSAIDs效果不佳，可根据医生建议使用生物制剂或靶向合成药物。

2. **止疼药的辅助使用**：

- 在严重疼痛时，短期使用止疼药可以快速缓解症状，但必须遵守医生的剂量建议。

3. **避免误区**：

- **抗炎药 ≠ 消炎药 ≠ 止疼药**：虽然它们目标相似，但具体作用不同，应根据病情需要合理选择。

- 切勿自行调整药物剂量或频率，长期使用药物需定期监测可能的副作用（如胃肠道损伤或肾功能问题）。

---

总之，如果您对于药物的使用仍然感到困惑，建议您及时与风湿免疫科医生沟通，并定期复查疾病的活动情况。同时，您可以结合物理治疗、运动疗法等非药物手段，控制病情进展，改善生活质量。

1. **目前我已确诊为强直性脊柱炎，在后续治疗过程中我产生了如下疑惑:吃了止疼药之后我还是疼，能再吃一片么？请你作为一名专业的医务工作人员向我提供健康意见**

作为一名专业的健康助手，我可以为您提供以下建议，但请注意，这并不能代替您的医生的具体建议。如果您在服用止痛药后仍然感到疼痛，请谨慎处理，以下是一些供您参考的建议：

### 1. **遵守医嘱**

- 请严格按照医生的处方服用止痛药（如布洛芬、对乙酰氨基酚或其他药物）。如果医生已经明确指出每日最大剂量，请不要随意加量。

- 有些止痛药如果服用过多，可能会引起严重的副作用，例如胃肠道出血、肝肾损伤等。

### 2. **不要轻易加量**

- 如果目前的药物剂量并未完全缓解疼痛，不建议擅自增加剂量。因为这可能带来更大的副作用风险。

- 建议在疼痛未缓解的情况下，联系您的医生或风湿科专科医生，寻求调整治疗方案的建议。

### 3. **记录疼痛**

- 记录疼痛的时间、强度及触发因素，并将这些信息提供给医生。这些信息有助于您和医生更好地评估是否需要调整药物剂量或选择其他治疗方案。

### 4. **寻求其他缓解疼痛的方法**

- 在医生指导下，可以尝试物理疗法、热敷、牵伸运动等非药物疗法来辅助止痛。

- 适当的休息和避免过度劳累是管理疼痛的重要一环。

### 5. **潜在的治疗调整**

- 如果常规止痛药效果不佳或负担较重，可以与医生讨论是否需要引入其他治疗选择，例如改善病情的药物（DMARDs）、生物制剂或肾上腺皮质激素。

### 6. **紧急情形**

- 如果疼痛突然加剧，表现出其它异常症状（如肿胀、发热、呼吸困难等），请立即就医。

必须强调，您的医生是最了解您病情的专业人士，建议您在症状未缓解时随时联系他们调整治疗方案或获取专业指导。

1. **目前我已确诊为强直性脊柱炎，在后续治疗过程中我产生了如下疑惑:吃完药之后我感觉我的尿很黄，是副作用么？请你作为一名专业的医务工作人员向我提供健康意见**

强直性脊柱炎（AS）的治疗通常涉及药物治疗，包括非甾体抗炎药（NSAIDs）、免疫抑制剂或生物制剂。有时候，服药后尿液颜色的改变（如尿发黄）可能引起担忧。在此我给出以下专业的健康建议：

1. **尿液发黄的常见原因**：

- 多数情况下，尿液发黄是较为正常的现象，可能与体内水分不足引起尿液浓缩有关。如果饮水量较少，尿液颜色通常会加深。

- 某些药物和维生素（特别是维生素B族）可能使尿液呈现深黄色，但这通常是无害的副作用。

- 某些药物在体内代谢后可能通过尿液排出，也可能会改变尿液的颜色。例如，磺胺类药物或一些特定的抗生素可能引起尿液颜色变化。

2. **需要注意的药物副作用**：

- 尿液颜色异常有时可能提示药物对肝肾功能的影响。例如，如果伴随以下症状，应及时就医：

- 持续尿色深黄甚至发棕。

- 泡沫尿（提示蛋白尿）。

- 出现皮肤或眼白发黄（黄疸）。

- 腰部疼痛、乏力、恶心或食欲减退。

- 在服用免疫抑制剂或生物制剂时，定期进行肝肾功能监测是必要的，以确保安全用药。

3. **处理建议**：

- **增加饮水量**：保持每日足量饮水（通常建议1.5-2升），观察尿液颜色是否有所改善。

- **药物回顾**：与主治医生或药师沟通，确认是否有使用某些药物或补充剂可能导致尿液颜色改变。

- **定期随访**：尤其是强直性脊柱炎患者，应定期复查肝肾功能、炎症指标和其他相关监测项目。

- **存疑时就医**：如果尿液颜色变化伴随其他不适症状，或持续时间较长，应立即就医，排查是否存在严重副作用或其他健康问题。

如果需要更具体的解释或者您有详细的用药清单，可以提供详细信息，我可以为您进一步解答。如果担忧，请尽早与您的主治医生沟通，确保治疗的安全和效果。

1. **目前我已确诊为强直性脊柱炎，在后续治疗过程中我产生了如下疑惑:听说生物制剂疗效很好，什么是生物制剂？请你作为一名专业的医务工作人员向我提供健康意见**

强直性脊柱炎（AS）是一种慢性炎症性疾病，多数与免疫系统异常活跃有关，可能会导致脊柱及其他关节疼痛、僵硬，甚至影响身体其他部位的器官。你提到的**生物制剂**是一类近年来在治疗强直性脊柱炎等自身免疫性疾病中广泛应用的新型药物，以下是关于生物制剂的详细科普与健康建议：

---

### **什么是生物制剂？**

1. **定义：**

- 生物制剂是一类从生物体内提取或利用基因工程技术制备的药物，其主要作用是通过靶向干预免疫系统中关键的炎症因子，达到减轻炎症反应、控制病情的目的。

- 常见的生物制剂多为针对 **TNF-α（肿瘤坏死因子α）** 或 **IL-17（白介素17）** 的抑制剂，这些炎症因子在强直性脊柱炎中起主要作用。

2. **主要类型：**

- **TNF-α抑制剂：** 如依那西普（Enbrel）、阿达木单抗（Humira）。

- **IL-17抑制剂：** 如司库奇尤单抗（Cosentyx）。

- 其他新型药物类别也在不断研发和临床应用中。

---

### **生物制剂的优点**

- **快速缓解症状：** 通常相比传统药物（如非甾体抗炎药NSAIDs、免疫抑制剂），生物制剂能更快缓解病症如背部疼痛、晨僵。

- **保护关节及功能：** 延缓脊柱和关节损伤，降低长期致残的风险。

- **较少副作用：** 与传统免疫抑制剂相比，对全身免疫系统的抑制作用较小，更具靶向性。

- **改善生活质量：** 很多患者在使用生物制剂后，可恢复一定程度的运动功能并显著提升生活质量。

---

3.### **适应人群**

. 当患者对传统治疗（例如NSAIDs或慢作用抗风湿药物）效果不佳时，经医生评估可以使用生物制剂。

. 患者具备明显的脊柱炎症活动体征，并伴影像学证据（如MRI显示脊柱或骶髂关节病变）。

. 腰椎或骶髂关节长期疼痛、僵硬，影响日常生活。

. 同时存在系统性炎症表现（如眼炎、肠炎）。

---

4.### **使用注意事项**

. **评估感染风险：** 生物制剂会影响免疫系统，可能增加感染风险，尤其是结核和肝炎等。如果准备使用生物制剂，必须在治疗前筛查潜在感染并根据医生建议接受预防性治疗。

. **治疗持续性：** 生物制剂非短期疗效药，一定需要遵医嘱长期、规律使用，忌随意停药。

. **定期随访：** 使用期间需定期复诊，包括血液检查和影像检查，以评估病情变化及药物副作用。

. **经济因素：** 生物制剂对部分患者来说费用较高，建议了解相关医疗保障政策或咨询医生是否有合适的替代方案。

---

5.### **友情提示与健康建议**

. **综合治疗：** 生物制剂不是治愈方法，而是控制病情的重要手段之一，联合其他治疗（如物理治疗、健康管理）效果更佳。

. **生活方式调整：**

- 规律锻炼，特别是针对脊柱的康复训练，可帮助维持脊柱灵活性。

- 保持正确姿势，避免久坐和长期重体力劳动。

- 关注饮食营养，保持健康体重，避免加重关节负担。

. **心理支持：** 如果长期治疗对心理带来困扰，不妨寻求心理辅导帮助，维持积极心态。

---

**最后，强直性脊柱炎的治疗是一个长期过程，建议你在专业风湿免疫科医生的指导下制定个体化治疗计划。如有进一步疑问，可以及时咨询你的主治医师。我祝愿你早日通过科学的治疗和良好的生活管理，拥有更好的健康状态！**

1. **目前我已确诊为强直性脊柱炎，在后续治疗过程中我产生了如下疑惑:生物制剂副作用大么？请你作为一名专业的医务工作人员向我提供健康意见**

很高兴能够为您提供帮助！作为一名确诊为强直性脊柱炎（AS）的患者，了解生物制剂的作用和可能的副作用是十分重要的。针对您的疑问，我将尽量以清晰、专业的方式进行解答。

### 1. **生物制剂在强直性脊柱炎中的作用**

生物制剂是一类针对特定免疫系统机制的药物。强直性脊柱炎是一种自身免疫性疾病，可能与炎症因子如肿瘤坏死因子（TNF）和白介素-17（IL-17）的过度活跃有关。生物制剂通过抑制这些因子，减轻炎症反应，从而控制病情，缓解症状，预防疾病进展。常用的生物制剂包括：

- **抗TNF-α药物**：如阿达木单抗（Adalimumab）和依那西普（Etanercept）。

- **IL-17抑制剂**：如司库奇尤单抗（Secukinumab）。

### 2. **生物制剂的副作用**

生物制剂虽效果显著，但确实可能引发一定的副作用，具体包括以下几个方面：

- **免疫抑制相关**：

- 生物制剂可能使免疫系统被抑制，从而增加感染的风险，如呼吸道感染、结核病或真菌感染。部分患者在治疗前会进行结核筛查，以防止潜在感染。

- **局部反应**：

- 有些患者在注射部位可能出现皮肤红肿、疼痛等轻微不适，但通常是短暂的。

- **过敏反应**：

- 极少数患者可能对药物本身出现过敏反应，如皮疹、瘙痒甚至严重的过敏性休克。

- **罕见严重副作用**：

- 生物制剂长期使用可能与某些罕见疾病（如肿瘤、淋巴瘤）的风险增加相关，但总体风险较低。

### 3. **如何降低副作用风险？**

为了使治疗更加安全，建议采取以下措施：

- **严格遵循医生建议**：

- 按照之前的医疗方案来正确使用生物制剂，避免私自更改剂量或停药。

- **感染前筛查及监测**：

- 在开始生物制剂前应进行结核病及乙肝等传染性疾病筛查；治疗期间进行定期血液和健康监测以发现潜在问题。

- **健康生活方式**：

- 坚持良好的生活习惯，如均衡饮食、适量运动及规律作息，提高身体的免疫力基础。

### 4. **个性化治疗与医生沟通**

每个患者对生物制剂的耐受性不同，有些患者可能只出现轻微的不良反应，而有些患者可能完全没有副作用。因此，在整个治疗过程中，保持与主治医生的密切沟通。如果出现任何不适或副作用，及时告知医生并调整治疗方案。

### 总结

生物制剂在强直性脊柱炎的治疗中是非常重要的一类药物，其副作用是可控的。一旦严格按照医生的建议使用，并做好定期筛查和监测，其益处通常远大于风险。然而，治疗方案的选择应该是个性化的，建议您与专业的风湿免疫科医生保持密切联系，共同制定最适合的治疗方案。

祝您病情稳定，健康生活！如果有更多疑问，欢迎继续提问！

1. **目前我已确诊为强直性脊柱炎，在后续治疗过程中我产生了如下疑惑:生物制剂会成瘾么？请你作为一名专业的医务工作人员向我提供健康意见**

强直性脊柱炎属于自身免疫性疾病的一种，目前多数治疗策略集中在控制炎症和减轻症状，生物制剂（如TNF-α抑制剂、IL-17抑制剂等）是一种常见且有效的治疗方式。对于“生物制剂是否会成瘾”，我从医学角度向您解答如下：

### 1. **生物制剂不会成瘾**

生物制剂的作用机制是通过针对免疫系统特定部分（如炎性因子）的靶向调控，抑制炎症反应，从而控制疾病进展。这种机制与成瘾药物（如麻醉剂）作用大脑奖赏系统完全不同。因此，生物制剂并不会引起“成瘾”或依赖行为。

### 2. **长期使用的必要性**

生物制剂的使用往往是一个长期治疗的过程，其目的是为了持续控制炎症和预防疾病复发，避免关节和脊柱的不可逆损伤。这不是“成瘾”，而是疾病管理的需要。不过，具体用药方案需要医生根据您的病情情况个性化调整。

### 3. **正确关注长期治疗的可能副作用**

虽然不会成瘾，但长期使用生物制剂可能会带来一些副作用，如感染风险增加（因为免疫抑制），或局部注射不适等。您需要定期随访，与医生共同监测治疗效果和可能的副作用。

4.### 建议：

. **与医生保持良好沟通**：遵从医嘱，定期复诊，了解每个药物的风险和收益。

. **不随意停药**：自行停药可能导致炎症反弹，甚至病情加重。

. **健康生活**：适当锻炼（物理治疗）、保持良好的生活习惯有助于整体病情的控制。

. **谨慎对待网络或非专业建议**：如果对治疗方案有疑虑，应当及时向您的主治医生请教。

如果有其他相关问题，随时欢迎您沟通，我愿意为您提供更多医学建议！

1. **目前我已确诊为强直性脊柱炎，在后续治疗过程中我产生了如下疑惑:我如果有乙肝（结核/其它感染），不可以用生物制剂了的话该怎么办？请你作为一名专业的医务工作人员向我提供健康意见**

强直性脊柱炎（AS）是一种自身免疫性疾病，生物制剂（如TNF-α抑制剂）是治疗强直性脊柱炎的重要手段之一。然而，如果患者同时存在乙肝、结核或其他感染，使用生物制剂的风险会显著增加。因此，在这种情况下，需要非常谨慎地选择治疗方案。

以下是一些健康建议：

### 1. **明确感染情况**

- **乙肝感染：**

- 需要评估乙肝的具体状态，是**携带状态**、**慢性乙型肝炎**，还是已经**病毒复制活跃**。这可以通过检测乙肝表面抗原（HBsAg）、乙肝病毒DNA载量（HBV-DNA）以及肝功能指标（ALT、AST）来确认。

- **结核感染：**

- 使用生物制剂前，医生通常会进行结核筛查，包括胸部X光片和结核感染的皮肤试验/血液试验。如果发现有潜在结核感染，需要在开始生物制剂治疗前进行抗结核预防治疗。

- **其他感染：**

- 如果存在其他感染（如真菌感染或活动性细菌感染），需首先控制感染。

---

### 2. **替代治疗方案**

- 如果明确不适合使用生物制剂，可以尝试其他非生物制剂的药物治疗：

- **非甾体抗炎药（NSAIDs）：** 仍然是治疗AS的第一线药物，能够有效缓解疼痛和炎症。

- **柳氮磺吡啶（SSZ）：** 对于伴有周边关节受累的患者可能有一定效果。

- **免疫抑制剂（如甲氨蝶呤）：** 如果同时有周围关节炎，甲氨蝶呤可能是一种选项。

- 在某些情况下，可与风湿病科医生共同探讨个性化治疗方案。

---

### 3. **联合感染治疗与监控**

- **乙肝合并感染：**

- 在严格监测下，可以联合抗病毒治疗（如恩替卡韦或替诺福韦），降低HBV复发风险，并在整体评估下有选择性地使用生物制剂。

- **结核合并感染：**

- 在结核潜伏感染者中，先接受6-9个月的结核化学预防治疗后，再酌情考虑使用生物制剂。

- **定期监测：**

- 治疗过程中需密切监测肝功能、病毒载量（HBV-DNA），以及感染状态。

---

### 4. **多学科协作**

- 这种复杂的情况需要风湿科、感染科、甚至肝病科医生的共同参与，确保治疗的安全性与有效性。

- 定期随访是管理这一疾病的重要环节。

---

### 5. **生活方式管理**

- 避免吸烟和过量饮酒，以减轻身体负担。

- 适度运动（如瑜伽、游泳）帮助维持脊柱和关节的灵活性。

- 保持充足的营养摄入，增强免疫功能。

---

总的来说，虽然感染问题会对生物制剂的使用产生限制，但并非无法治疗。在感染得到良好控制或找到替代方案的情况下，依然可以有效管理强直性脊柱炎的症状。请根据自身的具体情况，与医生讨论最佳治疗方案。

1. **目前我已确诊为强直性脊柱炎，在后续治疗过程中我产生了如下疑惑:用了生物制剂还用吃其它药么？请你作为一名专业的医务工作人员向我提供健康意见**

作为一名专业的医务工作人员，我先向您说明，强直性脊柱炎（AS）的治疗是一个综合管理的过程，使用生物制剂（如TNF-α抑制剂或IL-17抑制剂）是其中一个重要选项，但往往会根据患者的具体病情决定是否需要同步使用其他药物或疗法。

以下是一些建议，供您参考：

### 1. **生物制剂的作用**

生物制剂主要用于控制炎症，减轻症状，延缓疾病进展，特别适合中重度和对传统治疗无效的患者。

- 如果使用生物制剂，可能不需要一部分传统抗风湿药（如柳氮磺胺吡啶等）。

- 但治疗通常是个性化的，医生会根据患者的疾病活动程度与身体状况综合考虑是否需要联合使用其他药物。

### 2. **是否需要其他药物？**

具体情况可能包括以下情形：

- **非甾体抗炎药（NSAIDs）**：如洛索洛芬、依托考昔等，通常作为强直性脊柱炎的一线药物，用于缓解疼痛和控制炎症。如果症状得到明显控制，医生可能酌情减少NSAIDs的用量。

- **传统抗风湿药DMARDs**：如甲氨蝶呤、柳氮磺胺吡啶，主要用于伴发周围关节炎的患者。对于脊柱为主的病变，其效果不显著，可能会被生物制剂部分或完全取代。

- **糖皮质激素**：这不是强直性脊柱炎的常规用药，但某些特殊情况下（如急性炎症严重发作）可能会短期使用。

### 3. **物理治疗和功能锻炼**

无论是否使用生物制剂，物理治疗都是治疗的重要组成部分。坚持腰背肌锻炼、呼吸运动等功能训练，可以维持脊柱灵活性，防止畸形。

### 4. **定期随访**

在治疗过程中，您需要定期复诊，检测以下内容：

- 疾病活动程度（如BASDAI评分）

- 生物制剂使用的安全性（如感染风险、检查血常规、肝肾功能、结核排查）

- 其他并发症的监测（如骨质疏松、关节外症状等）

### 5. **个体化调整**

根据您的具体症状、治疗反应及生活需求，医生可能会动态调整药物方案。具体是否需要继续使用辅助药物（如NSAIDs），需要医生根据您的疾病活动状况来判断。

### 总结

生物制剂在强直性脊柱炎治疗中效果显著，但并不总是单独使用。在治疗过程中，请您保持与风湿科医生或主诊医生的密切沟通，遵医嘱调整药物方案，同时结合物理治疗和健康生活习惯，这样能更好地控制疾病、提高生活质量。

如果有具体用药调整或病情变化，建议随时联系专业医生进一步商讨治疗方案。祝您早日稳定病情，健康生活！

1. **目前我已确诊为强直性脊柱炎，在后续治疗过程中我产生了如下疑惑:是不是只要开始使用生物制剂就得一直用，不能停了？请你作为一名专业的医务工作人员向我提供健康意见**

强直性脊柱炎（AS）是一种慢性炎性疾病，主要影响脊柱以及骶髂关节，可能影响生活质量。针对你的问题，我将给出以下专业建议：

### 一、生物制剂使用的基本原则

生物制剂（如肿瘤坏死因子抑制剂TNF-α抑制剂等）是治疗中重度强直性脊柱炎的重要手段，尤其是在传统非甾体抗炎药（NSAIDs）效果不足的情况下。其主要作用是减轻炎症、缓解症状以及减慢或阻止疾病的进展。而对于是否需要长期使用应根据个体病情、治疗目标和疗效评估来综合确定。

### 二、生物制剂的使用并不一定需要永远持续

1. **治疗目标与停药可能性**：

- 强直性脊柱炎的治疗目标是实现病情的全面控制（即低疾病活动状态或达成临床缓解）。

- 当病情稳定且持续缓解一定时间后（通常需要由专业医生根据具体指标判断，如评分标准BASDAI、影像学评估、血液检查结果等），可以讨论减少药物剂量或间断治疗的可能性。

2. **个体化管理**：

- **病情较轻**：对于症状较轻、影像学进展慢的患者，部分可能不需要长期使用生物制剂，而通过传统药物和锻炼维持疗效。

- **病情重或反复**：对于疾病活动性较高、停药后易复发的患者，通常建议长期维持治疗。

3. **停药的风险**：

- 生物制剂的停药可能导致症状复发或病情加重，因此停药或减量需要在医务人员的指导下逐步进行，避免突然中断。

4. **动态评估与随访**：

- 对于强直性脊柱炎患者，治疗过程中的定期随访和病情评估尤为关键，这有助于及时判断治疗效果和调整方案。

### 三、医生与患者共同决策

强直性脊柱炎是一种慢性且需长期管理的疾病，应与医生充分沟通病情、治疗效果和用药计划。尤其是考虑减量或停用生物制剂时，需结合以下因素进行综合决策：

- 病情（是否已控制）

- 用药耐受性和副作用

- 患者的个体意愿和生活需求

### 四、其他辅助措施

除了药物治疗，规律的功能锻炼、改善生活方式（如戒烟、调整姿势）以及心理支持也在疾病管理中占重要地位。

### 总结

是否需要长期使用生物制剂取决于你的病情控制情况和个人反应。治疗强直性脊柱炎需因人而异，坚持个体化治疗策略，并与主治医生保持密切沟通与随访。建议在接下来的治疗过程中，及时咨询风湿免疫科医生，以便为你制定更加科学有效的长期管理方案。

1. **目前我已确诊为强直性脊柱炎，在后续治疗过程中我产生了如下疑惑:激素能治疗强直性脊柱炎么？请你作为一名专业的医务工作人员向我提供健康意见**

强直性脊柱炎（AS）是一种慢性炎症性疾病，主要影响脊柱和骶髂关节，有时还会涉及其他关节和组织器官。针对您提出的问题，我将提供一些关于激素治疗的信息和专业建议：

### 1. **激素在强直性脊柱炎治疗中的作用**

激素（如糖皮质激素）主要用于调节炎症反应，具有很强的抗炎效果。然而，在强直性脊柱炎的治疗中，激素通常不是主要的治疗手段。其具体应用场景如下：

- **局部使用：** 对关节炎症较为集中的区域，如膝关节、髋关节或其他外周关节，可以通过关节腔内注射糖皮质激素来减轻炎症和疼痛。

- **系统使用：** 对于脊柱受累较严重或伴有全身性症状的患者，很少长期使用口服或静脉注射糖皮质激素，仅在短时间内缓解急性炎症时可能应用。

### 2. **激素治疗的局限性和副作用**

尽管激素能迅速缓解炎症，但在AS中长期使用可能存在以下问题：

- **疗效有限：** 激素对于改善脊柱的病理损伤（如骨质增生、椎间盘钙化）效果有限，不能阻止结构性破坏。

- **副作用：** 长期系统性使用可能会引起骨质疏松、体重增加、高血压、感染风险增加等问题。

- **替代疗法更佳：** 相较于激素，其他形式的抗炎疗法（如NSAIDs、TNF-α抑制剂、生物制剂）已被证明疗效更好，而且长期副作用较少。

### 3. **推荐的治疗策略**

目前，强直性脊柱炎的治疗遵循个性化、多学科管理的原则：

- **基础治疗：** 非甾体抗炎药（NSAIDs），如布洛芬、塞来昔布，可以作为一线治疗方案，用于减轻疼痛和控制炎症。

- **生物制剂/靶向合成药物：** 对于常规治疗效果不佳的患者，可以考虑应用如TNF-α抑制剂、生物制剂（如依那西普、阿达木单抗），或JAK抑制剂等。

- **物理康复：** 通过专业的运动锻炼（如伸展锻炼和姿势调整）可以改善关节功能和生活质量。

- **个性化医疗方案：** 根据每位患者的病情进展和伴随症状，与风湿科医生保持密切沟通，制定优化的治疗方案。

### 4. **我的专业建议**

- **激素的使用：** 如果病情需要，可以根据风湿科医生的建议酌情短期应用激素，但应避免长期、大剂量使用。

- **定期复查：** 强直性脊柱炎是一种慢性疾病，需要长期监测包括核磁影像、炎症标志物（如CRP、ESR）等，以评估病情进展和治疗效果。

- **心理调适与生活方式管理：** 通过合理饮食、健康运动、保持乐观情绪以及避免吸烟、酗酒等不良习惯，有助于整体健康状态的改善。

### 5. **温馨提醒**

强直性脊柱炎的治疗是一个长期过程，建议您定期与风湿科医生沟通，按照医嘱制定合理的治疗计划。如果对某些治疗手段存在疑虑，可以随时与医生讨论，用科学依据指导治疗选择。

1. **目前我已确诊为强直性脊柱炎，在后续治疗过程中我产生了如下疑惑:我准备做手术，我还能继续吃药/打生物制剂么？请你作为一名专业的医务工作人员向我提供健康意见**

强直性脊柱炎（AS）是一种慢性炎性疾病，其治疗以缓解症状、控制炎症和防止结构损伤为目标。关于您在手术期间是否可以继续使用药物（包括生物制剂），以下是一些专业建议，但具体方案需要在专科医生的指导下进行：

### 1. **手术期间药物管理的重要性**

手术期间药物使用的管理需要综合考虑手术类型、炎症活动情况、感染风险及潜在并发症。

### 2. **关于非甾体类抗炎药（NSAIDs）**

- **作用**：NSAIDs是强直性脊柱炎的一线治疗药物，可缓解疼痛和炎症。

- **注意事项**：手术前若长期使用NSAIDs，需告知医生。因为此类药物可能增加出血风险，手术当天通常建议停用。如果手术伤口出血风险较高，术前需提前几天停药。

### 3. **关于生物制剂（如TNF抑制剂或IL-17抑制剂）**

- **作用**：生物制剂能够有效控制强直性脊柱炎的炎症和疾病活动。

- **术前停药**：生物制剂可能会增加术后感染的风险，因此通常建议在手术前停用一段时间。这一时长因具体药物而异：

- TNF抑制剂（如依那西普、阿达木单抗等）：通常建议在最后一次用药后等待1-4周再进行手术。

- IL-17抑制剂（如司库奇尤单抗）：可能也需要术前停药，具体时长视药物半衰期而定。

- **术后用药**：手术后如果没有感染或其他并发症，并在伤口愈合稳定的情况下，可以在医生评估后重新开始使用生物制剂。

### 4. **糖皮质激素**

- 如果您长期使用糖皮质激素（如泼尼松），术前不能突然停药。相反，可能需要临时增加剂量以应对手术引起的应激反应。

- 手术过程中，医生会监测激素水平并适当调整剂量。

### 5. **预防感染**

- 手术期间感染是一个关键关注点，特别是对于使用生物制剂的患者。术前需进行全面评估，确保没有活动性感染。

- 医生可能会建议您在手术前后使用预防性抗生素。

### 6. **与您的医生密切沟通**

- 向您的手术医生和风湿病医生同时说明您的病情和用药情况。

- 制定围手术期的个体化药物管理方案，权衡炎症控制与感染风险。

### 总结建议

在您准备进行手术时：

1. 手术团队和风湿免疫科医生需密切协作。

2. 生物制剂通常需要在术前停用一段时间，并于术后根据恢复情况再重新使用。

3. NSAIDs需根据手术类型调整使用。

4. 确保术后预防感染措施完善。

请咨询您的主治医生，他们会基于您的病情和治疗方案给予更具体的建议和安排。祝您手术顺利，早日康复！

1. **目前我已确诊为强直性脊柱炎，在后续治疗过程中我产生了如下疑惑:治强直性脊柱炎的药能和感冒药一起吃么？请你作为一名专业的医务工作人员向我提供健康意见**

强直性脊柱炎（AS）的治疗常需要使用非甾体抗炎药（NSAIDs）、免疫抑制剂、生物制剂或一些其他药物。这类药物和感冒药是否能一起使用，需要具体分析两类药物的成分以及潜在的相互作用。以下是一些可能的风险和建议供您参考：

### 1. **非甾体抗炎药（NSAIDs）与感冒药**

常规治疗强直性脊柱炎的药物中，NSAIDs（如布洛芬、塞来昔布）是常见初步治疗手段，而许多感冒药也可能含有解热镇痛成分（如对乙酰氨基酚或布洛芬及伪麻黄碱）。两者之间的注意点在于：

- **避免过量同类成分**：如果感冒药中已经含有NSAIDs成分（如布洛芬），则需要小心不要和强直治疗中的NSAIDs叠加使用，否则可能增加胃肠道副作用（如溃疡、出血）或肾功能损害。

- **减少肝肾负担**：部分感冒药（如对乙酰氨基酚）和NSAIDs同时使用可能进一步增加肝肾负担，尤其是长时间服用。

### 2. **免疫抑制剂或生物制剂与感冒药**

强直性脊柱炎患者常会使用免疫抑制剂或生物制剂（如甲氨蝶呤、阿达木单抗等）来减缓疾病进展及控制炎症。这些药物可以削弱免疫系统，可能导致：

- **感染风险增加**：免疫抑制剂的使用会导致患者身体的抵抗力下降，即使是普通感冒也有可能引发较严重的感染。

- **需注意抗生素或抗病毒药物的使用**：若感冒伴发细菌感染或病毒感染，感冒药与抗感染药物的选择需慎重，以避免进一步加重免疫系统的压力。

### 3. **如何安全使用感冒药**

- **尽量选择单一成分**：优先选择成分明确且单一的感冒药，如单用对乙酰氨基酚（针对发热和疼痛）或伪麻黄碱（缓解鼻塞），避免含有多种复合成分的感冒药。

- **避免药物冲突**：感冒药不要与酒精或其他可能影响肾功能的药物一起使用。如果正在使用激素（如泼尼松），需要避免不必要的药物叠加。

- **就医指导**：如果感冒症状较重，或者您目前的用药方案涉及免疫抑制剂，需要在医生的指导下进行选择。

### 4. **最终建议**

不同个体的用药背景和身体状况不同，有效且安全的用药必须结合您的具体治疗方案、药物种类以及当前症状。在自行处理感冒药时需谨慎：

- **定期随访**：可向风湿科的主治医生或药师咨询安全的药物搭配。

- **记下所有药物清单**：便于医生及时判断不同药物之间的相互作用。

- **多饮水与休息**：普通感冒一般可通过增加休息和液体摄入自行缓解，若症状持续加重（如高烧、剧烈咳嗽），应及时就医。

如果您方便，可提供目前具体用药清单或感冒药种类，我可以为您进一步详细分析药物间的相互作用。希望您早日康复！

1. **目前我已确诊为强直性脊柱炎，在后续治疗过程中我产生了如下疑惑:得了强直性脊柱炎，我还可以怀孕/备孕么？请你作为一名专业的医务工作人员向我提供健康意见**

强直性脊柱炎（AS）患者确实可以怀孕或备孕，但备孕和整个怀孕过程需要在医生的指导下进行，并根据个体的病情和治疗状况进行周密计划。以下是一些专业建议，仅供参考，建议与您的主治医生进一步讨论：

### 1. **评估自身病情**

- **病情控制状况**：如果您的强直性脊柱炎处于较为稳定的阶段（无明显疾病活动），怀孕的风险会更低。如果病情活动性较高（如有频繁的关节炎症或疼痛），建议先在医生指导下积极控制疾病。

- **评估身体状况**：确保其他脏器（如心脏、肺）的功能正常，尤其是强直性脊柱炎有可能会影响某些器官。

### 2. **药物调整**

- **怀孕和药物兼容性**：某些治疗强直性脊柱炎的药物可能会对胎儿有影响，一些药物需要在备孕前停药（例如甲氨蝶呤），而另一些则可能可以继续使用（如某些生物制剂）。

- **药物指导**：请务必在专科医生（如风湿科医生或生殖医学科医生）的指导下调整和评估药物方案，不要自行停药或随意用药。

### 3. **计划怀孕**

- **与医生沟通**：在计划怀孕前，与风湿科医生和产科医生沟通，制定详细的怀孕计划。

- **健康生活习惯**：维持健康的生活方式，包括均衡饮食、定期锻炼、戒烟戒酒，以提高身体的健康水平。

### 4. **怀孕期间管理**

- **产科和风湿科双重管理**：怀孕期间需要在风湿科医生和产科医生的共同指导下管理疾病和妊娠。

- **监测病情**：怀孕期间需要定期监测强直性脊柱炎的病情，以便及时应对可能出现的病情波动。

### 5. **分娩与产后护理**

- **分娩方式**：绝大多数强直性脊柱炎患者可顺产，但若骨盆受累严重或存在其他妨碍顺产的因素，可能需要考虑剖宫产。

- **产后管理**：分娩后可能疾病会有一定复发风险，因此需要继续关注病情，并在医生指导下恢复或调整治疗。

### 6. **心理支持**

- 强直性脊柱炎患者可能会面临更多压力，包括疾病管理和怀孕的双重挑战，因此，调节心态并寻求心理支持极为重要。

- 可以加入一些患者支持群体，与同样经历怀孕的强直性脊柱炎患者分享经验。

### 特别注意

怀孕计划因人而异，需要考虑到您的具体病情、药物使用情况和身体状况。一定要与风湿科医生以及产科医生密切配合，共同制定最适合您的方案。

总之，强直性脊柱炎并不一定阻碍生育，但需要在专业医生指导下，进行科学、安全的准备。祝您健康顺利！

1. **目前我已确诊为强直性脊柱炎，在后续治疗过程中我产生了如下疑惑:备孕期间，我的药该怎么吃呀？请你作为一名专业的医务工作人员向我提供健康意见**

作为一名医务工作人员，我为您提供以下健康建议。对于确诊强直性脊柱炎（AS）的患者而言，在备孕期间的药物管理确实需要特别的注意和谨慎。这不仅关系到疾病的控制，也涉及到母婴的健康。以下是一些建议，供您参考：

### 1. **与您的主治医生定期沟通**

在备孕前和备孕期间，务必与您的风湿免疫科医生以及妇产科医生进行充分沟通。医生会根据您的病情严重程度、药物使用情况以及备孕计划，为您制定个性化的治疗方案。

### 2. **目前安全用药的调整**

许多治疗强直性脊柱炎的药物在备孕期和怀孕期可能需要调整，因为部分药物可能对胎儿发育产生影响。以下是一些常用药物的注意事项：

#### a. **非甾体抗炎药（NSAIDs）**

- 在备孕期，NSAIDs可能需要停止使用，因为它们可能影响排卵及子宫内膜功能。

- 怀孕后早期一般不建议使用。特别是在孕中晚期，NSAIDs有可能引起胎儿血液循环的改变。

#### b. **生物制剂（如TNF-α抑制剂）**

- 部分生物制剂（如依那西普、阿达木单抗）在怀孕期间被认为是比较安全的，特别是在备孕早期及孕期的前两个月。

- 但仍需根据个体的病情由医生权衡利弊，谨慎使用。

#### c. **免疫抑制剂（如甲氨蝶呤、来氟米特）**

- **甲氨蝶呤**：备孕前至少提前3-6个月停止使用，并进行药物清除；男女双方都需停药。

- **来氟米特**：备孕前需要清除药物（一般使用胆汁酸药物清除），并在血液中确定无残留。

- 一些较为安全的替代免疫调节剂可以在医生指导下选择。

#### d. **糖皮质激素**

- 如果病情需要，一定剂量的糖皮质激素（如泼尼松）在孕期被认为是相对安全的，但需要尽可能使用最低有效剂量。

### 3. **非药物治疗的重要性**

在备孕及孕期，可以通过一些非药物疗法来改善症状和提高生活质量：

- **适度的体育锻炼**：缓解强直性脊柱炎症状，如功能性拉伸和肩背锻炼。

- **健康饮食**：均衡饮食有助于减少炎症反应。

- **适当休息**：避免过度劳累和精神压力，这对于备孕也非常重要。

### 4. **多学科合作**

在备孕中可以寻求风湿科医生、妇产科医生、药师和儿科医生的联合管理，确保母婴健康与病情稳定兼顾。

### 5. **提前停药和监控病情**

任何药物的调整都不可以擅自进行，如需停药请遵医嘱，以防止病情加重。同时，备孕期间应定期随访，监测强直性脊柱炎的活动度，及时调整方案。

### 温馨提示

因为每位患者的情况都不同，上述建议仅提供一般性的指导，具体的用药调整和管理必须由您的主治医生根据您的具体病情、备孕需求和健康条件作出决定。

如果需要进一步帮助，请随时联系您的医生！祝您备孕顺利，早日迎来健康宝宝！

1. **目前我已确诊为强直性脊柱炎，在后续治疗过程中我产生了如下疑惑:用中药或者中医针灸能代替西药么？请你作为一名专业的医务工作人员向我提供健康意见**

很抱歉听到您确诊为强直性脊柱炎（AS），这种疾病虽然尚无根治方法，但通过积极治疗和管理，能有效控制症状、延缓病程，并提高生活质量。针对您的问题，以下是我的专业健康建议：

### 1. **中西医的结合与选择**

- **西药治疗的重要性：**

西药（如非甾体抗炎药NSAIDs、生物制剂如TNF抑制剂等）在强直性脊柱炎的治疗中起到关键作用，其目的是控制炎症、减轻疼痛、保护关节不受进一步的损害。西药治疗是国际公认的标准疗法，尤其是对中重度患者而言，不使用可能导致症状加重或并发症。

- **中医或针灸的辅助角色：**

中医药和针灸在缓解疼痛、促进血液循环、改善心理状态等方面可能具有一定辅助作用。尤其是在一些患者对西药不耐受或症状缓解不足的情况下，中医可作为补充治疗，但不能替代西药。当前尚无确切的临床证据表明单独使用中医中药或针灸能有效控制强直性脊柱炎的炎症和进展。

### 2. **合理采用治疗策略**

- 如果您有意尝试中医针灸或中药，应先与风湿科主治医生沟通，确保不会与当前西药疗程冲突或产生药物间的相互作用。

- 如果选择中医，建议寻找经验丰富的中医师，切勿自行服用未明确来源的中药，因为某些成分可能对肾脏或肝脏造成负担。

### 3. **综合管理及生活干预**

- **康复锻炼：** 规范的功能锻炼（如瑜伽、游泳等）对改善脊柱灵活性和关节功能、防止僵硬极为重要。

- **心理疏导：** 长期慢性病管理易引发焦虑等心理负担，可求助心理医生或加入同病患者群体进行交流。

- **健康生活方式：** 保持均衡饮食、戒烟、避免高酒精摄入等有助于长期健康。

### 4. **定期随访**

不论采用何种方式治疗，都需要定期复查（如血液指标、影像检查），以评估疾病活动度和治疗效果。

### 结论

中医药或针灸可以在强直性脊柱炎的治疗中扮演一定的辅助角色，但目前尚不能替代西药的主要作用。建议您在专科医生指导下以西医为主，结合中医的辅助手段，制定综合而个性化的治疗方案。

希望这些建议对您有所帮助，祝您早日减轻症状，恢复更好的生活状态！

1. **目前我已确诊为强直性脊柱炎，在后续治疗过程中我产生了如下疑惑:我平时就算腰痛也需要锻炼么？请你作为一名专业的医务工作人员向我提供健康意见**

强直性脊柱炎是一种慢性、炎性自身免疫性疾病，常影响脊柱、骶髂关节以及其他部位的关节功能。针对您提到的“腰痛情况下是否需要锻炼”，我的建议如下：

### 1. **锻炼的重要性**

即使存在一些腰部疼痛，适当的锻炼对于强直性脊柱炎的管理也是非常重要的。运动和功能锻炼能够：

- 改善关节的灵活性和活动范围，延缓脊柱强直；

- 防止脊柱畸形的发生；

- 缓解炎症引起的僵硬感；

- 改善体姿和日常活动能力；

- 提升呼吸功能（重要的是维持胸廓的活动性）。

### 2. **制定适合的运动计划**

虽然运动有益，但在疼痛情况下特别需要注意运动方式。以下是一些建议：

- **低冲击运动：** 例如游泳、散步或瑜伽，这类运动对关节压力小，帮助控制疼痛。

- **伸展运动：** 早晨起床后可以进行轻柔的伸展训练，放松僵硬的肌肉和关节。

- **姿势锻炼：** 专注于背部和脊柱正确的姿势，做一些改善体姿的训练，如脊柱伸展锻炼。

- **呼吸运动：** 保持胸廓灵活，深呼吸训练尤为重要。

- **物理治疗：** 在专业物理治疗师的指导下进行特定的康复运动是最安全的方式。

### 3. **避免过度或不当运动**

- **疼痛明显或急性发作时：** 应避免剧烈活动，以免加重病变部位的炎症和损伤。此时可选择静养为主，但不完全卧床。可能需要在医生指导下进行调整。

- **避免高冲击及扭转动作：** 例如跑步、高强度健身、举重等，可能对脊柱造成额外压力。

### 4. **关注其他治疗措施**

除了锻炼外，配合药物治疗（如非甾体抗炎药、DMARDs或生物制剂）以及定期复诊也是管理强直性脊柱炎的重要部分。保持良好的睡眠姿势、使用适当的床垫以及避免过度劳累，也有助于缓解相关症状。

### 5. **与医生沟通**

如果疼痛加重或锻炼过程中出现不适，建议及时与风湿免疫科或康复科医生沟通，调整治疗或运动方案。

**总结：** 腰痛情况下仍然建议适度锻炼，但需选择温和、适合的运动方式，并避免病情加重。如果对于运动计划存有疑问，建议与专业康复治疗师或医生共同制定个性化方案。希望您能积极应对疾病，保持良好生活质量！

1. **目前我已确诊为强直性脊柱炎，在后续治疗过程中我产生了如下疑惑:这个病需要做手术么？请你作为一名专业的医务工作人员向我提供健康意见**

强直性脊柱炎（AS）是一种以慢性植物性炎症为特征的疾病，主要累及脊柱和骶髂关节，甚至可能影响到外周关节和部分器官。以下是关于你提到问题的专业建议：

### 手术治疗是否需要？

**强直性脊柱炎的治疗主要以药物和非手术方法为主**，手术通常不是主要的治疗手段，只有在以下特定情况时才会考虑手术：

1. **脊柱变形严重**：如果强直性脊柱炎导致了显著的脊柱畸形（如脊柱过度弯曲，影响正常视线和姿势），可能需要矫形手术。

2. **髋关节病变显著**：如果累及髋关节，出现严重的关节破坏和功能障碍，导致无法行走或生活受到严重影响，可以考虑进行髋关节置换术。

3. **神经系统并发症**：在病情晚期，可能由于脊柱滑脱或严重变形压迫脊髓，导致神经损伤时，可能需要手术减压。

### 常规治疗手段

1. **药物治疗**：

- **非甾体抗炎药（NSAIDs）**：如布洛芬、奈普生，可帮助缓解疼痛和炎症。

- **生物制剂（如TNF-α抑制剂或IL-17抑制剂）**：用于病情较复杂病例，能够明显减缓疾病进程。

- **缓解病情抗风湿药（如柳氮磺吡啶、甲氨蝶呤）**：针对外周关节症状较重时使用。

2. **物理治疗与康复训练**：

- 通过规律的锻炼（如游泳、瑜伽或脊柱矫正运动）保持脊柱和关节的灵活性。

- 理疗、热敷或专业康复训练可以减轻疼痛、改善姿势。

3. **生活方式管理**：

- 注重正确的坐姿与站姿，避免身体的过度劳损。

- 戒烟，因为吸烟有可能加剧病情进展。

- 保持健康体重，减轻关节负担。

### 小结

绝大多数强直性脊柱炎患者，都可以通过药物、康复和生活方式调整进行良好的控制，并维持较高的生活质量。手术并不是常规治疗手段，仅在病情发展严重、功能活动严重受限或有重大并发症时才需考虑。如果对个人病情有进一步的担忧或疑问，建议与风湿免疫科的医生及时沟通，制定个性化的治疗方案。

希望我的回答能帮助到你！祝你早日康复！

Hunyuan

一、

1.腰背疼痛的常见原因**

#### **炎症性脊柱关节病（需优先排查）**

- **强直性脊柱炎（AS）**

- **典型表现**：慢性腰背痛（尤其是夜间翻身困难、晨僵＞1小时）、活动后缓解、逐渐进展的脊柱僵硬（“竹节样变”）。

- **好发人群**：青年男性（但女性也可能发病），家族史（如HLA-B27阳性）。

- **其他类型**：

- 银屑病关节炎（伴皮肤银屑病）、反应性关节炎（前驱感染后发作）、炎症性肠病相关关节炎（如克罗恩病）。

#### **机械性下背部疼痛（非炎症性）**

- **肌肉/韧带劳损**：久坐、不良姿势、运动损伤导致的肌肉拉伤或腰椎小关节紊乱。

- **腰椎间盘突出症**：疼痛可能放射至下肢，伴麻木/刺痛（需结合MRI判断）。

- **骨质疏松症**：多见于老年人或长期激素使用者，轻微外力易引发压缩性骨折。

#### **其他系统疾病**

- **感染性疾病**：椎间盘炎、脊柱结核、布鲁氏菌病（伴发热、体重下降）。

- **内脏牵涉痛**：肾结石（腰部绞痛+血尿）、胰腺炎（上腹痛向腰背放射）。

- **非骨骼肌肉疾病**：甲状腺功能减退、纤维肌痛、慢性疲劳综合征。

2.警惕AS的“危险信号”**

若您符合以下情况，需尽快就医并重点排查AS：

- **年龄＜40岁**，男性为主。

- **晨僵持续≥1小时**，且活动后减轻。

- **夜间疼痛醒后加重**，需下床活动才能缓解。

- **伴随其他症状**：髋关节或足跟疼痛、虹膜炎、反复口腔溃疡、皮疹（如银屑病）。

- **家族史**：直系亲属有AS或其他脊柱关节炎病史。

3.建议的下一步检查**

**实验室检查**：

- **血常规、血沉（ESR）、C反应蛋白（CRP）**：评估炎症程度。

- **HLA-B27基因检测**：阳性结果支持AS诊断（但并非确诊依据）。

- **骶髂关节MRI**：早期发现关节面炎症和骨髓水肿（AS的金标准）。

**影像学检查**：

- **X线片**：观察脊柱竹节样变、骶髂关节间隙狭窄（晚期特征）。

- **超声或CT**：辅助评估关节和软组织病变。

**专科就诊**：

- **风湿免疫科**：全面排查脊柱关节炎及其他自身免疫病。

- **骨科**：排除腰椎退行性病变或结构性损伤。

4.现阶段自我管理建议**

**保持活动**：

- 避免久坐久站，每日进行轻度有氧运动（如游泳、慢跑）以维持关节灵活性。

- 物理治疗（如瑜伽、普拉提）有助于改善姿势和肌肉力量。

**疼痛缓解**：

- 急性期可短期使用非甾体抗炎药（NSAIDs，如布洛芬），但需在医生指导下使用。

- 热敷或热水浴可能缓解晨僵和肌肉紧张。

**生活习惯调整**：

- 使用符合人体工学的座椅，睡眠时侧卧并夹枕头保持脊柱中立。

- 戒烟（吸烟会加速AS病情进展）。

5.常见误区澄清**

- **“腰背痛=AS”**：许多疾病均可引起腰痛，需结合症状、体征和检查综合判断。

- **“HLA-B27阳性=AS”**：该基因仅是风险因素，约80%的AS患者阳性，但阳性人群中仅有少数发病。

- **“AS无法治疗”**：早期干预（如生物制剂）可有效控制炎症，延缓关节畸变。

二、

1.夜间疼痛加重、活动后缓解的常见原因**

#### **强直性脊柱炎（AS）**

- **核心特征**：

- **夜间痛醒**：因炎症因子在夜间分泌高峰，导致脊柱周围组织水肿和僵硬（静息时疼痛加剧）。

- **晨僵＞1小时**：晨起时脊柱关节黏附，活动后逐渐缓解（需与普通腰肌劳损鉴别）。

- **活动后减轻**：炎症性疼痛在运动后因血液循环改善、关节滑液分泌增加而缓解。

- **其他关联症状**：

- 髋关节或足跟疼痛（约30%患者首发症状在此处）。

- 眼部虹膜炎、口腔溃疡、皮肤银屑病等（需警惕其他脊柱关节炎）。

#### **机械性下背部疼痛（需排除）**

- **腰椎小关节紊乱**：夜间睡眠姿势不良可能导致关节卡压，起床后活动复位后疼痛减轻。

- **肌筋膜疼痛综合征**：慢性肌肉劳损在夜间休息后可能出现痉挛性疼痛，活动后拉伸肌肉缓解。

- **腰椎间盘突出症**：极少数患者可能因夜间椎间盘压力变化引发疼痛，但通常伴随下肢放射痛或麻木。

#### **其他炎症性疾病**

- **纤维肌痛**：广泛性肌肉疼痛，但对活动反应不一，常伴疲劳、睡眠障碍。

- **慢性胰腺炎/肾结石**：疼痛可能放射至背部，但通常伴消化系统症状（如腹痛、血尿）。

2.AS的“预警信号”需重点关注**

若您符合以下情况，需**立即就医并优先排查AS**：

- **年龄＜40岁**，男性（女性症状可能较轻且不典型）。

- **晨僵持续≥1小时**，且活动后明显缓解。

- **夜间疼痛醒后需下床活动才能缓解**（称为“晨僵型”疼痛）。

- **伴随髋关节或足跟肿痛**（AS早期可能仅累及外周关节）。

- **家族史**：直系亲属有AS或银屑病、溃疡性结肠炎等疾病。

3.建议的就诊方向和检查**

#### **优先就诊科室**

- **风湿免疫科**：AS属于自身免疫病，需专科医生通过症状、体征和实验室/影像学综合判断。

- **骨科**：排除腰椎退行性病变或结构性损伤（如压缩性骨折）。

#### **关键检查项目**

- **骶髂关节MRI**：AS早期诊断的“金标准”，可发现关节面炎症、骨髓水肿。

- **HLA-B27基因检测**：阳性结果支持AS诊断（但需结合临床）。

- **炎症指标**：血沉（ESR）、C反应蛋白（CRP）升高提示活动性炎症。

- **X线或CT**：观察脊柱竹节样变、骶髂关节间隙狭窄（晚期特征）。

#### **症状日记记录**

- 记录每日疼痛时间、强度、诱因（如久坐、受凉）、缓解方式。

- 注意是否伴随发热、皮疹、关节肿胀等其他症状。

4.自我管理建议（短期缓解）**

**夜间疼痛应对**：

- 尝试侧卧并夹枕头保持脊柱中立位，避免俯卧压迫腰部。

- 睡前热敷疼痛部位（15-20分钟）或泡热水澡缓解肌肉紧张。

**适度活动**：

- 即使疼痛，也需避免长时间卧床，可进行轻度活动（如步行、伸展运动）。

- **游泳**（自由泳或仰泳）是AS患者的理想运动，可减轻脊柱压力。

**药物缓解**：

- 短期使用非甾体抗炎药（NSAIDs，如布洛芬）改善夜间疼痛，但需在医生指导下使用。

- **避免长期依赖止痛药**，尤其是激素类药物（如泼尼松），可能掩盖病情进展。

5.常见误区澄清**

- **“年轻人腰痛=腰肌劳损”**：AS多见于青壮年，且症状常被误认为普通劳损，延误诊断。

- **“HLA-B27阴性≠不是AS”**：约10%-20%的AS患者HLA-B27阴性，需结合其他检查综合判断。

- **“AS只能吃药控制”**：早期规范治疗（如生物制剂）可显著延缓关节畸变，恢复正常生活。

三、

1.晨僵的常见原因**

#### **强直性脊柱炎（AS）**

- **典型表现**：

- **晨僵＞1小时**：晨起时脊柱和髋关节周围肌肉、韧带僵硬，活动后逐渐缓解。

- **夜间疼痛加重**：因炎症因子夜间活跃，导致休息时疼痛和僵硬加剧。

- **活动后减轻**：与机械性劳损不同，AS的僵硬通过活动（如拉伸、行走）可明显改善。

- **关联症状**：

- 腰背痛、髋关节或足跟肿痛、虹膜炎、家族史等。

#### **类风湿性关节炎（RA）**

- **特点**：

- **对称性关节僵硬**：双手、腕关节同时受累，晨僵可持续数小时。

- **伴随症状**：关节肿胀、压痛，血检可见类风湿因子（RF）或抗环瓜氨酸肽抗体（CCP）阳性。

#### **骨关节炎（OA）**

- **特点**：

- **晨僵＜30分钟**：与炎症无关，因关节软骨退化或滑液减少导致僵硬。

- **活动后加重**：久坐后起身困难，但活动后反而可能因摩擦加剧疼痛。

#### **纤维肌痛（FM）**

- **特点**：

- **广泛性肌肉僵硬和疼痛**：不仅限于关节，可能伴疲劳、睡眠障碍。

- **晨僵不典型**：僵硬感较轻，但活动后可能改善不明显。

#### **机械性原因**

- **睡姿不当或床垫不合适**：导致肌肉或脊柱长时间受压，晨起后局部僵硬。

- **过度劳累或运动损伤**：肌肉拉伤或乳酸堆积引发短暂性僵硬。

2.AS的“危险信号”需特别关注**

若您符合以下情况，**优先排查AS**：

- **年龄＜40岁**，男性为主（女性症状可能较轻且不典型）。

- **晨僵持续≥1小时**，且活动后仅部分缓解（非完全恢复）。

- **夜间疼痛醒后需下床活动才能缓解**（与AS炎症高峰期相关）。

- **伴随外周关节肿痛**（如髋关节、脚踝）或足跟腱炎。

- **家族史**：直系亲属有AS、银屑病或炎症性肠病病史。

3.建议的检查和就医方向**

#### **优先就诊科室**

- **风湿免疫科**：AS属于自身免疫病，需专科医生通过症状、体征和实验室/影像学综合判断。

- **骨科**：排除腰椎退行性病变或骨质疏松症。

#### **关键检查项目**

- **骶髂关节MRI**：AS早期诊断的“金标准”，可发现关节面炎症和骨髓水肿。

- **HLA-B27基因检测**：阳性结果支持AS诊断（但需结合临床）。

- **炎症指标**：血沉（ESR）、C反应蛋白（CRP）升高提示活动性炎症。

- **X线或CT**：观察脊柱竹节样变、骶髂关节间隙狭窄（晚期特征）。

#### **其他可能需要的检查**

- **类风湿因子（RF）、抗CCP抗体**：排除类风湿性关节炎。

- **甲状腺功能、血糖检测**：排除甲状腺功能减退或糖尿病相关僵硬。

4.自我管理建议（短期缓解）**

**改善睡眠姿势**：

- 侧卧时双膝间夹枕头，保持脊柱中立位。

- 避免俯卧压迫腰部。

**晨起热身**：

- 缓慢活动脊柱（如猫牛式伸展）、髋关节（如抬腿屈膝）。

- 热敷僵硬部位（15-20分钟）促进血液循环。

**适度运动**：

- 选择低冲击运动（如游泳、瑜伽）减轻脊柱压力。

- 避免久坐久站，每小时起身活动5分钟。

**药物缓解**：

- 短期使用非甾体抗炎药（如布洛芬）改善晨僵，但需在医生指导下使用。

- **避免长期依赖止痛药**，尤其是激素类药物。

四、

1.腰疼与眼睛痒、腹泻的潜在关联**

#### *强直性脊柱炎（AS）本身通常不直接导致眼睛痒或腹泻**

- AS主要累及脊柱和髋关节，典型症状为夜间疼痛、晨僵、活动后缓解。

- **但需警惕AS的并发症或合并症**：

- **虹膜炎**：约25%-30%的AS患者可能并发虹膜炎（眼睛红肿、疼痛），但**瘙痒并非典型表现**。

- **肠道感染或炎症性肠病**：AS患者可能因免疫力异常或肠道菌群失调出现腹泻，但需排查其他原因（如克罗恩病、溃疡性结肠炎）。

#### **其他脊柱关节炎（需优先排查）**

- **反应性关节炎**：

- **关联表现**：

- 肠道感染（如沙门氏菌、志贺氏菌）或泌尿生殖道感染后，出现关节痛（腰背、膝踝）、腹泻、眼炎（结膜炎、虹膜炎）。

- 眼睛痒可能是结膜炎或过敏反应，需与感染性眼炎鉴别。

- **炎症性肠病相关关节炎（IBD-A）**：

- **克罗恩病/溃疡性结肠炎**患者可能伴随腰背痛、腹泻，但眼部症状较少见（除非合并其他自身免疫病）。

#### **全身性自身免疫疾病**

- **系统性红斑狼疮（SLE）**：

- **多系统受累**：关节痛、皮疹、肾炎、血液异常，少数患者可能因药物过敏引发腹泻或结膜炎。

- **干燥综合征（SS）**：

- **典型表现**：口干、眼干（干眼症），但腰疼和腹泻非特异性症状。

#### **感染或药物反应**

- **肠道感染**（如细菌性痢疾、病毒性胃肠炎）：

- 导致腹泻、腹痛，严重时可引发全身性反应（如发热、腰背肌肉酸痛）。

- **药物副作用**：

- 非甾体抗炎药（NSAIDs）可能引起胃肠道不适或过敏反应（如布洛芬导致腹泻、眼睛瘙痒）。

2.建议的就诊方向和检查**

#### **优先排查脊柱关节炎**

- **风湿免疫科**：

- **完善检查**：血沉（ESR）、C反应蛋白（CRP）、HLA-B27、骶髂关节MRI。

- **询问病史**：近期是否有腹泻、发热、尿道炎等症状（提示反应性关节炎）。

#### **排查胃肠道或感染性疾病**

- **消化内科**：

- **粪便常规、潜血、培养**：排查细菌/病毒感染、炎症性肠病。

- **肠镜检查**：必要时明确肠道病变（如克罗恩病）。

#### **眼科评估**

- **眼科门诊**：

- **裂隙灯检查**：排除结膜炎、虹膜炎、干眼症。

- **过敏原检测**：若眼睛痒持续，需排查过敏性结膜炎。

#### **其他可能检查**

- **自身抗体谱**：抗中性粒细胞胞浆抗体（ANCA）、补体C3/C4、抗核抗体（ANA）。

- **胸片、尿常规**：排除肺部或肾脏受累。

1. 自我管理建议**

**记录症状日记**：

- 记录腰疼时间、强度，腹泻频率、性状（如水样便、黏液便），眼睛痒是否伴随红肿、分泌物。

- 注意是否在腹泻后腰疼加重，或接触过敏原（如花粉、宠物）后眼睛痒发作。

**短期缓解措施**：

- **腹泻**：口服补液盐（ORS）防止脱水，避免乳制品和油腻食物。

- **眼睛痒**：冷敷、人工泪液缓解，避免揉眼（警惕感染）。

- **腰疼**：适度活动（如游泳）减轻僵硬，避免久坐。

**避免自行用药**：

- 慎用抗生素或止泻药（如蒙脱石散）掩盖病情，需医生指导。

五、

1.您的担忧是合理的，但腰疼本身并不一定意味着“严重疾病”。腰疼可能的严重程度分级**

#### **需紧急就医的“危险信号”**

- **立即就诊**：若出现以下情况，提示可能存在严重疾病（如感染、肿瘤、神经压迫）：

- **剧烈疼痛伴发热、寒战**：警惕脊柱感染（如椎间盘炎、结核）。

- **下肢麻木/无力、大小便失禁**：提示马尾综合征（脊髓受压，需急诊手术）。

- **体重骤降、夜间盗汗**：需排除恶性肿瘤或结核。

- **外伤后突发剧痛**：警惕骨折或椎体压缩性骨折（尤其老年人）。

#### **慢性腰疼的常见原因**

- **炎症性脊柱关节病（如AS）**：

- **特点**：慢性持续性疼痛，夜间加重，活动后缓解，可能逐渐进展至脊柱僵硬。

- **严重性**：若未及时治疗，可能导致脊柱畸形和关节功能受限，但早期干预可有效控制。

- **机械性腰痛（如腰肌劳损、腰椎间盘突出）**：

- **特点**：疼痛与姿势相关，休息后缓解，无晨僵或夜间加重。

- **严重性**：通常不会导致瘫痪，但可能反复发作影响生活质量。

- **其他系统疾病（如肾结石、胰腺炎）**：

- **特点**：疼痛常伴其他症状（如血尿、腹痛、黄疸）。

2.需优先排查以下疾病：

#### **强直性脊柱炎（AS）或脊柱关节炎**

- **腰疼特点**：符合炎症性疼痛模式（夜间加重、晨僵＞1小时、活动后减轻）。

- **眼睛痒的可能关联**：AS患者可能因免疫力异常或合并过敏反应出现结膜炎。

- **腹泻的可能关联**：肠道菌群失调、AS相关肠道炎症或药物副作用（如NSAIDs）。

#### **反应性关节炎**

- **特点**：

- 肠道或泌尿生殖道感染后（如沙门氏菌、衣原体）诱发关节痛、腹泻、眼炎。

- 眼睛痒可能是结膜炎或虹膜炎表现。

#### **炎症性肠病相关关节炎（IBD-A）**

- **特点**：克罗恩病或溃疡性结肠炎患者可能伴随腰背痛、腹泻，但眼部症状较少见。

#### **普通腰肌劳损或腰椎退变**

- **可能性较低**：但需通过影像学（如X线/MRI）排除结构性病变。

---

3.建议的下一步行动**

#### **尽快就医评估**

- **优先就诊风湿免疫科**：

- 完善检查：骶髂关节MRI（AS金标准）、HLA-B27、血沉（ESR）、C反应蛋白（CRP）。

- 询问病史：近期是否有腹泻、发热、尿道炎、皮疹等（提示感染或全身性疾病）。

- **眼科和消化内科会诊**：

- 眼科：裂隙灯检查排除结膜炎、虹膜炎。

- 消化内科：粪便常规、肠镜排查炎症性肠病或感染。

#### **自我管理措施（短期缓解）**

- **避免久坐久站**：每小时起身活动5分钟，使用腰部支撑坐垫。

- **适度拉伸**：如猫牛式、骨盆倾斜运动改善脊柱灵活性。

- **控制腹泻**：口服补液盐（ORS）防止脱水，避免乳制品和油腻食物。

- **冷敷/热敷**：急性疼痛期冷敷（每次15分钟），慢性僵硬热敷（每次20分钟）。

#### **药物注意事项**

- **非甾体抗炎药（NSAIDs）**：如布洛芬可短期缓解疼痛和晨僵，但需警惕胃肠道副作用。

- **避免激素类药物**：如泼尼松可能掩盖病情，需医生指导使用。

4.常见误区澄清**

- **“腰疼=癌症”**：恶性肿瘤性腰痛通常伴随体重下降、夜间盗汗等全身症状，您的症状更符合炎症性疼痛。

- **“AS一定会残疾”**：早期规范治疗（如生物制剂）可显著延缓病情，多数患者正常生活。

- **“眼睛痒=AS”**：AS本身不直接导致眼睛痒，但可能合并过敏或结膜炎，需眼科专科评估。

六、

1.立即就医的“危险信号”**

若您的腰疼伴随以下情况，**需24小时内就诊急诊或骨科/外科**：

**剧烈疼痛无法缓解**：如刀割样、电击样疼痛，或疼痛评分≥7/10（10为最痛）。

**下肢麻木/无力或大小便失禁**：提示脊髓受压（如马尾综合征），需急诊手术。

**外伤后突发剧痛**：警惕骨折、椎体压缩性骨折（尤其老年人或骨质疏松者）。

**发热伴寒战**：可能为椎间盘炎、脊柱感染（如结核）。

**体重骤降、夜间盗汗**：需排除肿瘤或严重感染。

2.需尽快就医的“黄牌信号”**

以下情况建议**24-72小时内就诊骨科或风湿免疫科**：

**慢性腰痛持续＞1个月**：无明确诱因（如外伤），且常规休息无法缓解。

**晨僵＞1小时**：晨起时腰部僵硬，活动后仅部分缓解。

**夜间疼痛加重**：平躺时疼痛加剧，需下床活动才能减轻。

**伴随其他症状**：

- 髋关节或足跟肿痛

- 眼睛红肿、视力下降

- 慢性腹泻、皮疹或口腔溃疡

**年龄＜40岁**：年轻患者腰痛需优先排查炎症性脊柱关节病（如AS）。

3.可自我观察的“绿牌情况”**

以下情况可**先尝试家庭护理1-2周**，若无改善再就医：

**急性肌肉劳损**：如久坐、搬重物后出现的酸痛，疼痛局限于肌肉群，无放射痛。

**腰椎小关节紊乱**：偶发短暂性腰痛，活动后可缓解，无晨僵。

**轻中度骨质疏松性疼痛**：老年人轻微腰背酸胀，无双下肢症状。

**已知原因的腰痛**：如近期剧烈运动后的乳酸堆积，或明确诊断为腰椎间盘突出症（已遵医嘱康复）。

4.自我管理建议（短期缓解）**

**休息与活动**：

- 避免久坐久站，每30分钟起身活动腰部。

- 急性期可平卧硬板床，但时间不宜超过3天（长期卧床可能加重僵硬）。

**物理缓解**：

- **热敷**：用热水袋或暖宝宝敷疼痛部位（每次20分钟，每日2-3次）。

- **轻柔拉伸**：如猫牛式、骨盆倾斜运动（需在无痛范围内进行）。

**药物辅助**：

- **非甾体抗炎药（NSAIDs）**：如布洛芬（短期使用，胃病患者需饭后服用）。

- **肌松药**：如盐酸乙哌立松（需医生处方，缓解肌肉痉挛）。

**避免误区**：

- **勿盲目推拿或正骨**：不当操作可能加重椎间盘或神经损伤。

- **勿长期卧床**：可能导致肌肉萎缩和关节粘连。

5.就医前的准备工作**

**记录症状**：

- 疼痛时间、频率、强度（如“晨起后持续2小时”）。

- 伴随症状（如麻木、发热、腹泻等）。

**整理病史**：

- 近期是否有感染、外伤、手术史？

- 是否长期服用止痛药或激素类药物？

**携带资料**：

- 既往病历、检查报告（如X光/MRI）。

- 当前用药清单。

七、

1. **HLA-B27基因**：

- **阳性人群风险**：约80%-90%的AS患者携带HLA-B27基因，但仅有约5%-10%的阳性者最终发病。

- **阴性人群风险**：约10%-20%的AS患者HLA-B27阴性，但家族史仍显著增加风险。

2. **家族聚集性**：

- 若一级亲属（父母、子女）患AS，您的患病风险约为10%-20%；二级亲属（兄弟姐妹、叔伯姑姨）风险较低（约2%-5%）。

---

3.AS的典型症状（需警惕以下表现）**

**慢性腰背痛**：

- **夜间加重**：疼痛醒后需活动才能缓解（“晨僵型”疼痛）。

- **晨僵＞1小时**：晨起时脊柱僵硬，活动后逐渐减轻。

- **活动后缓解**：与机械性劳损不同，AS的疼痛因关节滑液分泌增加而改善。

**脊柱与外周关节症状**：

- **髋关节或足跟疼痛**：约30%患者首发症状在外周关节。

- **脊柱僵硬与畸形**：晚期可能发展为“竹节样脊柱”。

**伴随症状**：

- 虹膜炎、口腔溃疡、银屑病皮疹（需排查其他脊柱关节炎）。

4.您是否需要警惕AS？**

#### **（需就医评估的情况）**

**年龄＜40岁**，男性（女性风险较低但症状可能不典型）。

**背痛符合AS模式**：夜间痛醒、晨僵＞1小时、活动后缓解。

**家族史明确**：直系亲属确诊AS或携带HLA-B27基因。

**其他关联症状**：髋关节肿痛、足跟腱炎、虹膜炎等。

**（可暂观察的情况）**

**背痛性质不符**：如疼痛与姿势相关、无晨僵、活动后加重（更倾向机械性劳损）。

**年龄＞40岁**：AS发病率随年龄增长显著下降。

**无其他系统症状**：仅偶发背痛，无家族史以外的危险信号。

5.建议的就医与检查**

#### **优先就诊科室**

- **风湿免疫科**：AS属于自身免疫病，需专科医生综合评估。

- **骨科**：排除腰椎退行性病变或骨质疏松性骨折。

#### **关键检查项目**

- **HLA-B27基因检测**：明确遗传风险（但需结合临床）。

- **骶髂关节MRI**：AS早期诊断的“金标准”，可发现骨髓水肿和关节炎症。

- **炎症指标**：血沉（ESR）、C反应蛋白（CRP）升高提示活动性炎症。

- **X线或CT**：观察脊柱竹节样变、骶髂关节间隙狭窄（晚期特征）。

#### **其他可能需要的检查**

- **肠道菌群检测**：AS患者常伴肠道微生态失调。

- **眼科检查**：排查虹膜炎等并发症。

6.自我管理与注意事项**

**记录症状日记**：

- 记录背痛时间、强度、诱因（如久坐、受凉）、晨僵时长、家族史细节。

**避免延误诊断**：

- **尽早就医**：若背痛持续＞1个月且符合AS特征，建议3个月内就诊。

**生活方式调整**：

- **戒烟**：吸烟会加速AS病情进展。

- **适度运动**：游泳、瑜伽等低冲击运动有助于维持脊柱灵活性。

**药物干预**：

- **非甾体抗炎药（NSAIDs）**：如布洛芬短期缓解疼痛和晨僵（需医生指导）。

7.常见误区澄清**

- **“有家族史=一定会得AS”**：遗传只是风险因素之一，环境、免疫异常等共同作用。

- **“AS只能年轻人发病”**：虽好发于青壮年，但极少数患者可能在40岁后确诊。

- **“背痛=AS”**：需排除腰椎间盘突出、肌肉劳损等其他疾病。

八、

1.AS背痛的独特特征**

**疼痛模式**：

- **夜间加重**：因炎症因子夜间活跃，导致脊柱周围组织水肿和僵硬（静息时疼痛加剧）。

- **晨僵＞1小时**：晨起时脊柱关节黏附，活动后逐渐缓解（非普通腰肌劳损）。

- **活动后减轻**：炎症性疼痛因血液循环改善而缓解（如游泳、行走后疼痛明显减轻）。

**伴随症状**：

- **外周关节肿痛**：髋关节、足跟、膝盖等部位疼痛（约30%患者首发症状在外周）。

- **晨僵范围广**：可能累及脊柱、髋关节、肩关节等多处。

- **虹膜炎、口腔溃疡**：约25%-30%患者可能并发眼部炎症或反复口腔溃疡。

**病程进展**：

- **慢性持续性**：疼痛持续数月甚至数年，逐渐进展为脊柱僵硬和畸形（如“竹节样脊柱”）。

- **年轻患者高发**：好发于15-30岁青年男性（女性症状较轻且不典型）。

2.其他常见腰背痛的特点**

| **疾病类型** | **疼痛特点** | **伴随症状** | **活动影响** |

|--------------------|----------------------------------|----------------------------------|---------------------------|

| **机械性腰背痛** | 与姿势相关（如久坐、弯腰搬重物） | 无晨僵，局部肌肉压痛或腰椎小关节紊乱 | 休息后缓解，活动后可能加重 |

| **腰椎间盘突出症** | 下肢放射性疼痛（如臀部→大腿后侧） | 伴麻木、刺痛，直腿抬高试验阳性 | 咳嗽、打喷嚏可能加重疼痛 |

| **骨质疏松性骨折** | 急性剧痛（老年人常见） | 无晨僵，可能有轻微外伤史 | 平躺加重，站立减轻 |

| **纤维肌痛** | 广泛性肌肉酸痛，触碰痛 | 疲劳、睡眠障碍、情绪焦虑 | 活动可能缓解但不明显 |

| **肾结石/胰腺炎** | 腰部绞痛或钝痛（结石）/上腹痛放射 | 血尿、发热（结石）/腹痛、黄疸（胰腺炎） | 体位改变可能缓解 |

3.AS背痛与其他疼痛的“关键区别表”**

| **特征** | **AS背痛** | **其他腰背痛** |

|--------------------|-------------------------------|------------------------------|

| **时间规律** | 夜间痛醒，晨僵＞1小时 | 白天活动后加重，休息缓解 |

| **活动影响** | 活动后减轻（如游泳、步行） | 活动后可能加重（如久坐后起身） |

| **伴随症状** | 髋关节痛、虹膜炎、家族史 | 局部压痛、下肢放射痛、发热等 |

| **年龄分布** | 青年男性为主 | 无年龄特异性（如中老年骨质疏松） |

| **影像学特征** | 骶髂关节MRI显示骨髓水肿 | X线/MRI可见椎间盘突出或骨折 |

4.建议就医的情况**

若您的背痛符合以下任意一条，需尽快就诊风湿免疫科：

**年龄＜40岁**，背痛持续＞1个月且符合AS模式（夜间痛、晨僵）。

**伴随髋关节或足跟肿痛**，或反复虹膜炎、口腔溃疡。

**家族史明确**：直系亲属确诊AS或HLA-B27基因阳性。

**常规治疗无效**：如非甾体抗炎药（布洛芬）无法缓解症状。

5.自我管理建议**

**避免久坐久站**：每30分钟起身活动，使用腰部支撑坐垫。

**低冲击运动**：如游泳、瑜伽（避免剧烈运动加重炎症）。

**睡姿调整**：侧卧时双膝间夹枕头，保持脊柱中立位。

**短期药物缓解**：

- **非甾体抗炎药（NSAIDs）**：如布洛芬（需饭后服用，胃病患者慎用）。

- **避免激素类药物**：如泼尼松可能掩盖病情，需医生指导。

6.常见误区澄清**

- **“AS背痛必须剧烈”**：早期疼痛可能轻微，呈隐匿性进展。

- **“只有男性会得AS”**：女性可能发病，但症状常被误认为腰肌劳损。

- **“AS无法确诊”**：骶髂关节MRI和HLA-B27检测可早期明确诊断。

九、

1.AS可能累及胸廓和肋骨关节**

**胸椎炎症与椎体融合**：

- AS的炎症可能从腰椎向上蔓延至胸椎，导致胸椎关节肿胀、僵硬，甚至椎体间骨桥形成（“竹节样变”）。

- **胸廓活动受限**：胸椎融合后，呼吸时胸廓扩张受限，可能引发深呼吸或咳嗽时的胸部闷痛。

**肋骨关节（肋椎关节）炎症**：

- AS可累及肋骨与胸椎的连接处（肋椎关节），导致局部疼痛，尤其在转身、侧弯或按压时加重。

- **肋间神经刺激**：炎症可能压迫肋间神经，引起放射性疼痛（类似心绞痛但位置更偏向背部或侧胸）。

2.AS的典型症状可能间接提示胸廓问题**

**慢性腰背痛与晨僵**：

- 若您长期存在夜间腰痛、晨僵＞1小时等症状，医生可能通过“脊柱关节炎”的线索联想到AS，并进一步排查胸廓受累。

**家族史与HLA-B27阳性**：

- 若您有AS家族史或HLA-B27基因阳性，医生会更警惕胸廓关节受累的可能性。

**其他伴随症状**：

- **虹膜炎、口腔溃疡**：AS患者可能合并其他脊柱关节炎表现，提示全身性炎症。

- **髋关节或足跟疼痛**：约30%的AS患者首发症状在外周关节，需全面排查。

3.医生如何区分胸口疼的其他原因？**

医生会通过以下步骤排除其他疾病，最终聚焦AS：

**病史询问**：

- 疼痛性质（如钝痛、刺痛、烧灼感）、持续时间、诱发因素（如呼吸、咳嗽、体位改变）。

- 是否伴随发热、皮疹、关节肿痛、胃肠道症状等。

**体格检查**：

- **胸廓扩张度测试**：测量吸气与呼气时胸围变化，AS患者可能因胸椎融合导致扩张受限。

- **压痛部位**：检查胸椎棘突、肋间关节是否有压痛，判断炎症位置。

- **脊柱活动度**：评估腰椎和胸椎的弯曲、旋转功能是否受限。

**影像学检查**：

- **胸部X线或CT**：观察胸椎骨质增生、椎体间隙狭窄、肋骨关节模糊等AS特征。

- **骶髂关节MRI**：AS的金标准检查，明确骶髂关节炎症和骨髓水肿。

**实验室检查**：

- **血沉（ESR）、C反应蛋白（CRP）**：升高提示炎症活动。

- **HLA-B27基因检测**：阳性结果支持AS诊断（但需结合临床）。

4.胸口疼的其他可能原因（医生需排除的疾病）**

**心血管疾病**：

- **心绞痛、心肌梗死**：胸骨后压榨性疼痛，放射至左肩或下颌，需立即急诊处理。

- **心肌炎、心包炎**：年轻患者可能表现为胸痛伴发热、呼吸困难。

**胸壁或肌肉问题**：

- **肋间神经炎**：病毒感染或创伤导致沿肋间神经的刺痛。

- **肌肉拉伤或劳损**：剧烈运动或久坐后的胸部肌肉疼痛。

**消化道疾病**：

- **胃食管反流病（GERD）**：胸骨后灼痛，平躺时加重。

- **食管痉挛或破裂**：突发剧烈胸痛，需紧急处理。

**肺部疾病**：

- **肺炎、胸膜炎**：胸痛伴咳嗽、咳痰、发热。

- **肺栓塞**：高危人群（长期卧床、术后）突发胸痛伴呼吸困难。

**焦虑或功能性疼痛**：

- **躯体化症状**：长期压力导致的胸痛，无明确器质性病变。

5.为什么医生会优先考虑AS？**

若您的胸口疼符合以下情况，医生可能将AS列为重点排查对象：

**年龄＜40岁**，男性为主（女性症状可能较轻）。

**胸痛伴随脊柱或髋关节症状**（如腰背痛、髋关节肿痛）。

**夜间疼痛加重、晨僵＞1小时**（AS典型炎症模式）。

**HLA-B27阳性**或家族史明确。

**影像学检查发现骶髂关节或胸椎异常**。

6.下一步建议**

**尽快完善检查**：

- **胸部CT或MRI**：评估胸椎和肋骨关节炎症。

- **心电图、心肌酶谱**：排除心脏病变。

- **胃镜或24小时食管pH监测**：排查GERD。

**就诊专科**：

- **风湿免疫科**：AS的确诊与治疗。

- **心内科、呼吸科、消化内科**：排除心血管、肺部、消化道疾病。

**自我管理**：

- **避免剧烈运动**：减少胸廓压力。

- **热敷或冷敷**：急性疼痛期冷敷（每次15分钟），慢性僵硬热敷（每次20分钟）。

- **记录症状日记**：详细记录胸痛时间、诱因、伴随症状（如发热、呼吸困难）。

7.常见误区澄清**

- **“AS只会导致腰背痛”**：AS可累及全身关节和胸廓，胸痛可能是早期表现。

- **“年轻人不会得心脏病”**：心绞痛、心肌炎等疾病在年轻人中也可能发生，需紧急排除。

- **“HLA-B27阳性=AS”**：仅作为辅助诊断指标，需结合临床症状和影像学结果。

十、

1.情绪压力如何导致腰疼？**

**肌肉紧张与痉挛**：

- **机制**：压力会触发交感神经兴奋，导致骨骼肌持续收缩（如竖脊肌、髂腰肌），引发局部缺血和乳酸堆积，表现为酸胀、刺痛。

- **典型表现**：久坐办公族、学生党因长期伏案产生的“腰肌劳损”多与此相关。

**炎症反应加剧**：

- **慢性压力**：长期压力会激活免疫系统，释放促炎因子（如IL-6、TNF-α），可能加重炎症性疼痛（如AS患者的脊柱炎症）。

**姿势代偿性改变**：

- **驼背、圆肩**：压力下不良姿势（如低头看手机、缩肩）会增加腰椎间盘和小关节压力，导致慢性损伤。

**睡眠质量下降**：

- **失眠、多梦**：压力引起的睡眠障碍会加重肌肉疲劳，形成“疼痛-失眠-更痛”的恶性循环。

2.情绪压力腰疼的典型特征**

| **症状** | **压力相关腰疼** | **其他原因腰疼** |

|----------------------|---------------------------------|------------------------------|

| **疼痛性质** | 隐匿性钝痛、酸胀感，无明确痛点 | 明确的刺痛、放射痛（如腰椎间盘突出） |

| **诱发因素** | 焦虑、久坐、熬夜、考试前 | 体力劳动、外伤、受凉 |

| **缓解方式** | 放松训练、热敷、心理咨询 | 物理治疗、药物干预 |

| **伴随症状** | 头晕、乏力、注意力下降 | 下肢麻木、发热、大小便失禁 |

3.需警惕的“危险信号”**

若您的腰疼伴随以下情况，**需优先排查器质性疾病**（如AS、腰椎间盘突出）：

**夜间痛醒**：平躺时疼痛加剧，需下床活动缓解。

**晨僵＞1小时**：晨起时腰部僵硬，活动后仅部分缓解。

**单侧下肢放射痛**：如从腰部到臀部、大腿后侧的“坐骨神经痛”。

**外伤史或体重骤降**：提示骨折、肿瘤或感染。

4.自我管理建议**

**压力调节技巧**：

- **深呼吸练习**：每日10分钟腹式呼吸，降低交感神经兴奋性。

- **正念冥想**：通过APP（如Headspace）进行每日15分钟训练。

- **渐进式肌肉放松**：从脚趾到头部逐组肌群先紧绷后放松。

**物理缓解措施**：

- **猫牛式伸展**：跪姿交替拱背（猫）和塌腰（牛），改善脊柱灵活性。

- **仰卧骨盆倾斜**：平躺屈膝，缓慢将下背贴向地面，缓解腰椎压力。

- **热敷或低频电疗**：促进局部血液循环，缓解肌肉痉挛。

**生活方式调整**：

- **每小时起身活动**：避免久坐，使用符合人体工学的腰靠和座椅。

- **睡眠姿势优化**：侧卧时双膝间夹枕头，保持脊柱中立位。

- **减少咖啡因和酒精摄入**：避免加重焦虑和肌肉紧张。

**心理干预**：

- **认知行为疗法（CBT）**：针对慢性疼痛的心理干预，改善疼痛感知。

- **运动疗法**：瑜伽、普拉提等低冲击运动兼具减压和康复效果。

5.何时需就医？**

若尝试自我管理1-2周后无改善，或出现以下情况，需及时就诊：

**疼痛持续加重**：影响日常生活（如无法弯腰系鞋带）。

**伴随神经系统症状**：下肢麻木、无力、大小便失禁（提示马尾综合征）。

**发热或体重下降**：需排除感染或肿瘤。

**已知AS家族史**：需优先排查炎症性脊柱关节病。

6.常见误区澄清**

- **“腰疼都是心理问题”**：压力可能是诱因，但需排除骨折、椎间盘突出等器质性疾病。

- **“年轻人不会得腰椎退化”**：长期不良姿势可能导致早发性腰椎劳损。

- **“忍痛就能扛过去”**：慢性腰疼可能进展为慢性疼痛综合征，增加治疗难度。

十一、

1. HLA-B27是一种存在于人体白细胞表面的蛋白质，属于人类白细胞抗原（HLA）系统的一部分。HLA基因主要负责调节免疫反应，不同HLA类型与疾病易感性相关。当您的检测结果显示**HLA-B27阳性**时，意味着您携带了这种基因型，

**与强直性脊柱炎（AS）的关联**

- **强直性脊柱炎**是一种慢性炎症性疾病，主要影响脊柱和骶髂关节，导致关节僵硬、疼痛，严重时可致残。

- **约90%的AS患者为HLA-B27阳性**，但并非所有阳性者都会患病（人群中HLA-B27阳性率约为5%-10%，仅少数发展为AS）。

- 因此，**HLA-B27阳性是AS的重要风险因素，但不是唯一诊断依据**。

其他可能相关的疾病**

2.HLA-B27阳性还可能增加以下疾病的患病风险：

- **反应性关节炎**：继发于感染（如肠道或泌尿系统感染）后的关节炎症。

- **银屑病性关节炎**：部分银屑病患者合并的关节炎。

- **炎症性肠病相关关节炎**：克罗恩病或溃疡性结肠炎伴发的关节病变。

- **幼年特发性关节炎**：儿童期发病的慢性关节炎。

3.### ** 阳性结果的临床意义**

- **若您有AS症状**（如持续性下腰背痛、晨僵>1小时、活动后缓解、夜间痛醒等），HLA-B27阳性可支持AS的诊断，但需结合影像学（如骶髂关节MRI）和炎症指标（如血沉、C反应蛋白）综合判断。

- **若无症状**：阳性结果仅表明未来患病风险略高（尤其是有家族史者），无需过度焦虑，定期监测即可。

4.### ** 下一步建议**

- **完善检查**：如骶髂关节影像学（X光/MRI）、血液炎症指标（ESR、CRP）、风湿免疫抗体（如ANA、RF）等。

- **专科就诊**：建议至风湿免疫科进一步评估，医生会根据症状、体征和检查结果制定个体化方案。

- **生活方式管理**：无论是否确诊，保持适度运动（如游泳、瑜伽）、避免久坐、戒烟均有助于关节健康。

十二、

1. CRP和ESR是什么**

- **C-反应蛋白（CRP）**：

由肝脏合成的急性期蛋白，当身体发生炎症（如感染或自身免疫病）时，血液中的CRP水平会迅速升高。它是一种**非特异性炎症标志物**，常见于多种炎症性疾病、感染或组织损伤。

- **血沉（ESR）**：

反映红细胞在血液中下沉的速度，受炎症因子（如纤维蛋白原、免疫球蛋白）影响。炎症越严重，ESR通常越快。同样属于**非特异性指标**，广泛用于评估炎症活动度。

2. 为什么AS患者会出现CRP和ESR升高？**

- **AS的炎症本质**：

强直性脊柱炎是脊柱和骶髂关节的慢性炎症性疾病，炎症反应会导致CRP和ESR升高，尤其在疾病**活动期**（症状加重时）更为明显。

- **与HLA-B27的关系**：

HLA-B27阳性人群的免疫系统更易触发异常炎症反应，可能导致CRP和ESR水平升高。但**即使HLA-B27阴性**，AS患者也可能因炎症而出现这两个指标升高。

3. CRP和ESR升高是否意味着一定患AS？**

- **答案是否定的**。

虽然AS患者常伴有CRP和ESR升高，但这两个指标**并非AS特有**，其他疾病也可能导致类似结果，例如：

- **其他脊柱关节炎**：如反应性关节炎、银屑病性关节炎。

- **类风湿性关节炎**（RA）：RA患者的CRP和ESR通常更高，但AS主要影响脊柱而非手部小关节。

- **感染或慢性炎症**：如细菌感染、结核、溃疡性结肠炎。

- **非炎症性疾病**：如恶性肿瘤、贫血、甲状腺功能异常。

4. 如何通过CRP和ESR辅助诊断AS？**

- **结合临床表现**：

AS的典型症状包括**持续性下腰背痛、晨僵＞1小时、夜间痛醒、活动后缓解**，若同时伴CRP/ESR升高，可支持AS的诊断。

- **排除其他疾病**：

若CRP/ESR显著升高，需排查感染、RA等其他炎症性疾病。例如：

- RA患者常伴有**对称性多关节肿痛**，而AS主要累及脊柱和骶髂关节。

- 感染性关节炎（如链球菌感染）可能伴随CRP急剧升高，但通常有明确感染史。

5. CRP和ESR在AS管理中的作用**

- **评估疾病活动度**：

在AS治疗过程中，医生会监测CRP和ESR水平的变化。若治疗后指标下降，可能提示炎症控制良好；若持续升高，可能需要调整治疗方案。

- **预测疾病进展**：

部分研究表明，基线CRP/ESR水平较高的AS患者可能更容易出现关节破坏或脊柱融合，但需结合影像学（如骶髂关节MRI）综合判断。

6. 下一步建议**

- **专科就诊**：

建议至**风湿免疫科**就诊，医生会结合您的症状、体征、HLA-B27结果及炎症指标，进一步排查AS或其他疾病。

- **完善检查**：

- **影像学检查**：骶髂关节X线/MRI（AS的确诊关键）。

- **其他炎症指标**：如血常规、ANA、RF（排除其他自身免疫病）。

- **感染筛查**：如血培养、结核菌素试验（排除感染性关节炎）。

- **动态监测**：

若初步检查无法确诊，医生可能建议您定期复查CRP/ESR，同时观察症状变化。

十三、

1. X光的局限性**

- **X光对早期病变不敏感**：

强直性脊柱炎（AS）的骶髂关节炎症早期可能仅表现为软骨水肿或轻微骨质增生，这些改变在X光上难以察觉，尤其是**疾病初期或非典型部位受累**时。此时需依赖**MRI或CT**（如骶髂关节MRI可更早发现骨髓水肿和滑膜炎症）。

- **其他原因未被X光捕捉**：

若疼痛来源于**软组织（如肌腱、韧带）**、**小关节（如腰椎小关节）**或**骨膜炎症**，X光可能无法清晰显示，而超声或MRI更敏感。

2. 可能导致疼痛但X光正常的疾病**

#### **强直性脊柱炎（AS）未到影像学明显阶段**

- **AS的典型表现**：

症状常先于影像学异常出现（如持续下腰背痛、晨僵）。若处于**早期或活动期**，X光可能正常，但**MRI或骶髂关节超声**可发现炎症迹象。

- **建议**：

→ 完善**骶髂关节MRI**（评估滑膜炎症和骨髓水肿）。

→ 动态观察：若症状持续，需复查X光或CT（AS晚期可见关节间隙变窄、骨赘形成）。

---

#### **其他脊柱或关节疾病**

- **肌腱炎/筋膜炎**：

如腰椎棘突间韧带炎、梨状肌综合征等，表现为局部剧痛，但X光无异常。

- **骨关节炎（OA）**：

早期OA可能仅表现为软骨磨损，X光在关节间隙狭窄或骨赘形成前可能正常，但**超声或MRI**可更早发现软骨退变。

- **反应性关节炎/银屑病性关节炎**：

这些脊柱关节炎与AS类似，但X光表现可能较轻或不典型，需结合HLA-B27、CRP/ESR等综合判断。

---

#### **非骨骼肌肉系统疾病**

- **肾结石/泌尿系感染**：

腰背部剧痛可能由肾结石或肾盂肾炎引起，X光可能漏诊（需超声或CT排查）。

- **带状疱疹**：

单侧剧烈神经痛伴皮疹，但X光无异常。

- **纤维肌痛症/慢性疲劳综合征**：

广泛性疼痛，但无器质性病变，X光正常。

3.下一步建议**

- **完善其他影像学检查**：

→ **骶髂关节MRI**（AS首选检查）。

→ **腰椎MRI或CT**（评估椎间盘、小关节、神经根压迫）。

→ **超声**（动态观察关节积液、肌腱炎症）。

- **实验室检查**：

→ 血常规、CRP、ESR（评估炎症活动度）。

→ HLA-B27、ANA、RF、抗中性粒细胞胞浆抗体（ANCA）等（排查其他自身免疫病）。

→ 尿常规、肾功能（排除肾脏疾病）。

- **专科就诊**：

→ **风湿免疫科**：排查AS、反应性关节炎等。

→ **骨科或疼痛科**：评估腰椎间盘突出、肌腱炎等机械性疼痛。

→ **泌尿外科或皮肤科**：排除结石、感染或带状疱疹。

- **对症处理**：

→ 短期使用非甾体抗炎药（NSAIDs）缓解疼痛和炎症。

→ 物理治疗（如热敷、理疗）改善局部血液循环。

→ 避免久坐、弯腰提重物等加重疼痛的行为。

十四、

1. **什么是骨髓水肿？**

骨髓水肿是骨髓腔内液体异常积聚的病理状态，常见于**炎症、感染、创伤或代谢性疾病**。它是对组织损伤或炎症的代偿性反应，并非独立疾病，而是某种疾病的“信号”。

2. **严重程度评估**

- **轻度**：仅局部骨髓腔轻微扩张，无明显症状（如偶然影像学发现）。

- **重度**：广泛骨髓水肿，可能伴随疼痛、肿胀或功能障碍（如急性感染或肿瘤压迫）。

- **关键点**：严重性与原发病直接相关，而非水肿本身。例如，AS引起的骨髓水肿可能提示炎症活动，但若及时控制，预后较好；而肿瘤或严重感染则可能危及生命。

3.、骨髓水肿与强直性脊柱炎（AS）的关系**

**AS如何导致骨髓水肿？**

- AS是一种慢性炎症性疾病，主要累及**骶髂关节和脊柱**。

- **早期阶段**：炎症因子（如TNF-α、IL-6）刺激骨髓造血干细胞，导致骨髓水肿（MRI上表现为“骨髓水肿征象”）。

- **中晚期**：关节软骨破坏、骨赘形成，X光可见关节间隙变窄，但MRI仍可捕捉到骨髓水肿。

**AS患者骨髓水肿的特点**

- **部位特异性**：多见于**骶髂关节**和腰椎椎体边缘（与AS发病部位一致）。

- **动态变化**：炎症活动期水肿加重，治疗后减轻。

- **影像学特征**：MRI的T2WI和脂肪抑制像可清晰显示水肿（呈高信号）。

**其他疾病也可能导致骨髓水肿**

- **非AS脊柱关节炎**：如反应性关节炎、银屑病性关节炎。

- **感染性疾病**：如布鲁氏菌病、结核性骶髂关节炎。

- **代谢性疾病**：如骨质疏松、骨坏死。

- **肿瘤或血液系统疾病**：如白血病、多发性骨髓瘤。

4.如何判断骨髓水肿是否与AS相关？**

**结合临床症状**

- AS典型症状：持续下腰背痛、晨僵＞1小时、夜间痛醒、活动后缓解。

- 若伴随**外周关节肿痛、虹膜炎、肠道症状**，更支持AS或脊柱关节炎。

**影像学检查**

- **骶髂关节MRI**：AS早期诊断的金标准，可发现骨髓水肿、滑膜炎症和软骨破坏。

- **X线或CT**：中晚期AS可见关节间隙狭窄、骨赘，但早期可能正常。

**实验室检查**

- **炎症指标**：CRP、ESR升高提示炎症活动。

- **HLA-B27基因检测**：约90%的AS患者阳性，但需结合其他检查。

- **排除性检查**：如血常规、ANA、RF、PPD试验（排除感染或自身免疫病）。

5.下一步建议**

**完善检查**

- **骶髂关节MRI**：明确骨髓水肿范围及是否伴随滑膜炎症。

- **炎症指标**：CRP、ESR、血常规。

- **HLA-B27检测**：评估AS风险。

- **其他检查**：如肠道镜检（排查炎症性肠病）、尿常规（排除泌尿系感染）。

**专科就诊**

- **风湿免疫科**：评估AS可能性及其他脊柱关节炎。

- **骨科或影像科**：分析骨髓水肿的具体原因（如肿瘤、感染）。

**对症处理**

- **控制炎症**：短期使用非甾体抗炎药（NSAIDs）缓解疼痛和水肿。

- **物理治疗**：热敷、理疗改善局部血液循环。

- **生活方式调整**：避免久坐、弯腰提重物，适度运动（如游泳、瑜伽）。

十六、

1.了解强直性脊柱炎的核心特征**

强直性脊柱炎（AS）是一种**慢性炎症性疾病**，主要累及脊柱和骶髂关节，典型表现为**炎症性疼痛、晨僵和关节活动受限**。以下是关键特征：

#### **典型症状**

- **下腰背痛**：

- 持续性钝痛或隐痛，夜间加重、晨起僵硬（**晨僵＞1小时**），活动后缓解。

- 可能逐渐向上蔓延至胸椎和颈椎（“竹节样脊柱”晚期表现）。

- **骶髂关节症状**：

- 髋部或臀部疼痛，尤其在久坐、站立或行走后加重。

- **外周关节受累**：

- 30%-50%患者早期出现膝、踝、足跟等关节肿痛（非对称性）。

- **其他全身症状**：

- 眼炎（虹膜炎）、口腔溃疡、肠道炎症（如克罗恩病）等。

#### **体征**

- **腰椎活动度受限**：

- 弯腰、后仰或侧弯时疼痛加剧，活动范围逐渐缩小。

- **骶髂关节压痛**：

- 检查时医生按压骶髂关节区域可能有压痛。

- **“4字试验”阳性**：

- 单膝跪地时，另一侧髋关节屈曲内收内旋引发疼痛（提示骶髂关节病变）。

2.自我评估工具：ASAS诊断标准（简化版）**

国际脊柱关节炎评估协会（ASAS）标准将AS诊断分为**影像学确诊**和**非影像学确诊**两类，供参考：

#### **影像学确诊AS（需骶髂关节MRI或CT）**

- **满足以下条件之一**：

- **腰背痛≥3个月**，且符合AS的影像学特征（如骶髂关节骨髓水肿、滑膜炎症）。

- **脊柱活动度受限**（如Schober试验＜2cm）。

#### *非影像学确诊AS（仅症状+实验室检查）**

- **腰背痛≥3个月**，且符合以下至少4项：

- （1）晨僵＞1小时；

- （2）夜间痛醒；

- （3）活动后疼痛缓解；

- （4）非甾体抗炎药（NSAIDs）治疗有效；

- （5）HLA-B27阳性；

- （6）CRP或ESR升高。

3.需要排除的其他疾病**

以下疾病可能与AS症状相似，需通过检查区分：

- **机械性腰背痛**：如腰椎间盘突出、肌腱炎、骨质疏松。

- **其他脊柱关节炎**：反应性关节炎、银屑病性关节炎、炎症性肠病相关关节炎。

- **类风湿性关节炎（RA）**：RA多累及手部小关节，对称性肿痛，AS主要影响脊柱。

- **感染性疾病**：如结核性骶髂关节炎、布鲁氏菌病。

4.就医建议：确诊AS需要哪些检查？**

**影像学检查**：

- **骶髂关节MRI**：AS早期诊断的金标准，可发现骨髓水肿、滑膜炎症。

- **X线或CT**：中晚期AS可见关节间隙狭窄、骨赘形成。

**实验室检查**：

- **HLA-B27基因检测**：约90% AS患者阳性。

- **炎症指标**：CRP、ESR升高提示炎症活动。

- **排除性检查**：ANA、RF、血常规、尿常规、PPD试验等。

**专科就诊**：

- **风湿免疫科**：综合症状、体征和检查结果明确诊断。

- **骨科或康复科**：评估脊柱功能和生活质量。

5.日常注意事项**

- **记录症状日记**：

记录每日疼痛时间、强度、活动后缓解情况，帮助医生判断病情。

- **避免久坐久站**：

每30分钟变换姿势，适度运动（如游泳、瑜伽）改善关节灵活性。

- **戒烟**：

吸烟会加速AS病情进展，增加脊柱融合风险。

十七、

1.医生开具X光、CT、MRI和抽血化验的目的是为了全面评估病情，但并非所有检查都必需。以下是具体分析，帮助您理解医生意图并做出合理选择：

各项检查的作用与必要性**

#### **影像学检查**

| **检查类型** | **用途** | **必要性** | **适用情况** |

|--------------|----------|------------|--------------|

| **X光** | 观察脊柱、骶髂关节骨结构（如关节间隙、骨赘） | **中晚期AS必备** | 疑似关节畸形或骨破坏时（如竹节样脊柱） |

| **CT** | 更清晰显示小关节、韧带钙化和骨细节 | **针对性检查** | 复杂关节受累（如髋关节）或X光不明确的病变 |

| **MRI** | 评估骨髓水肿、滑膜炎症、软骨损伤 | **早期诊断关键** | 疑似早期AS或需要判断炎症活动度时 |

#### **2. 抽血化验**

| **项目** | **作用** | **必要性** | **适用情况** |

|-----------------|---------------------------|------------|-----------------------------|

| **HLA-B27** | 风险筛查 | **推荐** | 有家族史或典型症状者 |

| **CRP/ESR** | 评估炎症活动度 | **推荐** | 判断病情轻重或治疗效果 |

| **血常规、肝肾功能** | 排除其他疾病或药物副作用 | **常规** | 初次就诊或长期用药者 |

| **ANA、RF** | 排除类风湿性关节炎 | **选择性** | 关节肿痛对称或多关节受累时 |

| **肠道/泌尿检查** | 排查炎症性肠病或感染 | **针对性** | 伴腹泻、腹痛或尿路症状时 |

2.是否需要全部完成？**

**根据病情阶段决定**：

- **早期AS**：MRI和炎症指标（CRP/ESR）是核心，X光/CT可能正常，无需过度检查。

- **中晚期AS**：X光/CT用于评估关节破坏程度，MRI仍可观察炎症活动。

**根据医生目的判断**：

- **明确诊断**：MRI和HLA-B27是关键，抽血化验排除其他疾病。

- **评估病情**：CRP/ESR和影像学（如MRI）监测炎症活动及进展。

- **个体化治疗**：肝肾功能和血常规为用药安全提供依据。

**您的权利**：

- **可要求医生解释**：每项检查的必要性及优先级。

- **分步完成**：若经济或时间有限，可先完成核心检查（如MRI+HLA-B27+CRP/ESR），再根据结果决定是否补充其他项目。

3.常见疑问解答**

#### **Q1：MRI和X光哪个更重要？**

- **答案**：

**MRI更早发现炎症**（如骨髓水肿），但X光能显示骨结构改变（如关节间隙狭窄）。两者互补，**早期诊断优先MRI，中晚期评估X光**。

#### **Q2：抽血化验是否都能用医保？**

- **答案**：

HLA-B27、CRP/ESR等检查通常医保覆盖，但需提前确认。部分医院可能要求自费，可咨询收费处。

#### **Q3：检查有辐射风险吗？**

- **答案**：

X光和CT有微量辐射，但单次检查风险极低，医生会权衡利弊。孕妇或儿童需谨慎，但成人无需过度担心。

4.建议行动**

**与医生沟通**：

- 明确医生开检查的**具体目的**（如“排除RA”或“评估AS活动度”）。

- 询问是否有**替代方案**（如超声代替CT，或短期复查代替初次MRI）。

**优先完成核心检查**：

- **必选**：骶髂关节MRI、HLA-B27、CRP/ESR、血常规。

- **可选**：X光/CT根据症状决定，肠道/泌尿检查仅在有相关症状时进行。

**拒绝不必要的检查**：

- 如无关节肿痛，ANA/RF可能无需优先检查。

- 若无肠道症状，炎症性肠病相关检测可暂缓。

**经济考量**：

- 部分医院支持**分阶段检查**，先完成关键项目，根据结果再补充。

- 咨询医保政策，避免重复检查费用。

5.总结**

- **并非所有检查都必需**，但医生开具的方案通常基于临床经验，目的是**不漏诊、不误诊**。

- **您的参与至关重要**：通过沟通明确需求，结合自身情况（如症状、经济条件）调整检查顺序。

- **早期诊断价值最高**：若怀疑AS，优先完成MRI和HLA-B27检测，其他检查可后续补充。

**最后提醒**：拒绝检查可能延误诊断，但过度检查也可能增加焦虑和经济负担。请在医生指导下理性选择，必要时寻求第二意见。

1. *

1.AS的发病年龄特点**

**典型发病年龄段**：

AS主要见于**青少年和年轻成人（15-30岁）**，但**任何年龄都可能发病**，包括30岁以上甚至中老年人。

- **儿童AS**：称为“幼年特发性关节炎（JIA）”，多在16岁前起病，男孩更常见。

- **成人AS**：30岁后起病者占一定比例，症状可能更隐匿，易被误诊。

**晚发性AS的可能性**：

- 部分患者可能因**炎症控制不佳**或**误诊**，直到中年才被确诊。

- **女性AS**：女性发病率较低，但症状可能更轻，易延迟诊断。

2.成年后患AS的常见原因**

**遗传因素**：

- **HLA-B27阳性**是重要风险因素（约90% AS患者携带）。

- 家族中有AS病史者，无论年龄均需警惕。

**免疫系统异常**：

- 成年人免疫力波动（如感染、压力）可能触发AS-like炎症。

**其他关联疾病**：

- **炎症性肠病（IBD）**：克罗恩病或溃疡性结肠炎患者AS风险增加。

- **反应性关节炎**：肠道或泌尿系感染后可能诱发AS。

3.成年AS的症状特点**

与青少年相比，成人AS可能表现不同：

| **症状** | **青少年AS** | **成人AS** |

|------------------|----------------------------|-----------------------------|

| **首发症状** | 下腰背痛、晨僵 | 腰痛、髋关节或足跟疼痛 |

| **受累关节** | 骶髂关节、脊柱 | 髋关节、腰椎、胸椎 |

| **进展速度** | 较快，易致关节畸形 | 较慢，但可能累积内脏病变 |

| **伴随症状** | 眼炎、皮疹较少见 | 更易合并高血压、心血管疾病 |

4.如何诊断成年AS？**

**核心检查**：

- **骶髂关节MRI**：早期发现骨髓水肿和滑膜炎症（金标准）。

- **HLA-B27检测**：阳性率约90%，但阴性者也不能排除AS。

- **炎症指标**：CRP、ESR升高提示活动性炎症。

**影像学特点**：

- **成人AS的X光表现**：关节间隙狭窄、骨赘形成（中晚期特征）。

- **MRI优势**：早期捕捉滑膜炎症，避免漏诊。

**鉴别诊断**：

- **机械性腰痛**：如腰椎间盘突出、肌腱炎。

- **其他脊柱关节炎**：银屑病性关节炎、反应性关节炎。

- **类风湿性关节炎（RA）**：RA多累及手部小关节，对称性肿痛。

5.成年AS的治疗与管理**

**早期干预是关键**：

- 即使年龄较大，**生物制剂（如TNF-α抑制剂）**仍可有效控制炎症，延缓关节破坏。

**生活方式调整**：

- **适度运动**：游泳、瑜伽、太极拳改善关节灵活性。

- **避免久坐**：每30分钟起身活动，减轻腰椎压力。

- **戒烟**：吸烟会加速脊柱融合，增加残疾风险。

**定期随访**：

- 监测炎症指标（CRP/ESR）和影像学变化，评估治疗效果。

- 心脏、眼部并发症筛查（如主动脉炎、虹膜炎）。

十八、

1.AS的典型特征与误诊风险**

强直性脊柱炎主要累及**脊柱和骶髂关节**，以**慢性炎症性疼痛、晨僵、活动后缓解**为特点。但由于其早期症状不典型或与其他疾病表现重叠，易被误诊。以下是常见误诊疾病及鉴别要点：

2.易被误诊的疾病**

#### **1. 机械性腰背痛（如腰椎间盘突出、肌腱炎）**

- **相似点**：下腰背痛、活动受限。

- **区别**：

- **AS的疼痛**：夜间加重、晨僵＞1小时、活动后缓解。

- **机械性疼痛**：与体位或动作相关（如弯腰、提重物），休息后缓解，无晨僵。

- **检查重点**：

- MRI或CT可发现椎间盘突出或肌肉劳损，但AS的骶髂关节炎症在影像学上更明显。

3.其他脊柱关节炎**

- **反应性关节炎**

- **相似点**：脊柱或外周关节炎症，可能伴随感染史（如腹泻、尿道炎）。

- **区别**：反应性关节炎多呈**自限性**，炎症消退后关节功能恢复，而AS为慢性持续性炎症。

- **银屑病性关节炎（PsA）**

- **相似点**：关节肿痛、晨僵，部分患者合并银屑病皮疹。

- **区别**：PsA多累及**手部小关节**（对称性），而AS主要影响脊柱和骶髂关节。

- **炎症性肠病相关关节炎（IBD-A）**

- **相似点**：肠道症状（腹泻、腹痛）伴关节炎症。

- **区别**：IBD-A关节病变多为**非对称性**，而AS的骶髂关节受累更典型。

类风湿性关节炎（RA）**

- **相似点**：慢性关节炎、晨僵、血沉/CRP升高。

- **区别**：

- **RA**：多累及**手部小关节**（掌指关节、近端指间关节），呈**对称性**肿痛。

- **AS**：主要影响**脊柱和大关节**（如髋关节），不对称。

- **实验室检查**：RA患者RF和抗CCP抗体阳性，而AS患者多为HLA-B27阳性。

感染性疾病（如结核性骶髂关节炎、布鲁氏菌病）**

- **相似点**：骶髂关节疼痛、发热、血沉升高。

- **区别**：

- **感染性关节炎**：常有明确感染史（如结核接触史、畜牧业暴露），需通过**PPD试验、病原体培养**或**PCR**确诊。

- **AS**：无感染源，HLA-B27阳性率高。

#### ** 代谢性或骨肿瘤性疾病**

- **骨质疏松性骨折**

- **相似点**：突发腰背剧痛，活动受限。

- **区别**：X线/MRI可见骨折线，无炎症表现（如CRP正常）。

- **骨肿瘤（如骨髓瘤、转移癌）**

- **相似点**：骨痛、夜间加重，影像学可见占位性病变。

- **区别**：AS无肿瘤标志物升高或骨破坏特征（如溶骨性改变）。

4.误诊的主要原因**

**早期症状不典型**：AS早期可能仅表现为腰痛或髋痛，未累及骶髂关节。

**依赖单一检查**：仅凭X光（早期正常）或CRP/ESR（非特异性）易漏诊。

**临床表现重叠**：如合并银屑病或肠道症状时，可能被归为其他疾病。

5.如何避免误诊？**

**完善检查**：

- **骶髂关节MRI**：AS早期诊断的金标准，可发现骨髓水肿和滑膜炎症。

- **HLA-B27检测**：阳性率高达90%，但需结合其他检查。

- **炎症指标**：CRP/ESR升高提示炎症活动，但需排除感染。

**专科就诊**：

- **风湿免疫科**：综合症状、体征和检查结果，制定诊断流程。

**动态随访**：

- 若症状持续（如腰痛＞3个月），需定期复查影像学和实验室指标。

十九、

1.先冷静分析：可能的原因**

即使多次被排除AS，您的症状仍可能由以下原因导致：

**AS早期未显影**：

- 骶髂关节MRI早期可见骨髓水肿和滑膜炎症（AS金标准），但若仅凭X光或CT可能漏诊。

**非典型AS表现**：

- 部分患者以髋关节、胸椎或颈椎受累为主，而非典型下腰背痛。

**其他脊柱关节炎**：

- 如反应性关节炎、银屑病性关节炎、炎症性肠病相关关节炎（IBD-A）。

**机械性或非炎症性疾病**：

- 腰肌劳损、腰椎间盘突出、骨质疏松、纤维肌痛症等。

**心理因素**：

- 长期焦虑可能放大躯体症状（如慢性疼痛综合征）。

2.建议行动：下一步诊疗计划**

#### ** 重新评估：寻找专科医生**

- **优先选择风湿免疫科**：

- AS属于风湿免疫病，需专科医生系统评估。若当前医生经验不足，可寻求**三甲医院风湿免疫科**或**AS诊疗中心**就诊。

- **要求详细问诊**：

- 记录症状日记（如疼痛时间、晨僵时长、活动后缓解情况），提供给医生参考。

#### ** 完善关键检查（尤其影像学）**

- **骶髂关节MRI**：

- AS早期诊断的核心检查，明确骨髓水肿、滑膜炎症及软骨损伤。

- **脊柱动态X线/MRI**：

- 观察腰椎活动度（如Schober试验）和椎间盘炎症。

- **超声检查**：

- 动态评估关节积液、肌腱炎等软组织病变。

#### ** 排除其他疾病**

- **感染性疾病**：

- 结核、布鲁氏菌病等可能导致骶髂关节痛，需通过PPD试验、血培养等排查。

- **代谢性骨病**：

- 骨质疏松、甲状旁腺功能亢进等，需检查骨密度、血钙磷代谢。

- **肿瘤或血液病**：

- 若疼痛剧烈或夜间加重，需排查骨髓瘤、转移癌（通过肿瘤标志物、PET-CT）。

#### **考虑HLA-B27阴性AS**

- **即使HLA-B27阴性**，仍可能有AS（约10%-20%患者为阴性）。

- 需结合临床症状和影像学结果综合判断。

3.若仍无法确诊：怎么办？**

**动态随访**：

- 每3-6个月复查骶髂关节MRI、CRP/ESR，观察病情变化。

**对症治疗**：

- **非甾体抗炎药（NSAIDs）**：缓解疼痛和炎症（如塞来昔布、布洛芬）。

- **物理治疗**：热敷、理疗、游泳/瑜伽改善关节灵活性。

**多学科会诊（MDT）**：

- 邀请风湿科、骨科、康复科医生共同讨论，制定个体化方案。

**心理支持**：

- 慢性疼痛和焦虑可能互为因果，必要时寻求心理咨询或抗焦虑药物。

4.常见误区与澄清**

#### **误区1：“所有AS患者HLA-B27都阳性”**

- **真相**：约10%-20% AS患者HLA-B27阴性，需结合其他检查（如MRI、CRP）诊断。

#### **误区2：“X光正常就排除AS”**

- **真相**：AS早期X光可能正常，MRI是更敏感的检查。

#### **误区3：“AS只能诊断AS”**

- **真相**：需排除其他脊柱关节炎（如银屑病性关节炎、反应性关节炎）。

二十、

1.首要原则：勿自行用药，优先明确病因**

虽然疼痛需要及时缓解，但**在未确诊前盲目用药可能掩盖病情或引发副作用**。建议：

**尽快就医**：

- 完善关键检查（如骶髂关节MRI、HLA-B27、CRP/ESR），明确疼痛原因（AS或其他疾病）。

- 若疼痛剧烈或持续加重，需急诊排除急性损伤、感染或肿瘤。

2.临时缓解疼痛的可行方案**

在等待确诊期间，可通过以下方式**安全缓解症状**：

#### **非甾体抗炎药（NSAIDs）**

- **适用情况**：

轻中度疼痛（如腰背钝痛、关节肿痛），需短期使用（≤7-10天）。

- **常用药物**：

- 布洛芬（400mg，每日2-3次，餐后服用）

- 塞来昔布（200mg，每日1-2次）

- 美洛昔康（15mg，每日1次）

- **注意事项**：

- 避免长期使用（可能引起胃肠道出血、肾功能损伤）。

- 对乙酰氨基酚（扑热息痛）可作为替代（每日≤3g），但无抗炎作用。

#### **局部外用药**

- **药物类型**：

- 非甾体抗炎药软膏（如双氯芬酸钠凝胶）

- 冷敷贴或热敷包（急性疼痛冷敷，慢性疼痛热敷）

- **优点**：

直接作用于局部，全身副作用小。

#### **肌松剂（需医生指导）**

- **适用情况**：

- 若疼痛伴肌肉痉挛（如腰肌劳损），可短期联用肌松药（如盐酸乙哌立松）。

- **注意**：

避免长期使用，可能引起乏力、头晕。

3.避免使用的药物**

**糖皮质激素**：

- 如泼尼松，虽能快速抗炎，但长期使用会导致骨质疏松、感染风险增加，仅限医生评估后短期使用。

**免疫抑制剂或生物制剂**：

- 如甲氨蝶呤、TNF-α抑制剂，**仅适用于确诊AS或类风湿关节炎等自身免疫病**，未确诊时滥用可能加重潜在感染或过敏反应。

**强效镇痛药（如阿片类）**：

- 除非疼痛无法控制且明确病因，否则避免使用（易成瘾、掩盖病情）。

4.非药物治疗建议**

**物理治疗**：

- 热敷、低频电疗、康复训练（如游泳、瑜伽）缓解肌肉紧张。

**生活方式调整**：

- 避免久坐、弯腰提重物，使用符合人体工学的座椅。

- 戒烟（吸烟会加重炎症和疼痛）。

**心理支持**：

- 慢性疼痛可能导致焦虑，可通过冥想、深呼吸或心理咨询缓解压力。

---

5、何时需立即就医？**

出现以下情况时需**紧急就诊**：

- 突发剧烈疼痛伴发热、红肿（警惕感染或痛风性关节炎）。

- 外伤后疼痛加重或出现神经压迫症状（如腿麻、大小便失禁）。

- 夜间痛醒频繁，无法通过休息缓解。

二十一、

1.常见影像学检查的辐射剂量**

不同检查的辐射剂量差异较大，以下为**典型值参考**（单位：毫希沃特，mSv）：

| **检查类型** | **单次辐射剂量** | **对比参考** |

|--------------------|------------------|----------------------------------|

| **胸部X光** | 0.02 - 0.1 mSv | ≈ 日常环境辐射（1天≈0.001-0.008 mSv） |

| **腰椎X光** | 0.1 - 0.3 mSv | |

| **骶髂关节CT** | 3 - 8 mSv | ≈ 胸部CT的1/3-1/2 |

| **全身CT扫描** | 10 - 20 mSv | 高风险（需严格评估必要性） |

| **MRI** | **0 mSv** | 无辐射 |

| **超声** | **0 mSv** | 无辐射 |

2.辐射对健康的潜在影响**

**低剂量辐射（＜10 mSv）**：

- **风险极低**：目前无明确证据表明单次低剂量辐射（如单次CT）会增加癌症或遗传疾病风险。

- **累积效应**：若短期内多次接受高剂量检查（如多次CT），风险可能上升，但医疗场景下通常严格把控。

**高剂量辐射（＞20 mSv）**：

- **需谨慎**：可能与癌症风险轻度升高相关（如乳腺癌、甲状腺癌），但医生会权衡诊断获益与风险。

3.、如何降低辐射风险？**

**优先选择无辐射检查**：

- **MRI或超声**：AS早期诊断首选MRI，可避免辐射。

- **X光**：用于骨结构评估（如关节间隙狭窄），但需控制次数。

**减少CT检查频率**：

- **仅在必要时使用**：如MRI无法明确病灶时，可选择CT。

- **优化扫描参数**：医生可通过调整设备设置降低辐射剂量。

**防护措施**：

- **铅屏蔽**：检查时穿戴铅围裙或护颈，保护生殖器、甲状腺等敏感部位。

- **缩短检查时间**：确保CT扫描时间尽可能短。

**替代方案**：

- **实验室检查**：如HLA-B27、CRP/ESR可辅助诊断，减少影像学依赖。

- **动态观察**：对早期AS患者，可定期复查MRI而非频繁CT。

4：影像学检查是诊断AS的重要工具，但合理选择和规范操作可最大限度保障安全。如有疑虑，可与医生讨论是否需要调整检查计划！

二十二、*

1.HLA-B27阳性的意义与局限性**

**HLA-B27的关联性与非特异性**

- **约90%的AS患者HLA-B27阳性**，但**并非所有阳性者都会患AS**。

- HLA-B27也可与其他疾病相关（如反应性关节炎、银屑病性关节炎、炎症性肠病），需结合其他检查排除。

**诊断AS需综合评估**

- 单纯HLA-B27阳性**无法确诊AS**，必须结合临床症状、影像学和实验室检查。

2.血常规异常的可能原因**

**与AS本身无关的异常**

- **慢性炎症反应**：长期炎症可能导致轻度贫血、白细胞升高或血小板增多，但无特异性。

- **药物副作用**：如非甾体抗炎药（NSAIDs）可能引起胃肠道出血或肝肾功能异常。

**需警惕其他疾病**

- **感染性疾病**：如结核、布鲁氏菌病可能导致血常规异常（如淋巴细胞升高、CRP显著升高）。

- **自身免疫病**：如类风湿性关节炎（RA）、系统性红斑狼疮（SLE）可能伴随血常规异常（如血小板减少、贫血）。

- **血液系统疾病**：如白血病、骨髓瘤等也可能导致血常规明显异常。

3.症状符合AS但未确诊的原因**

**AS的典型症状可能不典型**

- **早期AS**：可能仅表现为髋关节或胸椎疼痛，而非典型下腰背痛。

- **非对称性受累**：部分患者以外周关节（如膝、踝）肿痛为主，易误诊为RA。

**需排除其他脊柱关节炎**

- **反应性关节炎**：继发于感染（如腹泻、尿道炎），HLA-B27阳性但病程短暂，可自愈。

- **银屑病性关节炎（PsA）**：合并银屑病皮疹或指甲改变，关节受累多对称。

- **炎症性肠病相关关节炎（IBD-A）**：伴肠道症状（腹痛、腹泻），需肠镜确诊。

**影像学检查未达AS标准**

- **骶髂关节MRI**：AS早期可见骨髓水肿和滑膜炎症，但若仅显示轻微异常或正常，可能无法确诊。

- **X光/CT**：中晚期AS可见关节间隙狭窄、骨赘，但早期可能正常。

4.医生未确诊的可能考量**

. **证据不足**：

- **症状不典型**：如疼痛部位、晨僵时长未达AS标准（晨僵＜1小时）。

- **影像学不支持**：骶髂关节MRI未见明显炎症或骨破坏。

- **实验室检查矛盾**：如CRP/ESR正常，但血常规异常可能与AS无关。

**需排除其他疾病**：

- **机械性腰背痛**：如腰椎间盘突出、肌腱炎，需影像学（MRI/CT）鉴别。

- **感染或肿瘤**：如骶髂关节结核、骨髓瘤，需病原体检测或PET-CT排查。

**动态观察**：

- **早期AS可能漏诊**：若症状持续但影像学正常，医生可能建议随访（每3-6个月复查MRI）。

5.下一步建议**

**完善关键检查**：

- **骶髂关节MRI**：明确骨髓水肿、滑膜炎症及软骨损伤（AS金标准）。

- **炎症指标**：CRP、ESR、血沉，评估炎症活动度。

- **肠道/泌尿检查**：如便常规、尿培养，排除感染或IBD。

- **ANA、RF、抗CCP抗体**：排除RA或其他自身免疫病。

**专科就诊**：

- **风湿免疫科**：综合评估症状、体征和检查结果。

- **骨科或影像科**：复查影像学，必要时行骶髂关节超声或CT。

**对症处理**：

- **短期缓解疼痛**：非甾体抗炎药（如塞来昔布）改善症状。

- **物理治疗**：热敷、游泳、瑜伽缓解肌肉紧张。

**动态随访**：

- 若症状持续进展，需重新评估影像学和实验室检查。

二十三、

1.关于残疾风险的客观事实

**病程差异显著**：

- 约30%患者表现为轻度（晨僵<30分钟，活动后缓解）

- 50%中度（需药物干预控制）

- 20%重度（出现竹节样脊柱、髋关节强直）

**致残风险要素**：

- 晚期髋关节受累（发生率约30-50%）

- 颈椎关节病变（10-20%患者出现）

- 快速进展型病程（5年内关节破坏超过50%）

**现代治疗进展**：

- 生物制剂（如TNF-α抑制剂）可使90%以上患者达到疾病活动度低谷

- JAK抑制剂对传统治疗无效者有效率提升至65%

- 骶髂关节MRI早期诊断准确率达92%

2.二、三级预防体系构建

**第一级预防（确诊后）**

- **影像学监测**：每6个月拍摄骨盆正位片，使用BASDAI评分系统评估活动度

- **生物标志物跟踪**：定期检测CRP、ESR、HLA-B27水平及肠道菌群 composition

- **运动处方**：每日进行30分钟水中运动（减少关节负荷）+10分钟脊柱伸展训练

**第二级预防（症状活跃期）**

- **靶向治疗方案**：

- 初始治疗：MTX（甲氨蝶呤）+NSAIDs（塞来昔布200mg qd）

- 疗效不佳时：转换为生物制剂（依那西普40mg qw）

- 生物制剂耐药：联合IL-17抑制剂（司库奇尤单抗30mg qw）

- **微创介入治疗**：对于顽固性髋关节疼痛，可考虑关节腔注射糖皮质激素（曲安奈德40mg/mL）

**第三级预防（功能维持期）**

- **智能康复设备**：佩戴动态脊柱矫形器（每天8小时）

- **机器人辅助训练**：步态训练机器人（每周3次，每次30分钟）

- **职业康复**：根据ASODI指数（AS功能障碍指数）调整工作环境

3.生活质量提升策略

**日常活动优化**：

- 使用电动开瓶器、长柄取物夹等辅具

- 改造卫生间（安装扶手、防滑垫）

- 采用坐姿穿衣、电动剃须刀等省力技巧

**睡眠姿势管理**：

- 侧卧时双膝间夹薄枕（保持脊柱中立位）

- 使用记忆棉床垫（厚度5-8cm）

- 睡前进行10分钟猫牛式瑜伽序列

**营养支持方案**：

- 地中海饮食模式（ω-3摄入≥1.5g/d）

- 补充维生素D（维持血25(OH)D>50ng/mL）

- 益生菌制剂（含双歧杆菌、乳酸杆菌，每日1×10^9 CFU）

4.新兴治疗进展

**基因治疗**：

- IL-17A单克隆抗体皮下制剂（每2周给药一次）

- CRISPR编辑技术调节IL-23/IL-17通路（临床II期试验中）

**数字医疗技术**：

- 人工智能监测系统（通过可穿戴设备实时分析步态参数）

- 远程康复平台（VR模拟游泳、太极等运动场景）

**再生医学**：

- 脐带间充质干细胞移植（临床试验显示骶髂关节炎症改善率78%）

- 3D打印人工椎间盘（适用于终末期脊柱融合患者）

5.心理建设与支持系统

**认知行为疗法（CBT）**：

- 每月1次团体心理辅导

- 使用ACT（接纳承诺疗法）处理疾病焦虑

**社会资源整合**：

- 申请残疾人认证（符合GB/T 26341-2010标准者）

- 加入中国脊柱关节炎联盟患者社群（每月线上讲座）

**家庭支持计划**：

- 开展家属护理培训（每周2小时工作坊）

- 建立家庭医疗档案（包含用药记录、检查报告）

二十四、

1.治疗周期的阶段性特征

**急性期控制**（0-12个月）：

- **NSAIDs**：症状发作时短期使用（通常≤14天），如塞来昔布200mg qd

- **糖皮质激素**：严重炎症时短期冲击治疗（泼尼松龙0.5mg/kg/d，2周后减量）

- **传统DMARDs**：MTX起始剂量7.5mg/w，2-3个月起效

**达标维持期**（1-3年）：

- 生物制剂需持续使用至CRP<1mg/L、ESR<20mm/h

- JAK抑制剂疗程建议≥6个月（托法替布5mg qd）

**缓解期管理**（3年后）：

- 逐步减量生物制剂（如每3个月减20%剂量）

- 转换为MTX+NSAIDs维持方案

- 部分患者可停药（需满足以下条件）：

- BASDAI评分<1.5

- 脊柱活动度（Schober试验）>5cm

- 无新发关节肿胀

2.停药决策的客观指标

| 指标类型 | 达标标准 | 监测频率 |

|-----------------|-----------------------------------|--------------|

| 炎症标志物 | CRP<1mg/L，ESR<20mm/h | 每4周 |

| 影像学稳定 | 骶髂关节MRI脂肪抑制T2WI无新发渗出 | 每6个月 |

| 功能评估 | Berg平衡量表>40分 | 每3个月 |

| 症状控制 | 夜间痛醒次数≤1次/月 | 每月自评 |

3.影响治疗时长的关键因素

**疾病活动度评分**：

- ASDAS-CRP≥2.1分需强化治疗

- 达标患者平均停药时间缩短40%（vs未达标者）

**生物制剂类型**：

- TNF-α抑制剂：平均维持期3.2年

- IL-17抑制剂：平均维持期2.8年

- JAK抑制剂：平均维持期4.1年

**并发症管理**：

- 合并虹膜炎者治疗周期延长2.5倍

- 髋关节受累患者需终身用药概率增加37%

4.停药后复发预警信号

出现以下情况需立即重启治疗：

- 晨僵时间≥1小时（较基线增加50%）

- 疼痛视觉模拟评分（VAS）≥4分

- CRP上升>0.5mg/L

- 新发关节肿胀≥2个

5.长期管理建议

**停药后监测**：

- 每3个月复查血常规、肝肾功能

- 每年进行脊柱全长X线及髋关节MRI

**复发应急预案**：

- 备用口服生物制剂（如英夫利昔单抗预充剂型）

- 自备便携式CRP检测仪（监测炎症波动）

**生活方式干预**：

- 每日摄入ω-3脂肪酸≥1.5g（相当于三文鱼100g）

- 保持BMI≤24kg/m²（可降低复发风险42%）

6.请与主治医生共同制定动态调整方案，建议每3个月进行一次治疗评估。记住：坚持规范治疗的患者，85%以上可有效控制病情，避免残疾，部分患者甚至能达到临床治愈状态。

二十五、

1.疫苗接种的基本原则

**风险评估四象限法**：

- **X轴**：药物免疫抑制强度（从低到高：NSAIDs < MTX < 生物制剂 < JAK抑制剂）

- **Y轴**：疫苗类型风险（灭活疫苗<重组蛋白疫苗<mRNA疫苗<活疫苗）

- **决策区域**：高风险区（如JAK抑制剂+活疫苗）需严格禁忌，中低风险区需个体化评估

**国际指南共识**：

- EULAR 2022建议：AS患者接种疫苗应与风湿科医生及感染科医生共同决策

- ACRA 2021指南：明确生物制剂使用者需推迟减毒活疫苗至少14周

2.常用药物对疫苗的影响机制

| 药物类别 | 典型药物 | 免疫影响靶点 | 疫苗风险等级 | 建议调整 |

|----------------|---------------|-----------------------|------------|-------------------------|

| DMARDs | 甲氨蝶呤(MTX) | DNA合成抑制 | 中度 | 灭活疫苗无需推迟 |

| | 磺胺嘧啶银 | T细胞活化抑制 | 高度 | 避免活疫苗 |

| 生物制剂 | 抗TNF-α制剂 | TNF-γ受体阻断 | 高度 | mRNA疫苗需谨慎 |

| | 抗IL-17制剂 | IL-17/IL-23通路抑制 | 中度 | 重组蛋白疫苗可接种 |

| JAK抑制剂 | 托法替布 | JAK-STAT信号通路抑制 | 极高度 | 禁用所有活疫苗 |

| NSAIDs | 塞来昔布 | COX-2选择性抑制 | 低度 | 正常接种 |

3.疫苗类型接种建议

**安全推荐疫苗**：

- **灭活疫苗**：流感疫苗（每年接种）、肺炎链球菌疫苗（PCV13/23）、新冠灭活疫苗

- **重组蛋白疫苗**：乙肝疫苗、HPV疫苗（9价）、带状疱疹疫苗（Shingrix）

- **mRNA疫苗**：需根据药物作用机制评估（如抗TNF-α制剂可能降低效力）

**暂缓接种疫苗**：

- **活疫苗**：麻疹腮腺风疹疫苗（MMR）、水痘疫苗、卡介苗

- **减毒活疫苗**：带状疱疹减毒疫苗（ZVL）

** 特殊情况处理**：

- **COVID-19 mRNA疫苗**：

- 使用抗TNF-α制剂者：建议完成全程接种后继续用药

- 使用JAK抑制剂者：需在停药至少3天后接种第一剂

- **HPV疫苗**：建议在生物制剂停药6个月后接种以确保免疫原性

4.接种时机优化策略

```mermaid

graph TD

A[疾病活动度评估] --> B{CRP<1mg/L?}

B -->|是| C[选择灭活/重组疫苗]

B -->|否| D[推迟至炎症控制后]

C --> E[评估药物半衰期]

E --> F{药物清除期≥5个半衰期?}

F -->|是| G[正常接种]

F -->|否| H[延长间隔至安全期]

```

5.紧急情况处理预案

**意外暴露风险**：

- 麻疹暴露：立即注射免疫球蛋白（IVIG），即使已接种疫苗

- 乙型肝炎暴露：HBIG联合疫苗接种（0/7/21天方案）

**疫苗相关不良反应**：

- 局部反应（红肿>5cm）：使用冷敷+NSAIDs对症处理

- 系统性反应（发热>38.5℃）：暂停生物制剂，待恢复后重启

6.患者自我管理建议

**建立疫苗接种档案**：

- 记录每种疫苗类型、接种时间、药物使用周期

- 使用AS Vaccine Tracker App（iOS/Android可下载）

**旅行前免疫规划**：

- 热带地区旅行：提前2周接种甲肝疫苗、流脑疫苗

- 高海拔地区：接种流感和肺炎疫苗（降低呼吸道感染风险）

**与疫苗接种机构的沟通要点**：

- 提供完整用药清单（包括仿制药名称）

- 说明疾病活动度控制情况（如近期CRP水平）

二十六

1.药理机制的核心差异

| 药物类别 | 作用靶点 | 核心功能 | 典型药物（AS常用） |

|----------------|---------------------------|------------------------------|----------------------------|

| **抗炎药** | 炎症介质调控 | 抑制炎症级联反应（如IL-6、TNF-α） | MTX、生物制剂（依那西普）、IL-17抑制剂 |

| **消炎药** | 微生物感染控制 | 杀灭病原体或抑制其增殖 | 抗生素（如甲硝唑）、抗病毒药 |

| **止疼药** | 神经信号传递干预 | 阻断痛觉感知或减轻疼痛信号 | NSAIDs（塞来昔布）、阿片类药物（曲马多） |

2.临床应用场景对比

** 抗炎药（疾病控制核心）**

- **作用**：抑制滑膜增生、骨侵蚀，降低关节破坏风险

- **AS治疗地位**：

- 一线药物：MTX（7.5mg/w）联合NSAIDs

- 生物制剂：达标治疗的核心（如阿达木单抗40mg qw）

- **炎症标志物改善**：

- CRP下降幅度：生物制剂组较MTX组多35%（NEJM 2021）

- 骶髂关节MRI炎症渗出减少率：68%（12周治疗后）

** 消炎药（感染并发症管理）**

- **使用场景**：合并肠道感染（如艰难梭菌）、呼吸道感染

- **AS患者特殊性**：

- 因免疫抑制剂使用，感染风险增加2-3倍

- 常见抗生素：环丙沙星（覆盖革兰氏阴性菌）、利福平（结核预防）

** 止疼药（症状缓解工具）**

- **阶梯用药原则**：

- 一线：NSAIDs（塞来昔布200mg qd）

- 二线：弱阿片类（曲马多50mg prn）

- 三线：强阿片类（羟考酮纳洛酮缓释片10mg q12h）

- **疼痛控制目标**：

- VAS评分≤3分（夜间痛醒≤1次/月）

- Schober试验改善≥2cm（3个月内）

3.药物相互作用警示

| 药物组合 | 相互作用机制 | AS患者风险等级 | 管理建议 |

|-------------------------|-----------------------------|--------------|------------------------------|

| MTX + NSAIDs（如塞来昔布） | 增加胃肠道出血风险3倍 | 高危 | 联合用药时需加用PPI（奥美拉唑20mg qd） |

| 生物制剂 + 利福平 | 降低生物制剂浓度40-60% | 极高危 | 需调整生物制剂剂量（如依那西普增至60mg qw） |

| 阿片类药物 + 地高辛 | 增加中枢性呼吸抑制风险 | 中危 | 监测血药浓度，调整剂量 |

4.患者自我管理建议

**用药记录工具**：

- 使用Medication Tracker App记录：

- 抗炎药：服用时间、剂量、炎症指标变化

- 止疼药：疼痛诱因、VAS评分、服药频次

**不良反应监测**：

- MTX使用者：每周监测肝功能（ALT/AST）、血常规

- NSAIDs使用者：注意胃肠道症状（黑便、腹痛）

- 阿片类药物：警惕便秘（每日排便≤3次需干预）

**药物调整阈值**：

- NSAIDs连续使用>14天需评估疗效

- 止疼药升级至二线药物需风湿科医生评估

- 生物制剂疗效不佳时（ASDAS-CRP≥2.1）需转换靶点药物

二十七、

1.NSAIDs类药物的剂量安全阈值

| 药物名称 | 日最大剂量（成人） | 超量风险 | 疼痛未缓解时的正确处理 |

|---------------|------------------|------------------------------|----------------------------------|

| 塞来昔布 | 400mg/d（200mg×2） | 胃肠穿孔风险↑3倍，心血管事件风险↑1.5倍 | 立即联系医生，评估是否需要升级治疗方案 |

| 布洛芬 | 2400mg/d（800mg×3） | 胃溃疡风险↑4倍，肾功能异常风险↑2倍 | 同步使用胃黏膜保护剂（如铝碳酸镁） |

| 双氯芬酸钠 | 150mg/d（75mg×2） | 肝损伤风险↑5倍 | 考虑转换为选择性COX-2抑制剂（如艾瑞昔布） |

2.疼痛控制不佳的临床应对路径

```mermaid

graph TD

A[当前疼痛评分≥4分(VAS)] --> B{是否达到药物最大耐受剂量?}

B -->|是| C[立即就医评估]

B -->|否| D[检查用药依从性]

D --> E[漏服/减量?]

E -->|是| F[调整给药频率]

E -->|否| G[排查诱因（如感冒、疲劳）]

G --> H[物理治疗+热敷干预]

H --> I[24小时后仍无改善?]

I -->|是| J[急诊处理]

I -->|否| K[维持当前方案观察]

```

3.升级疼痛管理的医学策略

**多模式镇痛方案**：

- **基础治疗**：NSAIDs（塞来昔布200mg qd）+ 局部冷敷（每次15分钟，每日3次）

- **强化治疗**：加用弱阿片类药物（曲马多50mg prn，每日≤4次）

- **终极方案**：转诊疼痛科行脊髓电刺激植入术（有效率72%）

**药物转换技巧**：

- NSAIDs之间转换需间隔≥72小时（避免叠加肾毒性）

- 对乙酰氨基酚日剂量勿超3000mg（肝功能异常者≤2000mg）

**非药物干预措施**：

- 水中运动（减少关节负重，疼痛缓解率68%）

- 经皮神经电刺激（TENS，每周3次，每次30分钟）

- 呼吸训练（腹式呼吸改善胸廓活动度，VAS评分↓35%）

4.特殊人群用药警戒

** 胃肠高风险患者**（如合并慢性胃炎）：

- 禁用布洛芬、萘普生

- 优先选择艾瑞昔布（COX-2选择性抑制剂）

- 必须联用质子泵抑制剂（奥美拉唑20mg qd）

** 肾功能不全者**（eGFR<60ml/min/1.73m²）：

- 塞来昔布禁用（肾毒性风险↑7倍）

- 改用对乙酰氨基酚（肌酐清除率调整剂量）

- 监测尿蛋白（每日尿量<1000ml需警惕急性肾损伤）

** 心血管高危人群**：

- 罗非昔布禁用（心肌梗死风险↑3倍）

- 优先选择萘丁美酮（心血管事件风险最低的NSAIDs）

- 必须监测血压（目标<130/80mmHg）

5.紧急情况处理规范

** 急性剧烈疼痛（VAS≥7分）**：

- 立即口服曲马多100mg

- 同时冰敷疼痛部位

- 1小时内未缓解需急诊处理

** 药物过量风险**：

- 塞来昔布单次误服>800mg需立即洗胃

- 对乙酰氨基酚血药浓度>15μg/mL启动N-乙酰半胱氨酸治疗

6.患者自我监测工具

**疼痛日记模板**：

```markdown

| 时间 | 疼痛部位 | VAS评分 | 诱因 | 用药情况 | 副作用 |

|------|----------|---------|------|----------|--------|

| 08:00| 腰部 | 6 | 久坐 | 塞来昔布200mg | 轻微恶心 |

```

**剂量计算器**：

- NSAIDs：剂量=体重(kg)×5-10mg/kg（不超过日上限）

- 曲马多：单次剂量=50mg×(年龄/50)+25mg（50岁以上者）

二十八、

1.药物相关性尿液颜色变化的机制分析

| 药物类别 | 典型药物 | 代谢途径 | 尿液颜色变化特征 | 发生概率 |

|----------------|---------------|---------------------------|------------------------------|----------|

| **抗叶酸类** | 甲氨蝶呤(MTX) | 肝脏代谢→肾脏排泄 | 深黄色至琥珀色（似浓茶） | 60-80% |

| **NSAIDs** | 布洛芬 | 肾脏原型排泄 | 浅黄色加深（与剂量相关） | 15-30% |

| **生物制剂** | 阿达木单抗 | 肝脏代谢→胆汁排泄 | 金黄色尿液（胆红素升高） | 5-10% |

| **JAK抑制剂** | 托法替布 | 肾脏排泄 | 轻微加深（个体差异明显） | <5% |

2.非药物性尿液颜色干扰因素

**饮食因素**：

- 摄入大量β-胡萝卜素（胡萝卜、南瓜、红薯）→ 橙黄色尿液

- 服用维生素B族补充剂（尤其B2）→ 黄绿色尿液

**生理性原因**：

- 脱水（尿比重>1.030）→ 尿液浓缩呈深黄色

- 晨起首次排尿（夜间代谢产物积累）→ 正常生理现象

**病理性因素**：

- 肝功能异常（胆红素代谢障碍）→ 巩膜黄染+浓茶色尿

- 泌尿系感染（大肠杆菌分解尿素）→ 淡黄色浑浊尿

- 胆道梗阻（结合胆红素反流）→ 陶土样灰白色尿

3.临床评估与处理流程

A[尿液变黄] --> B{是否伴随以下症状?}

B -->|是| C[尿痛/尿频/发热/黄疸]

B -->|否| D[过去24小时饮水量<500ml?]

D -->|是| E[增加饮水至2000ml/d]

D -->|否| F[检查近期用药记录]

F --> G[确认是否使用MTX/阿达木单抗]

G -->|是| H[检测尿常规+肝功能]

G -->|否| I[排除饮食因素后观察3天]

H --> J[根据结果调整治疗方案]

I --> K[若持续需就医评估]

```

4.具体药物应对方案

** 甲氨蝶呤（MTX）使用者**：

- **预期现象**：用药后24-48小时出现深黄色尿

- **处理措施**：

- 确保每日饮水≥2000ml（维持尿量1500ml/d）

- 可口服碳酸氢钠片（0.5g tid）碱化尿液

- 检测MTX血药浓度（治疗窗：5-25μmol/L）

** 生物制剂（如阿达木单抗）**：

- **特殊预警信号**：持续金黄色尿+皮肤巩膜黄染

- **处理措施**：

- 立即停用生物制剂

- 检测总胆红素（正常值≤1.2mg/dL）

- 考虑转换为IL-17抑制剂（司库奇尤单抗）

** NSAIDs使用者**：

- **可逆性反应**：停药后2-3天恢复

- **处理措施**：

- 改用对乙酰氨基酚（日剂量≤3000mg）

- 检测肌酐清除率（eGFR≥60ml/min/1.73m²）

5.辅助检查建议（出现以下情况时）

| 检查项目 | 推荐指数 | 临床意义 |

|-------------------|----------|------------------------------|

| 尿常规 | ★★★★☆ | 排除尿路感染/结晶尿 |

| 肝功能（ALT/AST） | ★★★★☆ | 评估药物性肝损伤 |

| 尿量监测 | ★★★☆☆ | 判断脱水状态 |

| 胆红素分型检测 | ★★☆☆☆ | 区分肝细胞性/梗阻性黄疸 |

6.患者自我管理技巧

**尿液颜色对照表**：

- 透明淡黄（正常）

- 深琥珀色（需关注）

- 金黄色（警惕药物/肝胆问题）

- 茶色/酱油色（紧急就医）

**用药与饮水时间表**：

- MTX服用后每小时饮用250ml温水

- 生物制剂注射前后各补充500ml液体

**饮食调整方案**：

- 限制高β-胡萝卜素食物摄入（每日≤300mg）

- 增加柑橘类水果（维生素C促进药物代谢）

7.建议患者使用手机拍照记录尿液颜色变化，并在复诊时携带用药清单及检查结果。记住：90%的药物性尿液颜色改变是可逆的，及时沟通医生可获得有效管理。

二十九、

1.生物制剂本质与作用机制

**定义**：生物制剂是通过基因工程技术制备的蛋白质药物，靶向调控特定炎症通路的关键分子。

**与传统药物对比**：

| 药物类型 | 作用靶点 | 生产方式 | 给药途径 |

|----------------|-------------------------|-------------------|----------------|

| **生物制剂** | TNF-α、IL-17、IL-23等 | 细胞培养发酵 | 注射（皮下/静脉） |

| **传统DMARDs** | 磷酸二酯酶、叶酸代谢酶 | 化学合成 | 口服 |

**核心作用原理**：

- **精准打击炎症**：例如TNF-α抑制剂（如阿达木单抗）可中和血液中多余的促炎因子，降低滑膜炎症反应。

- **阻断病程进展**：IL-17抑制剂（如司库奇尤单抗）可抑制新骨形成，延缓脊柱竹节样变。

2.主要生物制剂类型与适应症

| 药物类别 | 代表药物 | 靶向通路 | 主要适用人群 | 疗效数据（52周） |

|-------------------|-------------------|-------------------|-----------------------------|-----------------------------|

| **TNF-α抑制剂** | 阿达木单抗 | TNF-α受体 | 对MTX反应不佳者 | BASDAI评分改善≥50%（82%患者） |

| | 英夫利昔单抗 | TNF-α | 严重外周关节炎 | 脊柱活动度恢复率67% |

| **IL-17抑制剂** | 司库奇尤单抗 | IL-17/IL-23受体 | 中轴型脊柱炎为主 | 疼痛缓解率91% |

| **IL-23抑制剂** | 乌司奴单抗 | IL-23/p19 | 对IL-17抑制剂耐药者 | 炎症标志物下降幅度达78% |

| **JAK抑制剂** | 托法替布 | JAK-STAT通路 | 激素依赖型患者 | 50%患者可减少激素用量 |

3.生物制剂的优势与临床价值

**快速起效**：

- TNF-α抑制剂通常在2-4周内显著改善症状（ESR/CRP下降50%以上）。

**靶向性治疗**：

- 对肠道菌群影响较小（与传统MTX相比，肠道感染风险降低40%）。

**预防关节破坏**：

- 早期使用可降低脊柱融合风险76%（10年随访数据）。

**个性化选择**：

- 根据HLA-B27状态选择（HLA-B27阳性者对IL-17抑制剂反应更佳）。

4.安全性管理与副作用应对

| 常见副作用 | 发生率 | 处理方案 | 监测指标 |

|---------------------|--------|---------------------------------|---------------------------|

| 注射部位反应 | 30-50% | 热敷、局部激素注射 | 每周评估红肿程度 |

| 感染风险 | 15-20% | 预防性使用复方新诺明（每周1次） | 每月筛查结核/肝炎病毒 |

| 诱发抗体 | 8-12% | 联合免疫抑制剂（如MTX） | 每3个月检测抗药抗体水平 |

| 心血管事件 | <1% | 评估基线心血管风险 | 定期监测血压/血脂 |

**特殊人群注意事项**：

- **乙肝携带者**：需联合抗病毒药物（如恩替卡韦）。

- **妊娠期患者**：IL-17抑制剂（如司库奇尤单抗）需停用3个月以上。

5.使用建议与个体化策略

**疗效评估周期**：

- 第0/4/12/24周评估BASDAI、ASDAS评分。

- 影像学监测：每6个月骶髂关节MRI（脂肪抑制T2WI）。

**停药时机**：

- 达标标准：CRP<1mg/L、ASDAS-CRP<2.1。

- 逐步减量（如每3个月减少20%剂量）。

**经济性考量**：

- 预计年均费用：3万-8万元（国产仿制药可降低60%成本）。

6.建议尽快到风湿免疫科进行生物制剂筛查（包括结核菌素试验、肝炎标志物），并参与「生物制剂疗效预测评分」（如BASDAI-EFFICACY评分）。记住：科学用药可使AS患者达到与健康人相似的生活质量和预期寿命。

三十、

1.生物制剂副作用的全景透视

**常见副作用及应对**

| 副作用类型 | 发生率（临床研究数据） | 典型表现 | 处理方案 | 监测频率 |

|--------------------|------------------------|-------------------------------|-----------------------------------|------------------|

| **注射部位反应** | 30%-50% | 红肿、瘙痒、硬结 | 热敷、局部糖皮质激素注射 | 每次注射后24小时 |

| **感染风险** | 15%-20% | 呼吸道感染、肠道感染 | 预防性使用复方新诺明（每周1次） | 每月筛查结核/肝炎 |

| **抗体诱导** | 8%-12% | 输液反应、疗效下降 | 联合MTX（可降低抗体生成率40%） | 每3个月检测抗体 |

| **胃肠道不适** | 5%-10% | 恶心、腹泻 | 餐后服用、调整给药间隔 | 每周评估症状 |

| **心血管风险** | <1% | 高血压、血栓形成 | 基线评估心血管风险，定期监测 | 每6个月心电图 |

**罕见但需警惕的副作用**

- **过敏反应**（发生率<0.1%）：表现为皮疹、喉头水肿，需立即停药并使用肾上腺素。

- **肝肾功能异常**（发生率<0.5%）：定期检测ALT、AST、肌酐（用药后第4周及每3个月）。

- **神经系统症状**（如头痛、眩晕）：多为一过性，严重者需停药。

2.副作用管理的核心原则

**个体化风险评估**：

- **基线筛查**：结核菌素试验（PPD）、肝炎病毒标志物、乙肝表面抗原。

- **基因检测**：IL-23R基因型（预测IL-17抑制剂疗效及抗体风险）。

**分级管理策略**：

| 副作用严重程度 | 处理措施 | 是否需要停药 |

|----------------|---------------------------------------|----------------------------|

| 轻度 | 对症治疗（如止痒药、退热药） | 继续用药 |

| 中度 | 暂停给药→症状缓解后重启（减量50%） | 是 |

| 重度 | 立即停药+急诊处理（如抗过敏、抗感染） | 是 |

**预防性干预措施**：

- **感染预防**：

- 乙型肝炎携带者：联合恩替卡韦（替诺福韦酯耐药率<1%）。

- 糖尿病患者：加强血糖监测（感染风险升高2倍）。

- **抗体管理**：

- 使用生物制剂前筛查抗TNF-α抗体（基线阳性者需谨慎）。

3.特殊人群用药注意事项

** 肝功能异常者**：

- 选择肝毒性低的药物（如IL-17抑制剂司库奇尤单抗）。

- 避免MTX联合生物制剂（肝毒性叠加风险↑3倍）。

** 肾功能不全者**：

- 避免使用NSAIDs（肾毒性风险↑5倍）。

- 生物制剂中阿达木单抗需调整剂量（eGFR<30ml/min/1.73m²时减量50%）。

** 妊娠期与哺乳期**：

- **FDA妊娠分级**：

- B级（较安全）：塞妥珠单抗、依那西普。

- C级（慎用）：英夫利昔单抗、阿达木单抗。

- 哺乳期建议：

- TNF-α抑制剂需停药3个月以上。

- IL-17抑制剂（司库奇尤单抗）乳汁浓度极低，可哺乳。

** 免疫缺陷患者**：

- 禁用JAK抑制剂（托法替布）。

- 生物制剂需联合免疫抑制剂（如MTX）。

4.降低副作用风险的实用技巧

**注射技术优化**：

- 选择腹部脂肪较厚部位（脐周2cm外）。

- 注射后按压5分钟+冷敷10分钟（减少红肿）。

**用药时间管理**：

- 固定注射时间（如每周三上午），减少应激反应。

- 避免在发热或感染期间用药。

**生活方式干预**：

- 戒烟（吸烟者感染风险↑2倍）。

- 保持BMI≤24kg/m²（降低心血管并发症风险）。

三十一、

1.生物制剂与成瘾的本质区别

** 作用机制决定成瘾可能性**

生物制剂通过靶向调控免疫细胞表面受体（如TNF-α、IL-17）或细胞内信号通路（如JAK-STAT），抑制过度炎症反应。

** vs **

传统成瘾药物（如阿片类）通过作用于中枢神经系统的μ受体，产生欣快感和依赖性。

** 药物代谢途径无成瘾相关靶点**

- **生物制剂**：在肝脏代谢后经肾脏或胆汁排泄，不穿透血脑屏障。

- **成瘾药物**：如可卡因、吗啡等直接作用于大脑奖赏回路（多巴胺系统）。

2."依赖性"的另一种可能：停药反应

部分患者可能因病情控制稳定后突然停药导致炎症反弹，但这属于**药理学依赖**，与成瘾无关。

**科学管理策略**：

**逐步减量**：

- 每3个月减少20%剂量（如阿达木单抗从40mg qw降至20mg qw）。

- 配合MTX（7.5mg/w）过渡，可降低复发风险45%。

**生物制剂转换**：

- 对IL-17抑制剂耐药者，转换为IL-23抑制剂（乌司奴单抗）可维持疗效。

**症状监控**：

- 停药期间每周监测CRP、ESR，出现晨僵加重需立即重启治疗。

3.患者常见误解与科学澄清

| 误解类型 | 科学解释 | 管理建议 |

|-------------------------|-------------------------------------------|----------------------------------|

| "用了生物制剂就停不下" | 炎症控制后需规范停药，非心理依赖 | 参与「停药过渡计划」，医生监督下减量 |

| "生物制剂会上瘾" | 无中枢神经作用，但需警惕治疗依从性 | 使用用药提醒APP，定期复诊 |

| "停药后会更痛苦" | 突然停药可能导致疾病活动度反弹（OR=2.3） | 预先制定应急预案（如备用生物制剂） |

4.长期用药的安全性证据

**1. 5年临床数据**：

- **成瘾相关事件**：0例（基于全球安全性数据库VigiAccess收录的12万AS患者）。

- **感染并发症**：年均12-15%（可控且可预防）。

** 戒断综合征风险**：

- 仅见于JAK抑制剂（托法替布），表现为关节痛、发热（发生率<1%），需立即重启药物。

5.患者自我管理建议

**建立用药依从性档案**：

- 记录生物制剂使用时间、剂量、停药计划。

**物理隔离药品**：

- 将生物制剂存放于儿童无法触及处，避免误服。

**认知行为干预**：

- 参与正念减压训练（MBSR），降低对药物的焦虑感。

6.建议患者通过医院「生物制剂停药门诊」制定个体化方案，并使用可穿戴设备监测关节活动度（如AxIT关节传感器），确保病情稳定后再逐步停药。记住：规范的生物制剂管理可使AS患者5年生存质量评分（EQ-5D）提升37%。

三十二、

1.感染与生物制剂使用的核心原则

**基本原则**：

生物制剂通过抑制免疫系统发挥作用，因此**活动性感染患者禁用**，但**潜伏感染或稳定感染者可谨慎使用**（需个体化评估）。

**国际指南共识**：

- EULAR 2022：AS患者使用生物制剂前需筛查HBV、HCV、TB等

- ACRA 2021：活动性感染未控制者禁止使用IL-17/IL-23抑制剂

2.具体感染类型的应对策略

| 感染类型 | 处理流程 | 生物制剂重启时机 | 监测指标 |

|----------------|--------------------------------------------------------------------------|--------------------------------------|-----------------------------------|

| **活动性乙肝** | 1. 立即停用生物制剂<br>2. 联合恩替卡韦/替诺福韦酯（耐药率<1%）<br>3. HBV DNA转阴后评估 | HBV DNA<2000 IU/mL且肝功能正常 | 每月检测HBV DNA、ALT/AST |

| **活动性结核** | 1. 先完成2HRZE/4HR抗结核治疗<br>2. 治疗结束后再启动生物制剂 | 结核病灶吸收、痰菌阴性 | 每周痰涂片+GeneXpert MTB/RIF |

| **带状疱疹** | 1. 首选阿昔洛韦（750mg q8h）治疗<br>2. 恢复后重启生物制剂 | 皮疹消退、无新发水疱 | 每日监测神经痛程度 |

| **细菌感染** | 1. 根据药敏结果选择抗生素（如MRSA用万古霉素）<br>2. 感染控制后重启生物制剂 | 体温正常、CRP<1mg/L | 每3天复查血常规+炎症标志物 |

| **真菌感染** | 1. 伊曲康唑/伏立康唑治疗至少2周<br>2. 再评估生物制剂使用 | 病原学清除、临床症状消失 | 每周监测G试验/GM试验 |

| **HIV感染** | 1. CD4+T细胞>200cells/mm³时可用IL-17抑制剂<br>2. 密切监测机会性感染 | 免疫功能稳定（CD4+T细胞>350cells/mm³） | 每月检测HIV RNA载量、CD4+T细胞计数 |

3.乙肝携带者的特殊管理方案

**治疗方案**：

- **恩替卡韦**：1mg qd（肾毒性低，孕妇可用）

- **替诺福韦酯**：200mg qd（需监测肾功能，肌酐清除率<30ml/min时减量）

**生物制剂选择**：

- **优先选择IL-17抑制剂**（司库奇尤单抗）：对HBV无直接影响，感染风险<0.5%

- **慎用TNF-α抑制剂**：可能诱发HBV再激活（发生率3-5%）

**监测要点**：

- 治疗前HBV DNA定量（IU/mL）

- 每4周检测肝功能、HBV DNA

- 出现黄疸立即停用生物制剂+紧急抗病毒治疗

4.结核潜伏感染的处置流程

**筛查步骤**：

1. **PPD试验**：≥10mm为强阳性

2. **γ-干扰素释放试验（IGRA）**：阳性提示潜伏TB

3. **胸片检查**：排除活动性病变

**处理方案**：

- **强阳性/IGRA阳性**：

- 异烟肼（5mg/kg qd）+ 利福平（10mg/kg qd）联合化疗3个月

- 生物制剂启动时机：完成化疗后6个月，且痰菌阴性

- **弱阳性/胸片正常**：

- 密切观察，生物制剂使用期间每3个月复查CT

5.合并感染的生物制剂调整策略

** 轻度感染（如上呼吸道感染）**：

- 暂停生物制剂1-2周，抗生素治疗后重启

- 优先选择IL-17抑制剂（司库奇尤单抗）：对病毒/细菌干扰较小

** 严重感染（如败血症）**：

- 立即停用所有生物制剂

- 使用广谱抗生素（如美罗培南）+ 免疫球蛋白支持治疗

- 恢复后转换为JAK抑制剂（托法替布5mg qd）过渡

** 病毒感染（如COVID-19）**：

- **无症状/轻症**：继续生物制剂（无证据显示增加重症风险）

- **重症/危重症**：暂停生物制剂，使用IL-6抑制剂（托珠单抗）治疗细胞因子风暴

6.预防性感染防控体系

** 生物制剂启用前筛查**：

- HBV五项、HCV抗体、TB-PPD/IGRA、HIV抗原

- 既往疫苗接种记录（如流感、肺炎球菌）

** 用药期间预防措施**：

- **复方新诺明**：每周1次（预防卡氏肺孢子菌肺炎）

- **阿昔洛韦**：500mg qd（预防带状疱疹，尤其HIV+患者）

- **流感疫苗**：每年接种灭活疫苗（ASDAS-CRP≥2.1者需提前2周用药）

** 环境防护**：

- 避免去人群密集场所（疫情期间佩戴N95口罩）

- 定期紫外线消毒居住环境（每日30分钟）

三十三、

1.生物制剂需联用的药物类型及原因

| 药物类别 | 联用目的 | 典型方案 | 临床数据支持 |

|----------------|-----------------------------------|-----------------------------------|----------------------------------|

| **传统DMARDs** | 延长疗效、减少生物制剂用量 | MTX（7.5mg/w）+ 生物制剂 | 联合用药者复发率降低37%（NEJM 2021） |

| **NSAIDs** | 快速缓解疼痛与晨僵 | 塞来昔布200mg qd + 生物制剂 | VAS评分改善率提升52%（PLOS ONE 2022） |

| **糖皮质激素** | 急性期炎症控制 | 泼尼松龙0.5mg/kg/d（2周后减量） | 关节肿胀消退速度加快40%（Ann Rheum Dis） |

| **JAK抑制剂** | 对生物制剂耐药者的挽救治疗 | 托法替布5mg qd + 生物制剂 | 50%患者可减少生物制剂剂量 |

2.联合用药的临床实践指南

** 初始治疗阶段**：

- **双药联用**：生物制剂（如阿达木单抗40mg qw）+ MTX（15mg qw）

- **目标**：24周内达到ASDAS-CRP<2.1分

** 疗效维持阶段**：

- **单药维持**：生物制剂（剂量减半）

- **停药过渡**：每3个月减少20%剂量，同时加用MTX（7.5mg/w）

** 急性加重期**：

- **三联疗法**：生物制剂 + MTX + 糖皮质激素（泼尼松龙40mg qd）

- **疗程**：2-4周后逐步减量至生物制剂单药

3.特殊场景下的联合用药方案

** 合并胃肠道疾病**：

- **方案**：生物制剂 + 艾瑞昔布（50mg qd） + 奥美拉唑20mg qd

- **理由**：艾瑞昔布对COX-2选择性更高，胃肠副作用降低40%

** 合并心血管风险**：

- **方案**：生物制剂 + 萘丁美酮（250mg qd） + 阿托伐他汀10mg qd

- **理由**：萘丁美酮心血管事件风险最低，阿托伐他汀控制血脂

** 合并银屑病**：

- **方案**：乌司奴单抗150mg q2w + MTX 10mg qw

- **理由**：IL-23抑制剂对银屑病皮损改善率70%（Lancet 2020）

4.药物相互作用警示

| 药物组合 | 相互作用机制 | AS患者风险等级 | 管理建议 |

|-------------------------|-----------------------------|--------------|------------------------------|

| MTX + 生物制剂 | 增加肝毒性风险 | 中危 | 每月监测肝功能，MTX剂量≤15mg/w |

| 生物制剂 + 糖皮质激素 | 隐藏性感染风险升高 | 高危 | 每周筛查CRP/ESR，避免长期联用 |

| NSAIDs + 抗凝药 | 增加胃肠道出血风险 | 极高危 | 搭配PPI（奥美拉唑20mg qd） |

| JAK抑制剂 + 生物制剂 | 可能导致免疫过度抑制 | 禁忌 | 严格避免联用 |

5.患者自我管理建议

**用药清单管理**：

- 使用电子药历记录所有用药（包括生物制剂、MTX、NSAIDs）

- 设置手机提醒（如每周MTX服用时间）

**不良反应监测表**：

| 症状 | 可能药物 | 处理措施 |

|-----------------|---------------|---------------------------|

| 恶心/呕吐 | MTX/NSAIDs | 加用维生素B6（10mg qd） |

| 皮疹 | 生物制剂 | 立即停药并联系医生 |

| 高血压 | 糖皮质激素 | 每日监测血压，调整剂量 |

**联合治疗依从性技巧**：

- 将MTX片剂分装至每日剂量小瓶

- 使用注射器预装生物制剂（如阿达木单抗预充式笔）

6.建议患者参与「生物制剂联合用药管理计划」，通过医院药房的药物相互作用检测系统（如TruvaxiScan）评估用药方案安全性。记住：科学联用可使AS患者5年关节破坏风险降低68%（Ann Rheum Dis 2023）。

三十四、

1.生物制剂是否需要终身使用？

**核心结论**：

**否**，生物制剂并非必须终身使用。约**70%-80%的AS患者最终可实现药物减停**，关键在于：

**早期规范治疗**（确诊后2年内启动生物制剂）

**严格疗效评估**（每3个月监测ASDAS-CRP评分）

**科学减量策略**（每3个月减少20%剂量）

2.停药决策的客观标准

| 指标类型 | 达标标准 | 监测频率 |

|-----------------|-----------------------------------|--------------|

| 炎症控制 | CRP<1mg/L，ESR<20mm/h | 每4周 |

| 影像学稳定 | 骶髂关节MRI无新发渗出 | 每6个月 |

| 功能恢复 | Schober试验>5cm，脊柱活动度恢复 | 每3个月 |

| 症状缓解 | 夜间痛醒≤1次/月，VAS评分≤3分 | 每月自评 |

3.停药流程的科学管理

A[满足停药标准] --> B{是否联合MTX?}

B -->|是| C[逐步减量生物制剂+维持MTX]

B -->|否| D[直接减量生物制剂]

C --> E[每3个月减少20%剂量]

D --> E

E --> F[剂量降至原剂量的25%时评估]

F -->|达标| G[停药并转为NSAIDs/MTX维持]

F -->|未达标| H[重启生物制剂]

4.停药后复发防控策略

**过渡期管理**：

- 减量期间联合MTX（7.5mg/w）可降低复发风险45%

- 备用生物制剂（如阿达木单抗预充剂型）以备急需

**复发预警信号**：

- CRP上升>0.5mg/L

- VAS评分≥4分（疼痛加剧）

- 新发关节肿胀

**应急处理方案**：

- **轻度复发**：重启生物制剂原剂量

- **重度复发**：联合糖皮质激素（泼尼松龙0.5mg/kg/d）冲击治疗

5.特殊人群停药注意事项

** 高危复发人群**：

- **合并髋关节强直**：需终身使用生物制剂（关节破坏不可逆）

- **HLA-B27阳性**：复发率较阴性者高30%，建议延长疗程

** 合并其他疾病者**：

- **银屑病患者**：优先选择IL-23抑制剂（乌司奴单抗），可长期维持

- **心血管高风险**：慎用TNF-α抑制剂，可转换为IL-17抑制剂

6.建议患者参与「生物制剂停药管理计划」，通过可穿戴设备（如Dexcom G6连续血糖仪）监测炎症指标，确保安全减停。记住：现代医学已使AS患者停药率大幅提升，关键在于早期干预和动态调整。

三十五、

1.激素在AS治疗中的定位与作用机制

** 核心作用**：

激素通过抑制NF-κB通路和MAPK信号传导，快速降低炎症因子（如TNF-α、IL-6）的释放，从而缓解急性炎症反应。

** 适应症**：

- **重度活动期**：BASDAI评分>7分或ASDAS-CRP≥2.1分

- **局部炎症**：单关节肿胀或附着点炎

- **急性并发症**：虹膜炎、葡萄膜炎

** 禁忌症**：

- 活动性感染（如结核、HIV）

- 严重骨质疏松（骨密度T值<-2.5）

- 糖尿病未控制（HbA1c>7%）

2.常用激素类药物及使用方案

| 药物类型 | 剂型 | 推荐剂量 | 作用特点 |

|------------------|--------------|--------------------------------|------------------------------|

| **系统性糖皮质激素** | 口服 | 泼尼松龙0.5mg/kg/d（最大剂量40mg/d） | 快速控制全身炎症，短期使用（≤4周） |

| **关节腔注射激素** | 局部注射 | 甲强龙40mg/mL（单次注射） | 针对单个肿胀关节，疗效持续4-6周 |

| **局部软膏激素** | 外用 | 强效类固醇（如丙酸氯倍他索） | 缓解皮肤或黏膜炎症 |

**临床数据支持**：

- 系统性激素可使AS患者疼痛缓解率提升60%（VAS评分下降≥3分，48小时内）

- 关节腔注射可使肿胀关节恢复率提高85%（1周内）

3.激素联用策略与注意事项

** 联合用药方案**：

- **生物制剂+激素**：

- 急性期：阿达木单抗40mg qw + 泼尼松龙0.5mg/kg/d（2周后减量）

- 优势：快速控制炎症，降低生物制剂耐药风险

- **MTX+激素**：

- MTX 15mg/w + 泼尼松龙10mg qd（晨僵严重者）

- 注意：MTX需空腹服用，激素餐后服用以减少胃肠道刺激

** 剂量调整原则**：

- **阶梯减量法**：每5-7天减少50%剂量，直至停药

- **监测指标**：

- 每日晨起血压、血糖

- 每周血常规、电解质（尤其钾、钠）

** 常见副作用及应对**：

| 副作用类型 | 高危人群 | 处理措施 |

|--------------------|---------------------------|------------------------------|

| 骨质疏松 | 长期使用者（>3个月） | 联合双膦酸盐（每周5mg阿仑膦酸钠） |

| 糖尿病 | BMI>25kg/m²或家族史 | 监测空腹血糖，必要时加用二甲双胍 |

| 消化道溃疡 | 联合NSAIDs使用者 | 加用PPI（奥美拉唑20mg qd） |

4.特殊人群激素应用规范

** 青少年患者**：

- **优先选择局部治疗**：关节腔注射（减少全身副作用）

- **剂量调整**：泼尼松龙剂量按体重计算（0.3-0.5mg/kg/d）

**妊娠期患者**：

- **FDA妊娠分级**：

- B级（较安全）：泼尼松龙（≤20mg/d）

- C级（慎用）：地塞米松（需权衡利弊）

- **停药时机**：妊娠32周后逐步减量至维持剂量

** 老年患者**：

- **起始剂量减半**：泼尼松龙0.25mg/kg/d

- **加强监测**：每周评估肌酐清除率、跌倒风险

5.疗效评估与停药时机

** 疗效判断标准**：

- **临床缓解**：BASDAI评分<1.5，晨僵<15分钟

- **炎症控制**：CRP<1mg/L，ESR<20mm/h

** 停药指征**：

- 症状完全缓解维持≥4周

- 影像学无新发骨侵蚀

- 无并发症（如虹膜炎）

** 复发处理**：

- **轻度复发**：重启泼尼松龙10mg qd，2周后逐步减量

- **重度复发**：联合生物制剂（如托珠单抗30mg qd）

6.建议患者通过医院「激素治疗监测系统」进行动态管理，包括血压、血糖、骨密度等指标的实时跟踪。记住：正确使用激素可使AS患者急性期症状缓解速度提升50%，但需严格遵循「短期、小剂量、个体化」原则。

三十六、

1.术前药物调整核心原则

** 免疫抑制剂管理**：

- **生物制剂**：术前2-4周停用（如阿达木单抗、英夫利昔单抗），降低术后感染风险（尤其是关节置换术）。

- **MTX**：术前2周减量至7.5mg/w，术后4周恢复原剂量（减少骨髓抑制风险）。

** 糖皮质激素**：

- 术前1周将泼尼松龙剂量减半（如从40mg/d降至20mg/d），术后24-48小时逐步恢复。

- 局部激素（如关节腔注射）：术前3天停用，避免局部组织脆弱。

** NSAIDs**：

- 术前48小时停用非选择性NSAIDs（如布洛芬），但可继续选择性COX-2抑制剂（如塞来昔布200mg qd）至术前6小时。

2.手术类型特异性调整方案

| 手术类型 | 生物制剂停药时间 | MTX调整策略 | NSAIDs管理 |

|----------------|------------------|------------------------------|------------------------------|

| **关节置换术** | 提前4周 | 术前2周减量至7.5mg/w | 术前48小时停用NSAIDs |

| **脊柱手术** | 提前3周 | 术前1周停用MTX | 术前三天改用对乙酰氨基酚 |

| **小关节手术** | 提前2周 | 维持MTX 7.5mg/w | 术前24小时停用NSAIDs |

3.术后药物重启策略

** 生物制剂**：

- 术后4-6周评估炎症控制（CRP<1mg/L），逐步重启生物制剂（剂量恢复至术前水平）。

- **优先级**：IL-17抑制剂（司库奇尤单抗）因对肠道菌群影响小，术后感染风险较低。

** MTX**：

- 术后4周恢复7.5mg/w剂量，联合维生素B12（1000μg qd）预防神经毒性。

** NSAIDs**：

- 术后24小时开始使用选择性COX-2抑制剂（塞来昔布200mg qd），持续4-6周。

4.围手术期感染防控

** 预防性抗生素**：

- **关节置换术**：术前2小时静滴头孢唑林钠2g，术后48小时持续使用。

- **脊柱手术**：术前3天口服多西环素100mg bid，术后7天继续。

** 术后监测**：

- 每日监测CRP/ESR，术后3天行血培养+药敏试验。

- 发热>38.5℃或CRP>50mg/L需立即启动广谱抗生素（如美罗培南）。

5.特殊人群管理

** 糖尿病患者**：

- 术前HbA1c需控制在<7%，术后继续胰岛素强化治疗（血糖目标7.8-10mmol/L）。

** 老年患者**：

- 术前评估肌酐清除率（eGFR），MTX剂量调整为5mg/w（原剂量的2/3）。

** 合并心血管疾病**：

- 术前停用NSAIDs（增加心血管事件风险），改用对乙酰氨基酚（日剂量≤3000mg）。

6.建议患者通过医院「围手术期药物管理系统」进行用药记录和监测，确保手术安全与疾病控制双达标。记住：科学管理围手术期用药可使AS患者术后感染率降低60%，关节功能恢复速度提升40%。

三十七、

1.药物相互作用风险评估

| 强直药物类别 | 典型药物 | 感冒药成分 | 相互作用机制 | 风险等级 | 管理建议 |

|------------------|---------------|---------------------|-----------------------------|----------|------------------------------|

| **NSAIDs** | 塞来昔布 | 对乙酰氨基酚 | 肝毒性叠加（风险↑3倍） | 高危 | 优先选择单一退热药，监测肝功能 |

| | 布洛芬 | 阿司匹林 | 胃肠道出血风险↑5倍 | 极高危 | 避免联用，改用对乙酰氨基酚 |

| **DMARDs** | 甲氨蝶呤(MTX) | 利巴韦林（抗病毒药） | 骨髓抑制风险↑7倍 | 禁忌 | 感冒期间暂停MTX，改用JAK抑制剂 |

| | 磺胺嘧啶银 | 复方新诺明 | 磺胺类药物过敏风险↑30% | 中危 | 交叉过敏者禁用，监测皮疹 |

| **生物制剂** | 阿达木单抗 | 解热镇痛药 | 无显著相互作用 | 低危 | 可常规联用，注意感染防控 |

| | 托法替布 | 抗组胺药（如西替利嗪） | 过敏反应风险↑2倍 | 中危 | 用药前筛查过敏史 |

2.合并感冒的阶梯式用药方案

** 轻症感冒（体温<38.5℃）**：

- **首选方案**：对乙酰氨基酚（3000mg/d，分4次）

- **强直药物调整**：无需停用NSAIDs/DMARDs，但需监测CRP/ESR

** 中重症感冒（体温≥38.5℃）**：

- **退热方案**：

- 布洛芬（400mg q6h） + 对乙酰氨基酚（500mg q4h）（交替使用）

- **强直药物调整**：

- 暂停NSAIDs（减少胃肠道出血风险）

- MTX减量至5mg/w（维持免疫抑制平衡）

** 特殊并发症处理**：

- **肺炎风险**：

- 联合使用阿奇霉素（500mg qd）+ 多西环素（100mg bid）

- **胃肠道症状**：

- 恶心呕吐：加用昂丹司琼（4-8mg qd）

- 便秘：启用缓泻剂（乳果糖30ml qd）

3.重要警示与禁忌

** 绝对禁忌联用**：

- **MTX + 利巴韦林**：增加淋巴瘤风险

- **NSAIDs + 抗凝药**：出血风险↑10倍

- **生物制剂 + 活疫苗**：诱发严重感染

** 需医生评估的情况**：

- 合并慢性肾病（eGFR<30ml/min）：需调整退热药剂量

- 合并心血管疾病：避免布洛芬（心血管事件风险↑3倍）

- 孕妇/哺乳期：优先选择对乙酰氨基酚（FDA妊娠分级B级）

4.患者自我管理技巧

**用药记录工具**：

- 使用「感冒-强直药物相互作用表」（附后）实时核对

**症状监测清单**：

- 每日记录体温、咽痛、咳嗽程度、关节肿痛变化

**紧急情况识别**：

- 出现呼吸困难、胸痛、意识模糊需立即就医

- 连续3天高热不退（>39℃）需启动抗病毒治疗（如奥司他韦）

5.建议患者下载「AS用药助手」APP，输入当前用药清单可自动检测潜在相互作用。记住：及时沟通医生比自行调整用药更安全！

三十八、

1.强直性脊柱炎对生育的影响

** 疾病本身**

- **直接影响**：强直性脊柱炎不损害生殖系统，但严重髋关节强直（>90%患者）可能限制活动，间接影响生育信心。

- **炎症因子影响**：高水平的TNF-α、IL-6可能干扰排卵功能（研究显示CRP>5mg/L者受孕周期延长2.3倍）。

** 并发症风险**

- **虹膜炎**：急性发作期可能诱发青光眼，需孕前控制炎症。

- **骨质疏松**：长期使用激素者骨密度下降，增加妊娠期骨折风险。

2.药物对妊娠的潜在风险

| 药物类别 | 典型药物 | 妊娠风险等级（FDA分级） | 关键注意事项 |

|----------------|---------------|-------------------------|-------------------------------------|

| **MTX** | 甲氨蝶呤 | X级（禁用） | 妊娠前3个月停用，改用JAK抑制剂或生物制剂过渡 |

| **生物制剂** | 阿达木单抗 | B级（较安全） | 妊娠期需继续使用（降低疾病活动度），但增加感染风险 |

| **NSAIDs** | 塞来昔布 | C级（慎用） | 妊娠中晚期禁用（影响胎儿心血管发育），可短期使用对乙酰氨基酚 |

| **糖皮质激素** | 泼尼松龙 | B级（较安全） | 妊娠期需监测血糖（剂量>20mg/d增加早产风险） |

3.备孕前科学准备

** 疾病控制目标**

- **炎症指标**：CRP<1mg/L，ESR<20mm/h（降低流产风险40%）

- **影像学稳定**：骶髂关节MRI无新发骨侵蚀（妊娠期复发率降低60%）

** 药物调整方案**

- **MTX使用者**：

- 妊娠前3个月停用，改用托法替布5mg qd过渡（JAK抑制剂妊娠分级B级）。

- **生物制剂使用者**：

- 继续使用（如阿达木单抗40mg qw），但需联合复方新诺明预防感染。

- **NSAIDs使用者**：

- 孕早期改用对乙酰氨基酚（日剂量≤3000mg），避免布洛芬（增加胎儿心脏缺陷风险）。

** 孕前检查清单**

- **生殖系统评估**：妇科超声（卵巢储备功能）、精子质量分析（男性患者）

- **感染筛查**：TORCH、HIV、乙肝、梅毒

- **骨密度检测**：Z值<-1.0需补充钙剂（1000mg/d）+维生素D（800IU/d）

4.孕期管理策略

**妊娠期药物调整**

- **生物制剂**：

- 妊娠中期可继续使用（如司库奇尤单抗），但需监测抗药抗体（发生率达8-12%）。

- **MTX**：

- 妊娠期禁用，紧急情况可短期使用甲氨蝶呤（≤0.4mg/kg/d），但需终止妊娠。

- **糖皮质激素**：

- 泼尼松龙剂量>20mg/d需每4小时监测血糖，必要时加用胰岛素。

** 病情监测指标**

- **每周评估**：VAS疼痛评分、晨僵时间、CRP/ESR

- **影像学监测**：每6个月骶髂关节超声（评估炎症活动度）

** 并发症预防**

- **早产风险**：

- 已知宫颈短缩（<2.5cm）者，孕14-16周行宫颈环扎术。

- **胎儿发育监测**：

- 妊娠20-24周行详细超声（排查关节畸形），胎心监护每周1次。

5.产后管理要点

** 疾病复发防控**

- **激素撤药风险**：产后1周内泼尼松龙剂量需逐步减量（避免病情反弹）。

- **生物制剂重启**：产后4-6周恢复生物制剂（如阿达木单抗40mg qw），联合MTX（7.5mg/w）预防复发。

** 母乳喂养建议**

- **生物制剂**：阿达木单抗、司库奇尤单抗乳汁浓度极低，可哺乳。

- **MTX**：哺乳期禁用（进入乳汁量<0.1mg/dL），需改用帕米膦酸盐。

- **糖皮质激素**：泼尼松龙剂量>20mg/d需弃奶（乳汁浓度达血浆浓度的5-10%）。

6.建议患者通过「AS妊娠管理平台」进行全程跟踪，包括用药记录、炎症监测、产检预约等功能。记住：规范管理可使AS患者妊娠成功率提升至85%，新生儿畸形率低于普通人群（1.2% vs 2.1%）。

三十九、

1.备孕前药物调整原则

**核心目标**：

在保证疾病控制的前提下，最大限度降低妊娠风险，需在孕前3-6个月启动药物过渡方案。

2.药物类别调整策略

| 药物类型 | 典型药物 | 备孕前处理方案 | 妊娠期建议 | 数据支持 |

|----------------|---------------|---------------------------------------|-------------------------------------|-----------------------------------|

| **MTX** | 甲氨蝶呤 | 妊娠前3个月停用，改用托法替布5mg qd过渡 | 禁用（妊娠期X级风险） | MTX可致胎儿神经管缺陷（风险↑3倍） |

| **生物制剂** | 阿达木单抗 | 继续使用（妊娠期B级安全） | 妊娠中期维持，每3个月监测抗药抗体 | 生物制剂妊娠期使用率82%（NEJM 2020） |

| **NSAIDs** | 塞来昔布 | 孕早期改用对乙酰氨基酚（≤3000mg/d） | 妊娠中晚期禁用（影响胎儿心血管） | 布洛芬致畸风险↑5倍（FDA警告） |

| **糖皮质激素** | 泼尼松龙 | 剂量减半（≤20mg/d），改用局部激素 | 妊娠期监测血糖（剂量>20mg/d增加早产风险） | 泼尼松龙乳汁浓度<0.1%（可哺乳） |

3.备孕前关键准备

** 疾病控制目标**：

- **炎症达标**：CRP<1mg/L，ESR<20mm/h（降低流产风险40%）

- **影像稳定**：骶髂关节MRI无新发骨侵蚀（妊娠期复发率↓60%）

** 药物调整方案**：

- **MTX使用者**：

- 妊娠前3个月停用MTX，改用托法替布5mg qd + 阿达木单抗40mg qw

- 补充叶酸5mg/d（持续至孕12周）

- **生物制剂使用者**：

- 继续使用生物制剂，联合复方新诺明（每周1次）预防感染

- **NSAIDs使用者**：

- 孕早期改用对乙酰氨基酚（发热时3000mg q6h），避免布洛芬

** 必备检查清单**：

- **生殖系统评估**：妇科超声（卵巢储备功能）、精子质量分析（男性）

- **感染筛查**：TORCH、HIV、乙肝、梅毒

- **骨密度检测**：Z值<-1.0需补充钙剂（1000mg/d）+维生素D（800IU/d）

4.孕期药物管理

** 妊娠期药物调整**：

- **生物制剂**：

- 妊娠中期继续使用（如司库奇尤单抗30mg qw），每3个月检测抗药抗体

- 感染风险增加20%，需加强监测（体温每日3次，CRP每周1次）

- **糖皮质激素**：

- 泼尼松龙剂量>20mg/d需胰岛素控制血糖（目标空腹7.0-9.0mmol/L）

- 产后1周内逐步减量至7.5mg/d

- **NSAIDs**：

- 孕晚期禁用，可短期使用对乙酰氨基酚（≤3000mg/d）

** 病情监测指标**：

- **每周评估**：VAS疼痛评分、晨僵时间、CRP/ESR

- **影像学监测**：每6个月骶髂关节超声（评估炎症活动度）

** 并发症预防**：

- **早产风险**：宫颈短缩（<2.5cm）者孕14-16周行宫颈环扎术

- **胎儿发育监测**：妊娠20-24周行详细超声（排查关节畸形），胎心监护每周1次

5.产后管理要点

** 疾病复发防控**：

- **激素撤药风险**：产后1周内泼尼松龙剂量需逐步减量（避免病情反弹）

- **生物制剂重启**：产后4-6周恢复阿达木单抗40mg qw，联合MTX（7.5mg/w）预防复发

**母乳喂养建议**：

- **生物制剂**：阿达木单抗、司库奇尤单抗乳汁浓度极低，可哺乳

- **MTX**：哺乳期禁用（进入乳汁量<0.1mg/dL），需改用帕米膦酸盐

- **糖皮质激素**：泼尼松龙剂量>20mg/d需弃奶（乳汁浓度达血浆浓度的5-10%）

6.建议患者通过「AS妊娠管理平台」进行全程跟踪，包括用药记录、炎症监测、产检预约等功能。记住：规范管理可使AS患者妊娠成功率提升至85%，新生儿畸形率低于普通人群（1.2% vs 2.1%）。

四十、

1.中西药治疗强直的底层逻辑差异

**西药作用机制**：

- **生物制剂**：靶向抑制TNF-α、IL-17等炎症因子（如阿达木单抗可降低CRP 50%-70%）

- **MTX**：抑制二氢叶酸还原酶，阻断DNA合成（可减缓关节破坏速度30%-50%）

**中药作用机制**：

- **补肾强督方**（如独活寄生汤）：通过调节TGF-β/Smad通路抑制骨赘形成

- **清热利湿药**（如四妙丸）：降低IL-6水平（动物实验显示可减少40%炎症因子）

**临床数据对比**：

- **西药组**：52周BASDAI评分改善≥50%（82%患者）

- **中药组**：同期改善率仅35%（且依赖个体辨证）

2.中药/针灸的辅助治疗价值

| 治疗方式 | 典型方案 | 作用靶点 | 临床证据等级 |

|----------------|-------------------------------|-------------------------|--------------|

| **中药熏蒸** | 苍术、威灵仙、红花组方 | 改善局部血液循环 | II级（RCT） |

| **针灸疗法** | 腰俞、命门、大椎穴电针刺激 | 调节Th1/Th2平衡（IL-4↓20%） | III级（Meta分析） |

| **中药灌肠** | 大黄、黄柏、苦参煎剂保留灌肠 | 抑制肠道菌群移位（减少内毒素入血） | II级（临床观察） |

**典型案例**：

- **针灸+中药组**：32例患者12周后VAS疼痛评分下降42%（vs 西药组58%）

- **中药外敷**：髋关节肿胀缓解率65%（但复发率较高，需配合MTX）

3.中西医结合的增效策略

** 急性期方案**：

- **生物制剂（阿达木单抗40mg qw）** + **中药清热解毒方（金银花30g煎服）**

- **作用**：快速控制炎症，降低生物制剂耐药风险（动物实验显示可减少抗药抗体生成）

** 缓解期方案**：

- **MTX 7.5mg/w** + **针灸足三里（双针）+ 艾灸关元穴**

- **作用**：维持疾病控制，减少MTX胃肠道副作用（恶心发生率降低30%）

** 顽固性晨僵**：

- **JAK抑制剂（托法替布5mg qd）** + **中药活血通络方（川芎6g、红花3g冲服）**

- **作用**：协同改善晨僵时间（15分钟→45分钟，p<0.01）

4.中医替代西药的禁忌与风险

** 单纯中药的风险**：

- **延误治疗**：中药控制炎症强度不足（ESR/CRP达标率仅28% vs 西药89%）

- **关节破坏加速**：未联合DMARDs者5年关节融合风险增加2.3倍

** 针灸的局限性**：

- **深度刺激风险**：肥胖患者刺入过深可能损伤腰椎间盘（发生率0.3%）

- **穴位耐受性**：长期刺激同一穴位可能导致局部纤维化（需轮换取穴）

** 中西药相互作用**：

- **雷公藤制剂+MTX**：骨髓抑制风险增加5倍（需监测血常规）

- **含甘草中药+激素**：血钠潴留风险升高（老年患者尤其需要注意）

5.国际指南与中国方案对比

**1. EULAR 2022建议**：

- 中药仅作为辅助治疗（证据等级Ⅳ级）

- 针灸可用于疼痛管理（但非一线选择）

**2. 《中国强直诊疗指南》**：

- 推荐中西医结合方案（循证级别A级）

- 明确中药使用禁忌症（活动性肝炎、肾功能不全者禁用）

6.患者自我管理建议

**中药选择标准**：

- 选择CFDA批准的药物（如尪痹片、金匮肾气丸）

- 避免不明成分的民间偏方（肝毒性风险增加3倍）

**针灸治疗规范**：

- 选择正规医疗机构（执业医师操作）

- 每周2-3次，每次20分钟（避免穴位感染）

**监测体系**：

- 每月检测CRP/ESR（评估中药疗效）

- 每半年骶髂关节MRI（监测骨赘进展）

四十一、

1.强直性脊柱炎腰痛的病理机制

** 炎症驱动性疼痛**：

- **骶髂关节炎症**：炎症因子（如TNF-α、IL-6）刺激神经末梢，引发疼痛（VAS评分≥4分者占68%）

- **椎间盘退变**：炎症导致椎间盘营养供应中断，纤维环破裂风险增加3倍

** 机械性疼痛**：

- **关节强直**：脊柱活动度下降→肌肉代偿性劳损（腰大肌、竖脊肌）

- **骨质疏松**：长期炎症导致骨密度下降（Z值<-1.0者占45%）

2.锻炼的必要性及科学依据

** 疼痛缓解机制**：

- **运动疗法**：通过牵拉挛缩组织（如猫牛式伸展）降低关节内压（研究显示可减轻疼痛40%）

- **内啡肽释放**：中等强度运动（Borg评分12-14）促使内啡肽分泌，天然镇痛

** 疾病进展防控**：

- **关节保护**：规律锻炼使关节滑液分泌增加20%，延缓骨赘形成

- **脊柱灵活性**：每日30分钟瑜伽训练可使脊柱活动度维持率提高55%（5年随访数据）

3.分阶段锻炼方案

| 阶段 | 病情特点 | 推荐锻炼类型 | 频率/时长 | 禁忌动作 |

|--------------|---------------------------|------------------------------|----------------------|---------------------------|

| **急性期** | CRP>5mg/L，晨僵>1小时 | 1. 腰部冷敷（每次15分钟）<br>2. 呼吸训练（腹式呼吸10次/组） | 每日2次，每次20分钟 | 禁止弯腰、扭转、负重 |

| **缓解期** | CRP<1mg/L，晨僵<15分钟 | 1. 悬吊训练（TRX）强化核心肌群<br>2. 游泳（自由泳/蛙泳）<br>3. 等长收缩训练（仰卧抬腿） | 每周3-5次，每次45分钟 | 避免竞技性运动 |

| **慢性期** | 脊柱强直>3节段 | 1. 坐姿脊柱屈伸训练<br>2. 呼吸肌训练（使用呼吸训练器）<br>3. 平衡垫站立训练 | 每周2-3次，每次30分钟 | 严禁突然用力或快速动作 |

4.特色康复训练方法

** Maitland关节松动术**：

- **操作要点**：治疗师徒手施加3级以内的牵引力，改善骶髂关节活动度（每次10分钟，每周3次）

- **疗效**：12周后骶髂关节间隙增宽0.5-1mm（MRI证实）

**水中运动疗法**：

- **优势**：水的浮力减少关节负荷（体重减轻80%），阻力训练增强肌肉力量

- **方案**：

- 第1周：水中步行（每日15分钟）

- 第2-4周：水中太极（每周3次）

- 第5周后：水中阻力带训练（每周2次）

** 呼吸-运动联动训练**：

- **腹式呼吸训练**：仰卧位，鼻吸气时鼓腹（5次/组，每日3组）

- **呼气肌训练**：缩唇呼吸（10秒/次，每日50次）

- **疗效**：6个月后肺活量提升15%，夜间痛醒次数减少42%

5.疼痛期运动禁忌与替代方案

** 绝对禁忌动作**：

- 弯腰搬重物（脊柱屈曲>30°时椎间盘压力增加5倍）

- 仰卧起坐（增加腰椎剪切力）

- 跑步、跳跃等高冲击运动

** 疼痛期替代方案**：

- **经皮电刺激（TENS）**：缓解急性疼痛（VAS评分下降≥3分，持续4小时）

- **麦肯基力学疗法**：

- 颈椎侧屈牵引（改善脑脊液循环）

- 腰椎后伸位加压（缓解神经根压迫）

6.个性化锻炼计划制定

** 功能评估体系**：

- **Berg平衡量表**：评估跌倒风险（<40分需加强平衡训练）

- **Schober试验**：测量脊柱活动度（<5cm需专项康复）

7.长期管理建议

**运动习惯养成**：

- 使用智能手环设置每日运动提醒（目标：每周150分钟中等强度运动）

- 加入病友运动社群（线上打卡率提升50%）

**损伤预防策略**：

- 运动前动态热身（5分钟关节活动+3分钟动态拉伸）

- 运动后静态拉伸（每个肌群保持30秒）

**进阶训练时机**：

- 当BASDAI评分<1.5且CRP<1mg/L时，可逐步增加抗阻训练强度

-8.建议患者通过「AS运动康复平台」进行全程管理，包括动作矫正指导、运动数据追踪和远程康复咨询。记住：正确的锻炼是强直性脊柱炎治疗的基石，科学训练可使患者预期寿命延长8-10年（与普通人群无异）！

四十二、

1.手术必要性与适应症

**核心手术指征**：

- **关节功能丧失**：髋关节强直（活动度<30°）或脊柱固定（胸廓扩张受限<2.5cm）

- **疼痛无法控制**：VAS评分≥8分，经规范药物/物理治疗3个月无效

- **并发症处理**：

- **髋关节坏死**：股骨头塌陷（MRI T2WI信号异常）

- **脊柱感染**：椎间盘炎或椎体脓肿（发热+CRP>50mg/L）

- **神经压迫**：马尾综合征（鞍区麻木+二便失禁）

**手术类型与适应症**

| 手术类型 | 典型术式 | 适应症特征 | 临床数据支持 |

|-------------------|---------------------------|-------------------------------------|-----------------------------------|

| **关节置换术** | 全髋置换术（THA） | 髋关节强直伴疼痛（VAS≥6分） | 术后1年疼痛缓解率92%，功能恢复率85% |

| **脊柱融合术** | 腰椎后路融合术（PLIF） | 脊柱后凸畸形（Cobb角>40°） | 术后5年矫正率78%，并发症率12% |

| **椎间盘切除术** | 微创椎间盘切除术 | 椎间盘突出压迫神经根（MRI证实） | 术后VAS评分下降≥5分（89%患者） |

| **关节镜手术** | 膝关节滑膜切除术 | 滑膜炎反复发作（超声示积液>3ml） | 术后复发率<15%，疼痛缓解率60% |

2.手术时机与风险收益比

** 早期手术优势**：

- **关节破坏早期**（病程<10年）：关节软骨保存率高，术后功能恢复更佳

- **脊柱柔韧性尚存**：椎体间植骨融合成功率提升30%

**. 晚期手术挑战**：

- **骨质疏松风险**：长期炎症导致骨密度下降（Z值<-1.0者占65%）

- **神经血管粘连**：病程>15年者术中损伤风险增加2倍

**风险控制策略**：

- **术前评估**：

- **骨密度检测**：双能X线吸收法（DXA）评估骨质疏松程度

- **血管成像**：CTA评估脊柱血供（减少术中缺血风险）

- **术中技术**：

- **微创技术**：经皮螺钉固定（出血量减少40%）

- **骨水泥强化**：针对骨质疏松患者（椎体压缩性骨折风险降低50%）

3.术后管理关键点

** 术后药物调整**：

- **生物制剂**：

- 术后4-6周恢复使用（如阿达木单抗40mg qw），降低感染风险

- 联合复方新诺明（每周1次）预防机会性感染

- **NSAIDs**：

- 术后24小时启用塞来昔布200mg qd，持续4-6周

** 康复方案**：

- **早期阶段**（0-2周）：

- 伤口护理+床上被动关节活动（如踝泵运动）

- 抗凝治疗（低分子肝素）预防深静脉血栓

- **中期阶段**（2-6周）：

- 步态训练（助行器→拐杖→独立行走）

- 物理治疗（超声波治疗+中频电疗）

- **长期阶段**（6周后）：

- 有氧运动（游泳、骑自行车）+ 核心肌群训练

- 每年评估关节功能（Harris髋关节评分）

4.现代外科技术可使AS患者术后5年生存质量评分（EQ-5D）提升40%，但手术决策需严格遵循「症状驱动」原则，避免过度治疗！

Kimi

一、

1.肌肉骨骼问题

- **姿势不良**：长时间保持不正确的姿势，如久坐、弯腰驼背等，可导致腰部肌肉持续处于紧张状态，进而引发腰背疼痛。

- **过度劳累或运动损伤**：过度使用腰部肌肉，如长时间搬重物、突然进行剧烈运动等，可能导致肌肉拉伤、韧带损伤，引起腰背疼痛。

- **脊柱结构异常**：如先天性脊柱侧弯、腰椎前凸等，会改变脊柱的正常受力分布，导致腰背疼痛。

2.### 脊柱关节炎相关

- **强直性脊柱炎**：主要表现为慢性腰背疼痛，通常在休息时加重，活动后缓解，疼痛可能伴有晨僵，且多发生在夜间，严重影响睡眠质量。

- **反应性关节炎**：通常与感染有关，如肠道感染、泌尿生殖道感染后，可能出现腰背疼痛，同时伴有下肢关节炎、结膜炎等症状。

- **银屑病关节炎**：部分患者除了皮肤银屑病表现外，还会出现腰背疼痛，常伴有指甲改变，如顶针样改变等。

- **未分化脊柱关节病**：有脊柱关节病的某些特征，但不符合某一特定疾病的诊断标准，也会出现腰背疼痛等症状。

3.其他疾病影响

- **腰椎间盘突出症**：当腰椎间盘发生退变或损伤时，髓核可能突出压迫周围神经，引发腰背疼痛，同时伴有下肢放射性疼痛、麻木等症状。

- **腰椎管狭窄症**：由于腰椎管狭窄导致脊髓或神经根受压，患者常出现腰背疼痛、间歇性跛行，行走时疼痛加重，休息后缓解。

- **类风湿关节炎**：主要累及小关节，但也可影响到脊柱等大关节，引起腰背疼痛，常伴有晨僵、关节肿胀、畸形等表现。

- **骨质疏松症**：骨密度降低，骨骼变得脆弱，轻微的活动或外力就可能导致腰椎等部位的骨折，从而引起腰背疼痛，疼痛程度可能较重，且伴有身高缩短、驼背等。

4.以上是一些常见的导致腰背疼痛的原因，但具体到个人，可能需要结合详细的病史、体格检查以及必要的影像学和实验室检查来明确诊断。如果腰背疼痛持续不缓解或伴有其他症状，建议及时就医。

第二题、

1.强直性脊柱炎

- **典型症状**：夜间痛是其常见的表现之一，疼痛通常在休息、静止时加重，活动后可缓解，还常伴有晨僵，即早晨起床时感觉腰部僵硬，活动受限。

- **发病人群**：多见于青少年和青壮年，男性发病较多。

- **其他表现**：除了腰背疼痛，还可能出现臀部、髋部疼痛，严重时可累及胸椎、颈椎，导致胸部疼痛、活动受限、颈椎疼痛和僵硬等。部分患者还可能出现虹膜炎等关节外表现。

2.未分化脊柱关节病

- **症状特点**：也会出现腰背疼痛，夜间痛和活动后缓解的情况与强直性脊柱炎类似，还可能伴有下肢关节疼痛、肿胀等。

- **发病人群**：和强直性脊柱炎类似，多见于青少年和青壮年。

- **其他表现**：可能出现疲劳、晨僵等症状，但病情相对较轻且复杂多样，因个体差异较大而不易明确归类。

3.无论是哪种疾病，都建议你及时就医，通过详细的检查和专业的诊断来明确病因，并采取相应的治疗措施。

第三题、

1.肌肉骨骼问题

- **姿势不良**：睡觉时姿势不当，如枕头过高、过低或床垫不合适，可能导致肌肉、韧带处于紧张状态，长时间压迫关节，引起晨僵。

- **过度劳累或运动损伤**：前一天过度运动或劳累，致肌肉微小损伤、痉挛，产生乳酸等代谢产物，堆积后晨起时肌肉僵硬疼痛，活动后代谢产物减少、血液循环恢复，僵硬感缓解。

2.关节炎相关

- **类风湿关节炎**：晨僵是其突出症状，常持续1小时以上，手、脚小关节僵硬、疼痛、肿胀，病情重时可影响全身关节，还可能伴有乏力、低热等。

- **骨关节炎**：多见于中老年人，晨僵时间较短，通常不超过30分钟，主要因关节软骨退变、磨损，关节周围炎症、滑膜渗出引起关节周围组织粘连、挛缩，活动后症状减轻。

- **强直性脊柱炎**：主要累及脊柱、骶髂关节等中轴关节，晨僵明显，可伴有腰背部疼痛、僵硬，病情进展可致脊柱强直、畸形，还可能有眼部病变、心血管病变等关节外表现。

3.其他疾病影响

- **纤维肌痛综合征**：以全身广泛性疼痛、僵硬为主要表现，常伴有疲劳、睡眠障碍、焦虑等，晨起时症状明显，可能与神经内分泌失调、睡眠质量差有关。

- **系统性红斑狼疮**：是一种自身免疫性疾病，可累及全身多个系统，部分患者可能出现晨僵，常伴有面部红斑、关节疼痛、发热、乏力等症状。

4.如果晨僵症状频繁出现且持续时间较长，建议及时就医，进行详细检查和专业诊断，以便明确病因并采取针对性治疗措施。

第四题、

1.腰疼、眼睛痒和拉肚子这些症状看似不相关，但在某些情况下可能的关联情况

- **系统性疾病**：如系统性红斑狼疮等自身免疫性疾病，可能累及多个系统，出现腰疼、眼部病变和胃肠道症状。此外，一些感染性疾病，如肠道感染，病原体或其毒素可能通过血液循环影响到眼部和腰部，引发相应症状。

- **过敏反应**：如果对某些食物、药物或环境中的过敏原过敏，可能出现眼睛痒、腹泻等症状，同时可能伴有腰疼等其他不适。

- **其他疾病**：如强直性脊柱炎等脊柱关节炎，主要表现为腰背疼痛和僵硬，严重时可能影响到眼部，导致虹膜炎等眼部病变，出现眼睛痒、红肿等症状。而拉肚子可能是由于合并了其他胃肠道疾病或受到感染。

2.建议

- **记录症状**：注意记录腰疼、眼睛痒和拉肚子的具体情况，包括发作时间、频率、持续时间、诱发因素等，以便就医时提供详细信息。

- **就医检查**：如果这些症状持续存在或加重，建议及时就医，进行全面检查，如血常规、尿常规、粪便常规、自身抗体检测、影像学检查等，以明确病因。

- **专科就诊**：根据主要症状，可先选择相应科室，如腰疼明显可先去骨科或风湿免疫科，眼睛痒可去眼科，拉肚子可去消化内科。在医生的指导下进行进一步检查和诊断。

第五题、

1. 强直性脊柱炎

- **特点**：主要表现为慢性腰背疼痛，通常在休息时加重，活动后缓解，疼痛可能伴有晨僵，且多发生在夜间，严重影响睡眠质量。还可能出现臀部、髋部疼痛，严重时可累及胸椎、颈椎，导致胸部疼痛、活动受限、颈椎疼痛和僵硬等。

- **检查**：影像学检查如X光、MRI可显示骶髂关节和脊柱的病变，实验室检查HLA-B27基因检测呈阳性。

2.腰椎间盘突出症

- **特点**：当腰椎间盘发生退变或损伤时，髓核可能突出压迫周围神经，引发腰背疼痛，同时伴有下肢放射性疼痛、麻木等症状。

- **检查**：通过CT或MRI检查可明确显示腰椎间盘突出的位置和程度。

3.腰椎管狭窄症

- **特点**：由于腰椎管狭窄导致脊髓或神经根受压，患者常出现腰背疼痛、间歇性跛行，行走时疼痛加重，休息后缓解。

- **检查**：影像学检查如X光、CT、MRI可显示腰椎管狭窄的情况。

4.类风湿关节炎

- **特点**：主要累及小关节，但也可影响到脊柱等大关节，引起腰背疼痛，常伴有晨僵、关节肿胀、畸形等表现。

- **检查**：血液检查可检测到类风湿因子、抗CCP抗体等自身抗体，影像学检查可显示关节病变。

5.骨质疏松症

- **特点**：骨密度降低，骨骼变得脆弱，轻微的活动或外力就可能导致腰椎等部位的骨折，从而引起腰背疼痛，疼痛程度可能较重，且伴有身高缩短、驼背等。

- **检查**：骨密度检查可明确诊断骨质疏松症，X光检查可显示骨质疏松的程度。

6.如果腰疼持续不缓解或伴有其他症状，建议及时就医，通过详细的检查和专业的诊断来明确病因，并采取相应的治疗措施。

第六题、

1. 可以先观察、暂时不需要就医的情况

- **轻微损伤**：如果腰痛是由于明显的轻微外伤，如偶尔的扭伤或过度运动导致，且疼痛程度较轻，可先自行观察，适当休息、热敷或使用一些非处方的止痛药（如布洛芬）进行对症处理，看症状是否能够缓解。

- **姿势不良**：长时间保持不良姿势后出现的腰痛，可在调整姿势、注意腰背部肌肉锻炼后观察疼痛是否减轻。

- **偶尔的肌肉酸痛**：如果腰痛只是偶尔出现的轻微肌肉酸痛，没有其他伴随症状，可先通过休息、按摩等方式缓解。

2.需要及时就医的情况

- **疼痛严重**：如果腰痛非常严重，难以忍受，影响正常生活和睡眠，甚至需要卧床休息，应尽快就医。

- **持续时间长**：腰痛持续时间超过2周，或呈现进行性加重的趋势，需要引起重视。

- **伴有下肢放射痛**：如果腰痛伴有沿坐骨神经路径向下肢放射的剧烈疼痛，可能提示腰椎间盘突出症等疾病，需要及时就医明确诊断。

- **伴有大小便失禁或性功能障碍**：这可能是马尾神经综合征的表现，是一种急症，需要立即就医进行治疗。

- **伴有发热、体重减轻等全身症状**：可能提示存在感染、肿瘤等严重疾病，应尽快就医进行相关检查。

- **外伤后腰痛**：如果腰痛是在明显的外伤后出现，如高处坠落、车祸等，需要警惕腰椎骨折等可能，应立即就医。

- **既往有相关病史**：如果本身有腰椎疾病史，如腰椎间盘突出症、腰椎管狭窄症等，此次腰痛加重或出现新的症状，建议及时就医调整治疗方案。

3.如果不确定自己的腰痛情况是否需要就医，也可以先咨询一下家庭医生或通过互联网医疗平台向专业医生进行初步咨询。

第七题、

1.可能的原因

- **肌肉骨骼问题**：如姿势不良、过度劳累、肌肉拉伤等，这是背痛最常见的原因，通常通过休息、热敷、按摩等可缓解。

- **强直性脊柱炎**：如果家族中有强直性脊柱炎患者，你患此病的风险可能会略高于普通人群。但该疾病的确切发病机制尚不完全明确，除遗传因素外，还涉及环境等多因素。

- **其他脊柱疾病**：如腰椎间盘突出症、腰椎管狭窄症等，这些疾病也会引起背痛，且症状可能与强直性脊柱炎部分相似。

2.建议

- **观察症状**：如果背痛伴有晨僵（晨起时背部僵硬，活动后缓解）、夜间痛加重、休息后无缓解且持续数周以上，建议及时就医。

- **就医检查**：可通过血沉、C反应蛋白、HLA-B27基因检测等血液检查，以及X光、MRI等影像学检查，帮助判断是否为强直性脊柱炎或其他脊柱疾病。

- **不要过度担忧**：背痛是常见症状，即使家族中有强直性脊柱炎患者，也不必过度紧张。保持良好生活习惯、正确姿势，适当进行背肌锻炼，可预防和缓解大部分背痛。

第八题、强直性脊柱炎的背痛与其他腰背痛在成因、症状表现及发病机制等方面存在差异：

1.痛感

- **夜间痛**：强直性脊柱炎患者常在夜间疼痛加剧，严重时可致患者痛醒，活动或服止痛药后痛感缓解。

- **晨僵**：晨起时，患者脊柱、骶髂关节等部位僵硬，活动受限，需活动一段时间后才能缓解或消失，僵硬程度与病情轻重相关。

- **持续性**：强直性脊柱炎的背痛通常持续时间较长，若不及时治疗和控制，可能逐渐发展为慢性持续性疼痛，甚至导致脊柱强直、畸形。

2.其他症状

- **关节外表现**：部分强直性脊柱炎患者会出现眼部病变，如虹膜炎，表现为眼睛红、痛、畏光、流泪等；还可能出现心脏病变，如主动脉根炎、心脏传导阻滞等，表现为心悸、胸闷、气短等症状。

- **脊柱及关节活动受限**：随着病情进展，患者脊柱的灵活性逐渐下降，腰部前屈、后伸、侧弯等活动受限，颈椎活动也可能受限，严重时整个脊柱强直，导致患者无法正常转身、弯腰等。同时，髋关节等外周关节也可能受累，出现疼痛、肿胀、活动受限等症状。

3.疾病性质

- **炎症性**：强直性脊柱炎是一种慢性炎症性疾病，主要累及脊柱、骶髂关节等中轴关节。其背痛是由关节及周围软组织的炎症反应引起的，这种炎症会导致关节滑膜增生、充血、渗出等，进而刺激神经末梢，产生疼痛。

- **全身性**：该疾病虽主要表现为脊柱和关节症状，但作为一种自身免疫性疾病，它还可能影响全身多个系统和器官，如眼部、心血管系统、肺部等，引发相应的病变和症状。

4.如果怀疑自己可能患有强直性脊柱炎，建议及时就医，通过专业检查和诊断来明确病情，并采取相应的治疗措施。

第九题、

1. **胸椎受累**：强直性脊柱炎可引起胸椎的炎症和病变，导致胸椎活动受限和疼痛。患者可能会感到胸部僵硬和疼痛，尤其是在夜间或长时间保持一个姿势后。

2.- **肋椎关节炎**：强直性脊柱炎可累及肋椎关节，导致肋骨和胸椎之间的关节炎症，引起胸部疼痛。

-3. **附着点炎**：强直性脊柱炎常引起附着点炎，即肌腱、韧带等软组织附着在骨骼上的部位发生炎症。在胸部，如肋软骨与胸骨的连接处发生附着点炎，可导致胸痛，尤其在深呼吸、咳嗽或活动时加重。

4.需要注意的是，胸口疼痛也可能由其他原因引起，如心血管疾病、消化系统问题等。如果医生怀疑是强直性脊柱炎，通常会结合病史、体格检查、影像学检查（如X光、MRI）和实验室检查（如HLA-B27基因检测）等来综合判断。

第十题、

1.情绪压力导致肌肉紧张

- **机制**：当人处于情绪压力下时，身体会处于一种紧张的状态，这种紧张会反射性地引起肌肉收缩。长期的肌肉收缩会导致肌肉疲劳、僵硬，进而引发腰疼。

- **举例**：比如一个人在工作压力大的时候，可能会不自觉地紧绷背部肌肉，时间一长，就会出现腰部酸痛、僵硬不适等症状。

2.情绪压力影响睡眠质量

- **机制**：压力大时，大脑处于兴奋状态，难以放松，导致睡眠质量下降，睡眠不足或睡眠质量差会使身体的修复和恢复过程受阻，肌肉、韧带等组织得不到充分的休息和修复，从而增加腰疼的发生概率。

- **举例**：如果一个人因为情绪压力而长期失眠或睡眠不好，第二天往往会感觉腰部乏力、疼痛。

3.情绪压力引发内分泌失调

- **机制**：长期的情绪压力会影响内分泌系统的平衡，导致激素水平的变化。例如，压力会促使肾上腺皮质激素的分泌增加，长期过量的皮质激素会影响钙的吸收和利用，导致骨质疏松，使腰椎等骨骼变得脆弱，容易引发腰疼。

- **举例**：一些长期处于高压状态的女性，可能会出现月经不调等内分泌失调的表现，同时伴有腰疼症状。

4.情绪压力降低疼痛阈值

- **机制**：情绪压力会改变人对疼痛的感知，使疼痛阈值降低，即使是一些轻微的腰部不适或损伤，也会被放大为明显的腰疼。

- **举例**：在情绪压力下，原本可能感觉不到的轻微腰部肌肉拉伤，现在会变得非常疼痛。

5.情绪压力影响免疫系统

- **机制**：长期的情绪压力会削弱免疫系统的功能，使身体更容易受到各种疾病的侵袭，包括一些可能导致腰疼的疾病，如强直性脊柱炎等自身免疫性疾病。

- **举例**：一些人在长期压力下，身体免疫力下降，患上了强直性脊柱炎，从而出现腰疼、僵硬等症状。

6.虽然情绪压力可能导致腰疼，但腰疼也可能是由其他多种原因引起的，如姿势不良、过度劳累、腰椎疾病等。如果腰疼症状持续存在或加重，建议及时就医，进行全面检查，以明确病因并采取相应的治疗措施。

十一、

1.HLA-B27阳性本身并不直接意味着患有某种疾病，而是一个与多种疾病相关的遗传标记。HLA-B27是一种人类白细胞抗原（HLA）的亚型，存在于人体免疫系统中。HLA系统在免疫反应中起着重要作用，帮助识别和抵御外来病原体。

2. HLA-B27与强直性脊柱炎的关系

HLA-B27与强直性脊柱炎（AS）密切相关。研究表明，HLA-B27阳性者患强直性脊柱炎的风险显著高于阴性者。HLA-B27是强直性脊柱炎的遗传易感因素之一，但它并不是唯一的决定因素。并不是所有HLA-B27阳性者都会发展为强直性脊柱炎，也不是所有强直性脊柱炎患者都是HLA-B27阳性。

3. HLA-B27与其它疾病的关系

HLA-B27还与一些其他疾病相关，如反应性关节炎、银屑病关节炎等。这些疾病通常具有免疫介导的炎症性特征，HLA-B27在其中可能起到一定的作用。

4.HLA-B27阳性需要注意什么

如果HLA-B27检测结果为阳性，意味着你可能具有较高的患某些疾病的风险，但并不意味着你已经患有这些疾病。以下是一些建议：

5.定期体检**：定期进行身体检查，关注关节、脊柱等部位的健康状况，以便及时发现异常。

- **注意症状**：如果出现腰背部疼痛、僵硬、关节肿胀等症状，应及时就医，并告知医生HLA-B27阳性的情况。

- **保持健康生活方式**：保持适当的运动，增强体质，避免过度疲劳和感染，有助于降低发病风险。

- **避免不必要的X射线暴露**：虽然X射线检查在诊断中有时是必要的，但应尽量减少不必要的暴露，以降低潜在的健康风险。

十二、

1.C-反应蛋白（CRP）和血沉（ESR）都是炎症指标，它们的升高通常意味着体内存在炎症反应。在强直性脊柱炎（AS）中，这两个指标可能会升高，但它们并不是特异性指标，也就是说，它们的升高并不一定意味着你患有AS，因为它们也可能与其他炎症性疾病相关。

2. C-反应蛋白（CRP）

- **正常范围**：通常小于10 mg/L。

- **升高意义**：CRP是一种急性期蛋白，当体内存在炎症、感染或组织损伤时，肝脏会迅速合成CRP并释放到血液中，使CRP水平显著升高。在AS中，由于存在慢性炎症，CRP可能会持续升高。

3. 血沉（ESR）

- **正常范围**：男性通常小于15 mm/h，女性通常小于20 mm/h。

- **升高意义**：血沉是红细胞在一定条件下沉降的速度，反映了体内炎症的程度。在AS中，由于炎症因子的作用，血沉可能会加快。

4. 与强直性脊柱炎的关系

- **炎症活动性**：在AS中，CRP和血沉的升高通常与疾病的活动性有关。当病情加重或处于活动期时，这些指标可能会明显升高。

- **诊断辅助**：虽然CRP和血沉不是AS的特异性诊断指标，但它们可以作为辅助诊断的参考。结合临床症状（如腰背部疼痛、僵硬、关节肿胀等）、影像学检查（如X光、MRI等）以及HLA-B27检测结果，医生可以更全面地评估是否患有AS。

5. 其他可能的原因

- **感染**：细菌或病毒感染也可能导致CRP和血沉升高。

- **其他炎症性疾病**：如类风湿关节炎、系统性红斑狼疮等自身免疫性疾病，也可能引起这些指标的升高。

- **组织损伤**：手术、创伤等导致的组织损伤，也会使CRP和血沉升高。

6.如果你的CRP和血沉都升高，并且伴有腰背部疼痛、僵硬等症状，建议及时就医，进行进一步的检查，如影像学检查和HLA-B27检测，以便明确诊断。医生会综合考虑你的临床表现和检查结果，给出准确的诊断和治疗方案。

十三、

1.疾病早期

- **炎症初期**：在强直性脊柱炎等疾病的早期，炎症可能仅累及软组织，如肌肉、韧带、肌腱等，而尚未累及骨骼。X光片主要显示骨骼结构，对于软组织的炎症和损伤不敏感，因此可能无法发现异常。

- **骨质改变不明显**：即使炎症已经开始影响骨骼，但在疾病早期，骨质的改变可能非常细微，X光片的分辨率有限，可能无法检测到这些微小的变化。

2.疾病性质

- **非结构性炎症**：有些疾病引起的疼痛是由于非结构性的炎症，如肌肉筋膜炎、纤维肌痛综合征等。这些炎症不会引起骨骼的结构改变，因此X光片无法显示异常。

- **神经源性疼痛**：某些情况下，疼痛可能是由于神经受压、神经炎等神经源性因素引起的。X光片无法直接显示神经的情况，因此无法发现导致疼痛的原因。

3.检查技术限制

- **X光片的局限性**：X光片是一种二维成像技术，对于某些部位的观察可能存在局限性。例如，对于脊柱的某些节段或关节的某些角度，可能因为投影的关系而无法清晰显示。

- **其他影像学检查更敏感**：与X光片相比，磁共振成像（MRI）对软组织和早期骨髓水肿等改变更为敏感。如果临床怀疑有病变，但X光片未显示异常，可以考虑进行MRI检查以进一步明确诊断。

4.个体差异

- **疼痛阈值**：不同患者的疼痛阈值不同，有些人对疼痛更为敏感，即使病变较轻也可能感到明显的疼痛。

- **心理因素**：心理状态也会影响疼痛的感知。焦虑、抑郁等心理因素可能会放大疼痛的感受，使患者感到疼痛更为剧烈。

5.如果患者疼痛明显但X光片未显示异常，建议与医生详细沟通，结合临床症状和其他检查结果（如血液检查、MRI等）进行综合判断，以明确病因并制定合适的治疗方案。

十四、

1.骨髓水肿是一种病理状态，通常是指骨髓内液体积聚增加，导致骨髓信号在影像学检查（如磁共振成像，MRI）上出现异常改变。骨髓水肿的严重性取决于其具体病因和病情，而骨髓水肿是否与强直性脊柱炎有关，需要结合临床表现、影像学检查和其他相关检查进行综合判断。

2. 骨髓水肿的严重程度

骨髓水肿的严重程度可以因多种因素而异，具体如下：

- **疼痛程度**：骨髓水肿常伴有明显的疼痛，疼痛的程度和性质可以作为评估严重程度的一个指标。如果疼痛剧烈且持续时间长，可能提示病情较为严重。

- **活动受限**：骨髓水肿可能导致关节活动受限，影响日常活动和生活质量。如果活动受限严重，可能需要更积极的治疗。

- **影像学表现**：在MRI上，骨髓水肿表现为T1加权像上的低信号和T2加权像上的高信号。信号改变的范围和强度可以作为评估严重程度的参考。

3.骨髓水肿与强直性脊柱炎的关系

骨髓水肿在强直性脊柱炎中具有一定的意义，具体如下：

- **早期炎症表现**：在强直性脊柱炎的早期，骨髓水肿可能是炎症活动的表现之一。它通常出现在骶髂关节或脊柱的受累部位，提示炎症的存在和活动性。

- **疾病进展指标**：骨髓水肿的持续存在可能与疾病的进展有关。一些研究表明，骨髓水肿的出现可能预示着后续的骨质破坏和强直的发生。

- **诊断依据之一**：结合临床症状（如腰背部疼痛、僵硬）、实验室检查（如HLA-B27阳性、CRP和血沉升高）以及其他影像学表现，骨髓水肿可以作为强直性脊柱炎诊断的一个重要参考指标。

4.骨髓水肿的其他原因

骨髓水肿并不特异于强直性脊柱炎，还可能由其他多种原因引起，具体如下：

- **外伤**：骨折、关节扭伤等外伤可能导致骨髓水肿，通常在受伤后短期内出现，随着伤情恢复逐渐消退。

- **其他炎症性疾病**：如类风湿关节炎、骨关节炎等，也可能引起骨髓水肿，需要结合临床表现和其他检查进行鉴别诊断。

- **代谢性疾病**：某些代谢性疾病，如骨质疏松症，也可能导致骨髓水肿。

5.如果发现骨髓水肿，建议及时就医，进行详细的临床评估和必要的检查，以明确病因并制定合适的治疗方案。

十五、

1. 临床表现

- **腰背痛和僵硬**：这是强直性脊柱炎的常见早期症状，通常在早晨起床时或长时间不活动后更为明显，活动后可缓解。

- **关节疼痛和肿胀**：除了腰背部，还可能出现髋关节、膝关节等其他关节的疼痛和肿胀。

- **脊柱活动受限**：随着病情进展，脊柱的活动范围可能会受到限制，如前屈、后伸、侧弯等动作可能受限。

- **其他症状**：部分患者可能出现疲劳、体重减轻、发热等全身症状，以及眼部炎症（如虹膜炎）、心血管疾病等关节外表现。

2.体征

- **骶髂关节压痛**：医生通过检查骶髂关节区域的压痛来评估是否存在炎症。

- **脊柱活动度评估**：包括前屈、后伸、侧弯等动作的评估，以确定脊柱的活动范围是否受限。

- **胸廓扩张度**：测量胸廓的扩张度，评估胸廓的活动是否受限。

3.实验室检查

- **HLA-B27检测**：HLA-B27阳性与强直性脊柱炎密切相关，但并非所有阳性者都会患病，也不是所有患者都是阳性。

- **炎症指标**：如C-反应蛋白（CRP）和血沉（ESR），这些指标升高提示体内存在炎症反应。

4.影像学检查

- **X光检查**：骶髂关节和脊柱的X光片可以显示骨质破坏、骨质增生、韧带钙化等改变，是诊断强直性脊柱炎的重要依据之一。

- **磁共振成像（MRI）**：对早期炎症改变更为敏感，可以发现X光片无法显示的骨髓水肿、软组织炎症等。

5.国际标准

根据国际脊柱关节炎评估工作组（ASAS）的诊断标准，强直性脊柱炎的诊断需要满足以下条件之一：

- 骶髂关节炎症的影像学证据（通过X光或MRI确认）加上至少1个脊柱关节炎的特征（如腰背痛、关节炎、虹膜炎、HLA-B27阳性、家族史等）。

- 对于没有影像学证据的患者，临床医生可能会参考其他症状和检查结果进行综合判断。

6.就医建议

如果你怀疑自己可能患有强直性脊柱炎，建议及时就医，前往风湿免疫科或相关专科进行详细检查和诊断。医生会根据你的具体情况进行综合评估，并制定相应的治疗方案。

十六、

1. X光检查

- **作用**：X光检查是初步的影像学评估手段，对于观察骨骼结构的完整性、关节间隙的变化以及有无明显的骨折或骨质破坏等具有重要价值。

- **必要性**：如果你存在关节疼痛、肿胀或外伤等情况，X光可以帮助医生初步判断是否存在明显的骨骼异常。

2. CT检查

- **作用**：CT检查能够提供更详细的横断面图像，对于观察骨骼的细微结构、发现X光难以显示的病变（如椎体骨折、关节面的细微破坏等）具有优势。

- **必要性**：当X光检查结果不明确，或者医生怀疑存在更复杂的骨骼病变时，CT检查可以提供更准确的信息。

3. 磁共振检查（MRI）

- **作用**：MRI对软组织的分辨能力非常强，能够清晰显示肌肉、韧带、关节囊、椎间盘等软组织结构，以及骨髓水肿等早期炎症改变。

- **必要性**：对于一些需要明确软组织损伤程度、评估关节炎症活动性或排除早期骨髓水肿等情况，MRI是不可或缺的检查手段。

4. 抽血化验

- **作用**：抽血化验可以检测多种指标，如炎症因子（C反应蛋白、血沉等）、自身抗体（类风湿因子、抗CCP抗体等）、代谢指标等，有助于判断体内是否存在炎症、免疫异常或代谢紊乱等情况。

- **必要性**：如果你有不明原因的疼痛、疲劳、关节肿胀等症状，抽血化验可以帮助医生明确病因，排除其他可能的疾病。

5.综合考虑

- **医生的建议**：医生开具这些检查通常是基于你的临床表现和初步诊断，为了更全面地了解你的病情，制定合适的治疗方案。

- **病情的复杂性**：有些疾病在早期或某些阶段可能不会在所有检查中都表现出异常。例如，强直性脊柱炎在早期，X光和CT可能无法发现明显病变，而MRI和抽血化验（如HLA-B27检测）可能更有助于诊断。

- **检查的互补性**：不同的检查方法有其独特的优势和局限性，它们之间是互补的。综合运用多种检查手段可以提高诊断的准确性和全面性。

6.建议

如果你对医生开具的检查项目有疑问或顾虑，可以与医生进行沟通，了解每项检查的具体目的和必要性。通常情况下，为了确保准确诊断和后续的有效治疗，按照医生的建议完成所有检查是比较稳妥的选择。

十七．

1.强直性脊柱炎的发病年龄特点

- **常见发病年龄**：强直性脊柱炎通常在青少年晚期或成年早期发病，发病高峰年龄在20-30岁之间。

- **儿童发病情况**：虽然较少见，但强直性脊柱炎也可以在儿童期发病，通常称为幼年型强直性脊柱炎。

- **中年及以后发病情况**：30岁以后发病者相对较少，但也有部分患者在40岁甚至50岁以后出现症状。随着年龄的增长，发病概率相对降低，但并不能完全排除。

2. 影响发病年龄的因素

- **遗传因素**：HLA-B27基因与强直性脊柱炎的发病密切相关。携带该基因的人群发病风险更高，但具体发病年龄还受到其他遗传因素和环境因素的共同影响。

- **环境因素**：感染、外伤等环境因素可能诱发强直性脊柱炎的发病。例如，某些细菌感染可能触发机体的免疫反应，导致关节炎症。

- **免疫系统变化**：随着年龄的增长，免疫系统的功能会发生变化，可能影响疾病的发生和发展。

3. 成年人患病的特点

- **症状表现**：成年人患强直性脊柱炎的症状可能与青少年相似，如腰背部疼痛、僵硬、关节肿胀等，但也可能因个体差异而有所不同。

- **诊断和治疗**：诊断时需要综合考虑临床表现、影像学检查和实验室检查结果。治疗方面，成年人可能需要更个体化的治疗方案，包括药物治疗、物理治疗等。

4.如果你有腰背部疼痛、僵硬或其他关节症状，建议及时就医，进行详细的检查和诊断。医生会根据你的具体情况，判断是否为强直性脊柱炎，并制定相应的治疗方案。

十八.

1.其他脊柱关节疾病

- **类风湿关节炎**：虽然类风湿关节炎主要影响手足等小关节，但当强直性脊柱炎累及周围关节时，可能被误诊为类风湿关节炎。类风湿关节炎通常有类风湿因子阳性，而强直性脊柱炎则多为阴性。

- **银屑病关节炎**：部分强直性脊柱炎患者可能伴有银屑病样皮疹，容易被误诊为银屑病关节炎。银屑病关节炎的皮疹通常更为典型，且关节受累形式与强直性脊柱炎有所不同。

2.腰椎疾病

- **腰椎间盘突出症**：强直性脊柱炎患者可能出现腰背部疼痛和僵硬，与腰椎间盘突出症的症状相似。腰椎间盘突出症通常有明显的放射性疼痛，且影像学检查可显示椎间盘突出等改变。

- **腰椎管狭窄症**：该病也可引起腰背部疼痛和下肢症状，需要与强直性脊柱炎进行鉴别。腰椎管狭窄症的影像学检查有其特征性表现。

3.其他炎症性疾病

- **反应性关节炎**：反应性关节炎常在感染后发生，伴有关节疼痛和炎症，可能与强直性脊柱炎混淆。反应性关节炎通常有明确的感染史，且HLA-B27阳性率相对较低。

- **肠病性关节炎**：如克罗恩病、溃疡性结肠炎等引起的关节炎，可能与强直性脊柱炎有相似的症状。肠病性关节炎通常伴有肠道疾病的典型表现。

4.软组织损伤

- **肌肉拉伤或扭伤**：如果患者近期有运动损伤或外伤史，腰背部的疼痛可能被误认为是肌肉拉伤或扭伤。肌肉损伤通常有明确的外伤史，且疼痛和压痛多局限于损伤部位。

5.代谢性疾病

- **骨质疏松症**：骨质疏松症可引起腰背部疼痛，尤其在老年人中较为常见。骨质疏松症的骨密度检查可显示骨量减少，而强直性脊柱炎则有其特有的影像学表现。

6.如果怀疑自己可能被误诊，建议及时就医，并与医生详细沟通自己的症状、病史和检查结果，以便进行准确的诊断和治疗。

十九.

1. **详细记录症状**：持续记录你的症状，包括疼痛的位置、强度、持续时间、缓解因素等，这有助于医生更全面地了解你的状况。

2. **寻求第二意见**：可以咨询其他风湿病专家，特别是那些专门研究脊柱关节疾病的医生，他们可能会提供不同的视角和诊断方法。

3. **复查和进一步检查**：要求进行更深入的检查，如MRI等高级影像学检查，以及更全面的实验室检查，以排除或确认强直性脊柱炎或其他类似疾病。

4. **关注其他可能的疾病**：考虑到强直性脊柱炎的症状可能与其他疾病重叠，医生可能会建议检查其他潜在的疾病，以确保没有遗漏。

5. **与医生深入沟通**：和医生讨论你的担忧和症状，了解他们排除强直性脊柱炎的具体理由，以及他们认为更可能的诊断是什么。

6.请记住，医生的诊断是基于你的症状、体检结果、检查报告等多方面因素综合考虑的。如果多位医生都认为你不太可能患有强直性脊柱炎，可能需要考虑其他原因导致你的症状。但如果你仍然感到不确定，积极与医疗提供者沟通，进行进一步的评估和检查，以确保得到准确的诊断和适当的治疗。

二十、

1.- **非处方止痛药**：如果疼痛难以忍受，可以考虑使用非处方的止痛药物，如对乙酰氨基酚（扑热息痛）等。但请注意，这类药物主要用于缓解轻至中度疼痛，且长期或过量使用可能带来肝肾功能损害等副作用。

-2. **非甾体抗炎药（NSAIDs）**：如布洛芬、双氯芬酸等，这类药物具有抗炎、镇痛的作用，对于一些炎症性疼痛可能有效。但同样，它们可能引起胃肠道不适、溃疡、出血等副作用，需谨慎使用。

- 3.**休息和保护**：在疼痛期间，适当休息，避免过度活动或劳累，减轻受累部位的负担，有助于缓解疼痛。可以使用热敷、冷敷等物理方法缓解疼痛和炎症。

- 4.**咨询医生或药师**：在使用任何药物之前，最好咨询医生或药师的意见，特别是如果你有其他健康问题或正在服用其他药物，他们可以根据你的具体情况提供更安全、合适的建议。

5.请记住，药物只是缓解症状的手段之一，关键是尽快明确诊断，针对性地进行治疗。

二十一.

1. X光检查

- **剂量**：普通胸部X光片的辐射剂量大约在0.1毫西弗（mSv）左右，腹部X光片的剂量可能稍高一些，约0.5-1.0mSv。

- **影响**：这个剂量相对较低，一般认为对健康的影响较小。不过，如果频繁进行X光检查，射线的累积效应可能会增加潜在风险。

2.CT检查

- **剂量**：CT检查的辐射剂量通常比普通X光片高。例如，头部CT的剂量大约是2-3mSv，腹部CT可能达到5-10mSv，胸部CT约5-7mSv。

- **影响**：CT检查的辐射剂量相对较高，可能会增加患癌症等疾病的风险，但这种风险在单次检查时仍然是相对较低的。在必要时，医生会权衡检查的必要性和潜在风险。

3.核磁共振成像（MRI）

- **剂量**：MRI检查不使用X射线，因此没有辐射剂量。

- **影响**：MRI是一种相对安全的检查方式，对健康没有已知的直接辐射风险，但其检查环境对某些人群（如装有心脏起搏器等金属植入物的人）可能不适合。

4.骨密度检查

- **剂量**：骨密度检查通常使用双能X线吸收法（DEXA），其辐射剂量非常低，大约在0.001-0.003mSv。

- **影响**：由于剂量极低，对健康的影响可以忽略不计。

5.需要注意的是，虽然单次检查的辐射剂量可能较低，但如果在短时间内接受了多次放射性检查，射线的累积剂量可能会增加潜在的健康风险。在进行任何医学检查前，建议与医生充分沟通，了解检查的必要性和潜在风险，权衡利弊后再做决定。

二十二.

1.诊断标准的综合性

- **临床表现**：强直性脊柱炎的诊断主要依据慢性下腰痛、晨僵、脊柱和关节活动受限等特征。如果你的症状不完全符合，或者医生认为症状可能由其他原因引起，可能不会轻易诊断。

- **体征**：医生通过检查发现骶髂关节压痛、脊柱活动度受限等体征。如果体征不典型或不明显，会影响诊断。

- **实验室检查**：HLA-B27阳性虽与强直性脊柱炎相关，但并非特异性指标。血常规异常可能由多种疾病引起，需结合其他检查综合判断。

- **影像学检查**：X光或MRI检查骶髂关节和脊柱，若未发现明确的骨质破坏、骨质增生、韧带钙化或骨髓水肿等改变，单凭症状和实验室检查难以确诊。

2. HLA-B27阳性的非特异性

- **其他疾病关联**：HLA-B27阳性还与反应性关节炎、银屑病关节炎等疾病相关。若你有相关疾病表现，可能需进一步检查以明确诊断。

- **携带者存在**：部分HLA-B27阳性者是携带者，不发展为强直性脊柱炎。若无家族史、典型症状或影像学证据，医生可能不轻易诊断。

3.血常规异常的多样性

- **其他病因提示**：血常规异常可能提示感染、贫血、血小板减少等其他疾病，需先排除这些疾病。

- **炎症指标参考性**：血常规中的炎症指标升高可反映炎症，但特异性低，需结合CRP、血沉等综合判断。

4.医生的谨慎判断

- **避免误诊**：强直性脊柱炎诊断需谨慎，避免仅凭部分指标误诊。误诊可能导致不必要治疗和心理负担。

- **个体化评估**：医生会综合考虑你的年龄、病史、症状持续时间等个体因素。若某些因素不符合典型强直性脊柱炎特征，可能需进一步观察。

5.- **进一步检查**：与医生讨论进行更深入的检查，如骶髂关节和脊柱的MRI检查，以明确是否存在早期病变。

- **定期随访**：定期复诊，监测症状和检查指标的变化，以便及时发现病情进展。

- **多学科会诊**：建议到风湿免疫科或相关专科进行多学科会诊，综合评估病情，明确诊断。

二十三.

1. **疾病活动性**：如果能够有效控制炎症，可以减少关节损伤和残疾的风险。

2. **早期诊断和治疗**：早期开始治疗有助于减缓疾病进展。

3. **治疗依从性**：严格按照医嘱进行治疗和康复训练，有助于更好地管理病情。

4. **生活方式**：保持适当的运动和健康的生活方式对控制病情和预防残疾很重要。

5. **药物治疗**：遵医嘱使用抗炎药、免疫抑制剂等药物。

6. **康复训练**：进行适当的运动，如游泳、瑜伽等，有助于保持关节灵活性和肌肉力量。

7. **姿势矫正**：保持正确的坐姿和站姿，避免长时间保持同一姿势。

8. **健康饮食**：均衡饮食，避免过度肥胖，减轻关节负担。

9. **定期复诊**：定期到医院复查，监测病情变化，及时调整治疗方案。

10.如果你对病情发展或治疗效果有疑问，建议及时咨询风湿免疫科医生，他们可以根据你的具体情况提供更专业的建议和治疗方案。

二十四.

1.疾病活动性

- **活动期**：在疾病活动期，如出现疼痛、晨僵、疲劳等症状明显时，可能需要使用非甾体抗炎药（NSAIDs）、改善病情抗风湿药（DMARDs）如柳氮磺吡啶、甲氨蝶呤等，以及生物制剂如肿瘤坏死因子-α（TNF-α）抑制剂等。这个阶段用药时间通常较长，可能需要数月甚至数年，直到症状得到控制。

- **缓解期**：当病情进入缓解期，症状明显减轻或消失时，可能逐渐减少药物剂量或延长用药间隔。但通常仍需继续用药维持治疗，以防止疾病复发。维持治疗的时间也较长，可能需要数年甚至终身。

2.治疗反应

- **个体差异**：不同患者对药物的反应存在差异。有些患者可能对某种药物反应良好，症状能迅速得到控制，用药时间可能相对较短；而另一些患者可能需要尝试多种药物或联合用药才能达到理想效果，用药时间会相应延长。

- **耐药性**：长期使用某些药物可能会出现耐药性，如生物制剂。一旦出现耐药性，可能需要更换药物或调整治疗方案，这也会使整个治疗过程中的用药时间增加。

3.病情严重程度

- **轻度**：对于病情较轻的患者，可能仅需使用NSAIDs等药物来缓解症状，用药时间可能相对较短，可能在数周至数月之间，但也要根据具体病情调整。

- **中重度**：而对于病情较重，如已出现关节结构破坏、脊柱强直变形等并发症的患者，可能需要长期甚至终身用药，同时配合其他治疗手段如物理治疗、手术等。

4.总之，AS患者需要在风湿免疫科医生的指导下进行个体化治疗，定期复诊，根据病情变化及时调整治疗方案，以达到最佳治疗效果并尽量减少药物的不良反应。

二十五.

1.药物种类与疫苗接种

- **非甾体抗炎药（NSAIDs）**：这类药物通常不影响疫苗接种，患者在使用NSAIDs期间可以正常接种疫苗。

- **改善病情抗风湿药（DMARDs）**：如柳氮磺吡啶、甲氨蝶呤等，可能会对疫苗的效果产生一定影响。例如，甲氨蝶呤可能抑制免疫系统，从而降低疫苗的免疫反应。因此，在使用这些药物期间接种疫苗，可能需要根据具体药物和剂量来评估是否适合接种，以及选择合适的疫苗类型。

- **生物制剂**：如肿瘤坏死因子-α（TNF-α）抑制剂等，对免疫系统的影响更为显著。在使用生物制剂期间，通常建议避免接种减毒活疫苗，因为可能会增加感染风险。对于灭活疫苗，也需要在医生的指导下谨慎接种。

2. 疫苗类型与接种建议

- **灭活疫苗**：如流感疫苗、肺炎球菌疫苗等，通常在AS患者中接种相对安全，但在服药期间可能会影响免疫反应的效果。如果病情稳定且药物剂量较小，可在医生建议下接种。

- **减毒活疫苗**：如麻疹疫苗、水痘疫苗等，由于其具有一定的活性成分，可能会在免疫系统受到抑制的情况下引发感染风险，因此在AS患者服药治疗期间一般不推荐接种。

3. 疾病状态与接种时机

- **疾病活动期**：当AS处于活动期，患者有明显的炎症症状时，接种疫苗可能会加重病情或增加不良反应的风险。此时应优先控制疾病活动，暂缓疫苗接种。

- **缓解期**：当病情处于缓解期，症状得到较好控制时，可根据具体药物使用情况和疫苗类型，在医生的指导下考虑接种疫苗。

4.总之，AS患者在服药治疗期间是否可以打疫苗，需要综合考虑所用药物、疫苗类型以及自身疾病状态等因素。在做出决定之前，务必咨询风湿免疫科医生或专业医疗人员，他们可以根据你的具体病情和治疗方案，提供个性化的建议和指导。

二十六.

1.抗炎药

- **定义与作用**：抗炎药是指能够抑制炎症反应的药物。它们主要通过抑制炎症介质的产生或释放，从而减轻炎症引起的红、肿、热、痛等症状。抗炎药不仅能够缓解疼痛，还能减轻炎症导致的组织损伤和功能障碍。

- **作用机制**：例如，非甾体抗炎药（NSAIDs）是一类常见的抗炎药，它们通过抑制环氧化酶（COX）酶的活性，减少前列腺素等炎症介质的合成。

- **适用症状**：适用于多种炎症性疾病，如类风湿关节炎、强直性脊柱炎等，可有效缓解关节疼痛、肿胀和晨僵等症状。

2.消炎药

- **定义与作用**：消炎药在医学上通常指的是具有消除炎症作用的药物，广义上抗炎药也可视为消炎药，但在日常生活中，人们有时会将一些外用的具有减轻炎症症状的药物称为消炎药。

- **作用机制**：比如，糖皮质激素类药物具有强大的抗炎、免疫抑制等作用，能迅速减轻炎症反应，但长期使用可能会有较多副作用。

- **适用症状**：适用于各种急慢性炎症，如皮肤炎症、眼部炎症等，可减轻局部的红肿热痛等症状。

3.止疼药

- **定义与作用**：止疼药主要是指具有镇痛作用的药物，其主要目的是缓解疼痛，但并不一定具有抗炎作用。

- **作用机制**：例如，对乙酰氨基酚是一种常用的止疼药，它通过抑制中枢神经系统中前列腺素的合成，起到解热镇痛的作用，但其抗炎作用较弱。

- **适用症状**：适用于多种疼痛症状，如头痛、牙痛、肌肉痛等，对于炎症引起的疼痛也有一定的缓解作用，但若存在明显的炎症，单纯使用止疼药可能无法控制炎症的发展。

4.在实际应用中，医生会根据病情的具体情况，选择合适的药物进行治疗。对于强直性脊柱炎患者，非甾体抗炎药常作为一线治疗药物，既能抗炎又能止痛，当病情较重或对NSAIDs疗效不佳时，可能还会联合使用其他类型的药物，如改善病情抗风湿药或生物制剂等。

二十七.

1. 药物作用机制与剂量限制

- **作用机制**：常见的止疼药如非甾体抗炎药（NSAIDs），主要是通过抑制环氧化酶（COX）酶的活性，减少前列腺素等炎症介质的合成，从而起到抗炎和镇痛的作用。但是，这类药物在一定剂量下才能发挥其最大效果，超过剂量并不一定能增强疗效，反而可能增加不良反应的发生风险。

- **剂量限制**：每种药物都有其规定的剂量范围，这是基于大量的临床试验和安全性评估确定的。例如，布洛芬的常规剂量为每次400-600mg，每日最大剂量不超过2400mg；塞来昔布的常规剂量为每次100-200mg，每日最大剂量不超过400mg。如果在规定剂量内疼痛仍未缓解，应先考虑其他因素，而不是盲目增加剂量。

2. 可能的原因及解决方法

- **药物选择不当**：不同类型的疼痛可能需要不同类型的止疼药。如果目前使用的药物对你的疼痛类型效果不佳，可以咨询医生是否需要更换其他种类的药物。例如，对于神经病理性疼痛，可能需要使用加巴喷丁、普瑞巴林等药物；而对于骨关节疼痛，可能需要联合使用氨基葡萄糖等软骨保护剂。

- **疾病活动度高**：如果疼痛是由于强直性脊柱炎病情活动引起的，仅依靠止疼药可能无法有效控制症状。此时，需要及时联系医生，评估是否需要调整治疗方案，如增加抗风湿药物的剂量或联合使用生物制剂等。

- **疼痛部位特殊**：某些部位的疼痛可能对药物的吸收和分布存在差异，导致药物在疼痛部位的浓度不足。这种情况下，可以咨询医生是否需要调整给药途径或使用局部外用药物来辅助缓解疼痛。

- **存在其他疾病或并发症**：如果同时患有其他疾病，如胃肠道疾病、肝肾功能损害等，可能会影响药物的代谢和排泄，从而降低药物的效果。此时，需要告知医生你的全部病情和用药史，以便医生综合考虑，调整药物或剂量。

3. 安全提示与就医建议

- **避免自行加量**：自行增加止疼药的剂量可能会导致药物过量，引发一系列不良反应，如胃肠道出血、肝肾功能损害、心血管事件等。因此，切勿在未经医生指导的情况下随意加量。

- **及时就医评估**：如果疼痛持续不缓解，建议及时联系你的风湿免疫科医生或疼痛科医生。医生可能会建议你进行一些检查，如血常规、炎症指标、影像学检查等，以评估病情和药物的效果，并根据结果调整治疗方案。

4.总之，面对止疼药效果不佳的情况，应先从多方面分析原因，并在医生的指导下进行合理的调整和处理，而不是盲目自行加量。

二十八.

1.- **药物代谢产物**：某些药物在体内代谢后会产生具有颜色的代谢产物，这些代谢产物通过尿液排出体外，从而使尿液颜色发生改变。例如，维生素B族药物、利福平等抗生素等可能会使尿液呈现黄色或橙色。

- **药物对肝脏的影响**：部分药物可能会对肝脏产生一定的负担或影响肝脏的代谢功能，导致胆红素代谢异常，进而使尿液中胆红素含量增加，出现尿液变黄的情况。但这种情况通常还会伴随其他肝功能异常的表现，如皮肤黄染、乏力等。

2.- **观察伴随症状**：如果尿液变黄的同时没有其他不适症状，如尿频、尿急、尿痛、腰痛、恶心、乏力等，可能是药物的正常代谢现象，不必过于担心。

- **多喝水**：增加水分摄入可以稀释尿液，有助于观察尿液颜色是否恢复正常。如果多喝水后尿液颜色仍然持续发黄，建议及时就医。

- **咨询医生**：如果对尿液颜色的变化感到担忧，或者怀疑与所服用的药物有关，应及时咨询医生或药师，他们可以根据具体药物和个体情况，判断是否需要进一步检查或调整治疗方案。

3.总之，药物可能会导致尿液变黄，但不一定是副作用。在没有其他不适症状的情况下，可以先观察并多喝水，如有疑虑及时咨询医生。

二十九.

1.- **定义**：生物制剂是利用基因工程、蛋白质工程等生物技术制造的药物，主要成分为大分子蛋白质，如单克隆抗体等。

- **作用原理**：生物制剂通过特异性靶向炎症反应中的关键因子，如肿瘤坏死因子-α（TNF-α）、白细胞介素-17（IL-17）等，来抑制炎症反应，从而减轻AS患者的症状和延缓疾病进展。

2.常见种类与适用情况

- **常见种类**：

- **抗TNF-α制剂**：如英夫利西单抗、阿达木单抗等，是目前应用最广泛的生物制剂。它们通过与TNF-α结合，阻止其与细胞表面的受体结合，从而抑制炎症反应。

- **抗IL-17制剂**：如司库奇尤单抗等，通过抑制IL-17的活性，减少炎症细胞的浸润和炎症因子的释放。

- **抗CD20制剂**：如利妥昔单抗，主要针对B细胞表面的CD20抗原，用于某些特定情况下的AS治疗。

- **适用情况**：生物制剂通常适用于对传统药物（如非甾体抗炎药、柳氮磺吡啶等）疗效不佳或不耐受的中重度AS患者，以及存在关节外表现或预后不良因素的患者。

3.优势与风险

- **优势**：

- **高特异性**：生物制剂能够精准地作用于炎症反应中的特定靶点，相比传统药物具有更高的特异性和针对性，从而在有效控制病情的同时，减少对正常生理功能的影响。

- **显著疗效**：在缓解AS患者的疼痛、晨僵、关节肿胀等症状，改善脊柱和关节的功能，以及延缓影像学进展等方面，生物制剂往往能够取得较为显著的疗效，尤其对于那些病情较为严重的患者。

- **风险**：

- **感染风险增加**：生物制剂可能会抑制免疫系统的正常功能，使患者感染的风险有所增加，如呼吸道感染、皮肤感染等。在使用过程中需要密切监测患者的感染症状，一旦出现感染迹象应及时处理。

- **其他不良反应**：部分患者可能会出现注射部位反应（如红肿、疼痛等）、过敏反应等不良反应。此外，长期使用生物制剂还可能存在一些潜在的安全性问题，如恶性肿瘤风险的增加等，但总体发生率相对较低。

4.使用注意事项

- **严格筛选患者**：在使用生物制剂之前，需要对患者进行全面的评估，包括病情严重程度、既往治疗史、合并症、感染史等，以确定是否适合使用生物制剂以及选择合适的种类。

- **规范治疗与监测**：治疗过程中，患者应严格按照医嘱用药，定期进行随访和监测，包括血常规、肝肾功能、炎症指标等检查，以便及时发现和处理可能出现的不良反应，并根据病情变化调整治疗方案。

- **预防接种**：由于生物制剂可能增加感染风险，在使用前应确保患者已完成必要的疫苗接种，如流感疫苗、肺炎球菌疫苗等，以降低感染发生率。

5.总之，生物制剂为AS的治疗提供了新的选择和希望，尤其在提高疗效和改善患者生活质量方面具有独特的优势。然而，其应用也需要在专业医生的指导下，充分权衡利弊，确保安全有效地使用。

三十.

1.常见副作用

- **感染风险增加**：这是生物制剂治疗中最受关注的副作用之一。由于生物制剂通过抑制免疫系统中的特定靶点来发挥作用，可能会使机体对细菌、病毒等病原体的抵抗力有所下降，从而增加感染的风险。常见的感染包括上呼吸道感染、泌尿系统感染、皮肤感染等。在使用生物制剂期间，如果出现发热、咳嗽、尿频、尿急等症状，应及时告知医生并进行相应检查。

- **注射部位反应**：部分患者在使用生物制剂时，可能会出现注射部位的局部反应，如红肿、瘙痒、疼痛等。这些反应通常较为轻微，可在数天内自行缓解。如果局部反应较为严重或持续时间较长，应咨询医生是否需要采取相应的处理措施，如局部冷敷、使用抗过敏药膏等。

- **过敏反应**：虽然相对较少见，但生物制剂也可能会引发过敏反应，表现为皮疹、荨麻疹、呼吸困难等。如果在用药过程中出现过敏症状，应立即停药并寻求医疗帮助。

2.相对严重的副作用

- **结核复发或激活**：对于有结核病史或潜伏性结核感染的患者，使用生物制剂可能会增加结核复发或激活的风险。因此，在开始生物制剂治疗前，通常需要进行结核筛查，如PPD试验、胸部X光等。如果筛查结果提示存在结核感染风险，需先进行抗结核治疗，待结核治愈后再考虑使用生物制剂。

- **神经系统疾病**：罕见情况下，某些生物制剂可能会增加多发性硬化等神经系统疾病的风险，尤其是针对TNF-α的生物制剂。如果患者在用药过程中出现视力模糊、肢体麻木、共济失调等神经系统症状，应立即就医进行检查。

- **恶性肿瘤风险**：长期使用生物制剂是否增加恶性肿瘤的风险一直是研究的热点问题。目前的研究结果显示，虽然总体恶性肿瘤的发生率并未显著增加，但在某些特定类型的肿瘤，如皮肤癌等，可能会有一定程度的风险增加。因此，在使用生物制剂期间，应注意定期体检，以便早期发现和处理潜在的肿瘤病变。

3.副作用的个体差异与预防

- **个体差异**：不同患者使用生物制剂时出现副作用的情况可能存在较大差异。一些患者可能在整个治疗过程中几乎没有明显的副作用，而另一些患者则可能出现多种不良反应。这可能与患者的个体免疫系统、基础健康状况、合并用药等因素有关。

- **预防措施**：为了降低生物制剂副作用的发生风险，在治疗前应进行全面的健康评估，包括详细病史采集、体格检查和必要的实验室检查。在治疗过程中，患者应严格遵循医嘱，定期进行随访和监测，以便及时发现和处理可能出现的副作用。同时，保持良好的生活习惯，如合理饮食、适量运动、避免吸烟和过度饮酒等，也有助于增强机体免疫力，减少副作用的发生。

4.总之，生物制剂在AS治疗中虽然存在一定的副作用，但大多数副作用是可控的。在专业医生的指导下，权衡利弊，合理使用生物制剂，可以最大限度地发挥其治疗效果，同时降低副作用对患者健康的影响。

三十一.

1.生制剂不会成瘾。

2.生物制剂是一种通过生物技术制造的药物，它在强直性脊柱炎（AS）的治疗中主要通过靶向作用于炎症反应中的特定因子来发挥作用。使用生物制剂治疗AS是为了控制病情活动、缓解症状和延缓疾病进展，需要根据患者的病情和治疗反应来决定使用时间和剂量。

3.如果患者在使用生物制剂期间病情得到了有效控制，症状缓解，炎症指标恢复正常，在医生的评估下可以考虑减量或停药。但是，减量或停药后病情可能会出现反复，这并非是因为生物制剂具有成瘾性，而是因为疾病本身具有慢性、易复发的特点。

4.在治疗过程中，医生会根据患者的病情变化、治疗反应以及安全性等因素综合考虑是否继续使用生物制剂。患者应严格遵循医嘱，定期复诊，以便医生及时调整治疗方案，避免自行随意停药或减量，以确保病情的稳定控制。

三十二.

1.传统药物治疗

- **非甾体抗炎药（NSAIDs）**：如布洛芬、塞来昔布等，仍然是控制强直性脊柱炎症状的一线药物。它们可以有效缓解疼痛和炎症，但长期使用可能会有胃肠道和心血管方面的副作用。

- **慢作用抗风湿药（DMARDs）**：如柳氮磺吡啶、甲氨蝶呤等，这些药物虽然起效较慢，但可以在一定程度上控制病情的进展。

2.物理治疗与康复训练

- **物理治疗**：包括热敷、按摩、超声波治疗等，有助于缓解肌肉紧张和关节僵硬，改善局部血液循环，减轻疼痛。

- **康复训练**：进行适当的康复训练，如游泳、瑜伽、脊柱伸展运动等，可以增强肌肉力量，保持关节的灵活性和活动范围，改善姿势。

3.定期监测与调整治疗方案

- **定期复查**：在治疗过程中，需要定期进行血常规、肝肾功能、炎症指标等检查，以评估病情和药物的副作用。

- **调整治疗方案**：如果在治疗过程中感染得到控制，可以与医生讨论是否可以重新考虑使用生物制剂或其他更有效的治疗方案。

4.总之，在存在其他感染的情况下，需要在医生的指导下，根据具体病情和感染类型，制定个体化的治疗方案，以确保安全有效地控制强直性脊柱炎的病情。

三十三.

1. 生物制剂与其他药物的联合使用

- **非甾体抗炎药（NSAIDs）**：即使使用了生物制剂，可能仍需要继续使用NSAIDs来缓解疼痛和炎症。因为生物制剂主要针对炎症反应中的特定靶点，起效可能需要一定时间，在这期间NSAIDs可以提供快速的对症治疗。

- **慢作用抗风湿药（DMARDs）**：如柳氮磺吡啶、甲氨蝶呤等，有时会与生物制剂联合使用，以增强疗效或减少生物制剂的使用剂量，从而降低生物制剂的副作用风险。

2. 生物制剂的使用时机与疗程

- **起始阶段**：在生物制剂治疗的初始阶段，为了快速控制病情，可能需要联合使用其他药物。随着生物制剂疗效的逐渐显现，医生可能会根据病情调整药物方案，减少或停用部分其他药物。

- **维持阶段**：当病情得到稳定控制后，可能仅需继续使用生物制剂进行维持治疗，但这也需要根据个体情况而定。有些患者可能在生物制剂维持治疗的基础上，仍需小剂量使用其他药物来保持病情的稳定。

3. 个体化治疗的重要性

每个患者的具体病情、身体状况、对药物的反应等都不同。因此，在使用生物制剂时是否需要同时吃其他药物，必须由医生根据患者的具体情况来决定。医生会综合考虑病情的严重程度、治疗反应、药物的副作用等因素，制定最合适的治疗方案。

4.总之，强直性脊柱炎的治疗是一个个体化的过程，生物制剂的使用可能会伴随其他药物的联合应用，以达到最佳的治疗效果。患者应严格遵循医生的指导，定期复诊，以便医生及时调整治疗方案。

三十四.

1.疾病缓解后的考虑

- **病情评估**：如果患者在使用生物制剂后病情得到了显著缓解，达到了临床缓解或低疾病活动度状态，并且维持了一段时间（通常为3个月以上），可以考虑逐渐减少生物制剂的剂量或延长用药间隔。

- **个体化决策**：是否停药需要根据患者的具体病情、病史、治疗反应等因素进行个体化决策。医生会综合评估患者的症状、体征、实验室检查结果以及影像学表现等，来判断是否适合停药。

2.停药后的监测与调整治疗方案

- **密切监测**：在停用生物制剂后，需要对患者进行密切的监测，包括定期的随访和必要的检查，如血沉、C反应蛋白、关节超声等，以及时发现病情的变化。

- **调整治疗方案**：如果停药后病情出现反复或加重，可能需要重新开始使用生物制剂或其他有效的治疗药物。医生会根据病情重新评估治疗方案，并做出相应的调整。

3.总之，强直性脊柱炎患者在使用生物制剂后是否能够停药，需要在医生的指导下，根据个体病情进行谨慎的评估和决策。患者应严格遵循医嘱，定期复诊，以便及时调整治疗方案，确保病情的稳定控制。

三十五、

1. 激素，即糖皮质激素，具有强大的抗炎和免疫抑制作用，能迅速缓解强直性脊柱炎的炎症反应和免疫异常，如减轻疼痛、晨僵，降低炎症指标。但长期使用可能引发骨质疏松、血糖升高、消化道溃疡等副作用，还可能抑制下丘脑-垂体-肾上腺轴功能。所以激素不是治疗强直性脊柱炎的首选药物。
2. 仅在特定情况下短期使用，如病情急性加重或出现严重关节外表现时，使用时需严格遵循医嘱，定期监测，以减少副作用风险。

三十六.

1.非甾体抗炎药（NSAIDs）

- **小手术**：如拔牙等小型口腔手术或皮肤手术等，如果患者正在使用的NSAIDs药物对凝血功能影响较小，且手术医生评估后认为可以接受，可能在手术前短暂停用几天后即可进行手术；但如果药物对凝血功能影响较大，则可能需要提前1周左右停用。

- **大手术**：对于脊柱或关节等较大创伤的手术，通常需要提前1-2周停用NSAIDs，以降低手术中出血风险和术后血栓形成风险。

2.慢作用抗风湿药（DMARDs）

- **甲氨蝶呤**：手术前通常需要停用2-4周，因为它可能会增加手术后的感染风险和影响伤口愈合。

- **柳氮磺吡啶**：对于肠道手术，可能需要提前2-3周停用，因为它在肠道内代谢，可能对肠道黏膜有一定刺激性，影响肠道手术后的恢复；但对于非肠道手术，可能在手术前1周左右停用即可。

- **来氟米特**：由于其在体内的半衰期较长，手术前可能需要提前3-4周停用，并且可能需要使用考来烯胺等药物进行药物清除，以降低其在体内的浓度和潜在的不良影响。

3.生物制剂

- **抗TNF-α制剂**：如英夫利西单抗、阿达木单抗等，手术前通常需要停用2-3个月。因为这类生物制剂会抑制免疫系统，增加感染风险，尤其是在手术创口愈合过程中可能会影响组织修复和抗感染能力。

- **其他生物制剂**：如抗IL-17制剂等，根据具体药物的特性和手术类型，停用时间可能有所不同，一般也需要提前数周至数月停用。

4.糖皮质激素

- **小剂量短期使用**：如果患者因病情需要在术前短期小剂量使用糖皮质激素，如泼尼松等，对于一些小型手术，在手术当天或前一天停用即可；但对于较大手术，可能需要提前3-5天逐渐减量停用，以避免突然停药引起的肾上腺皮质功能不全等撤药反应。

- **长期大剂量使用**：长期大剂量使用糖皮质激素的患者，手术前需要根据患者的具体病情和身体状况，在风湿免疫科医生和手术医生的共同指导下，制定个体化的减量和停药方案，可能需要提前数周甚至数月逐步减少剂量，同时密切监测患者的肾上腺皮质功能等指标，以确保手术安全。

5.在准备手术期间，患者应与手术医生和风湿免疫科医生保持密切沟通，根据具体的手术类型、药物种类以及自身的病情，制定合理的药物调整方案。同时，在手术后，医生会根据患者恢复情况和病情需要，决定何时重新开始使用相关药物来控制强直性脊柱炎的病情。

三十七.

1.非甾体抗炎药（NSAIDs）与感冒药

- **相同作用机制成分**：如果感冒药中也含有解热镇痛成分，如对乙酰氨基酚等，与NSAIDs同时使用可能会增加胃肠道的不良反应，如胃肠道溃疡、出血等风险。因此，建议在使用NSAIDs期间，如果需要服用感冒药，应选择不含有相同作用机制成分的感冒药，或在医生的指导下调整用药方案。

- **不同作用机制成分**：如果感冒药中的成分与NSAIDs的作用机制不同，如主要含有抗组胺成分用于缓解流涕、打喷嚏等症状，在医生的指导下可以谨慎同时使用，但仍需密切观察是否有不良反应的发生。

2.慢作用抗风湿药（DMARDs）与感冒药

- **柳氮磺吡啶、甲氨蝶呤等**：这些药物本身可能对骨髓造血功能有一定的抑制作用，影响白细胞、红细胞和血小板的生成。如果与某些感冒药中的成分同时使用，可能会进一步抑制骨髓功能，导致血细胞减少等不良反应加重。因此，在使用DMARDs治疗强直性脊柱炎期间，服用感冒药时需要特别谨慎，最好在医生的指导下选择对骨髓抑制作用较小的感冒药，并定期监测血常规等指标。

- **来氟米特等**：来氟米特在体内的代谢时间较长，与其他药物的相互作用较为复杂。某些感冒药中的成分可能会影响其代谢过程，增加不良反应的发生风险。所以在使用来氟米特时，应避免同时服用可能影响其代谢的感冒药，或在医生的指导下调整用药剂量和时间间隔。

3.生物制剂与感冒药

- **抗TNF-α制剂等**：生物制剂通过调节免疫系统来发挥作用，可能会增加感染的风险。如果在使用生物制剂期间同时服用某些可能影响免疫功能的感冒药，可能会进一步增加感染的发生概率或加重感染的严重程度。因此，在使用生物制剂治疗强直性脊柱炎时，如果需要服用感冒药，应选择对免疫系统影响较小的药物，并在医生的指导下严格控制用药剂量和时间，同时密切观察是否有感染症状的出现。

4.在治疗强直性脊柱炎期间，如果需要服用感冒药，建议先咨询医生或药师，告知他们目前正在使用的所有药物，包括治疗强直性脊柱炎的药物，以便他们能够根据具体情况给出合理的建议和指导，确保用药的安全性和有效性。

三十八.

1.疾病活动度与怀孕时机

- **病情稳定期**：如果病情处于稳定期，症状较轻，如晨僵、疼痛等症状不明显，炎症指标基本正常，在这种情况下怀孕，风险相对较低，胎儿的发育和健康通常不会受到明显影响。

- **疾病活动期**：如果病情处于活动期，有明显的关节疼痛、肿胀、晨僵，以及发热等全身症状，建议暂缓怀孕。因为此时怀孕可能会增加孕期并发症的风险，如流产、早产、胎儿生长受限等，同时疾病活动也可能对母亲的健康造成更大的负担。

2.药物调整与怀孕

- **停药或换药**：在备孕前，需要与风湿免疫科医生和妇产科医生共同讨论目前所用药物对怀孕的影响。一些药物如甲氨蝶呤等，在怀孕前需要提前停用足够长的时间，以确保药物在体内的浓度降低到安全水平。

- **使用安全药物**：在怀孕期间，如果病情需要继续用药控制，医生会选择对胎儿相对安全的药物。例如，某些非甾体抗炎药在怀孕早期可能需要慎用或避免长期大剂量使用，而在怀孕中后期可能在医生指导下短期、小剂量使用来缓解症状。

3.怀孕过程中的监测与管理

- **定期产检**：怀孕期间，需要按照妇产科医生的建议进行定期产检，密切监测胎儿的发育情况，包括超声检查、唐氏筛查等必要检查项目。

- **风湿科随访**：同时，也要定期到风湿免疫科随访，评估强直性脊柱炎的病情变化，根据需要调整治疗方案，以平衡疾病控制和胎儿安全之间的关系。

4.生活方式与怀孕

- **健康饮食**：保证营养均衡，摄入足够的蛋白质、维生素和矿物质等营养物质，有助于胎儿的健康发育，同时也有利于维持自身免疫系统的稳定。

- **适量运动**：在怀孕期间，进行适量的运动，如散步、孕妇瑜伽等，可以增强体质，缓解孕期不适，但要避免过度劳累和剧烈运动。

5.总之，得了强直性脊柱炎的女性患者在病情稳定、药物调整合理、孕期密切监测的情况下，是可以成功怀孕并生育健康宝宝的。但在整个过程中需要多科室协作和个体化管理，以确保母婴安全。

三十九.

1.与医生沟通

- **制定个体化方案**：在备孕前，一定要与风湿免疫科医生和妇产科医生进行充分的沟通，告知他们你的病情、目前使用的药物以及备孕的计划。医生会根据你的具体情况，综合考虑病情控制和药物对胎儿的潜在影响，制定个体化的药物调整方案。

- **定期复查与评估**：在备孕期间，要按照医生的建议定期进行复查，包括血常规、炎症指标、肝肾功能等检查，以便及时了解病情变化和身体状况，医生也可以根据复查结果及时调整治疗方案。

2.药物调整原则

- **停用高风险药物**：一些药物在备孕期间需要停用，如甲氨蝶呤等，因为它们可能对胎儿造成严重的不良影响，包括致畸等。通常需要在备孕前3-6个月停用此类药物，以确保药物在体内代谢清除，降低对胎儿的风险。

- **换用相对安全药物**：对于病情需要继续控制的情况，医生可能会选择在备孕期间相对安全的药物进行替代治疗。例如，某些非甾体抗炎药在医生指导下可以短期、小剂量使用来缓解症状，但也要根据具体药物和病情进行权衡。

- **逐渐减量过程**：如果病情相对稳定，医生可能会建议在备孕期间逐渐减少药物剂量，以尽量减少药物对胎儿的暴露，同时保持病情的稳定。减量过程需要在医生的严格指导下进行，避免自行突然停药或快速减量导致病情加重。

3.生活方式与辅助措施

- **健康饮食**：保证营养均衡，摄入富含蛋白质、维生素和矿物质的食物，如瘦肉、鱼类、新鲜蔬菜和水果等，有助于维持良好的身体状态和免疫功能，为备孕创造良好的内部环境。

- **适量运动**：进行适量的运动，如散步、瑜伽等，可以增强体质，提高身体的免疫力和抵抗力，同时也有助于缓解强直性脊柱炎的症状，保持关节的灵活性和肌肉力量。

- **避免不良因素**：远离吸烟、饮酒等不良生活习惯，避免接触有害化学物质和辐射等可能影响胎儿健康的因素，创造一个健康的生活环境。

4.总之，强直性脊柱炎患者在备孕期间需要在医生的指导下，根据自身病情和药物特点，合理调整用药方案，同时结合健康的生活方式，以确保母婴安全，提高怀孕的成功率和胎儿的健康水平。

四十.

1.中药的作用及局限性

- **缓解症状**：中药可以通过整体调理，改善患者的体质和免疫功能，从而在一定程度上缓解疼痛、疲劳等症状，提高生活质量。

- **调节免疫**：一些中药具有调节免疫功能的作用，可能有助于减轻强直性脊柱炎的免疫异常反应。

- **局限性**：中药的治疗效果相对缓慢，且缺乏足够的临床研究证据来证明其对强直性脊柱炎的根治或长期控制效果。

2.针灸的作用及局限性

- **缓解疼痛**：针灸通过刺激特定的穴位，调节经络气血的运行，起到通经止痛的作用，能够有效缓解强直性脊柱炎患者的关节疼痛、肌肉酸痛等症状。

- **改善功能**：针灸还可以促进局部血液循环，放松肌肉，增加关节的活动度，有助于改善患者的脊柱和关节功能，提高患者的活动能力和生活质量。

- **局限性**：针灸治疗通常需要较长时间的坚持才能见效，且其效果可能因个体差异而异。

3.西药的重要性

- **快速控制病情**：西药如非甾体抗炎药、慢作用抗风湿药和生物制剂等，能够快速有效地控制炎症反应，缓解症状，防止关节结构的进一步破坏。

- **长期维持治疗**：对于中重度强直性脊柱炎患者，西药在维持病情稳定、防止疾病进展方面具有重要作用，尤其是生物制剂，对许多患者有显著疗效。

4.综合治疗的建议

- **联合应用**：在治疗强直性脊柱炎时，可以将中药和针灸作为辅助治疗方法，与西药联合使用，以发挥各自的优势，提高治疗效果。

- **个体化治疗**：每个患者的具体情况不同，治疗方案应根据患者的病情、身体状况、对药物的反应等因素进行个体化制定。

- **专业指导**：在使用中药或针灸治疗时，应寻求专业的中医师或针灸师的指导，确保治疗的安全性和有效性。

5.总之，中药和针灸可以在一定程度上辅助治疗强直性脊柱炎，缓解症状、提高生活质量，但不能完全代替西药的治疗。患者应根据自身情况，在专业医生的指导下，合理选择和使用中西医结合的治疗方法，以达到最佳的治疗效果。

四十一.

1.锻炼的重要性

- **维持关节活动度**：强直性脊柱炎会导致脊柱和关节的僵硬和疼痛，适当的锻炼可以帮助维持关节的活动度，防止关节强直和畸形的发生。例如，进行腰部的屈伸、旋转等运动，可以保持腰椎的灵活性。

- **增强肌肉力量**：锻炼可以增强腰部和背部的肌肉力量，肌肉的强大可以更好地支撑脊柱，减轻腰痛的症状，并提高患者的日常活动能力。

- **改善姿势**：强直性脊柱炎患者容易出现驼背等不良姿势，通过锻炼可以纠正姿势，减轻因姿势不良导致的腰痛。

2.适合的锻炼方式

- **伸展运动**：如腰部的前屈、后伸、侧弯和旋转等动作，可以帮助缓解腰痛，增加腰部的柔韧性。

- **游泳**：是一种对关节冲击较小的有氧运动，可以锻炼全身的肌肉，特别是背部和腰部的肌肉，同时在水中运动还能减轻身体的重量对脊柱的压力。

- **瑜伽**：一些简单的瑜伽姿势和呼吸练习，有助于改善身体的柔韧性、平衡性和放松身心，缓解腰痛带来的不适。

- **散步**：作为一种低强度的有氧运动，散步可以促进血液循环，增强心肺功能，同时对腰椎和下肢关节的压力较小，适合大多数强直性脊柱炎患者。

3.注意事项

- **避免过度锻炼**：在腰痛严重时，应避免进行剧烈的运动或长时间的锻炼，以免加重疼痛和炎症。可以适当减少锻炼的强度和时间，等疼痛缓解后再逐渐恢复。

- **正确姿势**：锻炼时要注意保持正确的姿势，避免因姿势不当导致腰部受伤或疼痛加重。

- **结合热敷或按摩**：在锻炼前后，可以结合热敷或按摩等物理治疗方法，缓解腰部的肌肉紧张和疼痛，提高锻炼的效果和舒适度。

4.总之，强直性脊柱炎患者在腰痛的情况下，应根据自身的病情和身体状况，选择适合自己的锻炼方式，并在锻炼过程中注意保护腰部，避免加重疼痛。如有疑问或疼痛持续不缓解，应及时咨询医生或物理治疗师的建议。

四十二、

1. 关节严重破坏

- **关节置换术**：当强直性脊柱炎累及髋关节等大关节，导致关节严重破坏、疼痛难忍且功能丧失时，关节置换术是一种有效的治疗方法。例如，全髋关节置换可以显著改善患者的疼痛症状，恢复关节的活动能力，提高生活质量。

2. 脊柱畸形

- **脊柱矫正术**：在强直性脊柱炎的晚期，部分患者可能会出现脊柱的严重畸形，如驼背畸形等，导致患者姿势异常、平衡能力下降，甚至影响心肺功能和日常生活。脊柱矫正术旨在纠正脊柱的畸形，改善患者的姿势和平衡能力，缓解相关症状。

3. 手术风险与术后康复

- **手术风险**：手术治疗强直性脊柱炎存在一定的风险，包括感染、出血、神经损伤等。此外，术后还可能出现假体松动、脱位等并发症，需要再次手术处理。

- **术后康复**：术后康复对于手术效果至关重要。患者需要在医生和康复师的指导下进行系统的康复训练，以恢复关节功能和肌肉力量。康复训练通常包括早期的关节活动度练习、肌肉力量训练、平衡协调训练等，逐步提高患者的日常活动能力和生活质量。

4. 综合治疗的重要性

- **药物治疗**：在手术前后，通常仍需要继续使用药物控制病情，如非甾体抗炎药、慢作用抗风湿药等，以减少炎症反应，预防病情进一步发展。

- **物理治疗与康复训练**：除了手术和药物治疗外，物理治疗和康复训练也是强直性脊柱炎综合治疗的重要组成部分。物理治疗如热敷、按摩、超声波治疗等可以缓解肌肉紧张和关节僵硬；康复训练则有助于恢复关节功能和提高身体素质。

5.总之，强直性脊柱炎患者是否需要手术，需要根据具体的病情和并发症情况来决定。手术治疗只是强直性脊柱炎综合治疗的一部分，术后仍需要结合药物、物理治疗和康复训练等措施，以达到最佳的治疗效果和提高患者的生活质量。

### Wenxin

### 1.

### ****关键建议****

1. **优先就医**：
   1. **风湿免疫科就诊**：完善骶髂关节MRI（敏感检测早期炎症）、HLA-B27、血沉（ESR）、C反应蛋白（CRP）检测。
   2. **鉴别诊断**：排除感染、肿瘤或其他脊柱疾病。
2. **自我管理**：
   1. **适度运动**：游泳、瑜伽等保持脊柱灵活性，避免久坐。
   2. **物理疗法**：热敷、按摩缓解肌肉痉挛。
   3. **谨慎用药**：短期使用非甾体抗炎药（如布洛芬）需遵医嘱，避免胃肠道副作用。
3. **心理支持**：
   1. 慢性疼痛可能引发焦虑或抑郁，必要时寻求心理干预。

### **4.重要提醒**

- **避免自我诊断**：腰背疼痛病因复杂，需结合临床检查确诊。
- **早期干预价值**：若最终确诊为AS，早期使用生物制剂（如TNF抑制剂）可显著改善预后。

请及时就医并告知医生您的症状模式及家族病史，以便制定针对性检查方案。确诊前的任何猜测仅供参考，专业诊断才是关键。

2.

### ****关键行动建议****

1. **立即就医**：
   1. **风湿免疫科**：完善骶髂关节MRI（敏感检测骨髓水肿）、HLA-B27、ESR/CRP。
   2. **鉴别诊断**：通过查体排除感染或机械性腰痛（如直腿抬高试验）。
2. **自我记录**：
   1. 记录疼痛时间（如凌晨痛醒）、晨僵时长、活动后缓解时间，帮助医生判断。
   2. 观察有无虹膜炎、腹泻、皮疹等伴随症状。
3. **谨慎用药**：
   1. 短期使用NSAIDs（如塞来昔布）缓解症状，但需监测胃肠道副作用。
   2. 避免长期使用激素或盲目理疗，以免延误诊断。
4. **心理与生活方式**：
   1. 保持规律作息，避免睡前过度活动或不良姿势。
   2. 若疼痛影响睡眠，可尝试侧卧蜷腿姿势减轻压力。

### **5.重要提醒**

- **切勿拖延**：早期AS通过生物制剂（如依那西普）可阻断骨破坏，但需在确诊后使用。
- **警惕误诊**：部分机械性腰痛可能被误判为AS，需结合影像学和炎症指标综合判断。

建议尽快就诊，您的症状模式对AS具有高度提示性，但确诊仍需依赖客观检查。及时干预可显著改善预后。

3.

### ****关键行动建议****

1. **立即就医**：
   1. **风湿免疫科**：完善骶髂关节MRI（检测骨髓水肿）、HLA-B27、ESR/CRP。
   2. **鉴别诊断**：通过查体排除感染或机械性腰痛（如Schober试验评估脊柱活动度）。
2. **自我记录**：
   1. 记录晨僵持续时间、活动后缓解时间、夜间痛醒频率，帮助医生评估病情。
   2. 观察有无虹膜炎、腹泻、皮疹等伴随症状。
3. **谨慎用药**：
   1. 短期使用NSAIDs（如双氯芬酸钠）缓解症状，但需监测胃肠道/心血管副作用。
   2. 避免激素或免疫抑制剂（可能加重病情或增加感染风险）。
4. **心理与生活方式**：
   1. 保持规律作息，避免睡前过度活动或不良姿势（如蜷缩睡姿可能加重僵硬）。
   2. 晨起前可尝试热敷或温水浴缓解症状。

### ****5.重要提醒****

- **警惕并发症**：长期未控制的AS可能导致脊柱强直、骨质疏松或髋关节受累，需早期干预。
- **生物制剂价值**：若确诊AS，TNF抑制剂（如阿达木单抗）可显著改善晨僵和炎症，但需排除感染/肿瘤后使用。

建议尽快就诊，您的症状模式高度提示AS，但确诊需依赖影像学和炎症指标。及时干预可延缓病情进展，改善生活质量。

4.

### ****关键行动建议****

1. **优先就诊风湿免疫科**：
   1. 完善**骶髂关节MRI**（检测骨髓水肿）、**HLA-B27**、**ESR/CRP**。
   2. 告知医生眼部及肠道症状，评估是否需要眼科检查（裂隙灯）或肠镜检查。
2. **自我观察**：
   1. 记录腹泻频率、大便性状（是否带血/黏液）、眼痒是否伴红肿/分泌物。
   2. 警惕其他脊柱关节炎表现（如银屑病皮疹、指甲凹陷、尿道炎）。
3. **谨慎用药**：
   1. 短期使用NSAIDs（如美洛昔康）缓解症状，但需监测胃肠道副作用。
   2. 避免自行使用激素或免疫抑制剂，以免加重感染风险。
4. **生活方式调整**：
   1. 饮食：IBD患者需低渣、低脂饮食，避免辛辣/乳制品（若乳糖不耐受）。
   2. 眼部护理：避免揉眼，使用人工泪液缓解干眼（如确诊干燥综合征）。

### ****5.重要提醒****

- **警惕并发症**：未控制的AS可能导致脊柱强直、骨质疏松，而IBD可能引发肠梗阻或肠穿孔，需早期干预。
- **生物制剂价值**：若确诊AS合并IBD，TNF抑制剂（如英夫利昔单抗）可同时改善关节及肠道症状，但需严格筛查感染（如结核）。

建议尽快就医，您的症状模式提示可能存在脊柱关节炎的全身性表现，明确诊断后可制定更精准的治疗方案。

5.

### ****关键行动建议****

1. **立即就医**：
   1. **风湿免疫科**：完善骶髂关节MRI（检测早期炎症）、HLA-B27、ESR/CRP。
   2. **鉴别诊断**：通过血沉、C反应蛋白、血常规、影像学排除感染或肿瘤。
2. **观察警示信号**：
   1. 若出现**发热、体重下降、下肢麻木/无力、大小便障碍**，需急诊排查感染或神经压迫。
3. **早期治疗价值**：
   1. 若确诊AS，**生物制剂（如TNF抑制剂）**可显著延缓病情进展，避免脊柱强直。
   2. 即使未确诊，**NSAIDs短期使用**可缓解症状，但需监测副作用。
4. **心理支持**：
   1. 慢性疼痛易引发焦虑，可通过正念冥想、规律运动改善心理状态。
   2. 加入患者社群（如AS国际联盟），获取疾病管理经验。

### ****5.预后提示****

- **最佳情况**：若为AS，早期干预可维持正常生活和工作能力。
- **需警惕**：未控制的炎症可能导致脊柱融合、骨质疏松或关节外并发症（如虹膜炎、IBD）。

6.请尽快就诊，医生会通过查体、影像学和实验室检查明确方向。您的主动记录和沟通将极大帮助诊断，大多数腰痛患者通过规范治疗可显著改善生活质量。

6.

**行动建议：**
1.✅ **立即就诊风湿免疫科**，完善以下检查：

- 骶髂关节MRI（检测早期炎症）
- HLA-B27、ESR/CRP（评估炎症程度）
- 必要时眼科检查（裂隙灯排查虹膜炎）或肠镜（排查炎症性肠病）。

2.⚠️ **若出现以下情况，需急诊就医：**

- 发热、寒战（提示感染）
- 下肢麻木/无力、大小便障碍（警惕神经压迫）
- 体重明显下降（肿瘤风险信号）。

3.大多数腰痛可通过规范治疗控制，但明确病因是关键。您的症状模式高度提示慢性炎症性疾病，及时干预可显著改善预后。

### 7.

### ****. 关键行动建议****

1.✅ **立即就诊风湿免疫科**，完善以下检查：

- **骶髂关节MRI**：检测早期炎症（骨髓水肿是AS特征性表现）。
- **HLA-B27检测**：辅助诊断（阴性结果不能完全排除AS）。
- **炎症指标**：ESR、CRP升高提示活动性炎症。
- **必要时**：眼科检查（排查虹膜炎）、肠镜（排查炎症性肠病）。

2.⚠️ **若出现以下情况，需急诊就医**：

- 发热、寒战（提示感染）。
- 下肢麻木/无力、大小便障碍（警惕神经压迫）。
- 体重明显下降（肿瘤风险信号）。

### ****3. 预后与管理****

- **早期治疗价值**：若确诊AS，**生物制剂（如TNF抑制剂）**可显著延缓病情进展，避免脊柱强直。
- **生活方式调整**：保持规律运动（如游泳、瑜伽）、戒烟、避免长时间保持同一姿势。
- **心理支持**：慢性疼痛易引发焦虑，可通过正念冥想或心理咨询缓解。

**4.**您的家族史增加了AS风险，但需结合症状及检查确诊。即使未患AS，持续背痛也需明确病因，及时治疗。建议尽快就医，大多数患者通过规范治疗可控制病情，维持正常生活。

8.**强直性脊柱炎（AS）的背痛与其他腰背痛的核心区别如下：**

### ****1. 疼痛特点****

| **AS的背痛** | **其他腰背痛（如肌肉劳损、椎间盘突出）** |
| --- | --- |
| **夜间痛醒**，翻身困难 | 疼痛多在活动后加重，休息后缓解 |
| **晨僵＞30分钟**，活动后减轻 | 晨僵时间短（＜30分钟）或无晨僵 |
| 休息后加重（如久坐后疼痛加剧） | 休息后缓解 |
| 疼痛部位集中在骶髂关节、腰椎 | 疼痛可能局限在特定部位（如腰椎或肌肉） |

### ****2. 伴随症状****

**AS**：
✅ 外周关节炎（髋/膝/踝肿痛）
✅ 反复虹膜炎（眼红、畏光）
✅ 炎症性肠病（腹泻、血便）
✅ 银屑病/指甲凹陷
✅ 家族聚集性（尤其一级亲属患病）

**其他腰痛**：
❌ 通常无全身表现，可能伴随肌肉痉挛或神经放射痛（如坐骨神经痛）。

### ****3. 危险因素****

**AS**：
🔹 年轻男性高发（20-30岁）
🔹 HLA-B27基因阳性（非绝对）
🔹 感染后诱发（如腹泻、尿道炎后发病，提示反应性关节炎）

**其他腰痛**：
🔹 长期久坐、弯腰劳动史（肌肉劳损）
🔹 外伤或运动损伤史（椎间盘突出）

### ****4. 检查差异****

| **AS** | **其他腰背痛** |
| --- | --- |
| 骶髂关节MRI显示骨髓水肿 | X线/CT可见椎间盘突出或骨质增生 |
| HLA-B27阳性（约90%患者） | HLA-B27通常阴性 |
| ESR/CRP升高（提示炎症） | 炎症指标通常正常 |

### ****5. 预后与治疗****

**AS**：
💊 需长期抗炎治疗（NSAIDs、生物制剂）
💡 延误治疗可能导致脊柱强直或关节外并发症

**其他腰痛**：
💊 短期止痛药+物理治疗即可缓解
💡 多数无严重后遗症

9.

### ****1. AS的全身炎症特性****

**附着点炎**：AS属于脊柱关节炎谱系疾病，炎症不仅累及脊柱，还可侵犯肌腱、韧带在骨骼的附着点（如胸肋关节、胸骨柄）。
➜ **胸口痛机制**：胸肋关节炎症（尤其是第2-5肋软骨与胸骨连接处）可导致深呼吸、咳嗽或体位变动时疼痛加重。

**前胸壁炎症**：胸肋关节受累时，局部压痛明显，可能伴随肿胀或皮肤温度升高。

### ****2. 胸廓活动受限****

- AS晚期患者因脊柱强直和胸肋关节融合，胸廓扩张度下降，呼吸时可能诱发疼痛。

### ****3. 心脏并发症（罕见但需警惕）****

- **主动脉炎/心包炎**：AS患者炎症可能累及心血管系统，导致主动脉根部炎或心包炎，表现为胸痛（与呼吸无关，可能放射至肩背）。

### ****4. 鉴别诊断逻辑****

医生可能基于以下线索综合判断：

- **年龄与性别**：年轻患者（20-40岁）出现不明原因胸痛，尤其男性。
- **伴随症状**：腰痛（炎性特点）、晨僵、外周关节炎、眼炎/肠炎病史。
- **查体发现**：胸廓扩张度下降、胸肋关节压痛、Schober试验阳性（腰椎活动度受限）。

### ****5. 需排除的其他疾病****

- **心血管急症**：心绞痛、心梗、主动脉夹层（需心电图、心肌酶、主动脉CT排查）。
- **肺栓塞**：突发胸痛伴呼吸困难（D-二聚体、CT肺动脉造影）。
- **胃肠道疾病**：胃食管反流、食管痉挛（胃镜或钡餐检查）。
- **肌肉骨骼痛**：肋软骨炎、胸壁肌肉拉伤（通常无全身炎症表现）。

### ****6. 关键检查建议****

- **炎症指标**：ESR、CRP升高提示活动性炎症。
- **骶髂关节MRI**：检测脊柱炎症。
- **胸部CT/MRI**：观察胸肋关节及胸骨炎症。
- **心脏超声/心电图**：排查心包炎或主动脉病变。

### ****7. 治疗与管理****

- **抗炎治疗**：NSAIDs（如塞来昔布）或生物制剂（TNF抑制剂）可缓解附着点炎。
- **物理治疗**：改善胸廓活动度，减少疼痛。
- **定期监测**：警惕心血管并发症（尤其病程＞10年患者）。

10.

### ****1. 需警惕的叠加情况****

**纤维肌痛**：
🔹 心理压力是重要诱因，表现为全身广泛疼痛（含腰痛），伴疲劳、睡眠紊乱。

**肠脑轴紊乱**：
🔹 压力通过肠道菌群失调影响炎症水平，可能间接加重腰痛。

2.✅ **医学评估优先**：
🔹 排除器质性疾病（如椎间盘突出、感染、肿瘤）后再考虑心理因素。

3.✅ **身心同治策略**：

- **运动疗法**：瑜伽、普拉提改善肌肉柔韧性与力量（推荐每周3次，每次30分钟）。
- **放松训练**：深呼吸、渐进式肌肉放松降低交感兴奋。
- **认知行为疗法（CBT）**：纠正疼痛灾难化思维，打破“压力-疼痛”循环。
- **药物治疗**：短期使用NSAIDs（如布洛芬）缓解疼痛，必要时联用抗抑郁药（需精神科指导）。

4.✅ **生活方式调整**：
🔹 睡眠管理：保证7-9小时优质睡眠，必要时短期使用褪黑素。
🔹 时间管理：分解压力源，通过清单法减少焦虑。

### ****5. 何时需紧急就医****

若出现以下情况，需优先排查严重疾病：
⚠️ 夜间痛醒、发热、体重下降→警惕感染或肿瘤。
⚠️ 下肢麻木/无力、大小便障碍→警惕神经压迫。
⚠️ 胸痛、呼吸困难→排查心肺急症。

11.

**1.遗传关联性**
HLA-B27是特定基因亚型，阳性表明您携带该基因。全球约8-10%人群携带，但存在种族差异（如北欧人群阳性率更高）。

**2.疾病风险提示**
阳性者患强直性脊柱炎（AS）的风险较常人高，但需注意：

- 1. 并非所有阳性者都会发病（约5-10%携带者最终患病）
  2. 阴性者仍可能患AS（约10-15%患者为阴性）

**3.辅助诊断价值**
当您出现以下症状时，阳性结果支持AS诊断：
🔹 炎性腰背痛（持续>3个月，活动后改善）
🔹 晨僵
🔹 骶髂关节影像学改变（X线/MRI显示）
🔹 家族史

**4.与其他疾病关联**
阳性也可能与以下疾病相关：
▫️ 反应性关节炎
▫️ 银屑病关节炎
▫️ 葡萄膜炎
▫️ 炎症性肠病相关关节炎

4.可采取的行动
✅ 与风湿免疫科医生讨论症状细节
✅ 结合骶髂关节影像学检查（关键诊断依据）
✅ 必要时进行炎症标志物检测（如CRP、ESR）
✅ 若确诊，早期生物制剂治疗可显著改善预后

请避免仅凭阳性结果自我诊断，需结合临床综合判断。即使阳性未患病，也建议定期监测脊柱关节症状。

12.

**1. 指标的意义**
▸ **CRP**：肝脏产生的急性时相反应蛋白，反映全身炎症程度（正常值通常<5mg/L）
▸ **ESR**：红细胞沉降速率，非特异性炎症/感染标志物（男性正常<15mm/h，女性<20mm/h）

**2. 与强直的相关性**
✅ **支持炎症活动**：在强直活动期，约60-70%患者会出现CRP/ESR升高，提示疾病处于进展状态
✅ **与症状关联**：指标升高常伴随晨僵加重、夜间痛、外周关节肿痛等症状
✅ **治疗监测价值**：抗炎治疗后（如NSAIDs、生物制剂），指标下降提示病情控制有效

**3. 局限性**
⚠️ **非特异性**：感染、其他自身免疫病（如类风湿关节炎、系统性红斑狼疮）、肿瘤等均可导致升高
⚠️ **个体差异**：部分患者即使影像明确显示骶髂关节炎，炎症指标仍可能正常

**4. 临床建议**
🔹 **结合症状判断**：若同时存在炎性腰背痛（持续>3个月，活动后改善）、交替性臀区痛、肌腱端炎等表现，需高度怀疑强直
🔹 **影像学检查**：建议完善骶髂关节MRI（敏感发现骨髓水肿）或CT（显示结构改变）
🔹 **排除感染**：需通过血常规、尿常规等检查排除感染源
🔹 **动态监测**：若确诊强直，建议每1-3个月复查炎症指标以评估病情波动

13.

**1. 疾病阶段特性**
▸ **早期炎症阶段**：X光主要显示结构性改变（如骨侵蚀、关节间隙狭窄），而早期AS以骶髂关节炎症和骨髓水肿为主，此时X光可能完全正常。
▸ **MRI的优势**：建议通过骶髂关节MRI检测骨髓水肿（骨炎），这是早期AS最敏感的影像学表现。

**2. 疼痛来源多样性**
▸ **肌腱端炎**：足跟、坐骨结节等部位的肌腱附着点炎症可能引起剧烈疼痛，但X光难以直接显示。
▸ **脊柱旁软组织炎症**：肌肉、韧带炎症可导致疼痛，需通过查体或超声/MRI评估。
▸ **合并其他痛症**：纤维肌痛、肌筋膜痛综合征等可能与AS共存，需通过压痛点检查等鉴别。

**3. 影像学检查的局限性**
▸ **X光的滞后性**：结构改变通常滞后于症状出现约2-3年，早期患者可能已出现疼痛但X光尚未异常。
▸ **CT的补充作用**：对骶髂关节细微骨质变化的显示优于X光，但仍无法检测活动性炎症。

**4. 临床处理建议**
🔹 **完善MRI检查**：明确骶髂关节骨髓水肿或骨炎
🔹 **动态观察影像学**：间隔6-12个月复查X光/CT，追踪结构变化
🔹 **综合评估症状**：通过BASDAI（巴斯强直性脊柱炎疾病活动指数）等工具量化病情
🔹 **排除其他病因**：需鉴别机械性腰痛、感染、肿瘤等引起的疼痛

14.

**1. 骨髓水肿的本质**
▸ **炎症性改变**：本质是骨髓内血管通透性增加导致液体渗出，常见于感染、创伤或自身免疫性炎症
▸ **MRI敏感显示**：在骶髂关节MRI的STIR序列上呈高信号，是早期强直性脊柱炎（AS）的重要影像学标志

**2. 与强直的关系**
✅ **高度提示早期AS**：若骨髓水肿出现在骶髂关节，且伴随以下特征：
🔹 炎性腰背痛（持续>3个月，活动后改善）
🔹 晨僵>30分钟
🔹 HLA-B27阳性
🔹 CRP/ESR升高
则强烈支持AS诊断（符合ASAS分类标准）
⚠️ **非特异性表现**：骨髓水肿也可能由以下情况引起：
▫️ 暂时性应力反应（如运动员）
▫️ 感染（如骨髓炎）
▫️ 肿瘤（如白血病浸润）

**3. 严重程度判断**
▸ **范围与症状相关**：水肿范围越大，通常提示炎症反应越活跃，疼痛可能更显著
▸ **可逆性改变**：及时抗炎治疗（如NSAIDs、生物制剂）可使水肿消退，但未经治疗可能进展为结构破坏

4🔹 **动态观察**：建议3-6个月后复查MRI，若水肿持续/进展需高度警惕AS
5🔹 **综合评估**：需结合临床表现（如夜间痛、外周关节炎）和实验室指标（HLA-B27、炎症标志物）
6🔹 **鉴别诊断**：排除感染、肿瘤等需通过血常规、骨扫描等检查
7🔹 **治疗启动**：若临床高度怀疑AS，即使X光正常，也可根据ASAS标准启动抗TNF-α治疗

15.

1.判断是否为强直性脊柱炎（AS）需结合以下多维度信息，建议按以下步骤自我评估并及时就医：

**核心症状对照**
✅ **炎性腰背痛**（至少持续3个月，满足以下3项中2项）：
🔹 年龄<40岁
🔹 隐匿起病（非突发）
🔹 活动后改善，休息无缓解
✅ **晨僵**：持续>30分钟，活动后减轻
✅ **交替性臀区痛**：左右臀部交替出现深部疼痛
✅ **外周表现**（非必需但支持诊断）：
🔹 肌腱端炎（足跟痛、坐骨结节痛）
🔹 不对称性外周关节炎（尤其下肢大关节）
🔹 葡萄膜炎、银屑病、炎症性肠病病史

**关键检查解读**

1. **HLA-B27**：阳性增加AS风险，但阴性不排除（约10%患者阴性）
2. **炎症标志物**：
   CRP↑/ESR↑提示炎症活动，但需排除感染/其他自身免疫病
3. **影像学**：
   🔹 **X光/CT**：显示骶髂关节硬化、侵蚀（晚期敏感）
   🔹 **MRI**：检测骶髂关节骨髓水肿（早期敏感，特异性高）

**诊断分层建议**

1. **高度怀疑AS**：
   若符合ASAS分类标准（满足影像学+1条脊柱关节炎特征+至少2条其他SpA特征）
   ▶️ 需风湿免疫科确诊
2. **暂时无法确诊**：
   若仅症状+炎症指标↑但影像学阴性
   ▶️ 建议3-6个月后复查MRI，动态观察
3. **排除其他疾病**：
   ▶️ 机械性腰痛（与活动相关，叩击痛）
   ▶️ 感染（布氏杆菌病、结核等）
   ▶️ 肿瘤（骨髓瘤、转移癌等）

**2.记录症状日记**：包括疼痛时间、性质、缓解因素

**3.尽快就诊**：选择风湿免疫科或脊柱关节病专科

1. **检查优先级**：
   🔹 首选骶髂关节MRI（早期敏感）
   🔹 其次HLA-B27+CRP/ESR
   🔹 必要时行全脊柱MRI排查其他脊柱病变
2. **心理调适**：AS虽无法根治，但早期生物制剂治疗可显著控制病情

**16.**

### ****是否需要「全做」？****

1.✅ **推荐优先完成**：

- **MRI（骶髂关节）**：若病程<2年或X光正常，首选以捕捉早期炎症。
- **HLA-B27 + CRP/ESR**：快速获取遗传和炎症信息。
- **X光/CT**：若MRI提示结构异常或病程较长时补充。

2.⚠️ **可考虑简化**：

- 若已确诊AS且仅需评估病情活动度，可仅复查炎症指标+MRI。
- 若经济/时间受限，可优先MRI+抽血，暂不做CT（辐射较高）。

### ****3.决策建议****

1. **与医生沟通**：
   - 说明症状持续时间、家族史、对辐射的顾虑及经济情况。
   - 例如：「医生，我目前症状较轻且病程较短，能否先做MRI和抽血，若有必要再补充CT？」
2. **分阶段检查**：
   - **第一步**：MRI（明确骨髓水肿）+ HLA-B27 + CRP/ESR。
   - **第二步**：若MRI异常，再行X光/CT观察结构改变。
3. **特殊情况调整**：
   - 备孕/孕期：优先MRI（无辐射），避免CT。
   - 合并感染症状：需增加血常规、降钙素原等感染指标。

17.

### ****1. 发病年龄范围****

- **典型高峰**：20-30岁（青少年/青年为主）
- **成人发病可能**：约30%患者首诊年龄在30岁以上，部分患者40-50岁才出现症状
- **晚发型AS**：极少数患者50岁后发病，可能与基因易感性+环境诱因（如感染、创伤）相关

### ****2. 成人AS的特点****

- **起病更隐匿**：症状可能逐渐加重，而非青少年突发的剧烈疼痛
- **误诊风险高**：常被误判为「腰椎间盘突出」「腰肌劳损」或「骨关节炎」
- **外周关节受累多**：髋关节、膝关节等下肢大关节炎症更常见
- **合并症复杂**：可能伴随高血压、骨质疏松等老年性疾病，增加治疗难度

### ****3. 诊断需警惕的「陷阱」****

- **X光正常≠排除AS**：成人患者可能以脊柱炎症为主，骶髂关节结构改变较轻
- **需结合MRI**：检测脊柱/骶髂关节骨髓水肿（成人AS常见表现）
- **炎症指标波动**：部分患者CRP/ESR仅轻度升高，甚至正常

### ****4.****

✅ **若有以下情况，需排查AS**：

- 炎性腰背痛（夜间痛醒、晨僵>30分钟）
- 交替性臀区痛
- 足跟痛/坐骨结节痛（肌腱端炎）
- 反复虹膜睫状体炎
- 家族中AS/银屑病/炎症性肠病史

✅ **针对性检查**：

- **首选**：全脊柱MRI（观察椎体角炎、韧带骨赘）
- **次选**：骶髂关节MRI（排除早期骶髂关节炎）
- **抽血**：HLA-B27、CRP、ESR、血常规（排除感染/肿瘤）

✅ **治疗提示**：

- 生物制剂（TNF抑制剂）对成人AS同样有效
- 需加强骨质疏松预防（双膦酸盐类药物）
- 康复锻炼（游泳、普拉提）可延缓脊柱僵硬

5.年龄不是AS的“免死金牌”，若出现慢性腰背痛伴晨僵，务必尽早就诊风湿免疫科，避免关节结构永久性破坏。

18.

### ****1. 机械性腰痛（腰肌劳损/腰椎间盘突出）****

- **混淆原因**：均有腰背痛，但机械性腰痛通常：
  🔹 与活动相关（如久坐后加重，活动后缓解）
  🔹 叩击痛明显（AS叩击可减轻）
  🔹 无晨僵或持续时间<30分钟
- **鉴别要点**：AS疼痛夜间加重，活动后改善，伴炎性特征（如交替性臀痛）。

### ****2. 骨关节炎（脊柱退行性变）****

- **混淆原因**：老年患者腰痛常被归因于「老化」。
- **鉴别要点**：
  🔹 骨关节炎疼痛与体位变化相关（如久坐起立时加重）
  🔹 X光显示骨赘形成而非炎性侵蚀
  🔹 无全身炎症指标升高（CRP/ESR正常）。

### ****3. 类风湿关节炎（RA）****

- **混淆原因**：RA可累及脊柱，但更常见于小关节。
- **鉴别要点**：
  🔹 RA呈对称性多关节炎，类风湿因子（RF）/抗CCP抗体阳性
  🔹 AS主要累及中轴关节（骶髂关节、脊柱），HLA-B27阳性率高。

### ****4. 感染相关脊柱炎（布氏杆菌病/结核）****

- **混淆原因**：感染可导致脊柱炎症和骨质破坏。
- **鉴别要点**：
  🔹 感染常伴发热、盗汗等全身症状
  🔹 血常规异常（白细胞↑/淋巴细胞↑）
  🔹 病原体检测（结核菌素试验、布氏杆菌凝集试验）可确诊。

### ****5. 肿瘤（骨髓瘤/转移癌）****

- **混淆原因**：骨痛和影像学异常可能提示恶性病变。
- **鉴别要点**：
  🔹 肿瘤疼痛呈持续性进行性加重，夜间更显著
  🔹 血清蛋白电泳（M蛋白）、肿瘤标志物异常
  🔹 MRI显示溶骨性或成骨性病灶。

### ****6. 银屑病关节炎/肠病性关节炎****

- **混淆原因**：同属脊柱关节病（SpA），症状重叠。
- **鉴别要点**：
  🔹 银屑病关节炎伴皮疹/指甲改变
  🔹 肠病性关节炎合并炎症性肠病（IBD）
  🔹 AS以中轴关节受累为主，外周关节炎非对称。

### ****7. 纤维肌痛综合征****

- **混淆原因**：广泛肌肉疼痛伴疲劳、睡眠障碍。
- **鉴别要点**：
  🔹 纤维肌痛无炎症指标升高或影像学改变
  🔹 压痛点分布广泛（全身18个特定部位）。

8.若出现慢性腰背痛伴晨僵，建议尽早就诊风湿免疫科，通过综合评估减少误诊风险。

19.

### ****一、重新梳理症状，排除「信息差」****

**症状日记**：连续记录2周，重点标注：
🔹 疼痛时间（夜间vs白天）
🔹 晨僵持续时间（是否>30分钟）
🔹 活动后变化（加重/缓解）
🔹 伴随症状（虹膜炎、腹泻、皮疹等）

**关键细节复盘**：
🔹 是否向医生描述过「交替性臀区痛」或「肌腱端炎」？
🔹 家族中是否有银屑病、克罗恩病或AS病史？
🔹 过去是否有反应性关节炎（如肠道/泌尿感染后关节痛）？

### ****二、主动与医生沟通，推动深度排查****

**提出针对性疑问**：
🔹 「我的骶髂关节MRI是否有骨髓水肿？」
🔹 「我的症状是否符合未分化脊柱关节病（uSpA）？」
🔹 「是否需要排查其他SpA相关疾病（如银屑病关节炎）？」

**要求解释排除依据**：
🔹 「您认为我最可能的诊断是什么？依据是？」
🔹 「哪些检查结果排除了AS？为什么？」

### ****三、必要时寻求第二意见****

**选择专科医生**：
🔹 风湿免疫科（尤其擅长脊柱关节病方向）
🔹 如有炎症性肠病/银屑病，联合消化科/皮肤科会诊

**准备材料**：
🔹 整理所有检查报告（影像片+化验单）
🔹 打印症状日记，标注关键时间点

### ****四、心理调适与长期管理****

**接受「不确定性」**：
🔹 部分患者可能处于疾病早期或未分化阶段，需定期随访
🔹 关注症状变化而非过度纠结诊断标签

**症状管理策略**：
🔹 若存在慢性疼痛：尝试物理治疗（如超短波）、康复训练
🔹 若合并焦虑/抑郁：心理咨询（认知行为疗法）或正念练习

**患者社群支持**：
🔹 加入正规病友会（如强直性脊柱炎国际联盟）
🔹 避免在社交平台过度对比症状，减少信息焦虑

### ****五、关键提醒****

✅ **警惕过度检查**：若多家三甲医院已排除AS且缺乏炎症证据，需考虑「功能性疼痛」或「躯体症状障碍」可能。
✅ **动态观察**：即使目前未确诊，若出现新症状（如虹膜炎、炎性肠病），需及时复诊。
✅ **信任专业判断**：医学存在局限性，过度自我诊断可能导致医源性焦虑。

20.

### ****一、优先非药物干预****

**物理治疗**
🔹 热敷/冷敷交替：缓解肌肉痉挛（急性疼痛用冷敷，慢性疼痛用热敷）
🔹 干扰电治疗/超声波：减轻深部炎症（需康复科指导）

**运动疗法**
🔹 猫牛式伸展：改善脊柱活动度（每日2-3组，每组10次）
🔹 游泳/普拉提：增强核心肌群稳定性（避免负重运动）

**心理调节**
🔹 正念呼吸：缓解疼痛焦虑（推荐「478呼吸法」：吸气4秒→屏息7秒→呼气8秒）
🔹 认知重构：记录疼痛时的自动思维，用「可能」替代「一定」（如「我可能过度关注了」）

### ****二、药物选择需谨慎****

1. **NSAIDs（非甾体抗炎药）**
   🔹 适用场景：炎性疼痛（如晨僵、夜间痛）
   🔹 推荐选择：

布洛芬（短期使用，注意胃肠道保护）

塞来昔布（胃肠道风险较低，但心血管风险需评估）
🔹 注意：连续使用≤2周，避免掩盖病情进展

1. **肌肉松弛剂**
   🔹 适用场景：肌肉痉挛性疼痛（需排除神经压迫）
   🔹 常用药物：乙哌立松（需处方，可能引起嗜睡）

#### ****▶ 需避免的药物****

1. **阿片类止痛药**（如曲马多）：可能成瘾，且掩盖病情
2. **糖皮质激素**（如强的松）：未明确病因前禁用
3. **免疫抑制剂/生物制剂**（如TNF抑制剂）：需确诊后使用

### ****三、关键行动清单****

1. **疼痛记录表**：连续记录1周，标注疼痛性质（钝痛/锐痛）、诱因（姿势/活动）、缓解方式
2. **二次就诊准备**：
   🔹 整理所有检查报告（影像学+实验室）
   🔹 拍摄疼痛部位照片（如皮肤变化、肿胀）
   🔹 准备2-3个关键问题（如「我的骶髂关节MRI是否有骨髓水肿？」）
3. **多学科会诊**：若仍无明确诊断，可联合风湿免疫科+康复科+疼痛科综合评估

### ****四、警惕「红 quart flags」****

若出现以下情况，需立即就医：
🚩 疼痛突然加剧伴发热
🚩 下肢麻木/无力（提示神经受压）
🚩 体重下降/夜间盗汗（需排除肿瘤/感染）
🚩 视力障碍（警惕葡萄膜炎）

21

### ****一、常见检查的辐射剂量****

| **检查类型** | **平均辐射剂量（mSv）** | **相当于** |
| --- | --- | --- |
| **胸片（X光）** | 0.1 | 约10天自然背景辐射 |
| **腰椎X光** | 1.5 | 约6个月自然背景辐射 |
| **头颅CT** | 2 | 约2年自然背景辐射 |
| **胸部CT** | 7 | 约3年自然背景辐射 |
| **腹部/骨盆CT** | 10 | 约4年自然背景辐射 |
| **冠脉CTA** | 12 | 约5年自然背景辐射 |

**注**：

- 自然背景辐射（每年）≈2.4 mSv
- 往返纽约-东京航班≈0.1 mSv
- 吸烟者每年额外辐射≈1.5 mSv（来自烟草中的钋-210）

### ****二、健康影响评估****

**短期风险**：
🔹 单次检查（<50 mSv）致癌风险可忽略（约1/1,000,000）
🔹 孕妇需谨慎：胎儿对辐射更敏感（尤其妊娠前3个月）

**长期累积风险**：
🔹 年辐射暴露>100 mSv可能增加癌症风险（需长期跟踪）
🔹 儿童风险高于成人（细胞分裂更活跃）

### ****三、风险-收益平衡****

✅ **必要检查不可替代**：
🔹 骨折/肺炎诊断需X光
🔹 肿瘤/血管病变需CT
🔹 脊柱关节炎需骶髂关节CT/MRI

⚠️ **避免过度检查**：
🔹 反复低热剂量检查（如每年多次胸片）可能累积风险
🔹 非必要重复扫描需与医生沟通

### ****四、辐射防护建议****

**患者层面**：
🔹 检查前告知医生妊娠状态或近期其他检查
🔹 要求使用铅衣保护甲状腺/性腺

**技术层面**：
🔹 选择低剂量CT（如胸部LDCT筛查）
🔹 优先MRI/超声（无辐射，但MRI对金属植入物有限制）

### ****五、总结行动指南****

1.**单次检查**：无需过度担忧，辐射量通常低于日常活动风险

2.**多次检查**：与医生讨论检查必要性，优先无辐射替代方案

3.**特殊人群**：孕妇、儿童需严格把握适应症

22.

### ****一、诊断标准的「硬性要求」****

目前国际通用的AS分类标准（如**ASAS标准**）需满足：

**影像学证据**（必备）：
🔹 骶髂关节炎（X光/CT显示≥2级，或MRI显示活动性炎症）
🔹 **若缺乏此条，即使其他指标全符合，仍无法确诊AS**

**临床指标**（需满足至少1项）：
🔹 炎性腰背痛（晨僵>30分钟，活动后改善）
🔹 关节炎/肌腱端炎/虹膜炎等关节外表现

**您的现状可能**：
✅ HLA-B27阳性（支持但非必需）
✅ 炎症标志物升高（CRP/ESR↑）
✅ 症状符合（如夜间痛、晨僵）
❌ **但骶髂关节影像学未达确诊标准**（如仅0-1级硬化）

### ****二、医生未确诊的潜在考量****

**排除「模仿者」**：
🔹 感染（布鲁氏菌病、结核性脊柱炎）
🔹 肿瘤（骨髓瘤、转移癌）
🔹 其他脊柱关节病（银屑病关节炎、反应性关节炎）

**避免过度诊断**：
🔹 部分患者可能处于「未分化脊柱关节病（uSpA）」阶段
🔹 早期影像学可能仅显示骨水肿而无结构破坏

**动态观察需要**：
🔹 症状可能由机械性腰痛（如肌筋膜炎）叠加炎症引起
🔹 需随访观察影像学进展（建议6-12个月后复查MRI）

### ****三、下一步行动建议****

**深度沟通**：
🔹 询问医生：「我的骶髂关节影像学具体分级是？未达标的关键点在哪？」
🔹 探讨：「是否需要短期使用NSAIDs进行诊断性治疗？」

**针对性检查**：
🔹 骶髂关节MRI（检测骨髓水肿敏感性高于X光）
🔹 全脊柱MRI（排查椎角炎等其他SpA特征性病变）
🔹 感染/肿瘤筛查（如结核T-SPOT、骨髓穿刺）

**多学科评估**：
🔹 风湿免疫科+康复科+疼痛科联合制定管理方案
🔹 若合并肠病/银屑病，需消化科/皮肤科会诊

### ****四、症状管理策略****

即使未确诊AS，仍可采取以下措施：
🔹 **药物**：短期使用塞来昔布（需医生评估）
🔹 **康复**：施罗斯三维矫形训练改善姿势
🔹 **监测**：记录症状变化（如虹膜炎、炎性肠病新发病症）

23.

### ****1. 疾病发展的可能性****

强直性脊柱炎（AS）的核心问题是炎症导致的脊柱/关节骨化融合，若未有效控制：

- **晚期可能出现**：脊柱强直（“竹节样变”）、髋关节融合等结构性改变，影响活动能力。
- **但并非必然残疾**：现代医学通过早期干预可显著延缓病情，多数患者能维持正常生活。

### ****2. 决定预后的关键因素****

- **治疗是否及时规范**：生物制剂（如TNF抑制剂）可快速控制炎症，越早使用效果越好。
- **炎症控制水平**：持续炎症会加速骨化，需定期监测血沉（ESR）、C反应蛋白（CRP）等指标。
- **日常自我管理**：保持适度运动（如游泳、瑜伽）、避免久坐、纠正不良姿势能延缓脊柱僵硬。

### ****3. 残疾风险分层****

- **低风险人群**：早期诊断+规范治疗+依从性高者，多数可避免严重畸形。
- **高风险信号**：反复严重发作、髋关节受累、家族遗传性强直病史者需更积极干预。

### ****4. 关键行动建议****

- **医疗层面**：
  - 与风湿免疫科专家合作，制定个体化治疗方案（药物+康复）。
  - 每6-12个月评估影像学（X线/MRI）和炎症指标。
- **生活层面**：
  - **运动疗法**：进行脊柱灵活性训练（如猫牛式伸展）、呼吸训练（改善胸廓活动度）。
  - **心理支持**：加入病友社群，学习疾病管理技巧，减少焦虑对病情的影响。
  - **职业保护**：避免重体力劳动或长期保持同一姿势的工作。

### ****5. 残疾预防的积极信号****

- **药物革命**：IL-17抑制剂等新型生物制剂对难治性AS效果更优。
- **手术支持**：严重髋关节/膝关节受累可通过关节置换恢复功能。
- **早期筛查**：MRI可发现骶髂关节炎早于X线2-3年，为干预争取时间。

24.

### ****1. 用药时间的决定性因素****

- **疾病活动性**：若炎症指标（ESR/CRP）持续升高或影像学进展，需长期用药。
- **药物类型**：
  - **非甾体抗炎药（NSAIDs）**：按需使用，症状缓解后可减量或停用，但可能反复。
  - **生物制剂（如TNF抑制剂）**：多需持续使用，部分患者在病情稳定2-3年后可尝试减量或延长给药间隔。
  - **传统合成DMARDs（如柳氮磺吡啶）**：常需长期维持，尤其伴外周关节炎者。
- **个体化差异**：部分患者可能通过短期强化治疗实现长期缓解，但需密切监测。

### ****2. 停药或减量的条件****

- **临床缓解标准**：无活动性炎症（ESR/CRP正常）、无新发骨赘、症状消失≥6个月。
- **影像学稳定**：MRI或X线显示无新骨化进展。
- **医生评估**：需风湿免疫科专家综合判断，切勿自行停药。

### ****3. 长期用药管理建议****

- **定期复诊**：每3-6个月评估炎症指标、肝功能及药物副作用。
- **副作用监测**：生物制剂需注意感染、结核风险；NSAIDs警惕胃肠道/心血管风险。
- **药物调整策略**：病情稳定后，可能从“连续给药”转为“按需给药”，或联合更低剂量维持。

### ****4. 关键行动方向****

- **与医生合作**：制定个性化治疗目标（如“无药物缓解”或“最低有效剂量维持”）。
- **生活干预**：通过运动、戒烟等降低疾病活动性，减少药物依赖。
- **心理准备**：接受AS的慢性病程，理解用药可能是“动态调整”而非“固定疗程”。

25.

### ****1. 可接种的疫苗类型****

- **灭活疫苗**（如流感疫苗、新冠疫苗灭活制剂、肺炎球菌疫苗）：通常安全，免疫抑制患者推荐接种。
- **重组亚单位疫苗**（如乙肝疫苗、HPV疫苗）：可正常接种。

### ****2. 需谨慎的疫苗类型****

- **减毒活疫苗**（如水痘疫苗、麻疹-腮腺炎-风疹疫苗、黄热病疫苗）：
  ➤ **若正在使用生物制剂（如TNF抑制剂）或高剂量免疫抑制剂**：可能增加感染风险，需暂缓接种。
  ➤ **若病情稳定且未使用强效免疫抑制剂**：可咨询医生后接种。

### ****3. 接种时机选择****

- **最佳时机**：疾病稳定期、未处于急性炎症发作期。
- **与药物间隔**：
  ➤ **生物制剂**：部分指南建议接种灭活疫苗后暂停生物制剂1周（具体需遵医嘱）。
  ➤ **甲氨蝶呤/柳氮磺吡啶**：通常无需调整，但严重免疫抑制者需评估。

### ****4. 关键注意事项****

- **咨询主治医生**：告知当前用药（如生物制剂、激素、免疫抑制剂）及病情活动度。
- **抗体检测**：接种后若担心免疫应答不足，可检测保护性抗体水平（如乙肝、流感）。
- **优先推荐疫苗**：
  ➤ **流感疫苗**：每年接种，降低感染风险。
  ➤ **肺炎球菌疫苗**：建议免疫抑制患者接种。
  ➤ **新冠疫苗**：按当地指南完成全程接种。

5.通过合理规划疫苗接种，可在控制疾病的同时降低感染风险。具体方案请与风湿免疫科医生及疫苗接种医生共同制定。

26.

### ****1. 抗炎药（抗炎药物）****

- **核心作用**：抑制**无菌性炎症**（如AS的脊柱/关节炎症）。
- **代表药物**：非甾体抗炎药（NSAIDs），如布洛芬、双氯芬酸钠、塞来昔布等。
- **AS治疗价值**：
  ✅ **一线选择**：快速缓解腰背痛、晨僵及关节炎症。
  ✅ **抗炎+止疼双重作用**：既控制病因（炎症），又改善症状。
- **注意**：需警惕长期使用的胃肠道、心血管副作用。

### ****2. 消炎药（抗感染药物）****

- **核心作用**：杀灭或抑制**病原微生物**（如细菌、病毒、真菌）。
- **代表药物**：抗生素（青霉素、头孢类）、抗病毒药（奥司他韦）等。
- **AS治疗误区**：
  ❌ **不适用**：AS是自身免疫病，非细菌感染，抗生素无效。
  ❌ **避免滥用**：仅在有明确感染（如合并肺炎）时使用。

### ****3. 止疼药（镇痛药物）****

- **核心作用**：单纯**缓解疼痛**，不针对炎症或病因。
- **代表药物**：对乙酰氨基酚（扑热息痛）、弱阿片类（曲马多）等。
- **AS治疗局限**：
  ⚠️ **短期辅助**：可用于疼痛剧烈时临时缓解症状。
  ⚠️ **不控制病情**：无法阻止AS的炎症进展和骨破坏。

### ****4.行动建议****

**以抗炎药为核心**：NSAIDs是AS治疗的基石，需按医嘱规律使用。

**避免混淆“消炎”与“抗炎”**：拒绝无指征的抗生素使用。

**疼痛管理分层**：轻度疼痛用NSAIDs，重度可短期联合弱阿片类。

**定期评估**：与医生沟通调整药物，监测肝肾功能及副作用。

27.

在已确诊且疼痛未缓解的情况下，是否需要追加止痛药需综合考虑以下因素：

1. 药物类型与用药规则

非甾体抗炎药（如布洛芬、双氯芬酸钠）：通常需间隔4-6小时，24小时内不超过最大剂量（布洛芬成人≤1.2g/日）。若未达当日上限且间隔足够，可遵医嘱加服。

对乙酰氨基酚（扑热息痛）：需间隔4-6小时，24小时≤4g。过量可能导致肝损伤，需严格计算已服用量。

阿片类（如曲马多）：需严格遵医嘱，不可自行调整剂量。

2. 疼痛评估与替代方案

若当前药物未起效，可能需升级止痛方案（如联合用药或更换更强效药物），但需医生评估。

可尝试非药物止痛法（如冰敷、热敷、体位调整）辅助缓解。

3. 风险提示

自行加量可能增加胃肠道出血、肝肾负担或药物依赖风险。

长期未缓解的疼痛需警惕病情变化，建议及时复诊。

4.核对药物说明书或医嘱，确认已用剂量是否安全；

5.若未超量且间隔足够，可尝试加服一次；

6.若疼痛持续＞3天或加重，或出现新症状（如发热、肿胀），务必联系医生调整治疗方案。

28.

### ****1. 药物相关可能性****

- **维生素B2（核黄素）**：若复合维生素或营养补充剂中含此成分，会导致尿液明显发黄，属正常现象。
- **NSAIDs（如布洛芬、双氯芬酸）**：虽不直接染色尿液，但可能引起胃肠道不适导致脱水，使尿液浓缩变黄。
- **生物制剂/免疫抑制剂**：一般不会直接引起尿液颜色改变，但需警惕肝功能异常间接导致黄疸（详见下文）。

### ****2. 需警惕的健康信号****

- **肝功能异常**：某些药物（如甲氨蝶呤）可能损伤肝脏，导致胆红素升高，尿液呈深黄色或茶色，常伴皮肤/巩膜黄染。
- **尿路感染**：尿频、尿急、尿痛伴尿液浑浊或异味，需尿常规检查。
- **血尿**：尿液发红或酱油色，提示肾脏或泌尿系统问题，需紧急就医。

**4.观察伴随症状**：

- - 有无皮肤/眼睛发黄、乏力、恶心（提示肝损伤）。
  - 有无排尿不适、发热（提示感染）。
  - 尿液是否持续深黄或加重。

**5.调整生活习惯**：

- - **增加饮水**：每日饮水＞1.5L，观察尿液是否变淡。
  - **避免脱水**：服药后若出现腹泻/出汗增多，需及时补液。

**6.记录用药清单**：

- - 列出所有药物（包括中药、保健品），咨询医生或药师是否含染色成分。

**7.及时就医指征**：

- - 尿液持续深黄＞3天，伴疲劳、食欲下降。
  - 出现尿频/尿急/尿痛、尿液浑浊或血色。
  - 皮肤/巩膜黄染。

### ****8.与医生沟通要点****

- 提供尿液颜色变化的时间、伴随症状及用药清单。
- 建议检查：尿常规、肝功能（尤其胆红素）、肾功能。
- 勿自行停药，但需告知医生疑虑以便调整方案。

29.

### ****1. 什么是生物制剂？****

- **定义**：利用生物工程技术（如基因重组）制造的蛋白质类药物，可**特异性阻断导致炎症的关键分子**（如肿瘤坏死因子TNF-α、白细胞介素IL-17等）。
- **与传统药物的区别**：
  ➤ **作用靶点明确**：针对AS病理核心（如TNF-α驱动的炎症），而非广泛抑制免疫。
  ➤ **起效更快**：通常数周见效，优于传统DMARDs（需数月）。
  ➤ **骨保护作用强**：可显著抑制新骨形成，延缓脊柱强直。

### ****2. 生物制剂在AS中的核心作用****

- **适应症**：
  ✅ NSAIDs疗效不足或不耐受
  ✅ 伴外周关节炎、肌腱端炎
  ✅ 影像学提示早期骨破坏
  ✅ 高疾病活动度（BASDAI≥4）
- **常见类型**：
  ➤ **TNF抑制剂**：阿达木单抗、依那西普、英夫利昔单抗
  ➤ **IL-17抑制剂**：司库奇尤单抗、依奇珠单抗
  ➤ **IL-23抑制剂**：古塞奇尤单抗
  ➤ **JAK抑制剂**：托法替布（口服小分子靶向药）

### ****3. 优势与风险****

- **优势**：
  ➤ **快速控制炎症**：改善晨僵、疼痛及关节活动度
  ➤ **延缓疾病进展**：降低脊柱融合风险
  ➤ **提升生活质量**：部分患者可恢复工作能力
- **风险**：
  ⚠️ **感染风险增加**：需筛查结核、乙肝，治疗中警惕肺炎、皮肤感染
  ⚠️ **过敏反应**：注射部位红肿、荨麻疹，罕见严重过敏反应
  ⚠️ **免疫原性**：长期使用可能产生抗药抗体（尤其TNF抑制剂）

### ****4. 使用注意事项****

- **筛查要求**：用药前需检测结核、乙肝、丙肝，部分需胸部CT。
- **联用规则**：常与甲氨蝶呤联用增强疗效，减少抗药抗体产生。
- **禁忌症**：活动性感染、心力衰竭（III/IV级）、脱髓鞘病史。
- **监测频率**：每3-6个月评估血常规、肝肾功能及感染迹象。

### ****5. 与传统治疗的协同****

- **一线方案**：NSAIDs + 生物制剂（尤其伴外周关节炎或高疾病活动度）。
- **长期管理**：病情稳定后可能逐步减量生物制剂，保留传统药物维持。
- **个体化选择**：如TNF抑制剂无效，可换用IL-17/IL-23抑制剂。

30.

### ****一、常见副作用及发生频率****

| **副作用类型** | **典型表现** | **发生频率** | **预防措施** |
| --- | --- | --- | --- |
| **感染风险增加** | 上呼吸道感染、尿路感染、结核/乙肝复发 | 常见（约10%-30%患者） | 用药前筛查结核/乙肝/丙肝；治疗中警惕发热、咳嗽等症状 |
| **注射部位反应** | 红肿、瘙痒、疼痛 | 较常见（约20%） | 轮换注射部位；局部冷敷或抗组胺药 |
| **过敏反应** | 荨麻疹、皮疹；罕见过敏性休克 | 罕见（<1%） | 首次注射后观察30分钟；备肾上腺素 |
| **免疫原性** | 抗药抗体产生（导致疗效下降） | TNF抑制剂较高（约10%-20%） | 联用甲氨蝶呤可降低风险 |
| **肝功能异常** | 转氨酶升高 | 偶见（<5%） | 定期监测肝功能；避免酗酒 |
| **脱髓鞘疾病** | 多发性硬化（罕见） | 极罕见（<0.1%） | 用药前评估神经系统病史 |

### ****二、不同生物制剂的副作用差异****

- **TNF抑制剂**（如阿达木单抗）：
  ➤ 感染风险较高，尤其结核和机会感染。
  ➤ 可能诱发或加重心力衰竭（NYHA III/IV级禁用）。
- **IL-17抑制剂**（如司库奇尤单抗）：
  ➤ 黏膜感染（如口腔/肠道念珠菌病）相对多见。
  ➤ 炎症性肠病（IBD）患者可能加重病情，需谨慎。
- **IL-23抑制剂**（如古塞奇尤单抗）：
  ➤ 总体安全性较好，但长期数据较TNF抑制剂少。
- **JAK抑制剂**（如托法替布）：
  ➤ 血栓风险（尤其高龄、吸烟者）需关注。

### ****三、长期安全性与特殊人群****

- **妊娠与哺乳**：
  ➤ TNF抑制剂在妊娠中晚期使用相对安全（需权衡利弊）。
  ➤ 大部分生物制剂在哺乳期需谨慎（乳汁分泌少量可能）。
- **儿童与青少年**：
  ➤ 疗效明确，但长期感染风险需密切监测。
- **老年患者**：
  ➤ 感染风险更高，需严格筛查并调整剂量。

### ****四、副作用管理策略****

1. **用药前筛查**：
   - 结核（PPD/T-SPOT）、乙肝（HBsAg/HBcAb）、丙肝抗体、HIV。
   - 胸部CT（尤其高危人群）。
2. **治疗中监测**：
   - 每3-6个月复查血常规、肝肾功能。
   - 出现发热持续＞2天、咳嗽/尿频/皮肤破损等及时就医。
3. **感染预防**：
   - 接种灭活疫苗（如流感、肺炎球菌疫苗）避免活疫苗。
   - 避免接触结核患者；注意口腔卫生防念珠菌感染。
4. **副作用处理**：
   - 轻度感染：局部处理或口服抗生素，无需停药。
   - 严重感染：暂停生物制剂，联合抗感染治疗。
   - 过敏反应：立即停药，抗组胺或糖皮质激素治疗。

### ****五、综合决策建议****

✅ **适用人群**：

- NSAIDs疗效不足或不耐受
- 高疾病活动度（BASDAI≥4）伴炎症标志物升高
- 影像学提示早期骨破坏
  ⚠️ **慎用情况**：
- 反复感染史、活动性结核/乙肝
- 充血性心力衰竭、脱髓鞘病史
- 恶性肿瘤（近5年内）

31.

### ****1. 药理机制层面****

- **作用靶点明确**：生物制剂通过抑制特定炎症因子（如TNF-α、IL-17）发挥作用，**不作用于中枢神经系统**，不涉及多巴胺等成瘾相关通路的激活。
- **无精神活性**：与阿片类、苯二氮䓬类药物不同，生物制剂无镇静、欣快等主观效应，**缺乏生理依赖性基础**。

### ****2. 长期使用观察****

- **无成瘾性证据**：全球长期随访数据（如阿达木单抗超10年研究）未报告成瘾案例。
- **停药后反应**：部分患者停药后可能出现疾病复发（如关节疼痛、晨僵），但这是疾病自然病程的体现，**非戒断症状**。

### ****3. 心理依赖风险****

- **可能存在的认知偏差**：患者因感知到药物显著改善生活质量，可能产生“心理依赖感”，担心停药后症状反复。
- **应对策略**：
  ➤ 医患沟通：明确疾病需长期管理，药物是控制手段而非依赖对象。
  ➤ 逐步减量：病情稳定后，在医生指导下尝试降低剂量或延长用药间隔。

### ****4. 与其他药物的对比****

| **药物类型** | **成瘾性风险** | **作用机制** | **停药后反应** |
| --- | --- | --- | --- |
| **生物制剂** | 无 | 抑制炎症因子 | 疾病可能复发 |
| **阿片类止痛药** | 高 | 激活μ受体（多巴胺通路） | 生理戒断症状（如焦虑、颤抖） |
| **糖皮质激素** | 低（生理依赖可能） | 免疫抑制+抗炎 | 肾上腺功能不全（长期大剂量后） |

### ****5. 患者行动建议****

- **规范用药**：遵循医嘱，不自行调整剂量或停药。
- **定期评估**：每3-6个月复查疾病活动度（BASDAI、CRP、影像学）。
- **心理调适**：通过运动、康复锻炼等非药物手段增强疾病管理能力，减少对药物的“心理依赖感”。

32.

### ****一、优先处理感染****

1. **乙肝患者**
   1. **抗病毒治疗**：
      1. 首选强效低耐药药物（如恩替卡韦、替诺福韦）。
      2. 治疗目标：HBV-DNA转阴，肝功能稳定。
   2. **监测**：每3-6个月复查HBV-DNA、肝功能。
   3. **生物制剂使用条件**：抗病毒治疗后，HBV-DNA阴性且肝功能正常，可谨慎联用生物制剂（需密切监测）。
2. **结核患者**
   1. **抗结核治疗**：
      1. 标准四联疗法（异烟肼+利福平+吡嗪酰胺+乙胺丁醇）至少2周。
      2. 疗程需完成（活动性结核通常6个月）。
   2. **生物制剂重启时机**：抗结核治疗≥2周且病情稳定后，由专科医生评估。
3. **其他感染**
   1. **丙肝**：直接抗病毒药物（DAAs）治愈后，可安全使用生物制剂。
   2. **HIV**：抗病毒治疗后CD4>200/μL且病毒载量不可测时，可谨慎使用。
   3. **机会感染**：如真菌、分枝杆菌感染，需针对性治疗后评估。

### ****二、替代药物治疗方案****

#### ****1. 一线药物****

- **NSAIDs**（如塞来昔布、美洛昔康）：
  - 控制疼痛和炎症，但需警惕胃肠道/心血管风险。
  - 可联合PPI（如奥美拉唑）护胃。
- **糖皮质激素**（如甲泼尼龙）：
  - 短期用于急性炎症（如外周关节炎），避免长期大剂量。

#### ****2. 传统DMARDs****

- **甲氨蝶呤**（MTX）：
  - 每周7.5-25mg，可延缓影像学进展。
  - 乙肝患者需权衡肝毒性风险。
- **柳氮磺吡啶**（SSZ）：
  - 每日2-3g，对外周关节炎有效，起效较慢。
- **来氟米特**：
  - 每日10-20mg，适合MTX不耐受者。

#### ****3. 其他靶向药物****

- **JAK抑制剂**（如托法替布）：
  - 口服小分子药物，对AS有效，但需评估感染风险（如疱疹病毒再激活）。
  - 乙肝患者需密切监测病毒载量。

### ****三、非药物治疗****

1. **运动疗法**：
   - 游泳、瑜伽、脊柱灵活性训练，维持关节功能。
   - 避免高强度负重运动。
2. **物理因子治疗**：
   - 热敷、超声波、电刺激缓解疼痛和肌肉痉挛。
3. **患者教育**：
   - 了解疾病进程，避免久坐，保持正确姿势。

### ****四、监测与随访****

- **感染指标**：定期筛查乙肝、结核、丙肝等（如每3-6个月）。
- **疾病活动度**：BASDAI、ASDAS、CRP、影像学（每年脊柱X线）。
- **药物副作用**：监测肝肾功能、血常规。

### ****五、特殊情况处理****

- **妊娠需求**：
  - MTX、来氟米特需停药3-6个月，换用硫唑嘌呤或羟基氯喹过渡。
  - 生物制剂中TNF抑制剂在孕中晚期相对安全。
- **老年/儿童患者**：
  - 老年：优先NSAIDs+局部激素，避免过度免疫抑制。
  - 儿童：MTX或生物制剂（严格筛查感染）。

33.

### ****一、通常需要联用药物****

1. **传统DMARDs（如甲氨蝶呤）**
   1. **作用**：增强生物制剂疗效，减少抗药抗体产生（TNF抑制剂联用可降低免疫原性）。
   2. **剂量**：每周7.5-25mg（需根据肾功能调整）。
   3. **禁忌症**：严重肝病、酗酒、骨髓抑制。
2. **NSAIDs（如塞来昔布）**
   1. **作用**：快速缓解关节疼痛/晨僵，与生物制剂协同控制症状。
   2. **注意**：长期使用需监测胃肠道/心血管风险，可联合PPI（如奥美拉唑）。
3. **糖皮质激素（如甲泼尼龙）**
   1. **适用场景**：短期控制急性炎症（如外周关节炎、虹膜炎），或作为桥接治疗。
   2. **原则**：小剂量（≤7.5mg/日）长期使用需谨慎（骨质疏松、感染风险）。

### ****二、可能需要的辅助药物****

1. **钙剂+维生素D**
   1. **目的**：预防激素或活动期疾病导致的骨量流失。
   2. **监测**：每年骨密度检查（尤其长期使用激素者）。
2. **叶酸**
   1. **适用**：联用甲氨蝶呤时补充（5mg/周），减少血液毒性。
3. **局部用药**
   1. **外用NSAIDs**：局部关节肿痛可辅助使用（如双氯芬酸凝胶）。
   2. **关节腔注射激素**：难治性单关节炎症可局部注射。

### ****三、非药物治疗联合****

1. **运动疗法**：
   1. 游泳、瑜伽改善脊柱灵活性；抗阻训练增强肌肉力量。
2. **物理治疗**：
   1. 热敷、超声波缓解肌肉痉挛；支具纠正姿势。
3. **患者教育**：
   1. 戒烟、控制体重；避免久坐/不良姿势。

### ****四、特殊场景调整****

- **感染风险高**：停用免疫抑制剂（如MTX），仅保留生物制剂+NSAIDs。
- **妊娠计划**：停MTX、来氟米特，换用硫唑嘌呤或生物制剂单药治疗。
- **老年/儿童**：减少药物种类，优先生物制剂+最低有效剂量NSAIDs。

### ****五、动态调整原则****

1. **定期评估**：每3-6个月复查疾病活动度（BASDAI、ASDAS）、炎症指标（CRP）。
2. **减量策略**：病情稳定后，可尝试减少激素或NSAIDs剂量，避免长期副作用。
3. **停药指征**：持续缓解（BASDAI<2且影像学稳定）≥6个月，可逐步停用生物制剂。

34.

### ****一、停药可行性评估****

1. **核心标准**
   1. **持续临床缓解**：BASDAI评分<2分（无疲劳、疼痛、晨僵），持续≥6个月。
   2. **炎症指标正常**：CRP、ESR降至正常范围。
   3. **影像学稳定**：脊柱X线/MRI显示无新骨赘形成或炎症活动。
2. **个体化因素**
   1. **疾病病程**：早期患者停药后复发风险较低。
   2. **合并症**：无银屑病、肠炎等共病者更易成功停药。
   3. **药物反应**：首次使用生物制剂即达缓解者预后较好。

### ****二、停药策略****

1. **逐步减量法**
   1. **TNF抑制剂**：延长给药间隔（如从每2周→每4周），观察3个月。
   2. **IL-17抑制剂**：减量需谨慎（免疫原性风险较高）。
2. **直接停药法**
   1. **适用场景**：短期用药（<1年）且快速达缓解者。
   2. **监测**：停药后前3个月每4周复查，之后每8周监测。

### ****三、停药后管理****

1. **复发预警信号**
   1. 晨僵>30分钟、夜间痛醒、外周关节肿胀。
   2. CRP升高>5mg/L或血沉增快。
   3. 影像学提示新炎症病灶。
2. **复发处理方案**
   1. **早期复发**（停药后3个月内）：重启原生物制剂。
   2. **晚期复发**（>6个月）：重新评估病情，可能换用其他机制药物（如JAK抑制剂）。

### ****四、长期维持策略****

1. **非药物手段**
   1. **运动疗法**：每周3次游泳/瑜伽，维持脊柱活动度。
   2. **饮食管理**：地中海饮食（富ω-3脂肪酸）降低炎症。
   3. **戒烟/控重**：吸烟者复发风险增加2倍。
2. **药物桥接**
   1. **NSAIDs按需使用**：短期控制偶发症状。
   2. **传统DMARDs低剂量维持**：如甲氨蝶呤5-10mg/周。

### ****五、特殊场景决策****

| **场景** | **建议** |
| --- | --- |
| **妊娠计划** | 孕前3个月停生物制剂，换用硫唑嘌呤或暂停治疗（低疾病活动度时） |
| **术后/感染** | 暂停生物制剂，改用糖皮质激素短期过渡 |
| **经济因素** | 换用生物类似药（如阿达木单抗生物类似药），或申请患者援助计划 |

生物制剂的停用需严格遵循“评估-减量-监测-重启”流程，在医生指导下的个体化决策是避免病情反复的关键。

35.

### ****一、激素的适用场景****

1. **急性炎症控制**
   1. **外周关节炎**：如膝关节、踝关节严重肿胀，可局部关节腔注射（如甲泼尼龙）。
   2. **肌腱端炎**：如足跟痛（跟腱炎），局部注射快速缓解症状。
   3. **虹膜炎/葡萄膜炎**：眼科紧急情况时，需大剂量激素冲击（如静脉注射甲强龙）。
2. **桥接治疗**
   1. 在生物制剂/传统DMARDs起效前（通常需数周），短期口服激素（如泼尼松20-30mg/日）控制症状。
3. **全身炎症反应**
   1. 罕见情况下（如合并巨噬细胞活化综合征），需大剂量激素挽救生命。

### ****二、激素的使用原则****

1. **短期性**：连续使用≤3个月，避免长期依赖。
2. **最低剂量**：口服激素建议≤7.5mg/日（泼尼松等效剂量）。
3. **局部优先**：能局部注射不全身用药，减少副作用。

### ****三、激素的副作用与监测****

| **副作用类型** | **监测/预防措施** |
| --- | --- |
| **骨质疏松** | 补钙（1000-1200mg/日）+维生素D（800-1000IU/日）；骨密度检测（每年） |
| **感染风险** | 避免用于活动性结核/乙肝未控制者；监测血常规（白细胞变化） |
| **血糖升高** | 糖尿病患者加强血糖监测，必要时调整降糖方案 |
| **消化道损伤** | 联合PPI（如奥美拉唑）预防胃溃疡 |
| **心理影响** | 高剂量时可能出现情绪波动，需心理支持 |

### ****四、激素与其他药物的联用****

- **生物制剂**：激素可短期联用加速症状控制，但长期需逐步减量。
- **传统DMARDs**：如甲氨蝶呤、来氟米特起效后，优先替代激素。
- **NSAIDs**：激素与NSAIDs联用增加胃肠道风险，需密切监测。

### ****五、替代方案****

- **轻中度炎症**：优先NSAIDs（如塞来昔布）+物理治疗。
- **中重度活动**：生物制剂（如TNF抑制剂）+传统DMARDs（如甲氨蝶呤）。
- **难治性病例**：JAK抑制剂（如托法替布）或IL-17抑制剂（如司库奇尤单抗）。

激素在AS治疗中扮演“消防队员”角色，适合快速控制急性问题，但长期管理需依赖其他更安全的药物和非药手段。建议在风湿免疫科医生指导下制定个体化方案。

36.

### ****一、术前药物调整原则****

1. **生物制剂**
   1. **停药时机**：择期手术前1-2个药物半衰期（如TNF抑制剂需提前2-4周，IL-17抑制剂需更早）。
   2. **感染风险手术**（如关节置换、脊柱矫形术）：必须停用生物制剂，术后感染控制后再重启。
   3. **紧急手术**（如创伤）：无需等待停药，但需术中预防性抗生素。
2. **传统DMARDs**
   1. **甲氨蝶呤/来氟米特**：术前1周停用，避免术后伤口愈合延迟。
   2. **硫唑嘌呤**：无需调整，但需监测白细胞。
3. **NSAIDs**
   1. **术前3-5天停用**：减少出血风险（尤其阿司匹林、布洛芬）。
   2. **替代方案**：术后可改用塞来昔布（选择性COX-2抑制剂）镇痛。
4. **糖皮质激素**
   1. **生理剂量**（<7.5mg/日泼尼松）：无需调整，术中需应激剂量覆盖。
   2. **大剂量使用**：需逐渐减量，避免肾上腺危象。

### ****二、手术类型特异性管理****

| **手术类型** | **药物管理建议** |
| --- | --- |
| **小手术** | 仅停NSAIDs，生物制剂可继续使用（如眼科手术、牙科操作） |
| **择期大手术** | 停生物制剂+DMARDs，保留低剂量激素（如甲泼尼龙4-8mg/日） |
| **急诊手术** | 不中断治疗，术中加强感染预防（如万古霉素+头孢唑林） |
| **脊柱融合术** | 术前停用生物制剂≥4周，术后复查炎症指标决定是否重启 |

### ****三、术后恢复用药策略****

1. **重启时机**：
   1. **伤口愈合后**（通常术后2-4周）且无感染迹象时恢复生物制剂。
   2. **NSAIDs**：术后24小时即可恢复（无活动性出血时）。
2. **感染监测**：
   1. 术后3个月内每2周复查CRP、ESR，警惕隐匿感染。
   2. 发热或伤口红肿需立即评估（可能需暂停生物制剂）。

### ****四、多学科协作建议****

1. **术前多学科会诊**：
   1. 风湿免疫科、外科、麻醉科共同制定方案，权衡疾病控制与手术风险。
2. **患者教育**：
   1. 告知术后可能出现的疾病复发风险（如停药后疼痛加重），提前准备预案。
3. **过渡治疗**：
   1. 高疾病活动度患者：术前1天静脉输注激素（如甲强龙125mg）桥接治疗。

### ****五、特殊情况处理****

- **合并感染**：需先控制感染，推迟择期手术。
- **妊娠相关手术**：优先胎儿安全，停用致畸药物（如MTX、来氟米特）。
- **药物过敏反应**：术前需记录过敏史，避免交叉过敏（如TNF抑制剂间过敏）。

最终决策需结合手术紧迫性、疾病活动度及感染风险，建议在多学科团队指导下动态调整方案。

37.

### ****一、可安全合用的药物组合****

1. **生物制剂（如TNF抑制剂） + 对乙酰氨基酚**
   1. 无直接相互作用，可短期联用控制发热/疼痛。
2. **传统DMARDs（如甲氨蝶呤） + 伪麻黄碱**
   1. 短期缓解鼻塞，但需监测血压（伪麻黄碱可能升高血压）。
3. **激素（如泼尼松） + 氯雷他定**
   1. 抗组胺药缓解流涕/打喷嚏，无显著相互作用。

### ****二、需警惕的风险组合****

1. **NSAIDs（如塞来昔布） + 布洛芬/阿司匹林**
   1. **风险**：胃肠道出血风险叠加（尤其是高龄或溃疡病史者）。
   2. **建议**：避免联用，或换用对乙酰氨基酚。
2. **激素 + 对乙酰氨基酚长期联用**
   1. **风险**：肝毒性风险增加（尤其日剂量≥4g对乙酰氨基酚）。
   2. **建议**：短期联用（≤3天）且对乙酰氨基酚日剂量≤3g。
3. **生物制剂 + 复方感冒药（含布洛芬/阿司匹林）**
   1. **风险**：NSAIDs可能掩盖感染症状（如发热），延误感冒并发症（如肺炎）诊断。
   2. **建议**：优先选无NSAIDs成分的感冒药（如含右美沙芬+氯雷他定）。

### ****三、特殊注意事项****

1. **肝肾功能监测**
   1. AS患者长期用药可能已有肝肾负担，感冒期间需短期复查肝肾功能。
2. **感染预警**
   1. 生物制剂/激素使用者感冒后如出现黄痰、胸痛或持续发热，需立即排查感染。
3. **血压管理**
   1. 含伪麻黄碱的感冒药可能升高血压，与激素联用需加强血压监测。

### ****四、安全用药流程****

1. **核对成分**：优先选择单一成分感冒药（如仅含对乙酰氨基酚）。
2. **控制疗程**：感冒药连续使用≤5天，症状未缓解需就医。
3. **间隔服用**：AS药物与感冒药间隔1-2小时服用，减少胃肠刺激。
4. **咨询药师**：联用前向药师提供完整用药清单（包括中药/保健品）。

### ****五、替代方案****

- **鼻塞/流涕**：生理盐水冲洗鼻腔（无药物相互作用）。
- **咽痛**：温盐水漱口+含片（如西地碘片）。
- **咳嗽**：蜂蜜（1岁以上）或右美沙芬（无痰干咳）。

建议将当前AS用药清单拍照保存，就诊时向医生/药师展示，以确保用药安全。

38.

### ****一、疾病与妊娠的相互影响****

1. **疾病活动度**
   1. **孕期可能缓解**：约50%-70%患者孕期症状减轻（与雌激素升高相关），但产后3-6个月可能复发。
   2. **风险预警**：高疾病活动度（如BASDAI≥4分）可能增加早产、低体重儿风险。
2. **妊娠并发症**
   1. **早产风险**：AS患者早产率较健康人群高1.5-2倍，需加强孕期监测。
   2. **剖宫产率**：因髋关节活动受限或脊柱融合，剖宫产率可能升高（约30%-40%）。

### ****二、药物安全性分层管理****

| **药物类别** | **备孕/孕期使用建议** |
| --- | --- |
| **NSAIDs** | 孕晚期避免（可能导致胎儿动脉导管早闭）；孕早期短期使用相对安全（如塞来昔布） |
| **DMARDs** | 甲氨蝶呤/来氟米特：备孕前3个月停用，来氟米特需药物洗脱（消胆胺8g tid×11天） |
| **生物制剂** | TNF抑制剂（如依那西普、阿达木单抗）：多数指南推荐孕期继续使用（需知情同意） |
| **激素** | 泼尼松/甲泼尼龙：孕期可使用（剂量≤20mg/日泼尼松等效剂量），但需监测血糖、血压 |
| **沙利度胺** | 绝对禁忌（致畸性极高） |

### ****三、备孕前准备流程****

1. **疾病评估**：
   1. 确保病情稳定（如ESR/CRP正常，无活动性炎症）。
   2. 调整治疗方案（如停用致畸药物，换用生物制剂）。
2. **药物调整**：
   1. **提前3个月**：停用甲氨蝶呤/来氟米特，换用生物制剂或硫唑嘌呤（需权衡利弊）。
   2. **补充叶酸**：备孕前3个月开始（5mg/日），避免与甲氨蝶呤同时使用。
3. **多学科会诊**：
   1. 风湿免疫科、妇产科、产科共同制定孕期管理计划。

### ****四、孕期监测重点****

1. **疾病活动度**：
   1. 每1-2个月复查炎症指标（ESR/CRP），必要时调整治疗。
   2. 出现新发炎症（如夜间背痛、晨僵加重）及时就诊。
2. **胎儿监测**：
   1. 加强超声随访（尤其是心脏结构，部分生物制剂可能增加胎儿心律失常风险）。
   2. 孕晚期关注胎儿生长速度（预防宫内生长受限）。
3. **药物监测**：
   1. 使用TNF抑制剂者：检测药物血药浓度（如阿达木单抗），避免过度免疫抑制。
   2. 激素使用者：监测血糖、血压及骨密度。

### ****五、产后管理策略****

1. **疾病复发预防**：
   1. 产后立即恢复生物制剂或DMARDs（如甲氨蝶呤），避免哺乳期间疾病复发。
   2. 如需哺乳：优先选经乳汁分泌少的药物（如阿达木单抗＞依那西普）。
2. **康复支持**：
   1. 产后早期康复锻炼（如脊柱灵活性训练），预防关节僵硬加重。
   2. 心理支持（AS患者产后抑郁风险较高）。

### ****六、特殊情况处理****

- **辅助生殖技术**：使用促排卵药物时需调整AS治疗方案（如暂停生物制剂）。
- **男性患者备孕**：多数AS药物对精子影响小，但建议备孕前咨询生殖医学专家。

在充分准备和多学科协作下，多数AS患者可安全度过孕期。建议尽早规划，与医生共同制定个体化方案。

39.

### ****一、药物分类调整原则****

#### ****1. 需提前停用的药物****

- **甲氨蝶呤（MTX）**
  - **停药时机**：备孕前3个月停用（男性患者建议同周期）。
  - **替代方案**：换用生物制剂（如阿达木单抗）或硫唑嘌呤（需评估风险）。
- **来氟米特（LEF）**
  - **洗脱流程**：停药后立即使用消胆胺8g tid（每日三次），连续11天，加速药物清除。
  - **验证方法**：洗脱后检测血浆药物浓度（需＜0.02mg/L）。

#### ****2. 可继续使用的药物****

- **TNF抑制剂（如依那西普、阿达木单抗）**
  - **孕期使用**：多数指南推荐继续使用（需签署知情同意书）。
  - **剂量调整**：孕晚期（28周后）可考虑减半剂量（如阿达木单抗每2周20mg）。
- **激素（泼尼松/甲泼尼龙）**
  - **安全剂量**：≤20mg/日泼尼松等效剂量。
  - **监测重点**：血糖、血压及骨密度变化。
- **NSAIDs（如塞来昔布）**
  - **短期使用**：排卵后2周内（受精卵着床前）相对安全。
  - **限制**：避免在孕6周后使用（可能致胎儿动脉导管早闭）。

#### ****3. 绝对禁忌药物****

- **沙利度胺**：致畸性极高，备孕前至少6个月停用，需严格避孕。

### ****二、分阶段调整流程****

| **阶段** | **时间节点** | **关键行动** |
| --- | --- | --- |
| **备孕前3个月** | 停药+洗脱 | 停MTX/LEF，启动消胆胺洗脱；换用生物制剂；补充叶酸5mg/日（避免与MTX同时使用） |
| **备孕前1个月** | 方案确认 | 多学科会诊（风湿免疫科+妇产科+产科），确认孕期监测计划 |
| **排卵后2周** | 着床窗口期 | 避免新启动免疫抑制剂（如硫唑嘌呤）；NSAIDs短期使用需权衡利弊 |
| **确认妊娠后** | 孕4-6周 | 复查炎症指标（ESR/CRP），调整生物制剂剂量；建立产科随访档案 |

### ****三、特殊场景处理****

1. **疾病复发风险**
   - **替代方案**：如无法使用生物制剂，可短期使用激素（≤20mg/日）或关节腔注射激素。
   - **禁忌**：避免使用环孢素/他克莫司（致畸风险高）。
2. **辅助生殖技术**
   - **药物协调**：促排卵期间暂停生物制剂（如阿达木单抗），移植后根据胚胎着床情况恢复。
   - **多学科协作**：生殖医学中心与风湿科医生共同制定用药日历。
3. **男性患者备孕**
   - **药物影响**：多数AS药物对精子影响较小，但建议备孕前咨询生殖医学专家。
   - **替代方案**：如使用沙利度胺，需停药6个月以上。

### ****四、监测与应急计划****

- **疾病活动度**：每4-8周监测BASDAI、ASDAS评分，及时调整治疗。
- **胎儿监测**：孕20周后每4周超声检查（关注心脏结构及生长曲线）。
- **应急药物包**：准备低剂量激素（如泼尼松5mg）用于突发疼痛或炎症。

40.

### ****一、西医治疗的核心地位****

1. **循证医学证据明确**
   1. **NSAIDs**（如塞来昔布）：快速控制炎症性疼痛，是国际指南一线推荐。
   2. **生物制剂**（如TNF抑制剂）：针对AS病理机制（如阿达木单抗），可延缓影像学进展。
   3. **DMARDs**（如甲氨蝶呤）：长期控制病情，降低复发率。
2. **不可替代性**
   1. 西药的作用机制清晰，疗效经过大量临床试验验证。
   2. 擅自停用可能导致疾病活动度反弹（如ESR/CRP升高、新骨形成加速）。

### ****二、中药的辅助作用与局限性****

1. **可能益处**
   1. **抗炎调节**：部分中药（如雷公藤、白芍总苷）可能具有免疫调节作用，但需辨证施治。
   2. **症状缓解**：如改善疲劳、睡眠障碍等伴随症状。
2. **关键风险**
   1. **肝肾毒性**：部分中药成分（如何首乌、马兜铃酸类）可能诱发肝损伤或肾间质纤维化。
   2. **药物相互作用**：与西药联用可能增加不良反应（如华法林与丹参的出血风险）。
   3. **缺乏标准化**：中药方剂成分复杂，疗效受药材质量、炮制工艺影响大。

### ****三、针灸的适用场景****

1. **短期症状改善**
   1. **镇痛效果**：针灸可能通过调节神经递质（如内啡肽）缓解肌肉痉挛和疼痛。
   2. **改善功能**：辅助缓解晨僵、增加关节活动度。
2. **局限性**
   1. **无法控制炎症**：针灸不能抑制AS的核心病理过程（如附着点炎、骨破坏）。
   2. **个体差异大**：疗效与患者体质、操作者手法密切相关。

### ****四、综合决策建议****

1. **优先保障西药治疗**
   1. 在风湿免疫科医生指导下，规范使用NSAIDs、生物制剂或DMARDs。
   2. 定期复查炎症指标（如ESR/CRP）和影像学（如骶髂关节MRI）。
2. **谨慎选择中医辅助**
   1. **中药使用原则**：
      1. 避免含毒性成分（如雷公藤需严格监测血常规）。
      2. 与西药间隔1-2小时服用，减少相互作用。
   2. **针灸注意事项**：
      1. 选择正规医院针灸科，避免感染风险。
      2. 急性期避免过度刺激（如电针强度需控制）。
3. **多学科协作**
   1. 联合风湿免疫科、中医科制定个体化方案，动态调整治疗策略。

### ****五、需警惕的误区****

- **“中药无副作用”**：部分中药可能导致肝衰竭（如吡咯里西啶生物碱类）。
- **“针灸根治AS”**：目前AS无法根治，所有治疗以控制病情为目标。
- **自行停药**：突然停用生物制剂可能导致疾病反弹，需在医生指导下调整。

建议将中医方案作为提高生活质量的补充，而非替代核心治疗，尤其在疾病活动期（BASDAI≥4分）需优先控制炎症。

41.

### ****一、锻炼的核心价值****

1. **延缓脊柱强直**
   1. 通过关节活动度训练（如脊柱伸展、侧弯运动），维持关节灵活性。
2. **改善肌肉失衡**
   1. 针对性强化核心肌群（如背肌、腹肌）及下肢力量，纠正姿势异常。
3. **调节炎症循环**
   1. 适度运动可促进血液循环，帮助清除炎症因子（如TNF-α）。

### ****二、分阶段锻炼策略****

#### ****1. 急性期（BASDAI≥4分，夜间痛醒）****

- **以休息为主**：避免负重或高强度运动。
- **温和拉伸**：每日短时（10-15分钟）进行仰卧抱膝、猫牛式等轻柔动作。
- **辅助手段**：使用热敷或温水泳池中漂浮放松。

#### ****2. 稳定期（炎症指标正常，疼痛可控）****

- **运动类型选择**：
  - **低冲击有氧**：游泳（蛙泳/自由泳）、骑自行车。
  - **灵活性训练**：瑜伽（避免过度后弯）、普拉提。
  - **力量训练**：弹力带抗阻训练（重点加强背肌、臀肌）。
- **频率与强度**：
  - 每周3-5次，每次30-45分钟，心率控制在（220-年龄）×60%。
  - 循序渐进增加强度，避免突然剧烈运动。

### ****三、关键注意事项****

1. **避免禁忌动作**：
   - 高冲击运动（如跑步、跳跃）、过度前屈（可能加重胸椎炎症）。
   - 负重深蹲（增加骶髂关节压力）。
2. **疼痛管理**：
   - 锻炼前口服NSAIDs（如塞来昔布）预防疼痛加重。
   - 运动后冰敷炎症关节（每次15分钟）。
3. **个性化调整**：
   - 脊柱融合患者需侧重非负重运动（如上肢力量训练）。
   - 合并髋关节炎者避免深蹲，改用坐位腿伸展。

### ****四、协同治疗建议****

- **药物+运动**：在生物制剂（如阿达木单抗）控制炎症基础上，锻炼效果更显著。
- **康复评估**：每3-6个月由物理治疗师调整运动方案。
- **心理支持**：慢性疼痛可能影响运动依从性，必要时联合心理干预。

42.

### ****一、手术适用场景****

#### ****1. 关节严重破坏****

- **髋关节受累**：
  - 当髋关节出现骨性强直（X线显示关节间隙消失）、疼痛严重且影响行走时，需行**全髋关节置换术**。
  - 适应症：BASFI（功能指数）评分高、NSAIDs及生物制剂无法控制症状。
- **膝关节受累**：
  - 严重关节炎导致畸形或功能丧失时，可考虑**膝关节置换术**。

#### ****2. 脊柱畸形矫正****

- **脊柱后凸（驼背）**：
  - Cobb角＞40°，影响呼吸功能（如肺活量下降）或平衡能力时，需**脊柱截骨矫形术**。
  - 手术风险高，需严格评估心肺功能。

#### ****3. 骨折或神经损伤****

- **脆性骨折**：长期炎症导致骨量减少（如使用糖皮质激素），轻微外伤即可引发脊柱或股骨颈骨折。
- **脊髓压迫**：脊柱韧带骨化（如竹林样改变）压迫神经时，需紧急手术减压。

### ****二、为何手术非首选？****

1. **保守治疗优先**：
   - **生物制剂**（如TNF抑制剂）可延缓关节破坏，早期使用可降低手术需求。
   - **抗炎镇痛**（NSAIDs）联合**功能锻炼**可维持关节活动度。
2. **手术风险与局限性**：
   - **感染风险**：AS患者常伴免疫功能异常，术后感染概率较高。
   - **假体松动**：骨质量下降可能影响关节置换术长期效果。
   - **脊柱手术并发症**：矫形术后可能出现神经功能损伤或矫正角度丢失。

### ****三、如何降低手术风险？****

1. **早期规范治疗**：
   - 在疾病活动期（BASDAI≥4分）及时使用生物制剂控制炎症。
   - 定期监测影像学（如骶髂关节MRI、脊柱X线）以评估结构进展。
2. **多学科协作**：
   - 联合风湿免疫科、骨科、康复科制定个体化方案。
   - 术前需评估心肺功能（如肺功能测试、心脏超声）。
3. **术后管理**：
   - 关节置换术后需坚持抗凝治疗（预防血栓）、康复训练。
   - 脊柱术后需佩戴支具3-6个月，逐步增加活动量。

### ****四、患者行动指南****

✅ **重点监测**：

- 若出现关节持续固定性疼痛、行走距离＜500米，需警惕关节破坏，及时就诊。
- 驼背角度进行性加重（每年＞5°）需评估手术必要性。
  ⚠️ **风险规避**：
- 避免未评估的剧烈运动（如过度拉伸）以防骨折。
- 长期使用激素者需补充钙剂及维生素D。
  💡 **生活调整**：
- 使用辅助工具（如长柄取物器）减少弯腰动作。
- 睡眠时使用低枕或颈椎枕保持脊柱中立位。
