# Supplementary material for: Assessing the Quality of AI Responses to Patient Concerns About Axial Spondyloarthritis: Delphi-Based Evaluation
Source: JMIR AI. 2026 Jan 7;5:e79153. doi: 10.2196/79153 (PMC12824573; doi:10.2196/79153)

Figure S1. Flow diagram

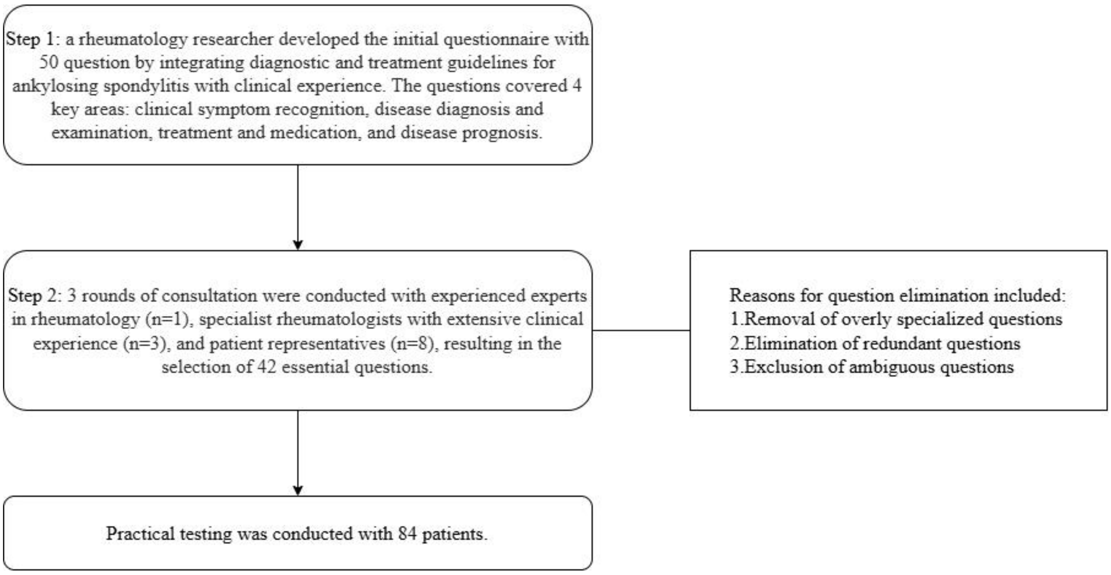

Figure S2. Rater Agreement Matrix with Unweighted and Linear Weighted Kappa Coefficients

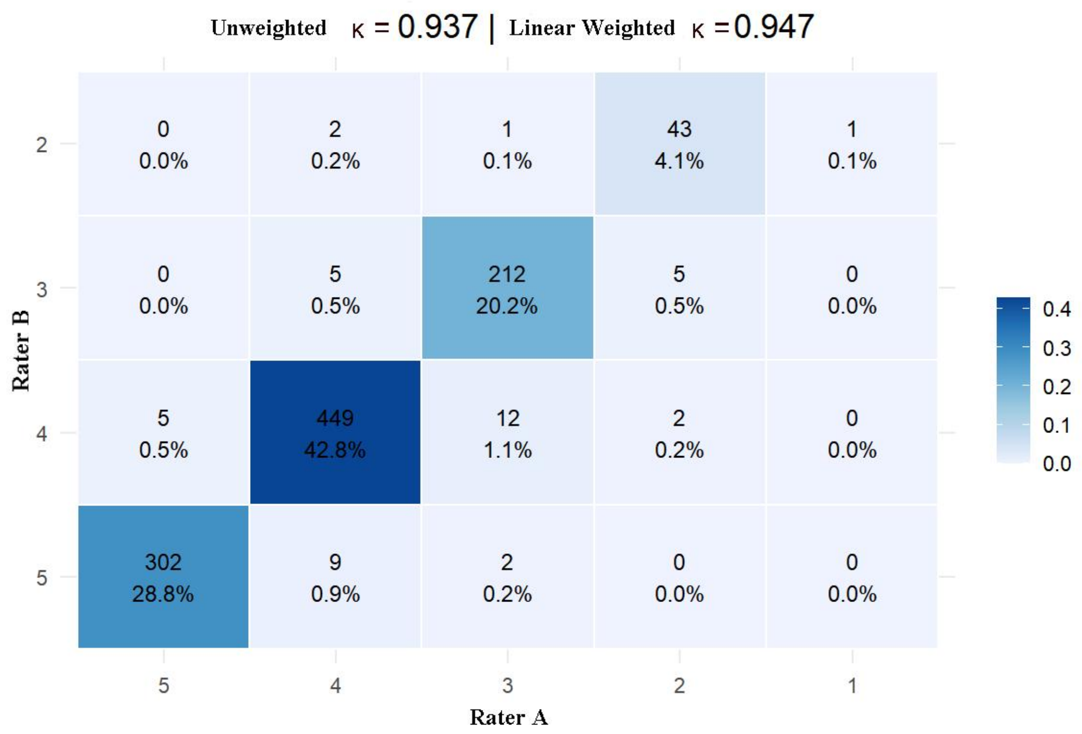

Figure S3. Inter-Model Comparison Heatmap Matrix for Multi-Dimensional Linguistic Metrics

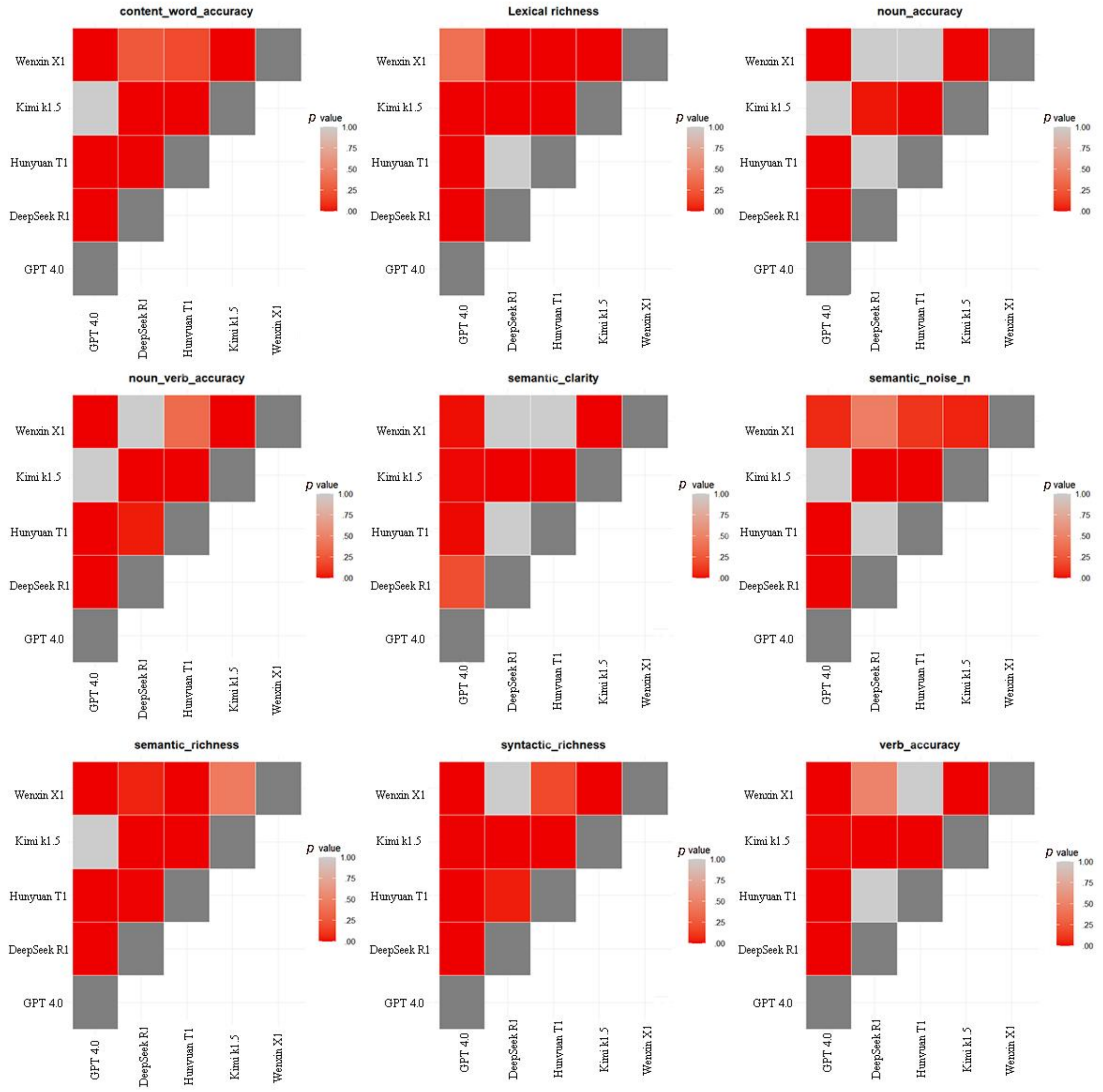

Figure S4. Disclaimers About Health Advice

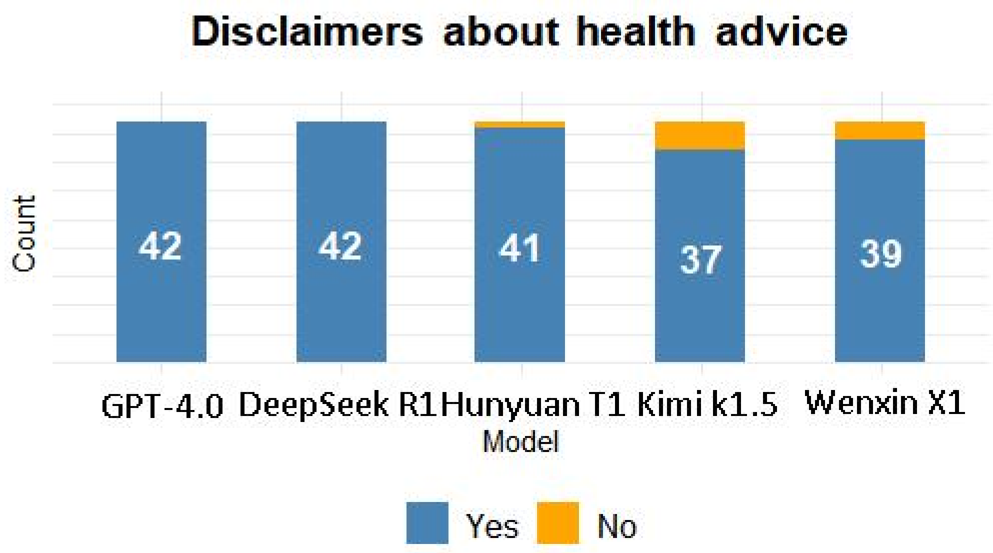

Supplement: Multimedia Appendix 4 [file ai_v5i1e79153_app4.pdf]
