# Supplementary material for: Assessing the Quality of AI Responses to Patient Concerns About Axial Spondyloarthritis: Delphi-Based Evaluation
Source: JMIR AI. 2026 Jan 7;5:e79153. doi: 10.2196/79153 (PMC12824573; doi:10.2196/79153)
Supplement: Multimedia Appendix 5 [file ai_v5i1e79153_app5.doc]

**Supplement Table3. Specific Results of the Chi-Square Test in Figure 2A.**

| **Question** | **ChiSq** | **p.value** |
| --- | --- | --- |
| 1 | 2.089493588 | 0.603339666 |
| 2 | 6.950390983 | 0.104889511 |
| 3 | 3.33717442 | 0.544145585 |
| 4 | 11.77557701 | 0.01229877 |
| 5 | 3.092870155 | 0.404259574 |
| 6 | 2.151742851 | 0.807819218 |
| 7 | 4.324547804 | 0.393460654 |
| 8 | 0.496708438 | 0.96330367 |
| 9 | 0.64 | 0.95610439 |
| 10 | 1.002170983 | 0.97330267 |
| 11 | 1.220117964 | 0.685531447 |
| 12 | 3.962446246 | 0.246275372 |
| 13 | 8.18349338 | 0.02289771 |
| 14 | 2.859158843 | 0.432756724 |
| 15 | 1.673625245 | 0.943005699 |
| 16 | 1.532168826 | 0.742225777 |
| 17 | 9.445128991 | 0.04259574 |
| 18 | 2.948935134 | 0.411058894 |
| 19 | 2.957781082 | 0.607139286 |
| 20 | 8.742141976 | 0.050094991 |
| 21 | 4.894905181 | 0.311868813 |
| 22 | 3.935562077 | 0.299370063 |
| 23 | 7.32070248 | 0.077892211 |
| 24 | 6.461080385 | 0.052694731 |
| 25 | 6.8144733 | 0.117488251 |
| 26 | 1.780614328 | 0.640035996 |
| 27 | 7.726789823 | 0.0369963 |
| 28 | 13.18219195 | 0.00559944 |
| 29 | 5.873567473 | 0.197380262 |
| 30 | 13.51300611 | 0.00149985 |
| 31 | 12.94235008 | 0.00339966 |
| 32 | 4.039539205 | 0.425257474 |
| 33 | 5.841527232 | 0.113688631 |
| 34 | 5.90579719 | 0.146585341 |
| 35 | 4.835894105 | 0.312568743 |
| 36 | 12.00319021 | 0.00849915 |
| 37 | 9.98854834 | 0.01539846 |
| 38 | 13.1333833 | 0.00579942 |
| 39 | 7.114972016 | 0.117288271 |
| 40 | 12.70191394 | 0.00769923 |
| 41 | 6.252437795 | 0.087391261 |
| 42 | 5.547512786 | 0.135386461 |
