# Supplementary material for: Assessing the Quality of AI Responses to Patient Concerns About Axial Spondyloarthritis: Delphi-Based Evaluation
Source: JMIR AI. 2026 Jan 7;5:e79153. doi: 10.2196/79153 (PMC12824573; doi:10.2196/79153)
Supplement: Multimedia Appendix 6 [file ai_v5i1e79153_app6.doc]

**Supplement Table4. Specific Results of the Chi-Square Test in Figure 3A.**

| **Question** | **ChiSq** | **p.value** |
| --- | --- | --- |
| 1 | 6.429336641 | 0.080591941 |
| 2 | 1.697810543 | 0.861313869 |
| 3 | 3.593963532 | 0.460053995 |
| 4 | 2.717095997 | 0.607439256 |
| 5 | 1.600096832 | 0.661133887 |
| 6 | 3.836161005 | 0.483151685 |
| 7 | 3.250110242 | 0.543845615 |
| 8 | 3.670161522 | 0.438756124 |
| 9 | 2.666152052 | 0.573742626 |
| 10 | 1.669901377 | 0.792620738 |
| 11 | 0.336691452 | 0.921807819 |
| 12 | 2.236645649 | 0.535246475 |
| 13 | 2.376291882 | 0.529947005 |
| 14 | 3.103506966 | 0.341065893 |
| 15 | 4.695237059 | 0.353664634 |
| 16 | 3.480355173 | 0.292370763 |
| 17 | 9.103363293 | 0.058494151 |
| 18 | 17.09988967 | 0.00129987 |
| 19 | 3.397551262 | 0.507649235 |
| 20 | 5.401242595 | 0.263973603 |
| 21 | 7.316252588 | 0.114188581 |
| 22 | 5.957065376 | 0.207279272 |
| 23 | 7.213805394 | 0.112088791 |
| 24 | 1.974914109 | 0.533146685 |
| 25 | 5.30192768 | 0.274272573 |
| 26 | 10.98759031 | 0.01349865 |
| 27 | 3.47727792 | 0.306169383 |
| 28 | 3.479079663 | 0.524547545 |
| 29 | 4.061935406 | 0.435056494 |
| 30 | 4.485738676 | 0.363363664 |
| 31 | 14.40604007 | 0.00609939 |
| 32 | 7.776302244 | 0.108189181 |
| 33 | 4.394423541 | 0.222377762 |
| 34 | 5.881554551 | 0.200879912 |
| 35 | 8.499217341 | 0.078592141 |
| 36 | 2.821043904 | 0.614838516 |
| 37 | 4.08699335 | 0.242975702 |
| 38 | 2.662373469 | 0.629437056 |
| 39 | 4.433859152 | 0.318968103 |
| 40 | 6.240151016 | 0.167483252 |
| 41 | 2.235488925 | 0.525247475 |
| 42 | 6.395648823 | 0.087491251 |
