# Supplementary material for: Assessing the Quality of AI Responses to Patient Concerns About Axial Spondyloarthritis: Delphi-Based Evaluation
Source: JMIR AI. 2026 Jan 7;5:e79153. doi: 10.2196/79153 (PMC12824573; doi:10.2196/79153)
Supplement: Multimedia Appendix 7 [file ai_v5i1e79153_app7.doc]

**Supplement Table 5. The scoring results of the various models**

| **Module** | **LLMs** | **Mean Inaccurate or inappropriate content** | **Mean Omissions** | **Mean Likelyhood of harm** | **Mean Extent of harm** | **Mean Bias** |
| --- | --- | --- | --- | --- | --- | --- |
| **Treatment and Medication** | **DS** | **3.79** | **3.71** | **4.00** | **4.79** | **4.71** |
| **Treatment and Medication** | **GPT** | **3.57** | **4.14** | **4.00** | **4.64** | **4.86** |
| **Treatment and Medication** | **HY** | **2.57** | **4.07** | **3.21** | **3.79** | **4.36** |
| **Treatment and Medication** | **KM** | **2.93** | **2.93** | **3.21** | **3.57** | **4.36** |
| **Treatment and Medication** | **WX** | **3.07** | **4.07** | **3.71** | **4.14** | **4.36** |
| **Symptoms and Etiology** | **DS** | **3.80** | **4.40** | **4.70** | **4.80** | **4.80** |
| **Symptoms and Etiology** | **GPT** | **4.10** | **4.10** | **4.50** | **4.70** | **4.50** |
| **Symptoms and Etiology** | **HY** | **2.80** | **4.40** | **4.10** | **4.40** | **4.50** |
| **Symptoms and Etiology** | **KM** | **2.60** | **2.60** | **3.20** | **3.70** | **4.70** |
| **Symptoms and Etiology** | **WX** | **2.90** | **3.80** | **3.80** | **4.50** | **4.20** |
| **Diagnosis and Examination** | **DS** | **3.83** | **4.25** | **4.33** | **4.58** | **4.50** |
| **Diagnosis and Examination** | **GPT** | **4.00** | **4.17** | **4.33** | **4.67** | **4.42** |
| **Diagnosis and Examination** | **HY** | **3.08** | **4.33** | **3.83** | **4.17** | **4.33** |
| **Diagnosis and Examination** | **KM** | **3.33** | **3.58** | **3.92** | **4.50** | **4.42** |
| **Diagnosis and Examination** | **WX** | **3.25** | **3.58** | **3.75** | **4.25** | **4.58** |
| **Prognosis and Rehabilitation** | **DS** | **3.83** | **4.17** | **3.83** | **4.67** | **4.67** |
| **Prognosis and Rehabilitation** | **GPT** | **3.67** | **4.50** | **3.67** | **4.50** | **4.67** |
| **Prognosis and Rehabilitation** | **HY** | **3.00** | **4.83** | **3.50** | **4.00** | **4.17** |
| **Prognosis and Rehabilitation** | **KM** | **3.17** | **3.17** | **3.33** | **3.50** | **4.50** |
| **Prognosis and Rehabilitation** | **WX** | **3.17** | **4.17** | **3.83** | **4.17** | **4.50** |
