# Supplementary material for: Assessing the Quality of AI Responses to Patient Concerns About Axial Spondyloarthritis: Delphi-Based Evaluation
Source: JMIR AI. 2026 Jan 7;5:e79153. doi: 10.2196/79153 (PMC12824573; doi:10.2196/79153)
Supplement: Multimedia Appendix 8 [file ai_v5i1e79153_app8.doc]

**Supplement Table 6. Specific Results of the Chi-Square Test in Figure S3.**

A.

| **contrast** | **estimate** | **SE** | **df** | **t.ratio** | **p.value** |
| --- | --- | --- | --- | --- | --- |
| Wenxin - Hunyuan | 0.227550865 | 0.092903471 | 40 | 2.449325777 | 0.187877728 |
| Wenxin - DS | -0.135598893 | 0.058199231 | 40 | -2.329908677 | 0.249430895 |
| Wenxin - ChatGPT 4.0 | -0.753138286 | 0.065585698 | 40 | -11.48327005 | < .001 |
| Wenxin - Kimi | -0.856862525 | 0.075153316 | 40 | -11.40152649 | < .001 |
| Hunyuan - DS | -0.363149758 | 0.088710013 | 40 | -4.0936727 | 0.002003246 |
| Hunyuan - ChatGPT 4.0 | -0.980689151 | 0.086734454 | 40 | -11.30680026 | < .001 |
| Hunyuan - Kimi | -1.084413391 | 0.104639767 | 40 | -10.36330092 | < .001 |
| DS - ChatGPT 4.0 | -0.617539393 | 0.065911011 | 40 | -9.369290201 | < .001 |
| DS - Kimi | -0.721263632 | 0.070405804 | 40 | -10.24437749 | < .001 |
| ChatGPT 4.0 - Kimi | -0.10372424 | 0.067732543 | 40 | -1.531379673 | 1 |

B.

| **contrast** | **estimate** | **SE** | **df** | **t.ratio** | **p.value** |
| --- | --- | --- | --- | --- | --- |
| Kimi - ChatGPT 4.0 | -0.3799 | 0.0333 | 40 | -11.3999 | < .001 |
| Kimi - DS | -0.5915 | 0.0329 | 40 | -18.0062 | < .001 |
| Kimi - Wenin | -0.4311 | 0.0377 | 40 | -11.4218 | < .001 |
| Kimi - Hunyuan | -0.5793 | 0.0321 | 40 | -18.0474 | < .001 |
| ChatGPT 4.0 - DS | -0.2116 | 0.0242 | 40 | -8.7566 | < .001 |
| ChatGPT 4.0 - Wenin | -0.0513 | 0.0239 | 40 | -2.1432 | 0.3823 |
| ChatGPT 4.0 - Hunyuan | -0.1994 | 0.0262 | 40 | -7.621 | < .001 |
| DS - Wenin | 0.1604 | 0.0284 | 40 | 5.6391 | < .001 |
| DS - Hunyuan | 0.0123 | 0.0205 | 40 | 0.599 | 1 |
| Wenin - Hunyuan | -0.1481 | 0.0304 | 40 | -4.8709 | < .001 |

C.

| **contrast** | **estimate** | **SE** | **df** | **t.ratio** | **p.value** |
| --- | --- | --- | --- | --- | --- |
| Wenxin - DS | -0.042 | 0.0606 | 40 | -0.6926 | 1 |
| Wenxin - Hunyuan | 0.0222 | 0.0716 | 40 | 0.3095 | 1 |
| Wenxin - Kimi | -0.3317 | 0.0825 | 40 | -4.0217 | 0.0025 |
| Wenxin - ChatGPT 4.0 | -0.4493 | 0.0784 | 40 | -5.7285 | < .001 |
| DS - Hunyuan | 0.0642 | 0.0688 | 40 | 0.9331 | 1 |
| DS - Kimi | -0.2897 | 0.0898 | 40 | -3.2276 | 0.0249 |
| DS - ChatGPT 4.0 | -0.4073 | 0.067 | 40 | -6.0813 | < .001 |
| Hunyuan - Kimi | -0.3539 | 0.0899 | 40 | -3.9368 | 0.0032 |
| Hunyuan - ChatGPT 4.0 | -0.4715 | 0.0826 | 40 | -5.7076 | < .001 |
| Kimi - ChatGPT 4.0 | -0.1176 | 0.0871 | 40 | -1.3506 | 1 |

D.

| **contrast** | **estimate** | **SE** | **df** | **t.ratio** | **p.value** |
| --- | --- | --- | --- | --- | --- |
| Wenxin - Hunyuan | 0.199379851 | 0.091709894 | 40 | 2.174027719 | 0.356784747 |
| Wenxin - DS | -0.103093661 | 0.064967577 | 40 | -1.58684787 | 1 |
| Wenxin - ChatGPT 4.0 | -0.814078973 | 0.064762526 | 40 | -12.57021645 | < .001 |
| Wenxin - Kimi | -0.9015642 | 0.086349382 | 40 | -10.44088773 | < .001 |
| Hunyuan - DS | -0.302473511 | 0.097299716 | 40 | -3.108678248 | 0.034545141 |
| Hunyuan - ChatGPT 4.0 | -1.013458823 | 0.089513868 | 40 | -11.32180799 | < .001 |
| Hunyuan - Kimi | -1.100944051 | 0.113847516 | 40 | -9.670338806 | < .001 |
| DS - ChatGPT 4.0 | -0.710985312 | 0.072006131 | 40 | -9.873955227 | < .001 |
| DS - Kimi | -0.798470539 | 0.085417264 | 40 | -9.347882395 | < .001 |
| ChatGPT 4.0 - Kimi | -0.087485227 | 0.076553173 | 40 | -1.142803407 | 1 |

E.

| **contrast** | **estimate** | **SE** | **df** | **t.ratio** | **p.value** |
| --- | --- | --- | --- | --- | --- |
| Hunyuan - Wenxin | -0.0031 | 0.0043 | 40 | -0.7278 | 1 |
| Hunyuan - DS | -0.0063 | 0.0039 | 40 | -1.5995 | 1 |
| Hunyuan - ChatGPT 4.0 | -0.0157 | 0.0043 | 40 | -3.6535 | 0.0074 |
| Hunyuan - Kimi | -0.0336 | 0.0052 | 40 | -6.4606 | < .001 |
| Wenxin - DS | -0.0031 | 0.0031 | 40 | -0.991 | 1 |
| Wenxin - ChatGPT 4.0 | -0.0125 | 0.0036 | 40 | -3.4401 | 0.0137 |
| Wenxin - Kimi | -0.0304 | 0.0055 | 40 | -5.4887 | < .001 |
| DS - ChatGPT 4.0 | -0.0094 | 0.0039 | 40 | -2.4161 | 0.2035 |
| DS - Kimi | -0.0273 | 0.0053 | 40 | -5.1334 | < .001 |
| ChatGPT 4.0 - Kimi | -0.0179 | 0.0048 | 40 | -3.716 | 0.0062 |

F.

| **contrast** | **estimate** | **SE** | **df** | **t.ratio** | **p.value** |
| --- | --- | --- | --- | --- | --- |
| Hunyuan - Wenxin | 4.8955 | 1.8189 | 40 | 2.6914 | 0.1034 |
| Hunyuan - DS | 2.2988 | 2.1513 | 40 | 1.0686 | 1 |
| Hunyuan - Kimi | 10.288 | 1.9005 | 40 | 5.4133 | < .001 |
| Hunyuan - ChatGPT 4.0 | 8.6285 | 1.5491 | 40 | 5.57 | < .001 |
| Wenxin - DS | -2.5967 | 1.2663 | 40 | -2.0506 | 0.469 |
| Wenxin - Kimi | 5.3925 | 1.8181 | 40 | 2.9659 | 0.0507 |
| Wenxin - ChatGPT 4.0 | 3.733 | 1.3049 | 40 | 2.8607 | 0.0669 |
| DS - Kimi | 7.9892 | 1.9401 | 40 | 4.1179 | 0.0019 |
| DS - ChatGPT 4.0 | 6.3297 | 1.5635 | 40 | 4.0484 | 0.0023 |
| Kimi - ChatGPT 4.0 | -1.6595 | 1.1516 | 40 | -1.4411 | 1 |

G.

| **contrast** | **estimate** | **SE** | **df** | **t.ratio** | **p.value** |
| --- | --- | --- | --- | --- | --- |
| DS - ChatGPT 4.0 | 0.046 | 0.0052 | 40 | 8.8074 | < .001 |
| DS - Kimi | 0.0388 | 0.0049 | 40 | 7.9035 | < .001 |
| DS - Hunyuan | -0.0526 | 0.0133 | 40 | -3.9648 | 0.003 |
| DS - Wenxin | -0.0215 | 0.0073 | 40 | -2.9549 | 0.0522 |
| ChatGPT 4.0 - Kimi | -0.0072 | 0.0052 | 40 | -1.4018 | 1 |
| ChatGPT 4.0 - Hunyuan | -0.0986 | 0.0121 | 40 | -8.1788 | < .001 |
| ChatGPT 4.0 - Wenxin | -0.0675 | 0.0062 | 40 | -10.8328 | < .001 |
| Kimi - Hunyuan | -0.0914 | 0.0128 | 40 | -7.1189 | < .001 |
| Kimi - Wenxin | -0.0603 | 0.0069 | 40 | -8.7616 | < .001 |
| Hunyuan - Wenxin | 0.0311 | 0.015 | 40 | 2.0687 | 0.4508 |

H.

| **contrast** | **estimate** | **SE** | **df** | **t.ratio** | **p.value** |
| --- | --- | --- | --- | --- | --- |
| DS - Hunyuan | 0.0842 | 0.0272 | 40 | 3.0949 | 0.0359 |
| DS - Wenxin | 0.0106 | 0.0109 | 40 | 0.9717 | 1 |
| DS - Kimi | -0.0586 | 0.0096 | 40 | -6.0915 | < .001 |
| DS - ChatGPT 4.0 | -0.091 | 0.0077 | 40 | -11.774 | < .001 |
| Hunyuan - Wenxin | -0.0736 | 0.0298 | 40 | -2.4722 | 0.1778 |
| Hunyuan - Kimi | -0.1428 | 0.0242 | 40 | -5.9038 | < .001 |
| Hunyuan - ChatGPT 4.0 | -0.1752 | 0.0239 | 40 | -7.3422 | < .001 |
| Wenxin - Kimi | -0.0692 | 0.014 | 40 | -4.9569 | < .001 |
| Wenxin - ChatGPT 4.0 | -0.1016 | 0.0109 | 40 | -9.3621 | < .001 |
| Kimi - ChatGPT 4.0 | -0.0324 | 0.0079 | 40 | -4.0766 | 0.0021 |

I.

| **contrast** | **estimate** | **SE** | **df** | **t.ratio** | **p.value** |
| --- | --- | --- | --- | --- | --- |
| Wenxin - Hunyuan | -0.0136 | 0.0887 | 40 | -0.1536 | 1 |
| Wenxin - DS | -0.1557 | 0.0771 | 40 | -2.0183 | 0.503 |
| Wenxin - ChatGPT 4.0 | -0.8508 | 0.0863 | 40 | -9.8566 | < .001 |
| Wenxin - Kimi | -1.2127 | 0.1215 | 40 | -9.9812 | < .001 |
| Hunyuan - DS | -0.142 | 0.1066 | 40 | -1.3318 | 1 |
| Hunyuan - ChatGPT 4.0 | -0.8371 | 0.1082 | 40 | -7.7356 | < .001 |
| Hunyuan - Kimi | -1.199 | 0.1435 | 40 | -8.3538 | < .001 |
| DS - ChatGPT 4.0 | -0.6951 | 0.1024 | 40 | -6.789 | < .001 |
| DS - Kimi | -1.057 | 0.1213 | 40 | -8.716 | < .001 |
| ChatGPT 4.0 - Kimi | -0.3619 | 0.092 | 40 | -3.9318 | 0.0033 |

a . Tables A to I represent respectively :

“content_word_accuracy”,”Lexical_richness”,”noun_accuracy”,”noun_verb_accuracy”,”semantic_clarity”,”semantic_noise_n”,”semantic_richness”,”syntactic_richness”,”verb_accuracy”.
