# Supplementary material for: Assessing the Quality of AI Responses to Patient Concerns About Axial Spondyloarthritis: Delphi-Based Evaluation
Source: JMIR AI. 2026 Jan 7;5:e79153. doi: 10.2196/79153 (PMC12824573; doi:10.2196/79153)
Supplement: Multimedia Appendix 9 [file ai_v5i1e79153_app9.doc]

**Supplement Table 7. Baseline Characteristics of Different Age Group**

| **Characteristics** | **Total (N1=52,N2=32)** | **Group 1** （n % / Mean ± SD） | **Group 2** （n % / Mean ± SD） | **p value** |
| --- | --- | --- | --- | --- |
| **Gender** |  |  |  |  |
| **Male** |  | 0.8077 | 0.625 | 0.111 |
| **Female** |  | 0.1923 | 0.375 |  |
| **Education Level** |  |  |  |  |
| **Primary school or below** |  | 0.0385 | 0.0313 | 0.4847 |
| **Junior high school** |  | 0.0962 | 0.1563 |  |
| **Senior high school** |  | 0.2308 | 0.375 |  |
| **Bachelor's degree** |  | 0.4615 | 0.3125 |  |
| **Master's degree or above** |  | 0.1731 | 0.125 |  |
| **Sedentary Occupation** |  |  |  |  |
| **Yes** |  | 0.7115 | 0.8125 | 0.1411 |
| **No** |  | 0.2885 | 0.1875 |  |
| **Parental Health Status** |  |  |  |  |
| **Good** |  | 0.6731 | 0.6876 | 0.5828 |
| **Fair** |  | 0.2692 | 0.2813 |  |
| **Poor** |  | 0.0577 | 0.0313 |  |
| **Personal Health Status** |  |  |  |  |
| **Good** |  | 0.3856 | 0.4063 | 0.4682 |
| **Fair** |  | 0.5385 | 0.4375 |  |
| **Poor** |  | 0.077 | 0.1563 |  |
| **Family History of axSpA** |  |  |  |  |
| **Yes** |  | 0.3846 | 0.2188 | 0.1802 |
| **No** |  | 0.6154 | 0.7813 |  |
| **Family History of Hereditary Diseases** |  |  |  |  |
| **Yes** |  | 0.2692 | 0.1563 | 0.3506 |
| **No** |  | 0.7308 | 0.8438 |  |
| **Daily Internet Usage Duration** |  |  |  |  |
| **Less than 6 hours** |  | 0.6154 | 0.7813 | 0.1174 |
| **More than 6 hours** |  | 0.3846 | 0.2187 |  |
